# Three Component Thio‐ and Carboboration of Alkynes: A Modular Route to Functionalised Bicyclic Boronates

**DOI:** 10.1002/anie.9548215

**Published:** 2026-05-27

**Authors:** Laura Winfrey, Gary S. Nichol, Stephen P. Thomas, Dominic R. Willcox, Michael. J. Ingleson

**Affiliations:** ^1^ EaStCHEM School of Chemistry University of Edinburgh Edinburgh UK; ^2^ Institute of Chemical Sciences Heriot‐Watt University Edinburgh UK

**Keywords:** benzoxaborinines, boron trifluoride, borylative cyclisation, carboboration, thioboration

## Abstract

The three‐component elemento‐boration of alkynes using a borane and a nucleophile is a highly efficient method to generate complex alkenyl‐boranes. Benzoxaborinines are a class of alkenyl boranes of considerable importance, including in the fight against antibiotic resistance. However, a three‐component elemento‐boration process to form functionalised benzoxaborinines was an unmet challenge before this work. Herein, we report operationally simple three‐component thio‐ and carbo‐boration reactions to form functionalised benzoxaborinines using commercially available reagents. The processes also were applicable to form functionalised benzazaborinines, which are of interest as naphthalene bioisosteres. The nucleophile scope included thioethers, thiols, and (hetero)arenes. In contrast, when using amine nucleophiles, alkyne hydroamination occurred to form boranils. Mechanistic studies revealed a disparity between thioboration using thioethers and using thiols. While thioethers are effective nucleophiles in their own right, when using thiols the in situ formation of tri‐thioboranes ((RS)_3_B) proceeded prior to thioboration, with thioborate anions ([(RS)_4_B)]^−^) calculated to be the key nucleophile. Note, combining Et_2_O∙BF_3_/RSH and a hindered base is attractive as a simple route to form tri‐thioboranes in situ. Overall, this work is a notable addition to the toolbox for making functionalised bicyclic boronates, while demonstrating that the ubiquitous borane Et_2_O·BF_3_ can still be used to discover new borylation processes.

## Graphical abstract

Three‐component thio‐ and carbo‐boration reactions to form functionalised benzoxaborinines and benzazaborinines are reported. The nucleophile scope includes thioethers, thiols, and (hetero)arenes. Mechanistic studies revealed a disparity between thioboration using thioethers and using thiols. While thioethers were effective nucleophiles, when using thiols the in situ formation of tri‐thioboranes ((RS)_3_B) proceeded prior to thioboration.

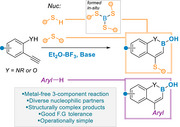

## 1 Introduction

**FIGURE 1:**
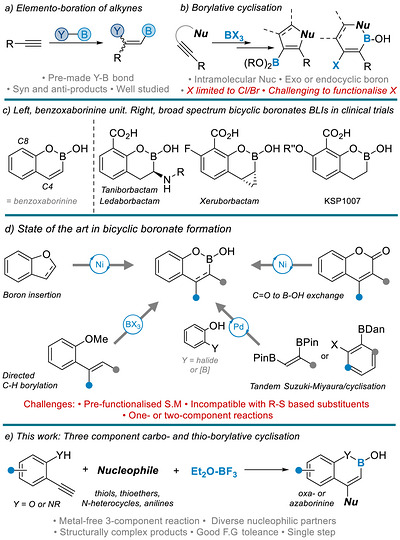
(a) Elemento‐boration of alkynes. (b) Borylative cyclisation. (c) Select bioactive bicyclic boronates. (d) State of the art in benzoxaborinine synthesis. e) This work.

The direct elemento‐boration of alkynes is a widely used method to generate alkenyl boranes [1, 2, 3, 4]. It generally uses a Lewis acidic borane and results in the syn addition of a B–Y bond to the alkyne (Figure 1a) [5]. A related class of reactions, termed borylative cyclisation, uses alkynes containing a pendant nucleophile, Nu (Figure 1b) [6]. Over the past 15 years, this has also developed into a powerful method to covert functionalised alkynes into useful (hetero)cyclic products where the boron moiety is either exo‐, or endo‐cyclic (Figure 1b) [7, 8, 9, 10, 11, 12, 13, 14]. In contrast to both of these processes, three‐component elemento‐boration reactions use a separate alkyne, nucleophile and boron source [15]. These are thus highly efficient methods to access complex products. While there has been notable metal‐mediated three‐component elemento‐borations reported [16], the area still has significant unmet challenges. For example, alkyne carboboration invariably requires pre‐functionalised hydrocarbyl units (e.g., organo‐halides or organo‐boranes) [16, 17], a carboboration process involving direct C–H functionalisation of a (hetero)arene would be more efficient [17]. Regarding alkyne thioborations, while several are reported [5, 18, 19, 20, 21, 22], there are no thioboration reactions that use thiols directly, instead current routes require separate formation and isolation of sensitive thioboranes (e.g., (RS)_3_B or PinB‐SR). Furthermore, there are no general three‐component alkyne thioboration process reported to date. The development of three‐component thio‐/carbo‐borations that address these limitations while being operationally simple and using commercial reagents would be highly notable given the utility of alkenyl boranes [20, 23].

Borylative cyclisation recently has been applied to generate benzoxaborinines (Figure 1c, left) [24] and benzazaborinines [25], which are both important bicyclic boronates (vide infra). Their formation by the borylative‐cyclisation reactions reported to date used BCl_3_/BBr_3_. As the calculated mechanism for this process proceeded by a double‐haloboration/retro‐haloboration sequence, it unavoidably leads to addition of one B‐X unit (X = Cl/Br) to the alkyne, thereby limiting the nucleophile scope to chloride and bromide installation. As functionalisation of the formed vinyl‐X unit in these benzoxaborinines was challenging [24], a borylative cyclisation method that directly installs an exogenous nucleophile instead of a halide from BX_3_ would be highly desirable as an efficient method to rapidly form functionalised derivatives of these BO and BN‐containing heterocycles. While the significance of BO‐heterocycles as bioactives was established with the benzoxaboroles [26], recent work has focused on other bicyclic boronates [27, 28]. In particular, benzoxaborinine derivatives have attracted considerable attention as bioactives in their own right and as key intermediates in the synthesis of ultra‐broad‐spectrum *beta*‐lactamase inhibitors (BLIs, Figure 1c) [29, 30, 31, 32, 33, 34]. The growing demand for these bicyclic boronates has led to an increased need for efficient and modular synthetic routes. Recent work has led to new synthetic methods using transition metal‐catalysed boron insertion into sp^2^C─O and into C(sp^3^)─O bonds (Figure 1d) [35, 36, 37]. This has been followed by the nickel catalysed conversion of coumarins into benzoxaborinines [38, 39], and a Pd‐catalysed coupling approach (Figure 1d) [40, 41]. One metal‐free method involving directed C–H borylation of *ortho*‐substituted styrenes using BBr_3_ has also been reported [42]. However, in all these approaches functional groups need to be pre‐installed prior to formation of the bicyclic boronate or added in a separate subsequent step. The development of a modular methodology to make bicyclic boronates that concomitantly installs an additional functional group would be a significant breakthrough.

Herein, we report the three‐component thio‐ and carbo‐borylative cyclisation of alkynes to give C4‐functionalised‐benzoxaborinines and benzazaborinines in one step. This uses the commercial borane Et_2_O·BF_3_ as the Lewis acid and functions with a wide range of exogenous nucleophiles (thioethers, thiols, and (hetero)arenes) to enable rapid access to libraries of these important bicyclic boronates.

## 2 Results and Discussions

To preclude the alkyne haloboration observed using BCl_3_ and BBr_3_ in previous borylative cyclisation reactions [24], we focused on fluoroboranes, particularly the commercial and easy to handle reagent Et_2_O·BF_3_. Direct alkyne fluoroboration using fluoroboranes does not occur due to the strong B─F bond [4]. This presented an opportunity to access a distinct mechanism (to that operating with BCl_3_) which would allow for the introduction of exogenous nucleophiles into borylative cyclisation processes.

### 2.1 Thioboration

**SCHEME 1:**
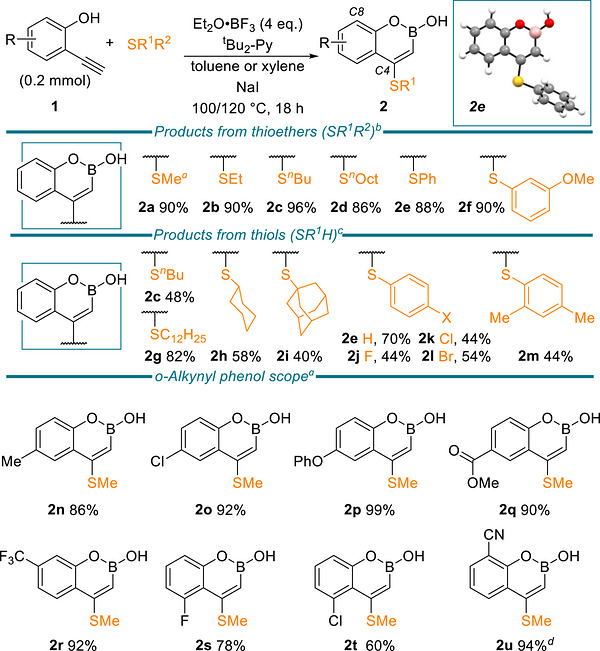
Scope of the thio‐borylative cyclisation reaction. ^a^ *o*‐Ethynyl phenol (0.2 mmol), thioether (3 equiv.), *^t^* Bu_2_‐Py base (2.5 equiv), toluene, 100°C. Yields versus an internal standard. ^b^Conditions a with NaI (2 equiv). ^c^ *o*‐Ethynyl phenol (0.2 mmol), thiol (2.5 equiv), *^t^* Bu_2_‐Py base (2.0 equiv) *o*‐xylene, 120°C. ^d^Thioether (3.4 equiv).

Optimisation studies (see Tables S1–S6) were initiated using 2‐ethynylphenol **1a**, dimethylsulfide as the nucleophile and Et_2_O·BF_3_ targeting a C4‐SMe derivatised benzoxaborinine. From this, it was found that the thioboration reaction proceeded effectively on heating in CPME or in toluene in the presence of 2,6‐di‐*tert*‐butyl‐4‐methylpyridine (termed *^t^* Bu_2_‐Py herein) as a hindered Brønsted base. The benzoxaborinine **2a** was formed in good yield (90%) after aqueous work‐up which converts the primary product containing a B‐F unit into the desired B‐OH product **2a** (Scheme 1). Note, in the absence of the hindered base < 5% of **2a** was formed. Optimising the methodology with longer‐chain thioethers (i.e., SEt_2_) led to the inclusion of NaI (Table S7) to facilitate dealkylation of sulfonium intermediates (vide infra) [43], with the thio‐borylative cyclisation proceeding in only moderate yield in the absence of NaI. Using these conditions (Scheme 1) longer chain symmetric dialkyl thio‐ethers led to formation of products containing SEt (**2b**), S*^n^* Bu (**2c**), and S*^n^* Oct (**2d**) substituents in high yields (86%–96%). Thioanisole derivatives also were amenable in this thioboration and proceeded to give the demethylated products containing SPh (**2e**) and S(3‐(MeO)‐Ph) (**2f**) in 88% and 90% yield, respectively. The structure of **2e** was confirmed by single crystal x‐ray diffraction analysis (inset top right, Scheme 1) [44]. These are the first C4‐thiolated bicyclic boronates reported to our knowledge.

We then re‐optimised the process for the use of thiols as the exogenous nucleophile (Tables S10–S12). The only noteworthy difference in the reaction conditions is that while cyclisation using thiols proceeded at 100°C, higher temperatures (120°C) led to improved yields, necessitating a switch from toluene to *o*‐xylene as solvent. Dodecanethiol was used to form **2g** in 82% yield, while cyclo‐hexylthiol and adamantane thiol gave the thiolated‐benzoxaborinines **2h** and **2i** in moderate yields (58% and 40%, respectively). Thiophenol was used to form **2e** successfully (70% yield), before testing *p*‐halo substituted thiophenols. These all underwent successful thioboration, forming **2j**, **2k** and **2l** in moderate yields (44%–54%). 2,4‐Dimethylphenylthiol also could be incorporated forming **2m** in 44% yield, with this aryl‐sulfur fragment found in approved pharmaceuticals (e.g., vortioxetine).

**SCHEME 2:**
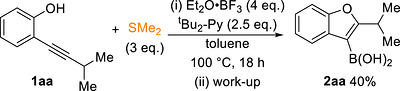
Borylative cyclisation of an internal alkyne to form C3‐borylated benzofuran.

Dimethylsulfide then was selected as the model nucleophile to test the tolerance of this process to substituents on the phenolic ring. Note, ‐SMe units are particularly prevalent in bioactives, including thioridazine, pergolide, and egaten. Notably, methyl (**2n**), chloro (**2o**), phenoxy (**2p**), and ester substituents (**2q**) all were tolerated, forming the respective benzoxaborinine products in high yields (86%→99%). This process therefore tolerates electron‐donating and electron‐withdrawing groups *para* to the OH unit. The reaction also proceeded effectively with a strongly electron‐withdrawing group, CF_3_, *para*‐ to the alkyne, forming product **2r** in 92% yield. Substituents *ortho*‐to the alkyne were tolerated with the fluoro and chloro derivatives, **2s** and **2t**, formed in 78% and 60% yield, respectively. Attempts to perform thio‐borylative cyclisation of a substrate with an ester *ortho* to the phenol failed due to O,O chelation of the hard fluoroborane Lewis acid [24]. However, C8‐cyano substitution (*ortho* to alcohol) was tolerated, with **2u** formed in good yield (94%). Combined, the formation of **2n** – **2u** demonstrates that substituents at all positions on the phenyl ring are tolerated. This transformation was limited however to terminal alkynes. When using an internal alkyne, formation of the C3‐borylated benzofuran proceeds instead (Scheme 2). Presumably, this is due to the differing stability of the vinyl cation derived from interaction of a boron electrophile with the alkyne at the carbon bound to the aryl unit for a terminal versus an internal alkyne as discussed previously [11, 24].

**SCHEME 3:**
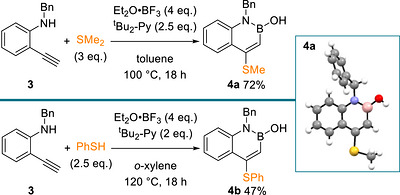
Thio‐borylative cyclisation of *N*‐benzyl‐2‐ethynylaniline using thioethers and thiols. Inset right, the solid‐state structure of **4a**.

The nitrogen analogues, benzazaborinines, are a related class of compounds that also have attracted significant interest, for example, as bioisosteres of naphthalene that have enhanced therapeutic potency [45]. Despite their importance, three component routes to benzazaborinines are extremely rare [46], with none reported to our knowledge for forming the BN‐positional isomer accessed herein. Notably, the standard conditions for both of the three component thio‐borylative cyclisation processes also worked with *N*‐benzyl‐2‐ethynylaniline, **3**. With dimethylsulfide/Et_2_O·BF_3_ this formed C4‐thiolated benzazaborinine **4a** in 72% yield (Scheme 3), while using thiophenol/Et_2_O·BF_3_ led to formation of **4b** in 47% yield. The identity of **4a** was confirmed by single crystal x‐ray diffraction studies [44]. A comparison of the solid‐state structures of C4‐thiolated benzoxaborinine **2e** and the benzazaborinine **4a** revealed that the key structural metrics in the boracycle unit (e.g., S─C, C═C, and C─B distances) are effectively identical.

With an effective thio‐borylative cyclisation process developed that provides access to functionalised benzoxa‐ and benzaza‐borinines, our attention turned to understanding the mechanism when using thioethers and thiols as nucleophiles.

### 2.2 Mechanistic Studies

#### 2.2.1 Thioboration With R_2_S

We first sought to understand the stoichiometry of the optimised conditions which require excess thioether and excess Et_2_O·BF_3_. The Brønsted base is required to deprotonate the initially formed ArylOH‐BF_3_ Lewis adduct and this led to formation of [(*^t^* Bu_2_−Py)H][BF_4_] (observed as a stoichiometric by‐product) and (ArylO)*_x_* BF_3−_ *_x_* (vide infra). This step and a subsequent fluoride abstraction step (vide infra) means that ≥3 equiv of Et_2_O·BF_3_ are required. Formation of a sulfonium salt by‐product also was observed in these borylative cyclisation reactions (with and without the use of NaI as additive). With [R_3_S][BF_4_] isolated (post chromatography) in effectively quantitative yield relative to the yield of the benzoxaborinine, the formation of sulfonium salts as a stoichiometric by‐product accounts for the requirement for ≥2 equiv of R_2_S.

Next the reactions using Me_2_S were monitored by in situ NMR spectroscopy. This revealed that several new species were formed rapidly at room temperature with resonances at *δ*_11B_ 13–16. These are consistent with (ArylO)BF_2_ and (ArylO)_2_BF [47]. On heating, these resonances were replaced with a new resonance at *δ*_11B_ = 26, which grows in concomitantly with a new alkenyl C–*H* resonance in the ^1^H NMR spectra. These are assigned to the thio‐borylative cyclisation product containing a three coordinate boron centre bound to F. This was converted into **2a** on aqueous work‐up. To probe the importance of (ArylO)*_x_* BF_3‐_ *_x_* formation prior to the *anti*‐thioboration step, a reaction in the absence of the phenolic unit was attempted. Under the standard reaction conditions, the combination of phenylacetylene, SMe_2_, Et_2_O·BF_3_, and *^t^* Bu_2_‐Py resulted in no alkyne thioboration. These observations are consistent with a mechanism where 2‐ethynyl‐phenol initially reacts with Et_2_O·BF_3_/*^t^* Bu_2_‐Py to generate (ArylO)*_x_* BF_3‐_ *_x_* (and protonated base). (ArylO)*_x_* BF_3‐_ *_x_* then undergoes thio‐borylative cyclisation by anti‐addition of the SR_2_ nucleophile/boron electrophile. Subsequent fluoride abstraction by a further equivalent of Et_2_O·BF_3_ and dealkylation, either by another equivalent of SR_2_ or by iodide (to initially form R‐I that subsequently alkylates SR_2_ to produce the observed sulfonium cation by‐product), ultimately produces the B‐F benzoxaborinine. Aqueous work‐up then hydrolyses the B‐F unit to give product **2**.

**SCHEME 4:**
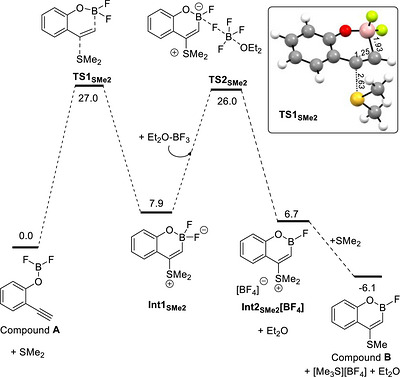
Calculated mechanism for the thio‐borylative cyclisation of **A** with SMe_2_ (Δ*G* (kcal/mol)).

To probe this mechanism further, calculations were performed at the M06‐2X/ma‐def2‐tzvpp (SMD: toluene)//M06‐2X/ma‐def2‐svp (SMD: toluene) level of theory. The dealkylation step was modelled using SMe_2_ as the nucleophile as this reaction was found experimentally to proceed effectively in the absence of NaI. Note, the starting alkyne used is the (ArylO)BF_2_ derivative (Scheme 4 compound **A**), as this compound is observed to form extremely rapidly in solution at room temperature prior to thio‐borylative cyclisation occurring. Compound **A**, combined with Et_2_O·BF_3_ and 2 equiv of SMe_2_ converts to the B‐F benzoxaborinine product, **B**, along with Et_2_O and [Me_3_S][BF_4_] as by‐products, in an overall exergonic process (−6.1 kcal/mol). Note, compound **B** corresponds to the cyclisation product observed in situ in solution that is converted into products **2** on aqueous work up. The thio‐borylative cyclisation proceeds from **A** through a concerted anti‐addition transition state, **TS1_SMe2_**, at 27.0 kcal/mol, to form the zwitterionic intermediate **Int1_SMe2_** at +7.9 kcal/mol. Demethylation directly from **Int1_SMe2_** using SMe_2_ (Figure S153) proceeded with a very high barrier (+39.7 kcal/mol), therefore it is more likely that demethylation occurs after a fluoride abstraction step. Indeed, **Int2_SMe2_[BF_4_]** can be formed by fluoride transfer to Et_2_O·BF_3_ through **TS2_SMe2_** at +26.0 kcal/mol, with this being an S_N_2 at boron process involving an effectively planar equatorial BF_3_ unit in the five coordinate (at boron) transition state. The demethylation of **Int2_SMe2_** by SMe_2_ then proceeds through **TS3_SMe2_** at +25.0 kcal/mol (see Figure S154).

#### 2.2.2 Thioboration With RSH

Moving to thioboration with PhSH as the nucleophile, a related mechanism starting from compound **A** and PhSH (see Figure S155) was calculated and found to have a first transition state (**TS1_PhSH_** +32.2 kcal/mol) and a first intermediate (**Int1_PhSH_** +20.1 kcal/mol) considerably higher in Gibbs free energy relative to that calculated using SMe_2_ (e.g., *δ*Δ*G* = 5.2 kcal/mol for **TS1**). Given the thio‐borylative cyclisation reactions with SMe_2_ and PhSH do both proceed under identical conditions (albeit they are slower at 100°C using PhSH), a mechanism starting from PhSH that has a similar highest energy transition state to that calculated for SMe_2_ is required. This requirement indicates that PhSH is not the key nucleophile in this thioboration process. Considering other possible nucleophiles, the involvement of the thiolate anion, [PhS]^−^, is disfavoured as these reactions are performed in the presence of excess boron Lewis acid which will rapidly sequester [PhS]^−^ to form thioboranes and anionic thioborates (e.g., [(PhS)BF_3_]^−^) in an exergonic step [20].

**SCHEME 5:**
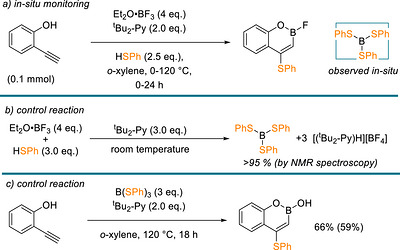
Mechanistic investigation of thioboration using PhSH.

To gain insight into the thio‐borylative cyclisation process using PhSH the reaction was monitored in situ by NMR spectroscopy. At ambient temperature, multiple new ^11^B resonances were observed, these included (ArylO)*_x_* BF_3‐_ *_x_* species at *δ*_11B_ ≈ 15, but no resonances for mixed thio‐borane species for example (ArylO)*_x_* B(SPh)_3‐_ *_x_* [48]. Significantly, a new resonance at *δ*_11B_ = 62 also was observed. This was only observed in reactions using thiols, with no resonance in this region observed when using thioethers. The *δ*_11B_ = 62 resonance is consistent with B(SPh)_3_ (Scheme 5a). This was confirmed by the independent synthesis of B(SPh)_3_ using the reported method [20]. On heating the in situ monitored reactions, the resonances for (ArylO)*_x_* BF_3‐_ *_x_* and B(SPh)_3_ both decreased concomitantly with an increase in thio‐borylative cyclisation product resonances (specifically a *δ*_11B_ = 27 ppm and a new alkenyl C–*H* resonance).

The above indicated that the combination of Et_2_O·BF_3_/PhSH/*^t^* Bu_2_‐Py could form B(SPh)_3_ in situ and that this reagent (or a species derived from it) is crucial in the thio‐borylative cyclisation. The former was confirmed by reacting Et_2_O·BF_3_/PhSH/*^t^* Bu_2_‐Py in a 4:3:3 ratio, which led to selective formation of B(SPh)_3_ and [(*^t^* Bu_2_‐Py)H][BF_4_] in effectively quantitative yield by NMR spectroscopy (Scheme 5b). This is notable as it represents a simple in situ method to make tri‐thioboranes starting from easy‐to‐handle precursors. A Brønsted base was essential for this transformation, with no B(SPh)_3_ observed on combining just Et_2_O·BF_3_ and PhSH (see Figures S9–S14). Based on the above, B(SPh)_3_ was used in the thio‐borylative cyclisation of *o*‐ethynyl phenol, with no other source of organosulfide present. From this reaction (Scheme 5c), the benzoxaborinine product was isolated in 59% yield, indicating that tri‐thioboranes are productive in this chemistry. Given the ratio of PhSH:Et_2_O·BF_3_ used in the optimised conditions (2.5:4), fluoroboranes will still be the major species present in solution, consistent with the observation of (ArylO)*_x_* BF_3‐_ *_x_* by in situ ^11^B NMR spectroscopy. The propensity for main group compounds to favor bonding to all “hard” or all “soft” substituents [49] is consistent with the observation of B(SPh)_3_ and (ArylO)*_x_* BF_3‐_ *_x_* but no “mixed hard‐soft species,” for example (ArylO)*_x_* B(SPh)_3‐_ *_x_*, in the reaction mixture by in situ ^11^B NMR spectroscopy. The formation of thioborate anions (e.g., [(PhS)_4_B]^−^) are also feasible in these mixtures (e.g., from deprotonation of the Lewis adduct (PhSH)B(SPh)_3_). These will be more nucleophilic (than PhSH) and they have been proposed previously as the key nucleophile in alkene hydrothiolation [50]. Therefore, we considered a thioboration mechanism involving anti‐addition to the alkyne of (ArylO)BF_2_ and a thioborate anion. A mechanism proceeding by direct thioboration of the alkyne by B(SPh)_3_ is disfavoured based on previous studies by Uchiyama and coworkers [20].

**SCHEME 6:**
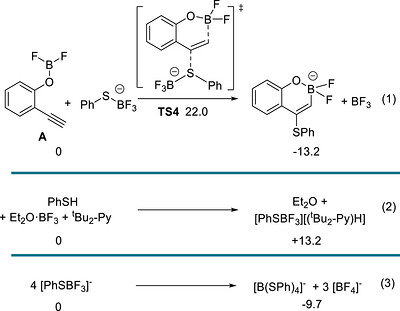
Calculations on thioboration/thioborates (Δ*G* kcal/mol).

Calculations were performed for the anti‐thioboration step initially using [(PhS)BF_3_]^−^ as the organosulfur nucleophile. This thioborate will be the primary product formed from deprotonation of the Lewis adduct (PhSH)BF_3_ by *^t^* Bu_2_‐Py. Significantly, the transition state for the thio‐borylative cyclisation of **A** using [(PhS)BF_3_]^−^ as the nucleophile was 22.0 kcal/mol (**TS4**, Scheme 6, Eq. 1), considerably lower in energy than the analogous process involving **A** and PhSH (**TS1_PhSH_** 32.2 kcal/mol). Given the lower energy of **TS4** relative to **TS1_PhSH_**, the free energy change for the formation [(PhS)BF_3_]^−^ was determined, as this step precedes the thioboration step. This revealed that the formation of [(PhS)BF_3_]^−^ was endergonic by +13.2 kcal/mol (Scheme 6, Eq. 2). When this step is sequenced before the anti‐thioboration step it results in **TS4** now being at +35.2 kcal/mol, too high an energy for this to be a viable pathway.

**SCHEME 7:**
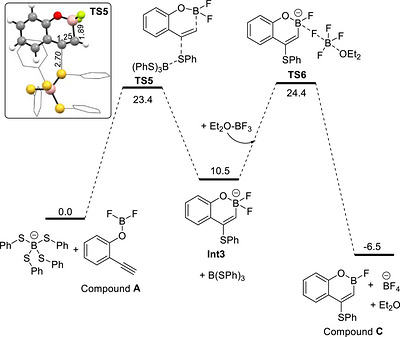
Calculated mechanism for the thio‐borylative cyclisation of **A** with [B(SPh)_4_]^−^ (Δ*G* (kcal/mol)).

Therefore, an analysis of the free energy change for formation of the series of thioborates [(PhS)*_x_* BF_4‐_ *_x_*]^−^ (*x* = 1–4) was performed. Note, the presence of all these thioborates is feasible in solution as substituent scrambling in mixtures or boranes/[borates]^−^ is rapid (consistent with the rapid formation of B(SPh_3_) observed at room temperature in this work). These calculations revealed that formation of [(PhS)_4_B]^−^ (Δ*G* = −9.7/mol, Scheme 6, Eq. 3) was the only thioborate anion whose formation was significantly favoured relative to that of [(PhS)BF_3_]^−^. Therefore, [(PhS)_4_B]^−^ was explored as the nucleophile for thio‐borylative cyclisation of **A** (Scheme 7). Note, compound **A** is used as the starting point as it is again observed to form rapidly at room temperature in these reactions (by in situ NMR spectroscopy).

This revealed an energetically feasible mechanism (Scheme 7) with a highest barrier of 23.4 kcal/mol and an overall conversion to product **C** being exergonic by −6.5 kcal/mol (relative to [(B(SPh)_4_]^−^). The major difference between this mechanism and that calculated for SMe_2_ (Scheme 4) is when using [(PhS)_4_B]^−^ as the nucleophile an anionic product is formed (**Int3**). No sulfonium unit is produced (as present in **Int1_SMe2_**) due to B‐SPh cleavage occurring during the thio‐borylative cyclisation step (involving **TS5**). Given the potential involvement of thioborate anions in thio‐borylative cyclisation starting from PhSH, we assessed the feasibility of forming [(MeS)BF_3_]^−^ from Me_2_S and Et_2_O·BF_3_. However, the formation of [Me_3_S][(MeS)BF_3_] is considerably endergonic (+32.5 kcal/mol). This disfavours the involvement of thioborate anions in the borylative cyclisation using thioethers.

### 2.3 Carboboration

**SCHEME 8:**
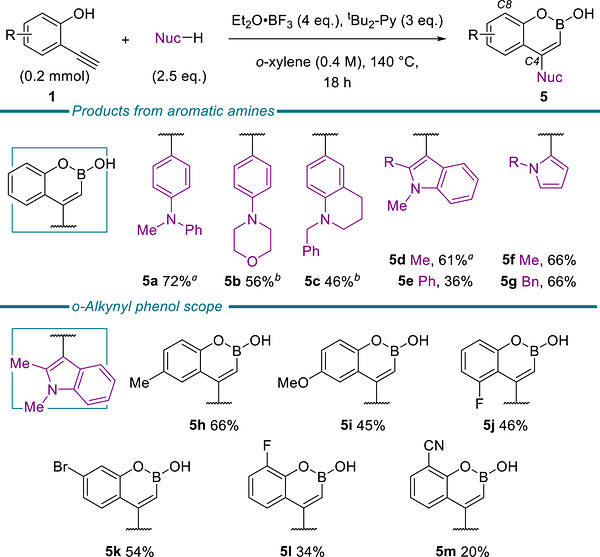
Scope of the carbo‐borylative cyclisation reaction. ^a^150°C. ^b^100°C. Yields versus an internal standard.

With an understanding of the thio‐borylative cyclisation mechanisms in hand, the use of other nucleophiles in three‐component borylative cyclisation was investigated. Given that other three‐component alkyne carboborations invariably require a pre‐functionalised organic moiety (e.g., an organo‐halide) [17], we targeted incorporating a C‐H functionalisation step into the borylative cyclisation process to improve step‐efficiency. The anti‐carboboration of 2‐ethynyl phenol using Et_2_O∙BF_3_ and a range of (hetero)arenes proceeded efficiently at 140°C (Scheme 8). The use of diphenylmethylamine produced **5a** in good yield (72%), while a morpholine substituted arene was also successful, forming **5b** in 56% yield. Tetrahydroquinolines are a common motif in pharmaceuticals and this heterocycle can be used to generate the C4 functionalised benzoxaborinine **5c**. Moving to heteroarenes, indole is a privileged unit in natural products and pharmaceuticals [51]. 1,2‐Dimethylindole was utilised as the nucleophile and was converted into **5d** in 61% yield. 1‐Methyl‐2‐phenylindole also was viable; however, this proceeded in a lower yield to form **5e** (36% yield), presumably due to the increased steric bulk in the C2‐position. *N*‐Methyl and *N*‐benzyl pyrrole also were viable and were functionalised selectively at the C2 position of the pyrrole to form **5f** and **5g** both in 66% yield. This carbo‐boration process requires nucleophilic (hetero)arenes, with 2‐methyl‐thiophene and 1,2‐dimethoxybenzene leading to minimal (<10%) formation of the target products under identical conditions. Nevertheless, the formation of a range of novel bis‐heterocyclic compounds in one pot from three separate components is highly notable, with routes to combine two unsymmetrical heterocycles in one molecule of interest as these can provide desirable biological properties due to synergistic effects [52].

To assess the *o*‐alkynyl phenol scope, 1,2‐dimethylindole was selected as the model nucleophile due to its prevalence in biologically active molecules [52]. Notably, electron‐donating and electron‐withdrawing substituents were tolerated, specifically: methyl (**5h**), methoxy (**5i**), fluoro (**5j** and **5l**), and bromo (**5j**), with yields of the benzoxaborinines comparable to that of the parent carbo‐boration reaction (34%–66%). Furthermore, the formation of **5h**–**5l** confirms that substituents at all positions on the phenyl ring are tolerated. Given the importance of a carboxylate unit at the C8 position in bicyclic boronate BLIs, the C8‐cyano derivative again was targeted using the standard conditions. This led to formation of compound **5m** in 20% yield.

### 2.4 Hydroamination

**SCHEME 9:**
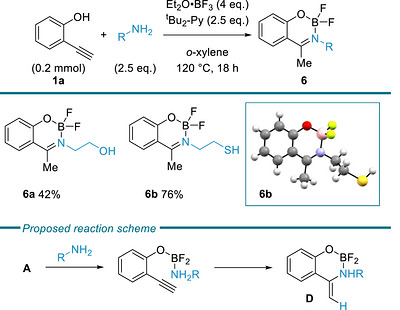
Boron‐directed hydroamination of alkynes with amines.

The thioboration and carboboration reactions discussed above both use “soft” nucleophiles. To determine if analogous amino‐/oxy‐borylative cyclisation reactions would proceed despite using harder N/O‐based nucleophiles (which will have a higher propensity to bind to boron Lewis acids), Et_2_O and Me_2_NBn were used as nucleophiles in the optimised conditions. However, this led to complex mixtures containing minimal (<10%) benzoxaborinine product (see Table S6). Ethanol‐amine was used next to determine if the presence of E‐H units (E = RO or RNH) enabled the borylative cyclisation reaction. However, this led to an alternative outcome, specifically the formation of boranil product **6a** (Scheme 9). This presumably proceeds by coordination of nitrogen to boron in compound **A** which enables the hydroamination of the terminal alkynyl group via initial alkyne protonation (Scheme 9, bottom) [53]. Tautomerisation of the enamine‐intermediate **D** would then lead to boranil **6a**. Cysteamine (HSCH_2_CH_2_NH_2_) was utilised next to determine if thioborylative cyclisation or boranil formation would be preferred. The use of cysteamine led to formation of boranil **6b** (structure confirmed through single crystal x‐ray diffraction studies, see inset of Scheme 9) [44], with no benzoxaborinine formation observed. Note, boranils are of significant interest in their own right as fluorophores [54], with **6a** and **6b** representing novel functionalised boranils. The absence of benzoxaborinine formation indicates that hard nucleophiles are either: insufficiently nucleophilic for this borylative cyclisation process or preferentially coordinate to the boron Lewis acids present in the reaction mixture.

**SCHEME 10:**
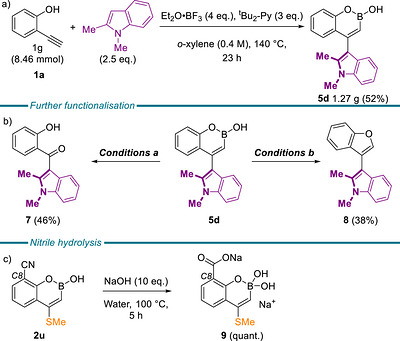
a) and b) Scale‐up and derivatisation of **5d**. c) formation of the C8‐carboxylate benzoxaborinine. Conditions a) 30% H_2_O_2_ (1.0 mL), 3.0 N NaOH (1.0 mL), THF/EtOH (2.0 mL/0.5 mL), rt, 10 min. Conditions b) Cu(OAc)_2_ (0.2 equiv), Ag_2_CO_3_ (3.0 equiv), 1,10‐phenanthroline (0.22 equiv), EtOH (2.0 mL), H_2_O (0.1 mL), air, 80°C, 22 h. Isolated yield in parentheses.

The applicability of this methodology was demonstrated further through a scaled‐up synthesis of **5d**, forming 1.27 g of product in 52% isolated yield (Scheme 10a). This product was transformed into two other novel C3‐functionalised indoles. Oxidation led to ketone **7** (Scheme 10b, left), presumably through a benzofuranone intermediate [55]. The synthesis of diaryl **8** was achieved using boron‐deletion skeletal editing conditions [56], this provides a novel route to a new di‐heterocyclic compound (Scheme 10b, right). Finally, for a C8‐cyano substituted benzoxaborinine, it was important to confirm that the cyano group could be converted into a carboxylate without any off‐target reactivity as a carboxylate unit at the C8 position is essential for ultra‐broad spectrum BLI activity (Figure 1c). Therefore, **2u** was heated in basic aqueous media at 100°C which led to formation of the disodium salt **9**, in effectively quantitative yield (Scheme 10c). Formation of the disodium salt directly is desirable as the bicyclic boronate BLIs containing C8‐carboxylates are generally used as the disodium salts [29].

## 3 Conclusion

This work showcases multiple novel three‐component alkyne elemento‐boration reactions. These are operationally simple as they use the easy to handle and inexpensive borane Et_2_O·BF_3_ as the Lewis acid and commercial nucleophiles. The nucleophile scope includes many examples that are unprecedented in current three component elemento‐boration methodologies for example the direct use of thiols in alkyne thioboration. Furthermore, the ability to perform alkyne carboboration reactions involving direct C‐H functionalisation with a simple borane is also a notable feature of this work that enables formation of structurally complex bis heterocycles in a single step. Mechanistic studies revealed a disparity between thio‐borylative cyclisation using thioethers and thiols as the nucleophile. With the former, our studies indicated that the thioether was the key nucleophile, but for thiols in situ formation of a tri‐thioborane (B(SR)_3_) proceeded first, with a tetra‐thioborate anionic nucleophile ([(RS)_4_B]^−^) then calculated to be the key nucleophile for this thio‐borylative cyclisation. This work also introduced a new method to form B(SR)_3_ in situ from easy‐to‐handle reagents: Et_2_O∙BF_3_, RSH, and a hindered base. While this borylative cyclisation approach cannot be extended to harder nucleophiles (e.g., R_2_O and R_3_N), the use of primary amines enabled a different reaction, boron directed alkyne hydroamination, which represents a new route to form boranils, which are an increasingly important class of fluorophores.

In addition to the above, this work provided facile (one‐pot) access to a range of structurally complex BO and BN‐containing heterocycles that species are of considerable current interest [26]. The ability to form bicyclic boronates containing Lewis basic sulfur units is particularly notable as it demonstrates that this methodology has a functional group compatibility profile complimentary to established nickel and palladium catalysed processes. The utility of our approach was confirmed by it being applied to a wide range of functionalised substrates and reagents, being easy to scale and applicable to make C8‐carboxylate containing bicyclic boronate derivatives (which is essential for broad BLI activity). Overall, this process is a useful addition to the toolbox for making functionalised bicyclic boronates, while demonstrating that there is still significant scope in using the simple borane Et_2_O·BF_3_ to discover novel borylation processes.

## Author Contributions

**Laura Winfrey**: methodology, investigation, writing – original draft, writing – review and editing, data curation, formal analysis, conceptualisation, validation. **Gary S. Nichol**: data curation, formal analysis. **Stephen P. Thomas**: writing – review and editing, supervision, funding acquisition. **Dominic R. Willcox**: conceptualisation, writing – review and editing, methodology, investigation. **Michael. J. Ingleson**: conceptualisation, funding acquisition, writing – original draft, writing – review and editing, supervision, project administration, formal analysis, validation.

## Conflicts of Interest

The authors declare no conflicts of interest.

## Supporting information




**Supporting File 1**: anie72649‐sup‐0001‐SuppMat.pdf.

**Supporting File 2**: anie72649‐sup‐0002‐cif.zip.

**Supporting File 3**: anie72649‐sup‐0003‐xyz.zip.

## Data Availability

The data that supports the findings of this study are available in the Supporting Information of this article.
