# Supplementary material for: Three Component Thio‐ and Carboboration of Alkynes: A Modular Route to Functionalised Bicyclic Boronates
Source: Angew Chem Int Ed Engl. 2026 May 27;65(30):e9548215. doi: 10.1002/anie.9548215 (PMC13383196; doi:10.1002/anie.9548215)
Supplement: Supplementary file 1 — Supporting File 1: anie72649‐sup‐0001‐SuppMat.pdf. [file ANIE-65-e9548215-s001.pdf]

Supporting Information

Three Component Thio- and Carbo-boration of Alkynes: A Modular Route to Functionalised Bicyclic Boronates

L. Winfrey, Gary S Nichol, S. P Thomas, D. R Willcox and M. J Ingleson

Contents

S1. General Information 3
S2. Experimental and Characterisation Data 4
S2.1. Optimisation Reactions 4
S2.1.1. Optimisation of Thioboration with 2-Ethynylphenol and Dimethyl Sulfide 4
S2.1.2 Optimisation of Thioboration with 2-Ethynylphenol and Dibutyl Sulfide 6
S2.1.3 Optimisation of Thioboration with 2-Ethynylphenol and Thiophenol 7
S2.1.4 Optimisation of Carboboration with 2-Ethynylphenol and *N*-Methyl-*N*-phenylaniline 8
S2.2 Scope 9
S2.2.1 General Procedure 1: Thioboration of *o*-Alkynyl Phenols with S-Nucleophiles 9
S2.2.2 C3-Borylated Benzofuran From an Internal Alkyne 18
S2.2.3 Thioboration of *N*-Benzyl-2-ethynylaniline 19
S2.2.4 General Procedure 2: Carboboration of *o*-Alkynyl Phenols with C-Nucleophiles 20
S2.2.5 General Procedure 3: Hydroamination of *o*-Alkynyl Phenols with Amines 26
S2.3 Derivatisation of Organoborane Products 27
S3. Mechanistic Investigation 29
S4. Synthesis of Starting Materials 40
S5. NMR Spectra 49
S6. Computational Details 129
S6.1 Computational Data 130
S7. Crystallographic Details 135
S7.1 Crystallographic Data and Experimental 135

S1. General Information

Reaction setup: Unless otherwise stated, handling of air- and moisture-sensitive reagents was carried out under an inert atmosphere using either standard Schlenk techniques or an MBraun glovebox (< 0.1 ppm H_2O/O_2).

Solvents: unless otherwise stated dry solvents were obtained from an Inert PureSolv MD5 solvent purification system and stored over 3 Å molecular sieves. $CDCl_3$ was distilled over CaH_2 and stored over 3 Å molecular sieves protected from air and direct sunlight. Cyclopentyl methyl ether and C_6D_6 were degassed and stored over 3 Å molecular sieves protected from air and direct sunlight.

NMR spectroscopy: (^{1}H, ^{11}B, $^{13}C\{^{1}H\}$, and ^{19}F NMR) were recorded on Bruker Avance III 400, Bruker Avance III 500 MHz, Bruker PRO 500 MHz, or Bruker Avance I 600 MHz spectrometers. Chemical shifts are reported as dimensionless δ values and are frequency referenced relative to residual protio-solvent signals in the NMR solvents for ^{1}H and $^{13}C\{1H\}$, while ^{11}B and ^{19}F shifts are referenced indirectly, using the chemical shift of the deuterated lock solvent. It should be noted that the very broad signals observed at ca. 0 ppm in the ^{11}B NMR spectra are due to the use of borosilicate glass NMR tubes and boron containing parts in the NMR cavity. $^{13}C\{^{1}H\}$ NMR spectra are listed as ^{13}C. Multiplicities are shown as s (singlet), d (doublet), t (triplet), q (quartet), quin. (quintet), sext. (sextet), sept. (septet). Coupling constants J are given in Hertz (Hz) as positive values regardless of their real individual signs. Benzoxaborinines are known to form the anhydride in solution.[1] In some cases D_2O (40 µL) was added to the prepared NMR aliquots to hydrolyse the B-O-B unit and aid characterisation. In these cases, the proton signal of the boranol (B-OH) unit is absent in the ^{1}H NMR spectra. Unless otherwise stated NMR spectra were recorded at 20 °C. Reaction yields were measured by ^{1}H NMR spectroscopy versus an internal standard in a Bruker Avance III 500 MHz spectrometer or a Bruker Avance I 600 MHz spectrometer. NMR spectra were analysed by MestReNova processing software.

Mass spectrometry: was performed by the Scottish Instrumentation and Resource Centre for Advanced Mass Spectrometry (SIRCAMS) of the University of Edinburgh using either electron impact (EI) or electrospray ionisation (ESI) techniques. Accurate masses are calculated using the most abundant isotopes of each element.

Column chromatography: Column chromatography was carried out on a Teledyne ISCO CombiFlash NextGen 300+ using RediSep Rf normal phase silica flash columns (12, 25, 40, or 80 g; 20-40 microns). Substrates were purified using stated solvent mixtures on a gradient of 100:0 to 0:100 with flow rates of 10-110 mL min-1 depending on the size of column and ΔRf. Alternatively, manual columns were also carried out (40-63 µm silica).

Chemicals: All reagents were purchased from Sigma Aldrich, Alfa Aesar, Acros organics, Tokyo Chemical Industries UK, Fluorochem or Strem UK and were used without further purification unless otherwise stated. KF was obtained from Honeywell (>99%, spray dried), finely ground and stored in a glovebox without any further purification.

[1] L. Benhamou, D. W. Walker, D. K. Bučar, A. E. Aliev and T. D. Sheppard, *Org. Biomol. Chem.,* 2016, **14**, 8039–8043.

S2. Experimental and Characterisation Data

S2.1. Optimisation Reactions

S2.1.1. Optimisation of Thioboration with 2-Ethynylphenol and Dimethyl Sulfide

Table S1. Effect of base

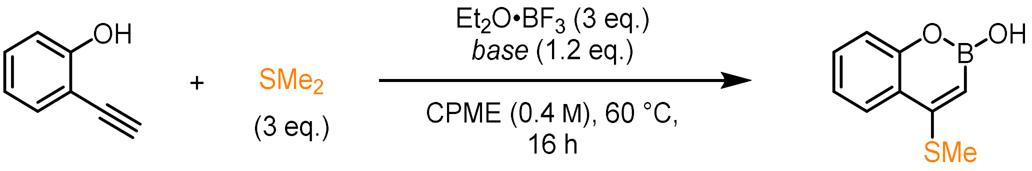

1a 2a

Entry[a]	Base	Yield **2a** (%)[b]
1	2,6-*t*Bu-4-Me-Py	40
2	PMP	9
3	Lutidine	25
4	Et_3N	13

[a]Reactions were performed using 0.2 mmol of 2-ethynylphenol in CPME (0.4 M). [b]Yields were determined by ^1H NMR analysis of the crude reaction mixture using nitromethane as internal standard.

Table S2. Effect of work-up

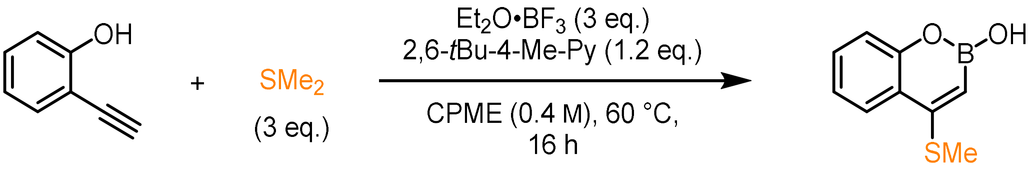

1a 2a

Entry[a]	Work-up	Yield **2a** (%)[b]
1	$CaCl_2$	40
2	HCl (1 M)	18
3	NaOH (1 M)	1
4	K_2CO_3	24
5	$NaHCO_3$	27

[a]Reactions were performed using 0.2 mmol of 2-ethynylphenol in CPME (0.4 M). [b]Yields were determined by ^1H NMR analysis of the crude reaction mixture using nitromethane as internal standard.

Table S3. Effect of sulfide equivalents

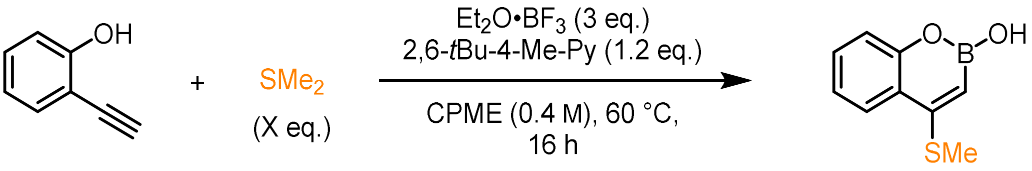

1a 2a

Entry[a]	SMe_2 (eq.)	Yield **2a** (%)[b]
1	2	26
2	3	40
3	4	40
4	6	46

[a]Reactions were performed using 0.2 mmol of 2-ethynylphenol in CPME (0.4 M). [b]Yields were determined by ^1H NMR analysis of the crude reaction mixture using nitromethane as internal standard.

Table S4. Effect of sulfide and base equivalents, solvent and temperature

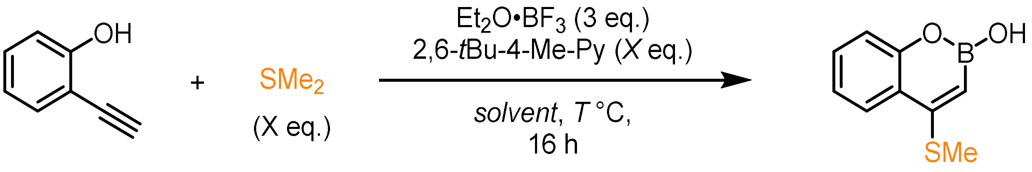

Entry[a]	SMe_2 (eq.)	Solvent	T (°C)	2,6-*t*Bu-4-Me-Py (eq.)	Yield **2a** (%)[b]
1	3	CH_2Cl_2	60	1.2	45
2	3	CPME	60	1.2	40
3	3	toluene	60	1.2	42
4	3	THF	60	1.2	0
5	3	hexane	60	1.2	49
6	3	CPME	20	1.2	1
7	3	CPME	40	1.2	11
8	6	CPME	60	1.5	46
9	6	CPME	60	2.0	55
10	6	CPME	80	2.0	80
11	6	CPME	100	2.0	62

[a]Reactions were performed using 0.2 mmol of 2-ethynylphenol in solvent (0.4 M). [b]Yields were determined by ^1H NMR analysis of the crude reaction mixture using nitromethane as internal standard.

Table S5. Effect of $Et_2O{\cdot}BF_3$ and base equivalents and time

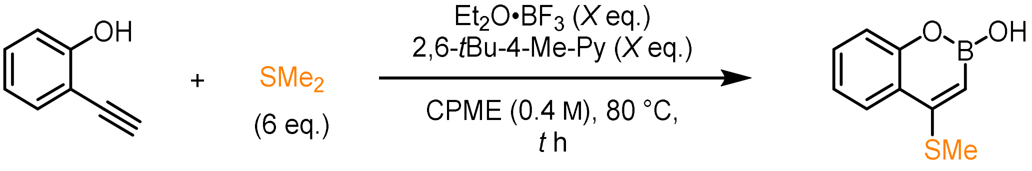

Entry[a]	$Et_2O{\cdot}BF_3$ (eq.)	2,6-*t*Bu-4-Me-Py (eq.)	Time	Yield **2a** (%)[b]
1	2	2.0	16	40
2	3	2.0	16	74
3	3	2.0	24	78
4	3	2.0	48	63
5	4	2.0	16	86
6	4	2.5	24	90
7	4	3.0	24	90

[a]Reactions were performed using 0.2 mmol of 2-ethynylphenol in CPME (0.4 M). [b]Yields were determined by ^1H NMR analysis of the crude reaction mixture using nitromethane as internal standard.

Table S6. Optimised conditions and controls

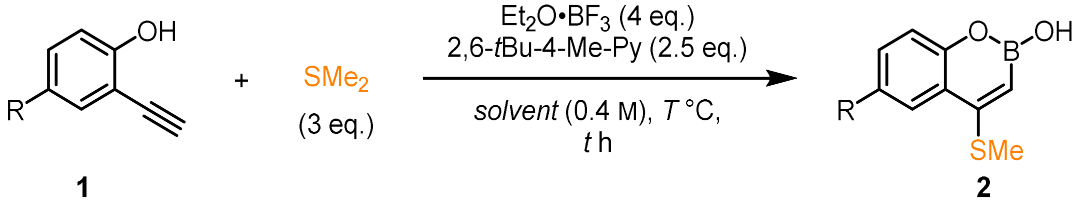

Entry[a]	R =	Solvent	T (°C)	Time	Control	Yield **2a** (%)[b]
1	H	CPME	80	24	-	86
2	H	toluene	100	18	-	90
3	H	toluene	100	18	**No base**	2
4	H	toluene	100	18	**No SMe_2**	0[c]
5	Cl	none	100	18	**Neat**	56

[a]Reactions were performed using 0.2 mmol of 2-ethynylphenol in solvent (0.4 M). [b]Yields were determined by ^{1}H NMR analysis of the crude reaction mixture using nitromethane as internal standard. [c]No thioboration product **2a**. Oxyboration product (ca. 10%) isolated as an inseparable mixture with C3-borylated benzofuran (tentative assignments).

S2.1.2 Optimisation of Thioboration with 2-Ethynylphenol and Dibutyl Sulfide

Note: The conditions for **2a** (CPME, 80 °C, 24 h) did not give universally good yields for alternative sulfides, so optimisation was continued using S^nBu_2.

Table S7. Additive Screen

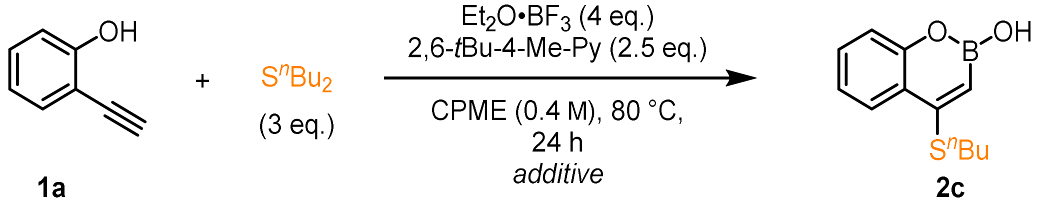

Entry	Additive (1 eq.)	Yield **2c** (%)
1	None	32
2	NaI	56
3	KI	46
4	Et_3N	40
5	PMP	40
6	$^iPr_2NH$	48
8	$(^nBu)_4NI$	26

[a]Reactions were performed using 0.2 mmol of 2-ethynylphenol in CPME (0.4 M). [b]Yields were determined by ^{1}H NMR analysis of the crude reaction mixture using nitromethane as internal standard.

Table S8. Effect of NaI equivalents

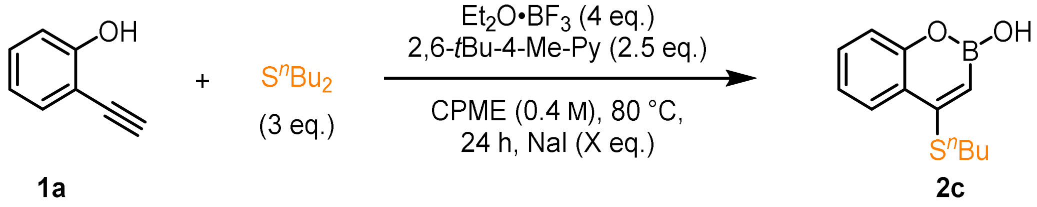

Entry	NaI (X eq.)	Yield **2c** (%)
1	none	46
2	0.5	54
3	1	54
4	2	60
5	5	52

[a]Reactions were performed using 0.2 mmol of 2-ethynylphenol in solvent (0.4 M). [b]Yields were determined by ^{1}H NMR analysis of the crude reaction mixture using nitromethane as internal standard.

Table S9. Change of solvent, time and temperature

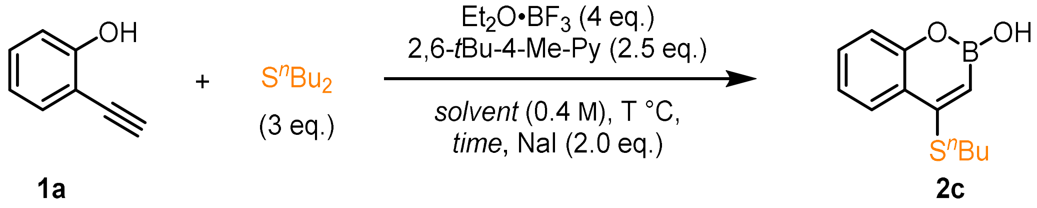

Entry	Solvent	T (°C)	Time (h)	Yield **2c** (%)
1	CPME	80	24	60
2	toluene	100	18	96

[a]Reactions were performed using 0.2 mmol of 2-ethynylphenol in solvent (0.4 M). [b]Yields were determined by ^{1}H NMR analysis of the crude reaction mixture using nitromethane as internal standard.

S2.1.3 Optimisation of Thioboration with 2-Ethynylphenol and Thiophenol

CAUTION: reactions heating toluene past its boiling point are performed in sealed tubes with significant headspace and should be performed using a blast shield.

Table S10. Effect of temperature

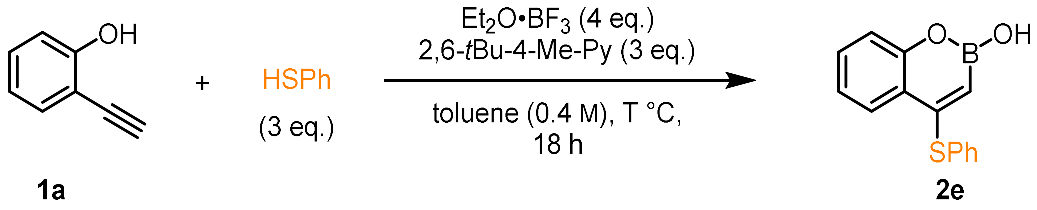

Entry[a]	T (°C)	Yield **2e** (%)[b]
1	80	24
2	100	54
3	120	66

[a]Reactions were performed using 0.2 mmol of 2-ethynylphenol in toluene (0.4 M). [b]Yields were determined by ^{1}H NMR analysis of the crude reaction mixture using nitromethane as internal standard.

Table S11. Effect of 2,6-*t*Bu-4-methylpyridine equivalents

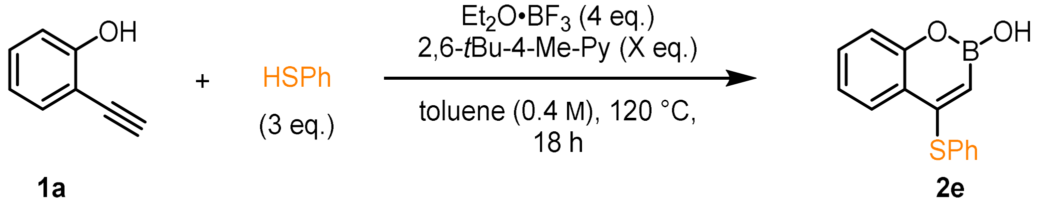

Entry[a]	2,6-*t*Bu-4-methyl-Py (eq.)	Yield **2e** (%)[b]
1	1.5	32
2	2	68
3	2.5	68

[a]Reactions were performed using 0.2 mmol of 2-ethynylphenol in toluene (0.4 M). [b]Yields were determined by ^{1}H NMR analysis of the crude reaction mixture using nitromethane as internal standard.

Table S12. Effect of thiophenol equivalents, solvent and temperature

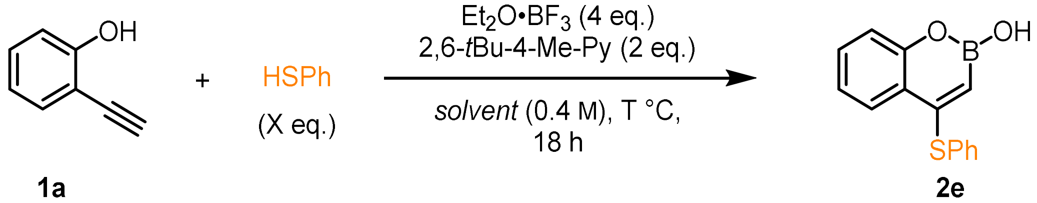

Entry[a]	solvent	T (°C)	HSPh (eq.)	Yield **2e** (%)[b]
1	toluene	120	2	66
2	*o*-xylene	120	2	62
3	*o*-xylene	120	2.5	70
4	*o*-xylene	140	2.5	50

[a]Reactions were performed using 0.2 mmol of 2-ethynylphenol in toluene (0.4 M). [b]Yields were determined by ^{1}H NMR analysis of the crude reaction mixture using nitromethane as internal standard.

S2.1.4 Optimisation of Carboboration with 2-Ethynylphenol and *N*-Methyl-*N*-phenylaniline

Table S13. Effect of 2,6-*t*Bu-4-methylpyridine equivalents and temperature

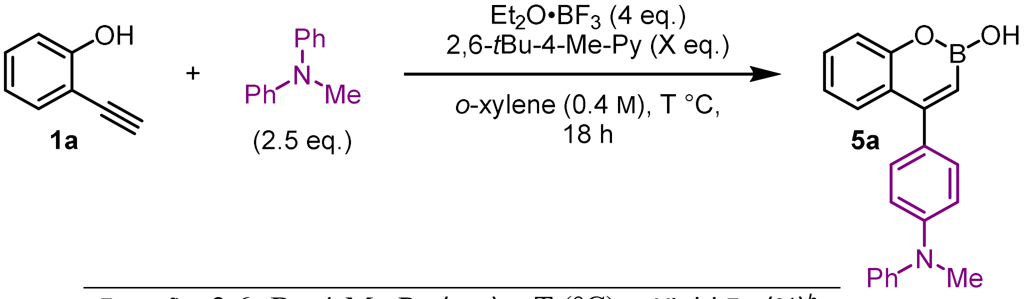

Entry[a]	2,6-*t*Bu-4-Me-Py (eq.)	T (°C)	Yield **5a** (%)[b]
1	2	120	30
2	3	120	38
3	4	120	24
4	3	140	60
5	3	150	72

[a]Reactions were performed using 0.2 mmol of 2-ethynylphenol in o-xylene (0.4 M). [b]Yields were determined by ^{1}H NMR analysis of the crude reaction mixture using nitromethane as internal standard.

Note: There was no observable improvement for almost all other carboboration products when the reaction was heated at 150 °C, so 140 °C was the chosen condition going forward.

S2.2 Scope

S2.2.1 General Procedure 1: Thioboration of *o*-Alkynyl Phenols with S-Nucleophiles

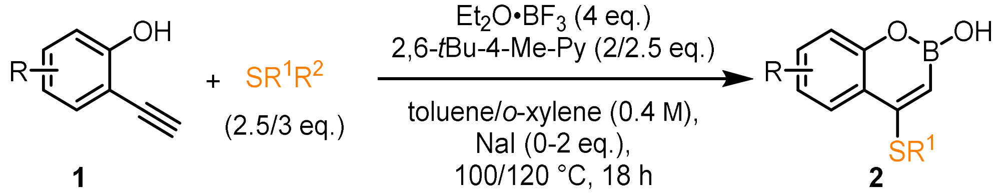

To an oven-dried J. Youngs ampoule equipped with a magnetic stir bar, $Et_2O{\cdot}BF_3$ (100 µL, 0.800 mmol) was added to a stirred solution of 2-ethynylphenol **1** (24.0 mg, 0.200 mmol) and 2,6-di-*tert*-butyl-4-methylpyridine (specified in GP 1a and 1b), then:

GP 1a: Sulfide (0.600 mmol) with 2,6-di-*tert*-butyl-4-methylpyridine (103.0 mg, 0.500 mmol) and NaI (60.0 mg, 0.400 mmol) in toluene (0.500 mL) at room temperature.

GP 1b: Thiol (0.500 mmol) with 2,6-di-*tert*-butyl-4-methylpyridine (82.0 mg, 0.500 mmol) in *o*-xylene (0.500 mL) at room temperature.

The mixture was then heated in a sealed tube for 18 hours at the specified temperature. After allowing to cool to room temperature, the reaction was quenched with saturated aqueous $CaCl_2$ solution (1.00 mL) and stirred vigorously for 15 minutes. The mixture was then diluted with water (10 mL) and extracted with EtOAc (3 × 10 mL). The combined organic layers were dried over $MgSO_4$, filtered, and concentrated *in vacuo*. The yield was determined by ^{1}H NMR spectroscopy using nitromethane as an internal standard. The crude residue was purified via flash column chromatography on silica gel to obtain the pure oxaborinines **2** (CombiFlash Isco NextGen300+, 4 g SiO_2, CH_2Cl_2:MeOH, 100:0 to 90:10).

Note: Working on a 0.2 mmol scale is subject to a more magnified impact on % yield when there is very little mass-loss (e.g. from filtering/column/transferring between vessels), which is why there is a disparity between isolated and spectroscopic yield.

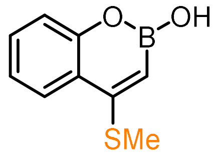

4-(Methylthio)-2-hydroxy-1,2-benzoxaborinine 2a

Following General Procedure 1a, using 2-ethynylphenol **1a** (24.0 mg, 0.200 mmol), 2,6-di-*tert*-butyl-4-methylpyridine (103.0 mg, 0.500 mmol) and dimethyl sulfide (44.0 µL, 0.600 mmol) in toluene at 100 °C gave title compound **2a** (90% spectroscopic yield). Purification by flash chromatography (CombiFlash Isco NextGen300+, 4 g SiO_2, CH_2Cl_2:MeOH, 100:0 to 90:10) gave the title compound **2a** as a white solid (24.3 mg, 0.13 mmol, 63%).

^{1}H NMR (500 MHz, acetone-d$_6$) δ 7.76 (dd, *J* = 8.0, 1.6 Hz, 1H), 7.70 (s, 1H), 7.43 (ddd, *J* = 8.2, 7.2, 1.6 Hz, 1H), 7.23 (dd, *J* = 8.3, 1.3 Hz, 1H), 7.17 (ddd, *J* = 8.0, 7.2, 1.3 Hz, 1H), 5.95 (s, 1H), 2.48 (s, 3H). **^{13}C NMR (126 MHz, acetone-d$_6$)** δ 157.8, 152.8, 130.8, 125.2, 123.9, 122.7, 119.7, 111.3 (br), 13.7. **^{11}B NMR (160 MHz, acetone-d$_6$)** δ 27.0. **HRMS (ESI$^+$):** calculated for ($C_9H_9{}^{11}BO_2SNa$ $[M+Na]^+$) = 215.0309; found 215.0316. **HRMS (ESI$^-$):** calculated for ($C_9H_8{}^{11}BO_2S$ $[M-H]^-$) = 191.0343; found 191.0348.

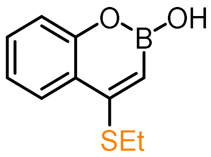

4-(Ethylthio)-2-hydroxy-1,2-benzoxaborinine 2b

Following General Procedure 1a, using 2-ethynylphenol **1a** (24.0 mg, 0.200 mmol), 2,6-di-*tert*-butyl-4-methylpyridine (103.0 mg, 0.500 mmol), diethyl sulfide (65.0 µL, 0.600 mmol) and NaI (60.0 mg, 0.400 mmol) in toluene at 100 °C gave title compound **2b** (90% spectroscopic yield). Purification by flash chromatography (CombiFlash Isco NextGen300+, 4 g SiO_2, CH_2Cl_2:MeOH, 100:0 to 90:10) gave the title compound **2b** as a clear oil (29.5 mg, 0.14 mmol, 72%).

^{1}H NMR (500 MHz, acetone-d$_6$) δ 7.79 (dd, *J* = 7.9, 1.6 Hz, 1H), 7.70 (s, 1H), 7.44 (ddd, *J* = 8.5, 7.4, 1.6 Hz, 1H), 7.22, (dd, *J* = 8.5, 1.2 Hz, 1H), 7.18 (ddd, *J* = 7.9, 7.4, 1.2 Hz, 1H), 6.00 (s, 1H), 3.05 (q, *J* = 7.4 Hz, 2H), 1.41 (t, *J* = 7.4 Hz, 3H)**. ^{13}C NMR (126 MHz, acetone-d$_6$)** δ 156.8, 153.1, 130.9, 125.4, 124.1, 122.8, 119.8, 112.0 (br) 25.2, 13.5. **^{11}B NMR (160 MHz, acetone-d$_6$)** δ 27.0. **HRMS (ESI$^+$):** calculated for ($C_{10}H_{11}{}^{11}BO_2SNa$ $[M+Na]^+$) = 229.04650; found 229.04650. **HRMS (ESI$^-$):** calculated for ($C_{10}H_9{}^{11}BO_2S$ $[M-H]^-$) = 205.0500; found 205.0497.

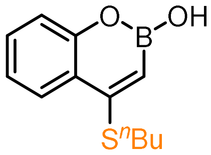

4-(*n*-Butylthio)-2-hydroxy-1,2-benzoxaborinine 2c

Following General Procedure 1a, using 2-ethynylphenol **1a** (24.0 mg, 0.200 mmol), 2,6-di-*tert*-butyl-4-methylpyridine (103.0 mg, 0.500 mmol), dibutyl sulfide (0.600 mmol, 105.0 µL) and NaI (0.400 mmol, 60.0 mg) in toluene at 100 °C gave title compound **2c** (96% spectroscopic yield). Purification by flash chromatography (CombiFlash Isco NextGen300+, 4 g SiO_2, CH_2Cl_2:MeOH, 100:0 to 90:10) gave the title compound **2c** as a red oil (28.6 mg, 0.12 mmol, 61%).

Following General Procedure 1b, using 2-ethynylphenol **1a** (24.0 mg 0.200 mmol), 2,6-di-*tert*-butyl-4-methylpyridine (82.0 mg, 0.400 mmol) and 1-butanethiol (54.0 µL, 0.500 mmol) in *o*-xylene at 120 °C gave title compound **2c** (48% spectroscopic yield).

^{1}H NMR (500 MHz, acetone-d$_6$+40 uL D_2O) δ 7.78 (dd, *J* = 7.9, 1.5 Hz, 1H), 7.42 (ddd, *J* = 8.5, 7.3, 1.5 Hz, 1H), 7.22, (dd, *J* = 8.5, 1.2 Hz, 1H), 7.16 (ddd, *J* = 7.9, 7.3, 1.2 Hz, 1H), 6.01 (s, 1H), 3.00 (t, *J* = 7.4 Hz, 2H), 1.73 (quin., *J* = 7.4 Hz, 2H), 1.51 (quin., *J* = 7.4 Hz, 2H), 0.95 (t, *J* = 7.4 Hz, 3H). **^{13}C NMR (126 MHz, acetone-d$_6$+40 uL D_2O)** δ 155.1, 151.4, 129.3, 123.8, 122.5, 121.1, 118.3, 110.9 (br) 29.3, 29.2, 21.3, 12.4. **^{11}B NMR (160 MHz, acetone-d$_6$)** δ 26.9. **HRMS (ESI$^+$):** calculated for ($C_{12}H_{16}{}^{11}BO_2S$ $[M+H]^+$) = 235.09586; found 235.0953. **HRMS (ESI$^+$):** calculated for ($C_{12}H_{15}{}^{11}BO_2SNa$ $[M+Na]^+$) = 257.0778; found 257.0784.

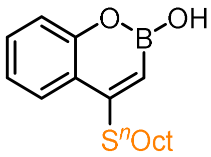

4-(*n*-Octylthio)-2-hydroxy-1,2-benzoxaborinine 2d

Following General Procedure 1a, using 2-ethynylphenol **1a** (24.0 mg, 0.200 mmol), 2,6-di-*tert*-butyl-4-methylpyridine (103.0 mg, 0.500 mmol) and dioctyl sulfide (0.18 mL, 0.600

mmol) in toluene at 100 °C gave title compound **2d** (86% spectroscopic yield). Purification by flash chromatography (CombiFlash Isco NextGen300+, 4 g SiO_2, CH_2Cl_2:MeOH, 100:0 to 90:10) gave the title compound **2d** as a white solid (22.4 mg, 0.08 mmol, 39%).

^{1}H NMR (500 MHz, acetone-d$_6$) δ 7.80 (dd, *J* = 7.8, 1.3 Hz, 1H), 7.67 (s, 1H), 7.44 (ddd, *J* = 8.4, 7.2, 1.3 Hz, 1H), 7.22, (dd, *J* = 8.4, 1.0 Hz, 1H), 7.16 (ddd, *J* = 7.8, 7.2, 1.0 Hz, 1H), 6.00 (s, 1H), 3.02 (t, *J* = 7.3 Hz, 2H), 1.78 (quin., *J* = 7.3 Hz, 2H), 1.52 (quin., *J* = 7.3 Hz, 2H), 1.40-1.28 (m, 8H), 0.88 (t, *J* = 7.0 Hz, 3H). **^{13}C NMR (126 MHz, acetone-d$_6$)** δ 157.0, 153.1, 130.9, 125.5, 124.2, 122.8, 119.8, 112.0 (br) 32.7, 31.1, 30.1, 30.0, 29.9, 28.9, 23.4, 14.5. **^{11}B NMR (160 MHz, acetone-d$_6$)** δ 27.1. **HRMS (LDI$^+$):** calculated for ($C_{16}H_{24}{}^{11}BO_2S$ $[M+H]^+$) = 291.1585; found 291.1586.

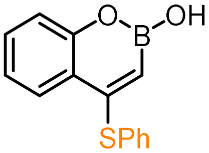

4-(Phenylthio)-2-hydroxy-1,2-benzoxaborinine 2e

Following General Procedure 1a, using 2-ethynylphenol **1a** (24.0 mg, 0.200 mmol), 2,6-di-*tert*-butyl-4-methylpyridine (103.0 mg, 0.500 mmol), thioanisole (70.0 µL, 0.600 mmol) and NaI (60.0 mg, 0.400 mmol) in toluene at 100 °C gave title compound **2e** (88% spectroscopic yield). Purification by flash chromatography (CombiFlash Isco NextGen300+, 4 g SiO_2, CH_2Cl_2:MeOH, 100:0 to 90:10) gave the title compound **2e** as a red solid (35.5 mg, 0.14 mmol, 70%).

Following General Procedure 1b, using 2-ethynylphenol **1a** (24.0 mg, 0.200 mmol), 2,6-di-*tert*-butyl-4-methylpyridine (82.0 mg, 0.400 mmol) and thiophenol (51.0 µL, 0.500 mmol) in *o*-xylene at 120 °C gave title compound **2e** (70% spectroscopic yield). Purification by flash chromatography (CombiFlash Isco NextGen300+, 4 g SiO_2, CH_2Cl_2:MeOH, 100:0 to 90:10) gave the title compound **2e** as a pale yellow oil (25.2 mg, 0.10 mmol, 50%).

^{1}H NMR (500 MHz, acetone-d$_6$+40 uL D_2O) δ 7.87 (dd, *J* = 7.9, 1.5 Hz, 1H), 7.57 (m, 2H), 7.53 (m, 3H), 7.46 (ddd, *J* = 8.4, 7.3, 1.5 Hz, 1H), 7.22, (dd, *J* = 8.4, 1.2 Hz, 1H), 7.16 (ddd, *J* = 7.9, 7.3, 1.2 Hz, 1H), 5.54 (s, 1H). **^{13}C NMR (126 MHz, acetone-d$_6$+40 uL D_2O)** δ 155.9, 151.4, 135.0, 129.5, 129.2, 129.0, 128.7, 123.6, 121.7, 121.2, 118.2, 113.1 (br). **^{11}B NMR (160 MHz, acetone-d$_6$)** δ 27.1. **HRMS (ESI$^-$)** calculated for ($C_{14}H_{11}{}^{11}BO_2SCl$ $[M+Cl]^-$) = 289.0267; found 289.0265.

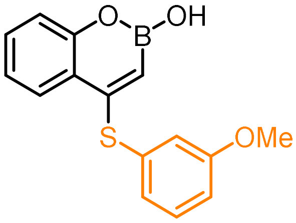

4-(3-Methoxyphenylthio)-2-hydroxy-1,2-benzoxaborinine 2f

Following General Procedure 1a, using 2-ethynylphenol **1a** (24.0 mg, 0.200 mmol), 2,6-di-*tert*-butyl-4-methylpyridine (103.0 mg, 0.500 mmol), 3-methoxythioanisole (92.5 mg, 0.600 mmol) and NaI (60.0 mg, 0.400 mmol) in toluene at 100 °C gave title compound **2f** (90% spectroscopic yield). Purification by flash chromatography (CombiFlash Isco NextGen300+, 4 g SiO_2, CH_2Cl_2:MeOH, 100:0 to 90:10) gave the title compound **2f** as a red solid (42.0 mg, 0.15 mmol, 74%).

^{1}H NMR (500 MHz, acetone-d$_6$+40 uL D$_2$O) δ 7.86 (dd, *J* = 7.9, 1.4 Hz, 1H), 7.46 (ddd, *J* = 8.6, 7.5, 1.4 Hz, 1H), 7.44 (t, *J* = 8.2 Hz, 1H), 7.25, (dd, *J* = 8.6, 1.2 Hz, 1H), 7.16 (ddd, *J* = 7.9, 7.5, 1.2 Hz, 1H), 7.13 (m, 2H), 7.08 (dddd, *J* = 8.2, 3.5, 2.6, 1.0 Hz, 1H), 5.62 (s, 1H), 3.82 (s, 3H). **^{13}C NMR (126 MHz, acetone-d$_6$+40 uL D$_2$O)** δ 160.0, 155.8, 151.6, 130.3, 130.0, 129.7, 127.1, 123.9, 121.9, 121.4, 120.0, 118.4, 115.0, 113.6 (br), 54.5. **^{11}B NMR (160 MHz, acetone-d$_6$)** δ 27.1. **HRMS (ESI$^+$):** calculated for ($C_{15}H_{14}{}^{11}BO_3S$ [M+H]$^+$) = 285.0751; found 285.0746. **HRMS (ESI$^+$):** calculated for ($C_{15}H_{13}{}^{11}BO_3SNa$ [M+Na]$^+$) = 307.0571; found 307.0573.

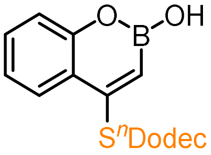

4-(*n*-Dodecylthio)-2-hydroxy-1,2-benzoxaborinine 2g

Following General Procedure 1b, using 2-ethynylphenol **1a** (24.0 mg, 0.200 mmol), 2,6-di-*tert*-butyl-4-methylpyridine (82.0 mg, 0.400 mmol) and 1-dodecanethiol (120.0 µL, 0.500 mmol) in *o*-xylene at 120 °C gave title compound **2g** (82% spectroscopic yield). Purification by flash chromatography (CombiFlash Isco NextGen300+, 4 g SiO_2, CH_2Cl_2:MeOH, 100:0 to 90:10) gave the title compound **2g** as a yellow oily solid (35.7 mg, 0.10 mmol, 52%).

^{1}H NMR (500 MHz, acetone-d$_6$+40 uL D$_2$O) δ 7.77 (dd, *J* = 7.9, 1.4 Hz, 1H), 7.41 (ddd, *J* = 8.3, 7.5, 1.4 Hz, 1H), 7.21, (dd, *J* = 8.3, 1.3 Hz, 1H), 7.15 (ddd, *J* = 7.9, 7.5, 1.3 Hz, 1H), 6.00 (s, 1H), 2.99 (t, *J* = 7.5 Hz, 2H), 1.74 (quin., *J* = 7.5 Hz, 2H), 1.49 (quin., *J* = 6.6 Hz, 2H), 1.34-1.25 (m, 16H), 0.84 (t, *J* = 7.1 Hz, 3H). **^{13}C NMR (126 MHz, acetone-d$_6$+40 uL D$_2$O)** δ 155.2, 151.5, 129.3, 123.8, 122.5, 121.2, 118.3, 110.6 (br), 31.2, 29.6, 28.9, 28.9, 28.8, 28.8, 28.6, 28.5, 28.3, 27.2, 21.9, 12.9. **^{11}B NMR (160 MHz, acetone-d$_6$)** δ 26.8. **HRMS (ESI$^+$):** calculated for ($C_{20}H_{32}{}^{11}BO_2S$ [M+H]$^+$) = 347.22106; found 347.2217. **HRMS (ESI$^+$):** calculated for ($C_{20}H_{31}{}^{11}BO_2SNa$ [M+Na]$^+$) = 369.2030; found 369.2032.

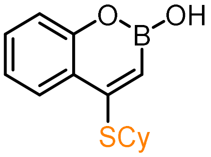

4-(Cyclohexylthio)-2-hydroxy-1,2-benzoxaborinine 2h

Following General Procedure 1b, using 2-ethynylphenol **1a** (24.0 mg, 0.200 mmol), 2,6-di-*tert*-butyl-4-methylpyridine (82.0 mg, 0.400 mmol) and cyclohexanethiol (61.0 µL, 0.500 mmol) in *o*-xylene at 120 °C gave title compound **2h** (58% spectroscopic yield). Purification by flash chromatography (CombiFlash Isco NextGen300+, 4 g SiO_2, CH_2Cl_2:MeOH, 100:0 to 90:10) gave the title compound **2h** as a pale yellow oil (21.2 mg, 0.08 mmol, 42%).

^{1}H NMR (500 MHz, acetone-d$_6$+40 uL D$_2$O) δ 7.80 (dd, *J* = 8.1, 1.5 Hz, 1H), 7.43 (ddd, *J* = 8.3, 7.5, 1.5 Hz, 1H), 7.22, (dd, *J* = 8.3, 1.1 Hz, 1H), 7.16 (ddd, *J* = 8.1, 7.5, 1.1 Hz, 1H), 6.08 (s, 1H), 3.40 (br s, 1H), 2.13 (m, 2H), 1.80 (m, 2H), 1.66 (m, 1H), 1.50 (app t, *J* = 10.1 Hz, 4H), 1.37 (m, 1H). **^{13}C NMR (126 MHz, acetone-d$_6$+40 uL D$_2$O)** δ 154.3, 151.8, 129.5, 124.2, 122.9, 121.3, 118.5, 111.7 (br) 42.0, 32.1, 25.3, 25.2. **^{11}B NMR (160 MHz, acetone-d$_6$)** δ 27.1. **HRMS (ESI$^+$):** calculated for ($C_{14}H_{18}{}^{11}BO_2S$ [M+H]$^+$) = 261.1115; found 261.1119.

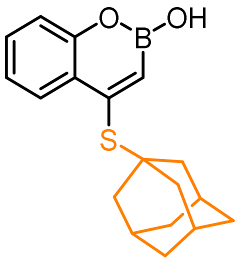

4-(1-Adamantylthio)-2-hydroxy-1,2-benzoxaborinine 2i

Following General Procedure 1b, using 2-ethynylphenol **1a** (24.0 mg, 0.200 mmol), 2,6-di-*tert*-butyl-4-methylpyridine (82.0 mg, 0.400 mmol) and 1-adamantanethiol (84.2 mg, 0.500 mmol) in *o*-xylene at 120 °C gave title compound **2i** (40% spectroscopic yield). Purification by flash chromatography (CombiFlash Isco NextGen300+, 4 g SiO_2, CH_2Cl_2:MeOH, 100:0 to 90:10) gave the title compound **2i** as a brown oil (11.3 mg, 0.04 mmol, 18%).

^{1}H NMR (500 MHz, acetone-d_6+40 uL D_2O) δ 8.17 (dd, *J* = 8.0, 1.7 Hz, 1H), 7.40 (ddd, *J* = 8.1, 7.1, 1.7 Hz, 1H), 7.20, (dd, *J* = 8.1, 1.3 Hz, 1H), 7.16 (ddd, *J* = 8.0, 7.1, 1.3 Hz, 1H), 6.55 (s, 1H), 2.00 (br s, 3H), 1.96, (br s, 3H), 1.95 (br s, 3H), 1.66 (br d, *J* = 2.2 Hz, 6H). **^{13}C NMR (126 MHz, acetone-d_6+40 uL D_2O)** δ 152.2, 149.7, 129.3, 127.4, 125.4, 121.1, 118.1, 48.9, 43.2, 35.3, 29.6. The resonance of the carbon bound to boron was not observed. **^{11}B NMR (160 MHz, acetone-d_6)** δ 26.8. **HRMS (ESI$^+$):** calculated for ($C_{18}H_{21}{}^{11}BO_2SNa$ [M+Na]$^+$) = 335.1248; found 335.1250.

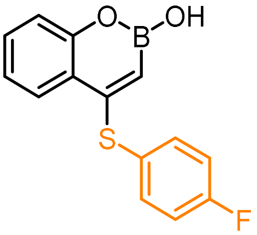

4-(4-Fluorophenylthio)-2-hydroxy-1,2-benzoxaborinine 2j

Following General Procedure 1b, using 2-ethynylphenol **1a** (24.0 mg, 0.200 mmol), 2,6-di-*tert*-butyl-4-methylpyridine (82.0 mg, 0.400 mmol) and 4-fluorothiophenol (53.0 µL, 0.500 mmol) in *o*-xylene at 120 °C gave title compound **2j** (44% spectroscopic yield). Purification by flash chromatography (CombiFlash Isco NextGen300+, 4 g SiO_2, CH_2Cl_2:MeOH, 100:0 to 90:10) gave the title compound **2j** as a red oil (11.8 mg, 0.04 mmol, 22%).

^{1}H NMR (500 MHz, acetone-d_6+40 uL D_2O) δ 7.86 (dd, *J* = 8.0, 1.6 Hz, 1H), 7.64 (dd, *J* = 9.0, 5.3 Hz, 2H), 7.48 (ddd, *J* = 8.5, 7.4, 1.6 Hz, 1H), 7.34 (app t, *J* = 9.0 Hz, 2H), 7.26, (dd, *J* = 7.4, 1.0 Hz, 1H), 7.23 (ddd, *J* = 8.5, 8.0, 1.0 Hz, 1H), 5.48 (s, 1H). **^{13}C NMR (126 MHz, acetone-d_6+40 uL D_2O)** δ 163.5 (d, *J* = 248.2 Hz, CF), 156.5, 151.9, 138.2 (d, *J* = 9.0 Hz), 130.1, 124.6 (d, *J* = 3.0 Hz) 124.0, 122.1, 121.7, 118.7, 116.9 (d, *J* = 22.3 Hz). The resonance of the carbon bound to boron was not observed. **^{11}B NMR (160 MHz, acetone-d_6)** δ 27.1. **^{19}F NMR (471 MHz, acetone-d_6)** δ -112.4 (app sept, *J* = 5.3 Hz). **HRMS (ESI$^-$):** calculated for ($C_{14}H_9{}^{11}BO_2FS$ [M-H]$^-$) = 271.0406; found 271.0412. **HRMS (ESI$^-$):** calculated for ($C_{14}H_{10}{}^{11}BO_2FSCl$ [M+Cl]$^-$) = 307.0173; found 307.0173.

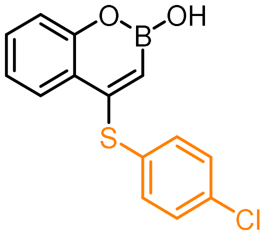

4-(4-Chlorophenylthio)-2-hydroxy-1,2-benzoxaborinine 2k
Following General Procedure 1b, using 2-ethynylphenol **1a** (24.0 mg, 0.200 mmol), 2,6-di-*tert*-butyl-4-methylpyridine (82.0 mg, 0.400 mmol) and 4-chlorothiophenol (72.3 mg, 0.500 mmol) in *o*-xylene at 120 °C gave title compound **2k** (46% spectroscopic yield). Purification by flash chromatography (CombiFlash Isco NextGen300+, 4 g SiO_2, CH_2Cl_2:MeOH, 100:0 to 90:10) gave the title compound **2k** as a clear oil (26.5 mg, 0.09 mmol, 46%).

^{1}H NMR (500 MHz, acetone-d$_6$) δ 7.80 (dd, *J* = 7.9, 1.4 Hz, 1H), 7.76 (s, 1H), 7.50 (d, *J* = 8.5 Hz, 2H), 7.46 (d, *J* = 8.5 Hz, 2H), 7.40 (ddd, *J* = 8.4, 7.4, 1.4 Hz, 1H), 7.20, (dd, *J* = 8.4, 1.0 Hz, 1H), 7.15 (ddd, *J* = 7.9, 7.4, 1.0 Hz, 1H) 5.52 (s, 1H). **^{13}C NMR (126 MHz, acetone-d$_6$)** δ 156.1, 152.1, 137.2, 135.6, 130.1, 129.9, 128.6, 124.4, 122.4, 121.9, 118.9. The resonance of the carbon bound to boron was not observed. **^{11}B NMR (160 MHz, acetone-d$_6$)** δ 32.1. **HRMS (ESI$^+$):** calculated for ($C_{14}H_{11}{}^{11}BO_2ClS$ $[M+H]^+$) = 289.0256; found 289.0244.

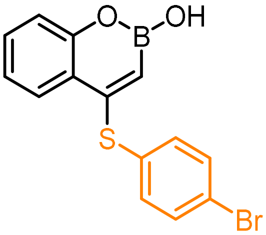

4-(4-Bromophenylthio)-2-hydroxy-1,2-benzoxaborinine 2l
Following General Procedure 1b, using 2-ethynylphenol **1a** (24.0 mg, 0.200 mmol), 2,6-di-*tert*-butyl-4-methylpyridine (82.0 mg, 0.400 mmol) and 4-bromothiophenol (94.5 mg, 0.500 mmol) in *o*-xylene at 120 °C gave title compound **2l** (54% spectroscopic yield). Purification by flash chromatography (CombiFlash Isco NextGen300+, 4 g SiO_2, CH_2Cl_2:MeOH, 100:0 to 90:10) gave the title compound **2l** as a purple oil (28.2 mg, 0.08 mmol, 42%).

^{1}H NMR (500 MHz, acetone-d$_6$+40 uL D_2O) δ 7.85 (dd, *J* = 7.9, 1.4 Hz, 1H), 7.72 (d, *J* = 8.5 Hz, 2H), 7.52 (d, *J* = 8.5 Hz, 2H), 7.48 (ddd, *J* = 8.5, 7.2, 1.4 Hz, 1H), 7.26, (dd, *J* = 8.5, 1.2 Hz, 1H), 7.22 (ddd, *J* = 7.9, 7.2, 1.2 Hz, 1H) 5.59 (s, 1H). **^{13}C NMR (126 MHz, acetone-d$_6$+40 uL D_2O)** δ 155.1, 151.8, 136.9, 132.7, 130.0, 128.8, 124.0, 123.3, 121.9, 121.6, 118.6. The resonance of the carbon bound to boron was not observed. **^{11}B NMR (160 MHz, acetone-d$_6$)** δ 27.0**. HRMS (ESI$^-$):** calculated for ($C_{14}H_9{}^{11}BO_2BrS$ $[M-H]^-$) = 330.9605; found 330.9592.

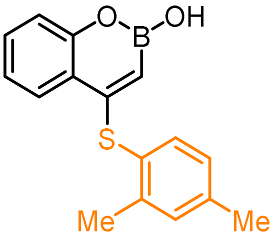

4-(2,4-Dimethylphenylthio)-2-hydroxy-1,2-benzoxaborinine 2m
Following General Procedure 1b, using 2-ethynylphenol **1a** (24.0 mg, 0.200 mmol), 2,6-di-*tert*-butyl-4-methylpyridine (82.0 mg, 0.400 mmol) and 2,4-dimethylbenzenethiol (68.0 µL, 0.500 mmol) in *o*-xylene at 120 °C gave title compound **2m** (44% spectroscopic yield). Purification by flash chromatography (CombiFlash Isco NextGen300+, 4 g SiO_2,

CH_2Cl_2:MeOH, 100:0 to 90:10) gave the title compound **2m** as a clear oil (10.5 mg, 0.04 mmol, 19%).

^{1}H NMR (500 MHz, acetone-d$_6$+40 uL D$_2$O) δ 7.91 (dd, *J* = 7.9, 1.5 Hz, 1H), 7.47 (ddd, *J* = 8.6, 7.2, 1.5 Hz, 1H), 7.43 (d, *J* = 7.9 Hz, 1H), 7.30 (s, 1H), 7.26-7.22, (m, 2H), 7.17 (d, *J* = 7.7 Hz, 1H), 5.28 (s, 1H), 2.37 (s, 3H), 2.32 (s, 3H). **^{13}C NMR (126 MHz, acetone-d$_6$+40 uL D$_2$O)** δ 155.6, 152.0, 142.8, 140.6, 136.9, 131.7, 129.9, 127.9, 124.5, 124.2, 122.3, 121.7, 118.6, 20.1, 19.3. The resonance of the carbon bound to boron was not observed. **^{11}B NMR (160 MHz, acetone-d$_6$)** δ 27.1. **HRMS (LDI$^+$)** calculated for ($C_{16}H_{16}{}^{11}BO_2S$ $[M+H]^+$) = 283.0959; found 283.0961.

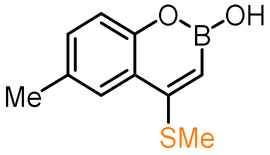

4-(Methylthio)-2-hydroxy-6-methyl-1,2-benzoxaborinine 2n

Following General Procedure 1a, using 2-ethynyl-4-methylphenol **1b** (26.4 mg, 0.200 mmol), 2,6-di-*tert*-butyl-4-methylpyridine (103.0 mg, 0.500 mmol) and dimethyl sulfide (44.0 µL, 0.600 mmol) in toluene at 100 °C gave title compound **2n** (86% spectroscopic yield). Purification by flash chromatography (CombiFlash Isco NextGen300+, 4 g SiO_2, CH_2Cl_2:MeOH, 100:0 to 90:10) gave the title compound **2n** as a white solid (26.1 mg, 0.13 mmol, 63%).

^{1}H NMR (500 MHz, acetone-d$_6$+40 uL D$_2$O) δ 7.54 (d, *J* = 1.2 Hz, 1H), 7.24 (dd, *J* = 8.5, 1.2 Hz, 1H), 7.11 (d, *J* = 8.5 Hz, 1H), 5.92 (s, 1H), 2.47 (s, 3H), 2.35 (s, 3H). **^{13}C NMR (126 MHz, acetone-d$_6$+40 uL D$_2$O)** δ 156.5, 149.9, 130.9, 130.6, 124.1, 122.5, 118.5, 110.3 (br), 19.9, 12.8. **^{11}B NMR (160 MHz, acetone-d$_6$)** δ 27.0. **HRMS (LDI$^+$):** calculated for ($C_{10}H_{12}{}^{11}BO_2S$ $[M+H]^+$) = 207.0646; found 207.0646.

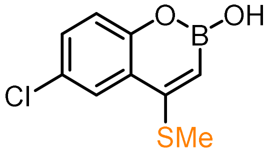

4-(Methylthio)-6-chloro-2-hydroxy-1,2-benzoxaborinine 2o

Following General Procedure 1a, using 4-chloro-2-ethynylphenol **1c** (30.5 mg, 0.200 mmol), 2,6-di-*tert*-butyl-4-methylpyridine (103.0 mg, 0.500 mmol) and dimethyl sulfide (44.0 µL, 0.600 mmol) in toluene at 100 °C gave title compound **2o** (92% spectroscopic yield). Purification by flash chromatography (CombiFlash Isco NextGen300+, 4 g SiO_2, CH_2Cl_2:MeOH, 100:0 to 90:10) gave the title compound **2o** as a white solid (32.1 mg, 0.14 mmol, 71%).

^{1}H NMR (500 MHz, acetone-d$_6$) δ 7.86 (s, 1H), 7.71 (d, *J* = 2.5 Hz, 1H), 7.44 (dd, *J* = 8.7, 2.5 Hz, 1H), 7.25 (d, *J* = 8.7 Hz, 1H), 6.01 (s, 1H), 2.52 (s, 3H). **^{13}C NMR (126 MHz, acetone-d$_6$+40 uL D$_2$O)** δ 155.0, 150.2, 129.2, 126.0, 123.9, 123.1, 120.3, 111.6 (br), 12.6. **^{11}B NMR (160 MHz, acetone-d$_6$)** δ 26.9. **HRMS (ESI$^+$):** calculated for ($C_9H_8{}^{11}BClO_2S$ $[M]^+$) = 226.0027; found 226.0022. **HRMS (ESI$^+$):** calculated for ($C_9H_9{}^{11}BClO_2S$ $[M+H]^+$) = 227.0099; found 227.0100.

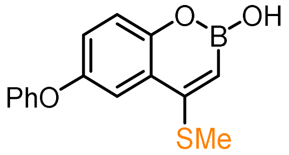

4-(Methylthio)-6-phenoxy-2-hydroxy-1,2-benzoxaborinine 2p

Following General Procedure 1a, using 4-phenoxy-2-ethynylphenol **1d** (42.0 mg, 0.200 mmol), 2,6-di-*tert*-butyl-4-methylpyridine (103.0 mg, 0.500 mmol) and dimethyl sulfide (44.0 µL, 0.600 mmol) in toluene at 100 °C gave title compound **2p** (>99% spectroscopic yield). Purification by flash chromatography (CombiFlash Isco NextGen300+, 4 g SiO_2, CH_2Cl_2:MeOH, 100:0 to 90:10) gave the title compound **2p** as a brown oil (39.7 mg, 0.14 mmol, 70%).

^{1}H NMR (500 MHz, acetone-d$_6$+40 uL D$_2$O) δ 7.37-7.34 (m, 3H), 7.26 (d, *J* = 9.0 Hz, 1H), 7.13 (dd, *J* = 9.0, 3.1 Hz, 1H), 7.11-7.08 (m, 1H), 6.99-6.97 (m, 2H), 5.99 (s, 1H), 2.45 (s, 3H). **^{13}C NMR (126 MHz, acetone-d$_6$+40 uL D$_2$O)** δ 158.8, 156.8, 151.8, 148.9, 130.7, 124.5, 123.9, 122.1, 120.9, 118.8, 115.0, 112.2 (br), 13.7. **^{11}B NMR (160 MHz, acetone-d$_6$)** δ 27.0. **HRMS (LDI$^+$):** calculated for ($C_{15}H_{13}{}^{11}BO_3S$ [M]$^+$) = 284.0679; found 284.0674.

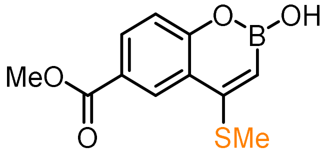

4-(Methylthio)-6-(methoxycarbonyl)-2-hydroxy-1,2-benzoxaborinine 2q

Following General Procedure 1a, using methyl-3-ethynyl-4-hydroxybenzoate **1e** (35.2 mg, 0.200 mmol) 2,6-di-*tert*-butyl-4-methylpyridine (103.0 mg, 0.500 mmol) and dimethyl sulfide (44.0 µL, 0.600 mmol) in toluene at 100 °C gave title compound **2q** (90% spectroscopic yield). Purification by flash chromatography (CombiFlash Isco NextGen300+, 4 g SiO_2, CH_2Cl_2:MeOH, 100:0 to 90:10) gave the title compound **2q** as a white solid (20.9 mg, 0.08 mmol, 42%).

^{1}H NMR (500 MHz, acetone-d$_6$+40 uL D$_2$O) δ 8.44 (d, *J* = 2.1 Hz, 1H), 8.05 (dd, *J* = 8.6, 2.1 Hz, 1H), 7.32 (d, *J* = 8.6 Hz, 1H), 6.03 (s, 1H), 3.89 (s, 3H), 2.52 (s, 3H). **^{13}C NMR (126 MHz, acetone-d$_6$+40 uL D$_2$O)** δ 165.6, 156.0, 155.1, 130.5, 125.8, 123.5, 122.5, 119.0, 111.1 (br), 51.4, 12.6. **^{11}B NMR (160 MHz, acetone-d$_6$)** δ 26.8. **HRMS (ESI$^+$):** calculated for ($C_{11}H_{11}{}^{11}BO_4SNa$ [M+Na]$^+$) = 273.0363; found 273.0363.

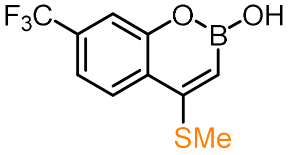

4-(Methylthio)-7-(trifluoromethyl)-2-hydroxy-1,2-benzoxaborinine 2r

Following General Procedure 1a, using 5-(trifluoromethyl)-2-ethynylphenol **1f** (37.2 mg, 0.200 mmol), 2,6-di-*tert*-butyl-4-methylpyridine (103.0 mg, 0.500 mmol) and dimethyl sulfide (44.0 µL, 0.600 mmol) in toluene at 100 °C gave title compound **2r** (92% spectroscopic yield). Purification by flash chromatography (CombiFlash Isco NextGen300+, 4 g SiO_2, CH_2Cl_2:MeOH, 100:0 to 90:10) gave the title compound **2r** as a brown solid (35.0 mg, 0.13 mmol, 67%).

^{1}H NMR (500 MHz, acetone-d$_6$) δ 7.93 (d, *J* = 8.4 Hz, 1H), 7.51 (s, 1H), 7.48 (d, *J* = 8.4 Hz, 1H), 6.10 (s, 1H), 2.52 (s, 3H). **^{13}C NMR (126 MHz, acetone-d$_6$)** δ 155.6, 151.7, 130.6 (q, *J* = 32.9 Hz), 125.9, 125.3, 123.8 (q, *J* = 271.4 Hz), 118.0 (q, *J* = 3.6 Hz), 115.7 (q, *J* = 3.9 Hz),

113.0 (br), 12.9. **^{11}B NMR (160 MHz, acetone-d$_6$)** δ 26.9. **^{19}F NMR (471 MHz, acetone-d$_6$)** δ -63.1. **HRMS (LDI$^+$):** calculated for ($C_{10}H_8{}^{11}BF_3O_2S$ [M]$^+$) = 260.0290; found 260.0288. **HRMS (LDI$^+$):** calculated for ($C_{10}H_9{}^{11}BF_3O_2S$ [M+H]$^+$) = 261.0363; found 261.0367.

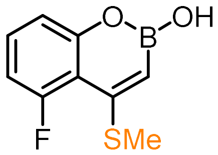

4-(Methylthio)-5-fluoro-2-hydroxy-1,2-benzoxaborinine 2s

Following General Procedure 1a, using 3-fluoro-2-ethynylphenol **1g** (27.2 mg, 0.200 mmol), 2,6-di-*tert*-butyl-4-methylpyridine (103.0 mg, 0.500 mmol) and dimethyl sulfide (44.0 µL, 0.600 mmol) in toluene at 100 °C gave title compound **2s** (78% spectroscopic yield). Purification by flash chromatography (CombiFlash Isco NextGen300+, 4 g SiO_2, CH_2Cl_2:MeOH, 100:0 to 90:10) gave the title compound **2s** as a white solid (23.3 mg, 0.11 mmol, 55%).

^{1}H NMR (500 MHz, acetone-d$_6$+40 uL D$_2$O) δ 7.41 (td, *J* = 8.3, 6.0 Hz, 1H), 7.08, (dt, *J* = 8.3, 1.1 Hz, 1H), 6.93, (ddd, *J* = 12.3, 8.3, 1.1 Hz, 1H), 5.92 (s, 1H), 2.42 (s, 3H). **^{13}C NMR (126 MHz, acetone-d$_6$)** δ 160.3 (d, *J* = 252.0 Hz), 155.9 (d, *J* = 7.2 Hz), 154.2 (d, *J* = 6.9 Hz), 130.2 (d, *J* = 10.9 Hz), 115.7 (d, *J* = 3.4 Hz), 112.9 (d, *J* = 13.5 Hz), 111.5 (br), 109.5 (d, *J* = 24.5 Hz), 14.6 (d, *J* = 12.2 Hz). The resonance of the carbon bound to boron was not observed. **^{11}B NMR (160 MHz, acetone-d$_6$)** δ 26.6. **^{19}F NMR (471 MHz, acetone-d$_6$)** δ -107.9 (dd, *J* = 12.3, 6.0 Hz). **HRMS (LDI$^+$):** calculated for ($C_9H_8{}^{11}BFO_2S$ [M]$^+$) = 210.0322; found 210.0318. **HRMS (LDI$^+$):** calculated for ($C_9H_9{}^{11}BFO_2S$ [M+H]$^+$) = 211.0395; found 211.0396. **HRMS (LDI$^+$):** calculated for ($C_9H_8{}^{11}BFO_2SNa$ [M+Na]$^+$) = 233.0214; found 233.0215.

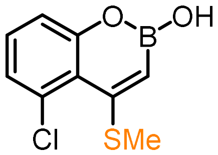

4-(Methylthio)-5-chloro-2-hydroxy-1,2-benzoxaborinine 2t

Following General Procedure 1a, using 3-chloro-2-ethynylphenol **1h** (30.5 mg, 0.200 mmol), 2,6-di-*tert*-butyl-4-methylpyridine (103.0 mg, 0.500 mmol) and dimethyl sulfide (44.0 µL, 0.600 mmol) in toluene at 100 °C gave title compound **2t** (60% spectroscopic yield). Purification by flash chromatography (CombiFlash Isco NextGen300+, 4 g SiO_2, CH_2Cl_2:MeOH, 100:0 to 90:10) gave the title compound **2t** as a white solid (24.9 mg, 0.11 mmol, 55%).

^{1}H NMR (500 MHz, acetone-d$_6$+40 uL D$_2$O) δ 7.36 (app t, *J* = 8.0 Hz, 1H), 7.24-7.21, (m, 2H), 6.05 (s, 1H), 2.41 (s, 3H). **^{13}C NMR (126 MHz, acetone-d$_6$)** δ 158.0, 154.3, 131.0, 129.4, 125.6, 121.6, 118.9, 16.1. The resonance of the carbon bound to boron was not observed. **^{11}B NMR (160 MHz, acetone-d$_6$)** δ 26.4. **HRMS (ESI$^+$):** calculated for ($C_9H_8{}^{11}BClO_2S$ [M+H]$^+$) = 227.0099; found 227.0104.

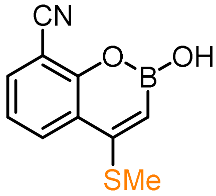

4-(Methylthio)-8-cyano-2-hydroxy-1,2-benzoxaborinine 2u

Following General Procedure 1a, using 3-ethynyl-2-hydroxybenzonitrile **1i** (28.6 mg, 0.200 mmol), 2,6-di-*tert*-butyl-4-methylpyridine (103.0 mg, 0.500 mmol) and dimethyl sulfide (50.0 µL, 0.681 mmol) in toluene at 100 °C gave title compound **2u** (94% spectroscopic yield). Purification by flash chromatography (CombiFlash Isco NextGen300+, 4 g SiO_2, CH_2Cl_2:MeOH, 100:0 to 90:10) gave the title compound **2u** as a white solid (31.2 mg, 0.14 mmol, 72%).

^{1}H NMR (500 MHz, acetone-d$_6$+40 uL D_2O) δ 8.01 (dd, *J* = 7.7, 1.6 Hz, 1H), 7.82 (d, *J* = 7.7, 1.6 Hz, 1H), 7.34 (t, *J* = 7.7, Hz, 1H), 6.07 (s, 1H), 2.52 (s, 3H). **^{13}C NMR (126 MHz, acetone-d$_6$+40 uL D_2O)** δ 155.5, 152.8, 133.2, 128.8, 123.4, 121.9, 115.2, 111.7 (br), 102.9, 12.6. **^{11}B NMR (160 MHz, acetone-d$_6$)** δ 26.8. **HRMS (LDI$^+$):** calculated for ($C_{10}H_9{}^{11}BO_2S$ $[M+H]^+$) = 218.0442; found 218.0442.

S2.2.2 C3-Borylated Benzofuran From an Internal Alkyne

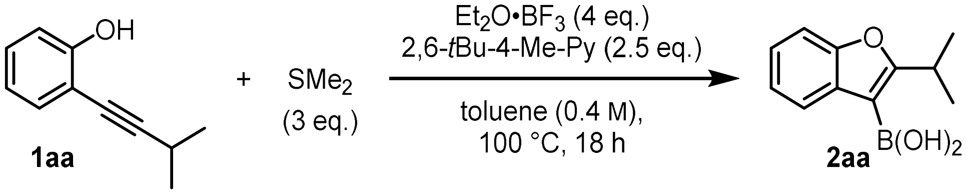

(2-Isopropylbenzofuran-3-yl)boronic acid 2aa

Following General Procedure 1a, using **1aa** (32.0 mg, 0.200 mmol), 2,6-di-*tert*-butyl-4-methylpyridine (103.0 mg, 0.500 mmol) and dimethyl sulfide (44.0 µL, 0.600 mmol) in toluene at 100 °C gave compound **2aa** (40% spectroscopic yield). Purification by flash chromatography (CombiFlash Isco NextGen300+, 4 g SiO_2, CH_2Cl_2:MeOH, 100:0 to 90:10) gave compound **2aa** as a white solid (15.2 mg, 0.07 mmol, 37%).

^{1}H NMR (500 MHz, acetone-d$_6$+40 uL D_2O+$CDCl_3$) δ 7.82-7.80 (m, 1H), 7.32-7.30 (m, 1H), 7.11-7.06 (m, 2H), 3.73 (sept., *J* = 7.0 Hz, 1H), 1.27 (d, *J* = 7.0 Hz, 6H). **^{13}C NMR (126 MHz, acetone-d$_6$+40 uL D_2O+$CDCl_3$)** δ 171.4, 153.9, 132.0, 122.1, 122.0, 121.5, 109.4, 27.2, 20.6. **^{11}B NMR (160 MHz, acetone-d$_6$+$CDCl_3$)** δ 28.5. **HRMS (ESI$^+$):** calculated for ($C_{11}H_{14}{}^{11}BO_3$ $[M+H]^+$) = 205.1031; found 205.1034.

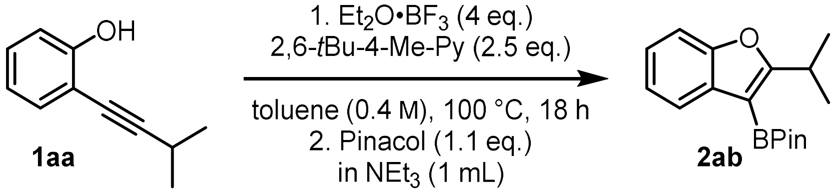

4,4,5,5-Tetramethyl-2-(2-isopropylbenzofuran-3-yl)-1,3,2-dioxaborolane 2ab

Following General Procedure 1a, using **1aa** (32.0 mg, 0.200 mmol), 2,6-di-*tert*-butyl-4-methylpyridine (103.0 mg, 0.500 mmol) in toluene at 100 °C. A work-up using pinacol (30.0 mg, 0.220 mmol) and NEt_3 (1 mL) gave compound **2ab** (52% spectroscopic yield). Purification by flash chromatography (CombiFlash Isco NextGen300+, 4 g SiO_2, CH_2Cl_2:MeOH, 100:0 to 90:10) gave compound **2ab** as a white solid (22.1 mg, 0.08 mmol, 39%).

^{1}H NMR (500 MHz, CDCl$_3$) δ 7.87-7.85 (m, 1H), 7.43-7.41 (m, 1H), 7.21-7.20 (m, 2H), 3.67 (sept., J = 6.9 Hz, 1H), 1.37-1.35 (overlapped d, 6H) 1.37-1.35 (overlapped s, 12H). **^{13}C NMR (126 MHz, CDCl$_3$)** δ 174.1, 154.7, 132.2, 123.2, 122.7, 122.4, 110.5, 83.1, 28.4, 25.1, 21.6. **^{11}B NMR (160 MHz, CDCl$_3$)** δ 30.1. **HRMS (ESI$^+$):** calculated for ($C_{17}H_{24}{}^{11}BO_3$ [M+H]$^+$) = 287.1813; found 287.1814.

S2.2.3 Thioboration of *N*-Benzyl-2-ethynylaniline

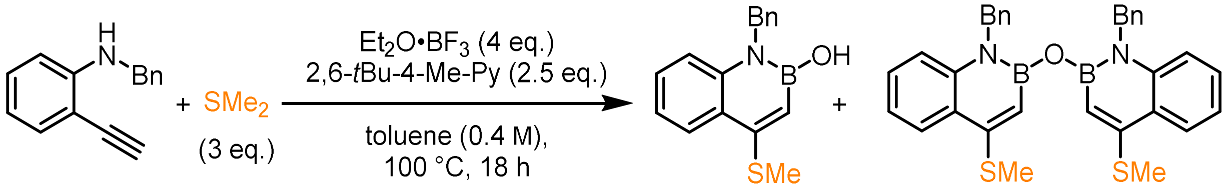

3 **4a** 72% (1.3:1) **4a-anhydride**

4-(Methylthio)-2-hydroxy-1-benzyl-1,2-benzazaborinine 4a

Following General Procedure 1a, using *N*-benzyl-2-ethynylaniline **3** (41.5 mg, 0.200 mmol), 2,6-di-*tert*-butyl-4-methylpyridine (103.0 mg, 0.500 mmol) and dimethyl sulfide (44.0 µL, 0.600 mmol) in toluene at 100 °C gave azaborinine as a 1.3:1 mixture of monomer **4a** and anhydride **4a-anhydride** (72% spectroscopic yield). Purification by flash chromatography (CombiFlash Isco NextGen300+, 4 g SiO_2, PE:EtOAc, 100:0 to 0:100) gave the monomer **4a** (19.1 mg, 0.07 mmol, 34%) and anhydride **4a-anhydride** as a white solid (11.6 mg, 0.03 mmol, 15%). Heating the anhydride in acetone-d$_6$ (0.5 mL) and D_2O (0.1 mL) at 80 °C for 2 h gave the monomer **4a** for an overall yield (30.7 mg, 0.11 mmol, 55%).

*Boronic acid monomer **4a**:* **^{1}H NMR (500 MHz, acetone-d$_6$, D$_2$O)** δ 7.85 (dd, J = 8.1, 1.4 Hz, 1H), 7.25-7.17 (m, 6H), 7.15-7.12 (m, 1H), 6.95 (ddd, J = 8.1, 7.0, 1.3 Hz, 1H), 6.28 (s, 1H), 5.14 (s, 2H), 2.45 (s, 3H). **^{13}C NMR (126 MHz, acetone-d$_6$, D$_2$O)** δ 150.8, 139.7, 137.3, 126.4, 126.1, 124.3, 124.1, 122.7, 120.6, 116.4, 113.3, 43.2, 10.0. The resonance of the carbon bound to boron was not observed. **^{11}B NMR (160 MHz, acetone-d$_6$, D$_2$O)** δ 27.9. **HRMS (ESI$^+$):** calculated for ($C_{16}H_{17}{}^{11}BNOS$ [M+H]$^+$) = 282.1118; found 282.1120.

*Boronic anhydride **4a-anhydride**:* **^{1}H NMR (500 MHz, CDCl$_3$)** δ 8.05 (dd, J = 8.0, 1.2 Hz, 2H), 7.32 (ddd, J = 8.6, 7.3, 1.2 Hz 2H), 7.21 (app d, J = 8.3 Hz, 2H), 7.18-7.15 (m, 6H), 7.09 (ddd, J = 8.1, 7.3, 0.9 Hz, 2H), 7.05 (m, 4H), 6.43 (s, 2H), 5.13 (s, 4H), 2.51 (s, 3H). **^{13}C NMR (126 MHz, CDCl$_3$)** δ 155.4, 142.0, 138.9, 129.3, 128.6, 126.7, 126.2, 125.6, 123.4, 119.7, 116.1, 46.9, 14.7. The resonance of the carbon bound to boron was not observed. **^{11}B NMR (160 MHz, CDCl$_3$)** δ 27.6. **HRMS (ESI$^+$):** calculated for ($C_{32}H_{31}{}^{11}B_2N_2OS_2$ [M+H]$^+$) = 545.2059; found 545.2069.

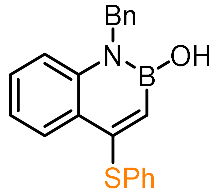

4-(Phenylthio)-2-hydroxy-1-benzyl-1,2-benzazaborinine 4b

Following General Procedure 1b, using *N*-benzyl-2-ethynylaniline **3** (0.200 mmol, 41.5 mg), 2,6-di-*tert*-butyl-4-methylpyridine (82.0 mg, 0.400 mmol) and thiophenol (51.0 µL, 0.500 mmol) in *o*-xylene at 120 °C gave azaborinine **4b** (47% spectroscopic yield). Purification by flash chromatography (CombiFlash Isco NextGen300+, 4 g SiO_2, PE:EtOAc, 100:0 to 0:100) gave a 1.1:1 mixture of monomer **4b** and anhydride **4b-anhydride** as a brown oil (30.6 mg,

0.09 mmol, 45%). Heating the mixture in acetone-d6 (0.5 mL) and D_2O (0.1 mL) at 80 °C for 2 h gave the monomer **4b** exclusively.

*Boronic acid monomer **4b**:* **^{1}H NMR (500 MHz, acetone-d6, D_2O)** δ 7.95 (dd, *J* = 8.1, 1.4 Hz, 1H), 7.45-7.41 (m, 5H), 7.26-7.23 (m, 1H), 7.22-7.11 (m, 3H), 7.15-7.11 (m, 3H), 6.97 (app t, *J* = 8.0, 1H), 6.05 (s, 1H), 5.10 (s, 2H). **^{13}C NMR (126 MHz, acetone-d6, D_2O)** δ 148.0, 138.3, 135.2, 130.3, 127.5, 125.7, 125.0, 124.9, 124.4, 122.6, 122.2, 121.6, 118.4, 115.0, 111.7, 41.6. The resonance of the carbon bound to boron was not observed. **^{11}B NMR (160 MHz, acetone-d6, D_2O)** δ 28.3. **HRMS (LDI):** calculated for ($C_{21}H_{18}{}^{11}BNOS$ [M]) = 343.1202; found 342.1191. **HRMS (LDI):** calculated for ($C_{21}H_{17}{}^{11}BNOS$ [M-H]) = 342.1129; found 342.1122.

S2.2.4 General Procedure 2: Carboboration of *o*-Alkynyl Phenols with C-Nucleophiles

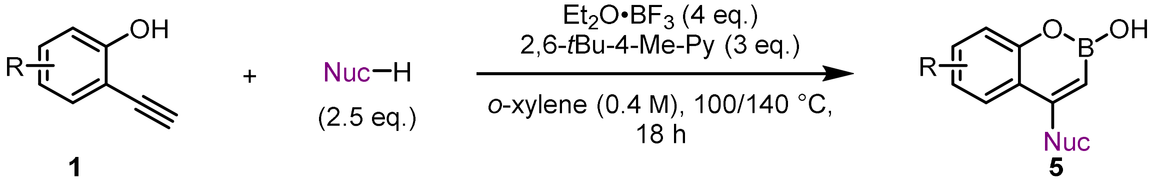

Same as General Procedure 1, with 2-ethynylphenol **1** (24.0 mg, 0.200 mmol), $Et_2O{\cdot}BF_3$ (100 µL, 0.800 mmol), 2,6-di-*tert*-butyl-4-methylpyridine (103.0 mg, 0.600 mmol) and nucleophile (0.500 mmol) in *o*-xylene (0.500 mL) at the specified temperature for 18 h.

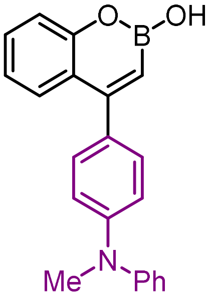

4-[4-(*N*-Methyl-*N*-phenylamino)phenyl]-2-hydroxy-1,2-benzoxaborinine 5a

Following General Procedure 2, using 2-ethynylphenol **1a** (24.0 mg, 0.200 mmol) 2,6-di-*tert*-butyl-4-methylpyridine (123.0 mg, 0.600 mmol) and *N*-methyldiphenylamine (87.2 µL, 0.500 mmol) in *o*-xylene at 150 °C gave title compound **5a** (72% spectroscopic yield). Purification by flash chromatography (CombiFlash Isco NextGen300+, 4 g SiO_2, CH_2Cl_2:MeOH, 100:0 to 90:10) gave the title compound **5a** as a yellow oil (35.1 mg, 0.11 mmol, 54%).

^{1}H NMR (500 MHz, acetone-d6 +40 uL D_2O) δ 7.46 (dd, *J* = 8.0, 1.6 Hz, 1H), 7.38 (ddd, *J* = 8.5, 7.3, 1.9 Hz, 1H), 7.33 (dd, *J* = 8.5, 7.6 Hz, 2H), 7.28 (d, *J* = 8.3 Hz, 2H), 7.27, (ddd, *J* = 8.5, 7.3, 1.3 Hz, 2H), 7.15 (app d, *J* = 7.5 Hz, 2H), 7.09-7.03 (m, 1H), 7.02 (d, *J* = 8.3 Hz, 2H), 6.06 (s, 1H), 3.34 (s, 3H). **^{13}C NMR (126 MHz, acetone-d6+40 uL D_2O)** δ 158.9, 152.8, 148.4, 148.1, 131.6, 128.8, 128.6, 126.8, 123.4, 122.1, 121.8, 121.0, 119.5, 118.3, 117.3, 39.1. The resonance of the carbon bound to boron was not observed. **^{11}B NMR (160 MHz, acetone-d6)** δ 27.6. **HRMS (LDI$^+$):** calculated for ($C_{21}H_{19}{}^{11}BNO_2$ [M+H]$^+$) = 328.1503; found 328.1503.

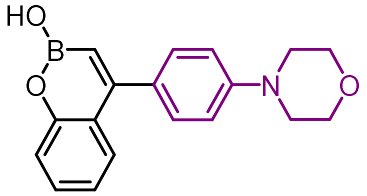

4-[4-(morpholinyl)phenyl]-2-hydroxy-1,2-benzoxaborinine 5b

Following General Procedure 2, using 2-ethynylphenol **1a** (24.0 mg, 0.200 mmol), 2,6-di-*tert*-butyl-4-methylpyridine (123.0 mg, 0.600 mmol) and 4-phenylmorpholine (81.6 mg, 0.500 mmol) in *o*-xylene at 100 °C gave title compound **5b** (56% spectroscopic yield). Purification by flash chromatography (CombiFlash Isco NextGen300+, 4 g SiO_2, CH_2Cl_2:MeOH, 100:0 to 90:10) gave the title compound **5b** as a brown oil (17.2 mg, 0.06 mmol, 28%).
^{1}H NMR (500 MHz, acetone-d$_6$+40 uL D_2O) δ 7.43 (dd, *J* = 8.1, 1.6 Hz, 1H), 7.39 (ddd, *J* = 8.1, 7.3, 1.6 Hz, 1H), 7.29 (d, *J* = 7.3 Hz, 2H), 7.26 (dd, *J* = 7.9, 0.9 Hz, 1H), 7.09-7.06, (m, 1H), 7.06 (d, *J* = 7.3 Hz, 2H), 6.03 (s, 1H), 3.80 (t, *J* = 4.8 Hz, 4H), 3.21 (t, *J* = 4.8 Hz, 4H). **^{13}C NMR (126 MHz, acetone-d$_6$+40 uL D_2O)** δ 159.3, 153.1, 151.0, 131.2, 129.1, 128.9, 127.1, 123.8, 121.2, 118.5, 114.6 66.2, 48.4 The resonance of the carbon bound to boron was not observed. **^{11}B NMR (160 MHz, acetone-d$_6$)** δ 27.6. **HRMS (LDI$^+$):** calculated for ($C_{18}H_{18}{}^{11}BNO_3$ $[M]^+$) = 307.1380; found 307.1378.

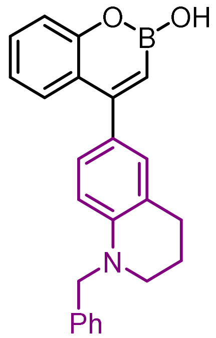

4-(1-Benzyl-1,2,3,4-tetrahydroquin-6-yl)-2-hydroxy-1,2-benzoxaborinine 5c
Following General Procedure 2, using 2-ethynylphenol **1a** (24.0 mg, 0.200 mmol), 2,6-di-*tert*-butyl-4-methylpyridine (123.0 mg, 0.600 mmol) and 1-benzyl-1,2,3,4-tetrahydroquinoline (111.7 mg, 0.500 mmol) in *o*-xylene at 100 °C gave title compound **5c** (46% spectroscopic yield). Purification by flash chromatography (CombiFlash Isco NextGen300+, 4 g SiO_2, CH_2Cl_2:MeOH, 100:0 to 90:10) gave the title compound **5c** as a brown oil (24.7 mg, 0.07 mmol, 34%).

^{1}H NMR (500 MHz, acetone-d$_6$+40 uL D_2O) δ 7.52 (dd, *J* = 7.9, 1.8 Hz, 1H), 7.38 (ddd, *J* = 8.4, 7.3, 1.8 Hz, 1H), 7.33-7.30 (m, 4H), 7.23 (dd, *J* = 7.9, 1.0 Hz, 2H), 7.05, (ddd, *J* = 8.3, 7.4, 1.4 Hz, 1H), 7.00-6.98 (m, 1H), 6.97 (dd, *J* = 8.2, 2.3 Hz, 1H), 6.57 (d, *J* = 8.7 Hz, 1H), 5.97 (s, 1H), 4.56 (s, 2H), 3.46 (t, *J* = 6.4 Hz, 2H), 2.84 (t, *J* = 6.4 Hz, 2H), 2.02 (quin., *J* = 6.4 Hz, 2H). **^{13}C NMR (126 MHz, acetone-d$_6$+40 uL D_2O)** δ 159.7, 153.2, 145.2, 138.8, 128.9, 128.8, 128.3, 127.6, 127.3, 127.0, 126.4, 126.3, 124.0, 121.6, 121.1, 118.5, 110.2, 54.3, 49.6, 27.7, 21.8. The resonance of the carbon bound to boron was not observed. **^{11}B NMR (160 MHz, acetone-d$_6$)** δ 27.3. **HRMS (LDI$^+$):** calculated for ($C_{24}H_{23}{}^{11}BNO_2$ $[M+H]^+$) = 368.1816; found 368.1825.

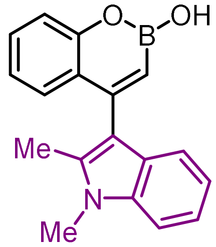

4-(1,2-Dimethyl-3-indolyl)-2-hydroxy-1,2-benzoxaborinine 5d
Following General Procedure 2, using 2-ethynylphenol **1a** (24.0 mg, 0.200 mmol), 2,6-di-*tert*-butyl-4-methylpyridine (123.0 mg, 0.600 mmol) and 1,2-dimethylindole (72.6 mg, 0.500 mmol) in *o*-xylene at 150 °C gave title compound **5d** (61% spectroscopic yield). Purification

by flash chromatography (CombiFlash Isco NextGen300+, 4 g SiO_2, CH_2Cl_2:MeOH, 100:0 to 90:10) gave the title compound **5d** as a brown oil (22.8 mg, 0.08 mmol, 39%).

Gram-scale reaction: Following General Procedure 3, using 2-ethynylphenol **1a** (1.00 g, 8.46 mmol), 2,6-di-*tert*-butyl-4-methylpyridine (5.21 g, 0.600 mmol) and 1,2-dimethylindole (3.07 g, 0.500 mmol) in *o*-xylene at 140 °C. Purification by flash chromatography (CombiFlash Isco NextGen300+, 40 g SiO_2, CH_2Cl_2:MeOH, 100:0 to 90:10) gave the title compound **5d** as a brown solid (1.27 g, 4.38 mmol, 52%).

^{1}H NMR (500 MHz, acetone-d$_6$+40 uL D_2O) δ 7.41 (d, *J* = 8.3 Hz, 1H), 7.37 (ddd, *J* = 8.3, 7.1, 1.7 Hz, 1H), 7.29 (ddd, *J* = 9.8, 8.4, 1.4 Hz, 2H), 7.17 (d, *J* = 7.4 Hz, 1H), 7.13, (ddd, *J* = 8.1, 7.0, 1.0 Hz, 1H), 6.99-6.95 (m, 2H), 6.12 (s, 1H), 3.79 (s, 3H), 2.34 (s, 3H). **^{13}C NMR (126 MHz, acetone-d$_6$+40 uL D_2O)** δ 153.1, 152.8, 136.2, 134.0, 128.7, 127.4, 126.7, 124.2, 120.8, 120.3, 118.8, 118.2, 118.1, 111.6, 108.5, 28.5, 10.0. The resonance of the carbon bound to boron was not observed. **^{11}B NMR (160 MHz, acetone-d$_6$)** δ 27.8. **HRMS (LDI$^+$):** calculated for ($C_{18}H_{16}{}^{11}BNO_2$ $[M]^+$) = 289.1269; found 289.1269. **HRMS (LDI$^+$):** calculated for ($C_{18}H_{17}{}^{11}BNO_2$ $[M+H]^+$) = 290.1347; found 290.1348.

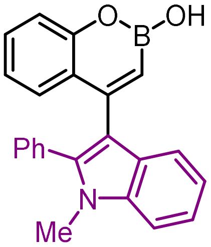

4-(1-Methyl-2-phenyl-3-indolyl)-2-hydroxy-1,2-benzoxaborinine 5e

Following General Procedure 2, using 2-ethynylphenol **1a** (24.0 mg, 0.200 mmol), 2,6-di-*tert*-butyl-4-methylpyridine (123.0 mg, 0.600 mmol) and 1-methyl-2-phenylindole (103.6 mg, 0.500 mmol) in *o*-xylene at 140 °C gave title compound **5e** (36% spectroscopic yield). Purification by flash chromatography (CombiFlash Isco NextGen300+, 4 g SiO_2, CH_2Cl_2:MeOH, 100:0 to 90:10) gave the title compound **5e** as an orange oil (18.0 mg, 0.05 mmol, 26%).

^{1}H NMR (500 MHz, acetone-d$_6$+40 uL D_2O) δ 7.54 (d, *J* = 8.3 Hz, 1H), 7.41-7.24 (m, 2H), 7.37-7.24 (m, 7H), 7.19 (dd, *J* = 8.3, 1.1 Hz, 1H), 7.06, (ddd, *J* = 8.0, 7.0, 1.0 Hz, 1H), 6.88 (ddd, *J* = 8.1, 7.2, 1.3 Hz, 1H), 6.06 (s, 1H), 3.77 (s, 3H). **^{13}C NMR (126 MHz, acetone-d$_6$+40 uL D_2O)** δ 153.2, 152.8, 137.9, 137.1, 131.3, 130.0, 128.8, 127.9, 127.8, 127.6, 127.2, 124.4, 121.8, 121.0, 119.6, 119.0, 118.2, 113.3, 109.7, 30.3. The resonance of the carbon bound to boron was not observed. **^{11}B NMR (160 MHz, acetone-d$_6$)** δ 27.5. **HRMS (LDI$^+$):** calculated for ($C_{23}H_{18}{}^{11}BNO_2$ $[M]^+$) = 351.1431; found 351.1434.

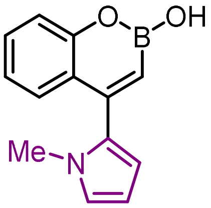

4-(1-Methylpyrrol-2-yl)-2-hydroxy-1,2-benzoxaborinine 5f

Following General Procedure 2, using 2-ethynylphenol **1a** (24.0 mg, 0.200 mmol), 2,6-di-*tert*-butyl-4-methylpyridine (123.0 mg, 0.600 mmol) and 1-methylpyrrole (44.0 µL, 0.500 mmol) in *o*-xylene at 140 °C gave title compound **5f** (66% spectroscopic yield). Purification by flash chromatography (CombiFlash Isco NextGen300+, 4 g SiO_2, CH_2Cl_2:MeOH, 100:0 to 90:10) gave the title compound **5f** as a pale brown oil (24.4 mg, 0.11 mmol, 54%).

^{1}H NMR (500 MHz, acetone-d$_6$+40 uL D$_2$O) δ 7.39 (ddd, J = 8.2, 7.5, 1.6 Hz, 1H), 7.33 (dd, J = 8.1, 1.6 Hz, 1H), 7.25, (dd, J = 8.2, 1.3 Hz, 1H), 7.08, (ddd, J = 8.1, 7.5, 1.3 Hz, 1H), 6.83 (app t, J = 2.8 Hz, 1H), 6.14 (dd, J = 3.4, 2.8 Hz, 1H), 6.11 (dd, J = 3.4, 1.6 Hz, 1H), 6.08 (s, 1H), 3.47 (s, 3H). **^{13}C NMR (126 MHz, acetone-d$_6$+40 uL D$_2$O)** δ 153.9, 151.6, 132.4, 130.3, 128.2, 125.2, 124.3, 122.6, 119.6, 110.3, 108.2, 34.7. The resonance of the carbon bound to boron was not observed. **^{11}B NMR (160 MHz, acetone-d$_6$)** δ 27.5. **HRMS (LDI$^+$):** calculated for ($C_{13}H_{13}{}^{11}BNO_2$ [M+H]$^+$) = 226.1034; found 226.1034.

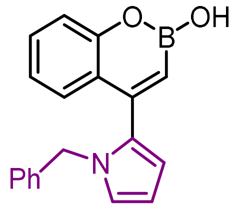

4-(1-Benzylpyrrol-2-yl)-2-hydroxy-1,2-benzoxaborinine 5g

Following General Procedure 2, using 2-ethynylphenol **1a** (24.0 mg, 0.200 mmol), 2,6-di-*tert*-butyl-4-methylpyridine (123.0 mg, 0.600 mmol) and 1-methylpyrrole (77.0 µL, 0.500 mmol) in *o*-xylene at 140 °C gave title compound **5g** (66% spectroscopic yield). Purification by flash chromatography (CombiFlash Isco NextGen300+, 4 g SiO$_2$, CH$_2$Cl$_2$:MeOH, 100:0 to 90:10) gave the title compound **5g** as a red oil (35.7 mg, 0.12 mmol, 59%).

^{1}H NMR (500 MHz, acetone-d$_6$+40 uL D$_2$O) δ 7.40-7.35 (m, 2H), 7.22 (dd, J = 8.4, 1.2 Hz, 1H), 7.19-7.11 (m, 3H), 7.07, (ddd, J = 8.4, 7.6, 1.2 Hz, 1H), 6.93-6.88 (m, 3H), 6.23 (dd, J = 3.5, 2.8 Hz, 1H), 6.18 (dd, J = 3.5, 1.6 Hz, 1H), 6.08 (s, 1H), 5.04 (s, 2H). **^{13}C NMR (126 MHz, acetone-d$_6$+40 uL D$_2$O)** δ 152.6, 150.3, 138.4, 131.3, 129.1, 127.8, 126.9, 126.7, 126.2, 124.0, 122.5, 121.3, 118.2, 109.7, 107.6, 50.1. The resonance of the carbon bound to boron was not observed. **^{11}B NMR (160 MHz, acetone-d$_6$)** δ 27.4. **HRMS (LDI$^+$):** calculated for ($C_{19}H_{17}{}^{11}BNO_2$ [M+H]$^+$) = 302.1347; found 302.1346. **HRMS (LDI$^+$):** calculated for ($C_{19}H_{16}{}^{11}BNO_2Na$ [M+Na]$^+$) = 324.1166; found 324.1166.

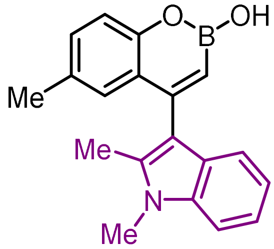

4-(1,2-Dimethyl-3-indolyl)-2-hydroxy-6-methyl-1,2-benzoxaborinine 5h

Following General Procedure 2, using 2-ethynyl-4-methylphenol **1b** (26.4 mg, 0.200 mmol), 2,6-di-*tert*-butyl-4-methylpyridine (123.0 mg, 0.600 mmol) and 1,2-dimethylindole (72.6 mg, 0.500 mmol) in *o*-xylene at 140 °C gave title compound **5h** (66% spectroscopic yield). Purification by flash chromatography (CombiFlash Isco NextGen300+, 4 g SiO$_2$, CH$_2$Cl$_2$:MeOH, 100:0 to 90:10) gave the title compound **5h** as a brown solid (23.6 mg, 0.08 mmol, 39%).

^{1}H NMR (500 MHz, acetone-d$_6$+40 uL D$_2$O) δ 7.41 (d, J = 8.2 Hz, 1H), 7.21-7.12 (m, 5H), 6.98 (ddd, J = 7.9, 7.2, 0.9 Hz, 1H), 6.10 (s, 1H), 3.80 (s, 3H), 2.35 (s, 3H), 2.15 (s, 3H). **^{13}C NMR (126 MHz, acetone-d$_6$+40 uL D$_2$O)** δ 154.5, 152.3, 137.6, 135.3, 131.3, 130.8, 128.8, 128.2, 125.2, 121.7, 120.1, 119.5, 119.3, 113.1, 109.9, 29.9, 20.8, 11.5. The resonance of the carbon bound to boron was not observed. **^{11}B NMR (160 MHz, acetone-d$_6$)** δ 27.8**. HRMS (LDI$^+$):** calculated for ($C_{19}H_{18}{}^{11}BNO_2$ [M]$^+$) = 303.1431; found 303.1427.

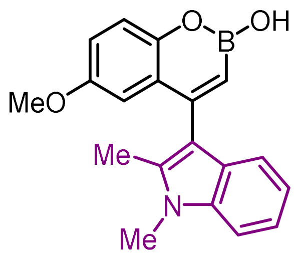

4-(1,2-Dimethyl-3-indolyl)-6-methoxy-2-hydroxy-1,2-benzoxaborinine 5i

Following General Procedure 2, using 4-methoxy-2-ethynylphenol **1j** (29.6 mg, 0.200 mmol), 2,6-di-*tert*-butyl-4-methylpyridine (123.0 mg, 0.600 mmol) and 1,2-dimethylindole (72.6 mg, 0.500 mmol) in *o*-xylene at 140 °C gave title compound **5i** (45% spectroscopic yield). Purification by flash chromatography (CombiFlash Isco NextGen300+, 4 g SiO_2, CH_2Cl_2:MeOH, 100:0 to 90:10) gave the title compound **5i** as a brown oil (27.9 mg, 0.09 mmol, 44%).

^{1}H NMR (500 MHz, acetone-d$_6$+40 uL D_2O) δ 7.41 (d, J = 8.1 Hz, 1H), 7.23-7.20 (m, 2H), 7.14 (ddd, J = 8.2, 7.2, 1.2 Hz, 1H), 7.00-6.97 (m, 2H), 6.87 (d, J = 3.1 Hz, 1H), 6.13 (s, 1H), 3.80 (s, 3H), 3.55 (s, 3H), 2.37 (s, 3H). **^{13}C NMR (126 MHz, acetone-d$_6$+40 uL D_2O)** δ 153.7, 153.0, 147.2, 136.4, 134.3, 126.7, 124.7, 122.0 (br), 120.5, 118.9, 118.4, 115.4, 111.7, 111.3, 108.7, 54.5, 28.7, 10.3. The resonance of the carbon bound to boron was not observed. **^{11}B NMR (160 MHz, acetone-d$_6$)** δ 27.8. **HRMS (LDI):** calculated for ($C_{19}H_{18}{}^{11}BNO_3$ [M]) = 319.1380; found 319.1377. **HRMS (LDI$^+$):** calculated for ($C_{19}H_{18}{}^{11}BNO_3Na$ [M+Na]$^+$) = 342.1272; found 342.1274.

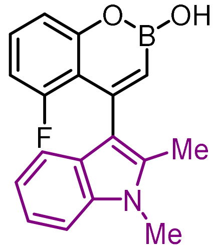

4-(1,2-Dimethyl-3-indolyl)-5-fluoro-2-hydroxy-1,2-benzoxaborinine 5j

Following General Procedure 2, using 3-fluoro-2-ethynylphenol **1g** (27.2 mg, 0.200 mmol), 2,6-di-*tert*-butyl-4-methylpyridine (123.0 mg, 0.600 mmol) and 1,2-dimethylindole (72.6 mg, 0.500 mmol) in *o*-xylene at 140 °C gave title compound **5j** (46% spectroscopic yield). Purification by flash chromatography (CombiFlash Isco NextGen300+, 4 g SiO_2, CH_2Cl_2:MeOH, 100:0 to 90:10) gave the title compound **5j** as a brown oil (23.3 mg, 0.08 mmol, 38%).

^{1}H NMR (500 MHz, acetone-d$_6$+40 uL D_2O) δ 7.37 (m, 2H), 7.20 (d, J = 7.9, Hz, 1H), 7.15 (app d, J = 8.2 Hz, 1H), 7.09 (ddd, J = 8.2, 7.1, 1.1 Hz, 1H), 6.94 (ddd, J = 7.9, 7.1, 1.0 Hz, 1H), 6.75 (ddd, J = 12.0, 8.1, 1.0 Hz, 1H), 6.11 (s, 1H), 3.75 (s, 3H), 2.33 (s, 3H). **^{13}C NMR (126 MHz, acetone-d$_6$+40 uL D_2O)** δ 159.4 (d, J = 254.0 Hz), 154.5 (d, J = 5.1 Hz), 152.7, 149.7, 136.1, 132.7 (d, J = 2.9 Hz), 129.0 (d, J = 11.4 Hz), 127.1 (d, J = 3.8 Hz), 120.2, 118.7, 117.7, 114.7 (d, J = 3.5 Hz), 108.9, 108.7, 108.5, 28.5, 9.8. The resonance of the carbon bound to boron was not observed. **^{11}B NMR (160 MHz, acetone-d$_6$)** δ 27.5. **^{19}F NMR (471 MHz, acetone-d$_6$)** δ -110.9 (dd, J = 12.0, 5.6 Hz). **HRMS (LDI$^+$):** calculated for ($C_{18}H_{15}{}^{11}BFNO_2Na$ [M+Na]) = 330.1072; found 330.1074.

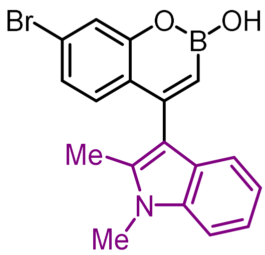

4-(1,2-Dimethyl-3-indolyl)-7-bromo-2-hydroxy-1,2-benzoxaborinine 5k
Following General Procedure 2, using 5-bromo-2-ethynylphenol **1k** (39.4 mg, 0.200 mmol), 2,6-di-*tert*-butyl-4-methylpyridine (123.0 mg, 0.600 mmol) and 1,2-dimethylindole (72.6 mg, 0.500 mmol) in *o*-xylene at 140 °C gave title compound **5k** (54% spectroscopic yield). Purification by flash chromatography (CombiFlash Isco NextGen300+, 4 g SiO_2, CH_2Cl_2:MeOH, 100:0 to 90:10) gave the title compound **5k** as a brown oil (30.1 mg, 0.08 mmol, 41%).

^{1}H NMR (500 MHz, acetone-d$_6$+40 uL D_2O) δ 7.48 (d, *J* = 2.0 Hz, 1H), 7.42 (d, *J* = 8.1 Hz, 1H), 7.21 (d, *J* = 8.5 Hz, 1H), 7.16-7.12 (m, 3H), 6.97 (ddd, *J* = 7.8, 7.2, 1.0 Hz, 1H), 6.15 (s, 1H), 3.79 (s, 3H), 2.34 (s, 3H). **^{13}C NMR (126 MHz, acetone-d$_6$+40 uL D_2O)** δ 153.3, 152.4, 136.2, 134.2, 128.9, 126.4, 123.9, 123.5, 121.2, 121.0, 120.4, 118.8, 117.9, 111.0, 108.5, 28.5, 9.9. The resonance of the carbon bound to boron was not observed. **^{11}B NMR (160 MHz, acetone-d$_6$)** δ 27.7. **HRMS (LDI$^+$):** calculated for ($C_{18}H_{16}{}^{11}BBrNO_2$ $[M+H]^+$) = 368.0452; found 368.0452.

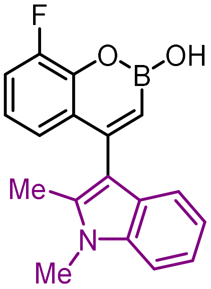

4-(1,2-Dimethyl-3-indolyl)-8-fluoro-2-hydroxy-1,2-benzoxaborinine 5l
Following General Procedure 2, using 6-fluoro-2-ethynylphenol **1l** (27.2 mg, 0.200 mmol), 2,6-di-*tert*-butyl-4-methylpyridine (123.0 mg, 0.600 mmol) and 1,2-dimethylindole (72.6 mg, 0.500 mmol) in *o*-xylene at 140 °C gave title compound **5l** (34% spectroscopic yield). Purification by flash chromatography (CombiFlash Isco NextGen300+, 4 g SiO_2, CH_2Cl_2:MeOH, 100:0 to 90:10) gave the title compound **5l** as a brown oil (20.2 mg, 0.07 mmol, 33%).

^{1}H NMR (500 MHz, acetone-d$_6$+40 uL D_2O) δ 7.42 (d, *J* = 8.3 Hz, 1H), 7.24 (ddd, *J* = 9.5, 8.1, 1.5 Hz, 1H), 7.18 (d, *J* = 7.2 Hz, 1H), 7.14 (ddd, *J* = 8.3, 7.0, 1.2 Hz, 1H), 7.11 (dt, *J* = 8.1, 1.3 Hz, 1H), 6.99-6.94 (m, 2H), 6.19 (s, 1H), 3.80 (s, 3H), 2.36 (s, 3H). **^{13}C NMR (126 MHz, acetone-d$_6$+40 uL D_2O)** δ 153.3 (d, *J* = 3.8 Hz), 152.1 (d, *J* = 245.2 Hz), 141.5 (d, *J* = 11.2 Hz), 136.7, 134.7, 127.1, 127.0 (d, *J* = 1.0 Hz), 123.2 (d, *J* = 2.9 Hz), 120.9, 120.8 (d, *J* = 7.7 Hz) 119.4, 118.5, 115.2 (d, *J* = 18.5 Hz), 111.9, 109.1, 29.0, 10.5. The resonance of the carbon bound to boron was not observed. **^{11}B NMR (160 MHz, acetone-d$_6$)** δ 27.7. **^{19}F NMR (471 MHz, acetone-d$_6$)** δ -137.6 (dd, *J* = 11.0, 5.2 Hz). **HRMS (LDI):** calculated for ($C_{18}H_{15}{}^{11}BFNO_2$ [M-H]) = 306.1102; found 306.1098.

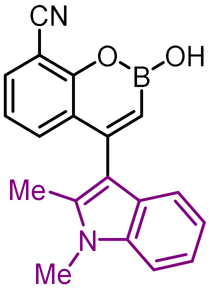

4-(1,2-Dimethyl-3-indolyl)-8-cyano-2-hydroxy-1,2-benzoxaborinine 5m

Following General Procedure 2, using 2-hydroxy-3-ethynylbenzonitrile **1i** (28.6 mg, 0.200 mmol), 2,6-di-*tert*-butyl-4-methylpyridine (123.0 mg, 0.600 mmol) and 1,2-dimethylindole (72.6 mg, 0.500 mmol) in *o*-xylene at 140 °C gave title compound **5m** (20% spectroscopic yield). Purification by flash chromatography (CombiFlash Isco NextGen300+, 4 g SiO_2, CH_2Cl_2:MeOH, 100:0 to 90:10) gave the title compound **5m** as a yellow oil (10.1 mg, 0.03 mmol, 16%).

^{1}H NMR (500 MHz, acetone-d$_6$+40 uL D_2O) δ 7.78 (dd, J = 7.6, 1.6 Hz, 1H), 7.58 (dd, J = 8.1, 1.6 Hz, 1H), 7.43 (d, J = 8.3 Hz, 1H), 7.19-7.13 (m, 3H), 6.98 (ddd, J = 8.1, 7.1, 1.0 Hz, 1H), 6.25 (s, 1H), 3.80 (s, 3H), 2.36 (s, 3H). **^{13}C NMR (126 MHz, acetone-d$_6$+40 uL D_2O)** δ δ 153.9, 152.0, 136.0, 134.4, 132.3, 132.1, 126.2, 124.8, 121.2, 120.3, 118.8, 117.7, 115.2, 110.3, 108.5, 102.2, 28.4, 9.7. The resonance of the carbon bound to boron was not observed. **^{11}B NMR (160 MHz, acetone-d$_6$)** δ 27.8, 0.16. **^{19}F NMR (471 MHz, acetone-d$_6$)** δ -151.5. **HRMS (LDI$^+$):** calculated for ($C_{19}H_{15}{}^{11}BN_2O_2$ $[M]^+$) = 314.1226; found 314.1223. **HRMS (LDI$^+$):** calculated for ($C_{19}H_{15}{}^{11}BN_2O_2Na$ $[M+Na]^+$) = 337.1119; found 337.1120. **HRMS (LDI$^+$):** calculated for ($C_{19}H_{15}{}^{11}BN_2O_2K$ $[M+K]^+$) = 353.0858; found 353.0860.

S2.2.5 General Procedure 3: Hydroamination of *o*-Alkynyl Phenols with Amines

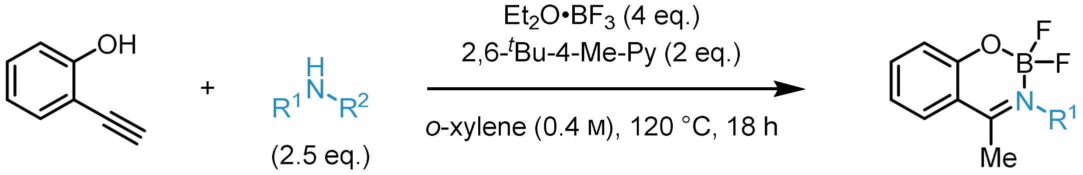

1a **6**

Same as General Procedure 1b, using 2-ethynylphenol **1a** (0.200 mmol), $Et_2O·BF_3$ (100 µL, 0.800 mmol), 2,6-di-*tert*-butyl-4-methylpyridine (82.0 mg, 0.400 mmol), then:
GP 3a: *N*-boc amine (0.500 mmol), which undergoes deprotection in-situ with $Et_2O·BF_3$.[2]
GP 3b: ethanol amine (0.500 mmol)
in *o*-xylene (0.500 mL) at 120 °C for 18 h. The yield was determined by ^{1}H NMR spectroscopy using nitromethane as an internal standard. The crude residue was purified via flash column chromatography on silica gel to obtain the pure boranil **6** (CombiFlash Isco NextGen300+, 4 g SiO_2, CH_2Cl_2:MeOH, 100:0 to 90:10).

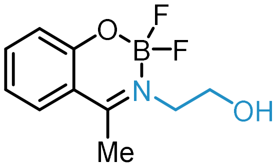

[2]E. F. Evans, N. J. Lewis, I. Kapfer, G. McDonald and R. J. K. Taylor, *Synthetic Communications*, 1997, **27**, 1819-1825.

2,2-Difluoro-3-(hydroxyethyl)-4-methyl-2*H*-benzo[e][1,3,2]oxazaborinin-3-ium-2-uide 6a

Following General Procedure 3a, using 2-ethynylphenol **1a** (24.0 mg, 0.200 mmol), 2,6-di-*tert*-butyl-4-methylpyridine (82.0 mg, 0.400 mmol) and *N*-boc-ethanolamine (77.0 µL, 0.500 mmol) in *o*-xylene at 120 °C gave title compound **6a** (42% spectroscopic yield). Purification by flash chromatography (CombiFlash Isco NextGen300+, 4 g SiO_2, CH_2Cl_2:MeOH, 100:0 to 90:10) gave the title compound **6a** as a pale blue oil (12.1 mg, 0.05 mmol, 27%).

^{1}H NMR (500 MHz, $CDCl_3$) δ 7.65 (ddd, *J* = 8.2, 1.6 Hz, 1H), 7.55, (ddd, *J* = 8.5, 7.2, 1.6 Hz, 1H), 7.11 (dd, *J* = 8.5, 1.3 Hz, 1H), 6.99 (ddd, *J* = 8.2, 7.2 1.3 Hz, 1H), 4.08 (br s, 2H), 4.07 (br s, 2H), 2.72 (s, 3H). **^{13}C NMR (126 MHz, $CDCl_3$)** δ 174.4, 158.2, 137.3, 128.9, 120.5, 120.4, 120.1, 61.6, 50.2, 17.0. **^{11}B NMR (160 MHz, $CDCl_3$)** δ 0.3. **^{19}F NMR (471 MHz, $CDCl_3$)** δ -142.3 (q, *J* = 16.4 Hz). **HRMS (ESI$^+$):** calculated for ($C_{10}H_{12}{}^{11}BF_2NO_2$ $[M]^+$) = 227.0924; found 227.0917.

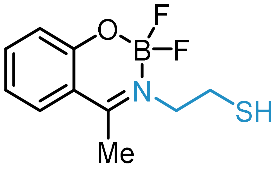

2,2-Difluoro-3-(sulfanylethyl)-4-methyl-2*H*-benzo[e][1,3,2]oxazaborinin-3-ium-2-uide 6b

Following General Procedure 3a, using 2-ethynylphenol **1a** (24.0 mg, 0.200 mmol), 2,6-di-*tert*-butyl-4-methylpyridine (82.0 mg, 0.400 mmol) and 2-(boc-amino)ethanethiol (84.0 µL, 0.500 mmol) in *o*-xylene at 120 °C gave title compound **6b** (76% spectroscopic yield). Purification by flash chromatography (CombiFlash Isco NextGen300+, 4 g SiO_2, CH_2Cl_2:MeOH, 100:0 to 90:10) gave the title compound **6b** as a pale blue oil (20.6 mg, 0.08 mmol, 42%).

Following General Procedure 3b, using 2-ethynylphenol **1a** (24.0 mg, 0.200 mmol), 2,6-di-*tert*-butyl-4-methylpyridine (82.0 mg, 0.400 mmol) and cysteamine (40.0 µL, 0.500 mmol) in *o*-xylene at 120 °C gave title compound **6b** (28% spectroscopic yield).

^{1}H NMR (500 MHz, acetone-d_6) δ 7.92 (dd, *J* = 8.2, 1.6 Hz, 1H), 7.62, (ddd, *J* = 8.4, 7.2, 1.6 Hz, 1H), 7.04 (ddd, *J* = 8.2, 7.2, 1.1 Hz, 1H), 7.01 (dd, *J* = 8.4, 1.1 Hz, 1H), 4.08 (br t, *J* = 8.7 Hz, 2H), 2.96 (app q, *J* = 8.7 Hz, 2H), 2.89 (s, 3H). **^{13}C NMR (126 MHz, acetone-d_6)** δ 175.5, 159.0, 138.0, 130.8, 120.6, 120.4, 118.4, 51.9, 23.9, 17.0. **^{11}B NMR (160 MHz, acetone-d_6)** δ 0.3. **^{19}F NMR (471 MHz, acetone-d_6)** δ -142.0 (q, *J* = 17.4 Hz). **HRMS (ESI$^+$):** calculated for ($C_{10}H_{12}{}^{11}BF_2NOS$ $[M]^+$) = 243.0695; found 243.0696.

S2.3 Derivatisation of Organoborane Products

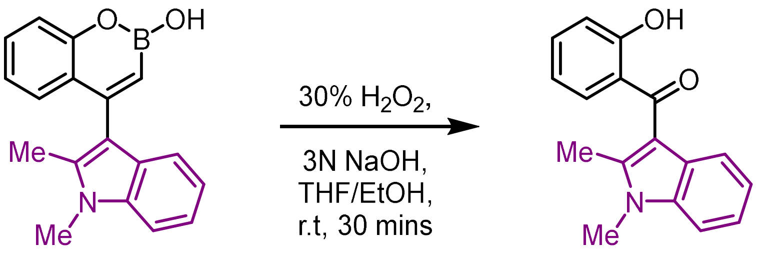

5d, (0.2 mmol) **7**

To a stirred solution of benzoxaborinine **5d** (57.8 mg, 0.200 mmol) in THF (2 mL)/EtOH (0.5 mL) solvent system, was added 30% H_2O_2 (1 mL) and NaOH (3N, 1 mL) drop-wise and

sequentially. The reaction was stirred under air for 30 mins. Volatiles were removed under reduced pressure before $Na_2S_2O_3$ (20 mL) was added with stirring. The aqueous layer was separated and extracted with DCM (3 x 10 mL). The combined organic layers were dried over $MgSO_4$, filtered and concentrated *in vacuo*. Purification by flash chromatography (CombiFlash Isco NextGen300+, 4 g SiO_2, PE:EtOAc, 100:0 to 0:100) gave product **7** as a yellow oil (24.4 mg, 0.09 mmol, 46%). Reaction proceeds via a coumaranone intermediate.[3]

^{1}H NMR (500 MHz, $CDCl_3$) δ 12.29 (s, 1H), 7.72 (dd, *J* = 8.0, 1.6 Hz, 1H), 7.50 (d, *J* = 8.1 Hz, 1H), 7.47 (ddd, *J* = 8.6, 7.4, 1.6 Hz, 1H), 7.34 (d, *J* = 8.1 Hz, 1H), 7.25 (app t, *J* = 8.1 Hz, 1H), 7.14 (app t, *J* = 7.4 Hz, 1H), 7.07, (app d, *J* = 8.6 Hz, 1H), 6.82 (app t, *J* = 8.0 Hz, 1H), 3.76 (s, 3H), 2.59 (s, 3H). **^{13}C NMR (126 MHz, $CDCl_3$)** δ 196.8, 162.4, 143.3, 136.7, 135.5, 133.6, 126.9, 122.3, 121.6, 121.5, 120.8, 118.5, 118.0, 113.4, 109.4, 29.9, 12.6. **HRMS (LDI$^+$):** calculated for ($C_{17}H_{15}NO_2Na$ $[M+Na]^+$) = 288.0995; found 288.0999.

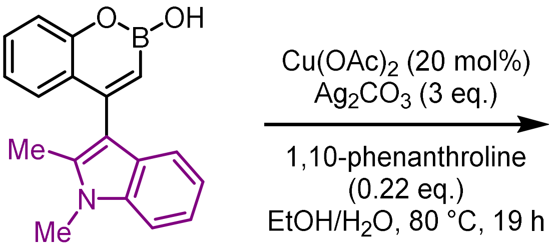

5d, (0.2 mmol) **8**

According to literature procedure,[4] benzoxaborinine **5d** (57.8 mg, 0.200 mmol), $Cu(OAc)_2$ (8.00 mg, 0.040 mmol), Ag_2CO_3 (165 mg, 0.600 mmol) and 1,10-phenanthroline (8.70 mg, 0.044 mmol) was dissolved in EtOH (2 mL) and water (0.1 mL). The reaction was sealed under air and stirred at 80 °C for 19 h. The crude was loaded directly onto celite. Purification by flash chromatography (CombiFlash Isco NextGen300+, 4 g SiO_2, PE:EtOAc, 100:0 to 0:100) gave product **8** as a pink oil (20.1 mg, 0.08 mmol, 38%).

^{1}H NMR (500 MHz, $CDCl_3$) δ 7.69 (s, 1H), 7.60 (d, *J* = 8.2 Hz, 1H), 7.54 (d, *J* = 7.9 Hz, 2H), 7.37 (app t, *J* = 8.2 Hz, 2H), 7.27-7.24 (m, 2H), 7.12, (app t, *J* = 7.4 Hz, 1H), 3.79 (s, 3H), 2.48 (s, 3H). **^{13}C NMR (126 MHz, $CDCl_3$)** δ 155.6, 142.3, 137.0, 134.7, 128.6, 127.7, 124.3, 122.6, 121.4, 121.3, 119.7, 119.3, 115.4, 111.7, 108.9, 102.8, 30.0, 11.5. **HRMS (LDI$^+$):** calculated for ($C_{18}H_{15}NO$ $[M]^+$) = 261.1154; found 261.1149.

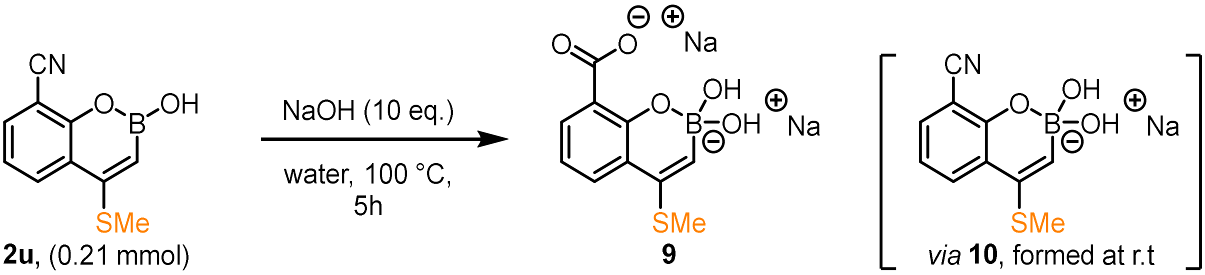

2u, (0.21 mmol) **9**

To a J Youngs ampule, was added **2u** (45.0 mg, 0.21 mmol), NaOH (83.0 mg, 2.10 mmol, 10 eq.) and water (1.0 mL). The reaction was heated at 100 °C for 5 hours. Acetone was then added drop-wise to the reaction until solid crashed out, which was filtered and washed with acetone (3 x 2 mL). The solid was then dissolved in MeOH and solid impurities were filtered off. Removal of volatiles *in vacuo* gave **9** as a pale-brown solid (61.8 mg, 0.21 mmol, quant.).

---

[3]Z. Tongm Z. Tang, C.-T. Au and R. Qiu, *J. Org. Chem.* 2020, **85**, 8533−8543.
[4]Y. Sumida, R. Harada, T. Sumida, K. Johmoto, H. Uekusa and T. Hosoya, *Org. Lett.,* 2020, **22**, 6687-6691.

*Disodium salt **9**:* **^{1}H NMR (500 MHz, D_2O)** δ 7.51 (d, *J* = 7.7 Hz, 1H), 7.23 (d, *J* = 7.7 Hz, 1H), 6.81 (t, *J* = 7.7 Hz, 1H), 5.90 (s, 1H), 2.40 (s, 3H). **^{13}C NMR (126 MHz, D_2O)** δ 178.5, 152.0, 134.0, 129.0, 126.3, 124.2, 124.1, 116.3, 13.1. The resonance of the carbon bound to boron was not observed. **^{11}B NMR (160 MHz, D_2O)** δ 1.57. **HRMS (ESI$^+$):** calculated for ($C_{10}H_8{}^{11}BNa_2O_4S$ $[M-H_2O+H]^+$) = 281.0026; found 281.0026.

*Monosodium salt **10**:* **^{1}H NMR (500 MHz, D_2O)** δ 7.73 (dd, *J* = 7.8, 1.5 Hz, 1H), 7.48 (dd, *J* = 7.8, 1,5 Hz, 1H), 6.81 (t, *J* = 7.8 Hz, 1H), 5.98 (s, 1H), 2.40 (s, 3H). **^{13}C NMR (126 MHz, D_2O)** δ 159.1, 133.1, 131.2, 128.9, 124.9, 119.8, 117.4, 99.6, 13.1. The resonance of the carbon bound to boron was not observed. **^{11}B NMR (160 MHz, D_2O)** δ 1.63.

S3. Mechanistic Investigation

in-situ monitoring

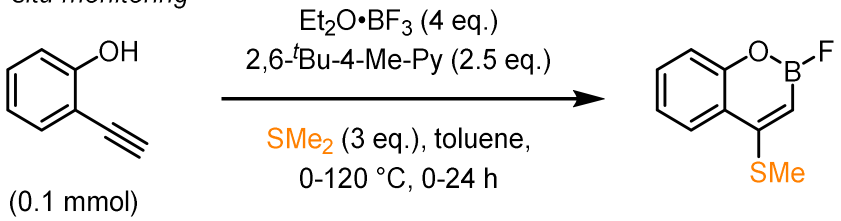

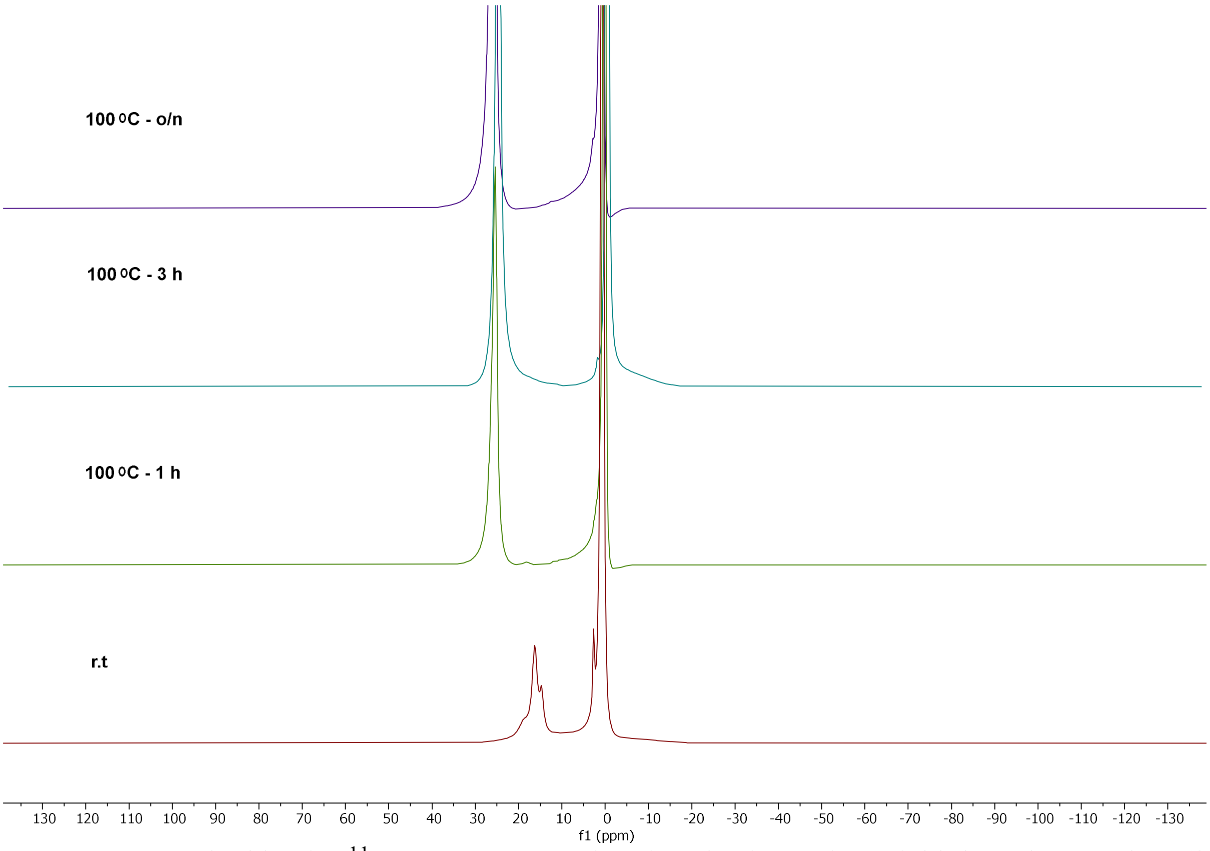

Figure S1. Stacked in situ ^{11}B NMR spectra showing the formation of thioboration product (δ = 26 ppm) over time.

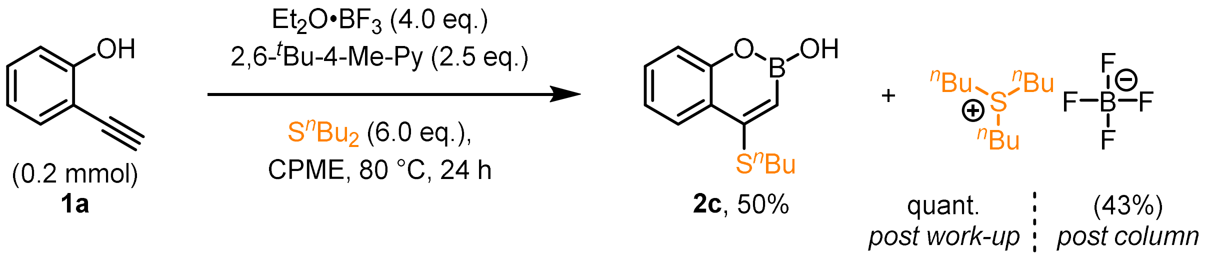

Following General Procedure 1a, using 2-ethynylphenol **1a** (24.0 mg, 0.200 mmol), 2,6-di-*tert*-butyl-4-methylpyridine (103.0 mg, 0.500 mmol), dibutyl sulfide (1.20 mmol, 209 µL) in CPME at 80 °C gave benzoxaborinine **2c** (50% spectroscopic yield) and tri-*n*-butylsulfonoium tetrafluoroborate (50% spectroscopic yield). Purification by column chromatography (eluent = 10% MeOH/DCM) gave tri-*n*-butylsulfonoium tetrafluoroborate as a brown oil (48.5 mg, 0.17 mmol, 43%).

^{1}H NMR (500 MHz, $CDCl_3$) δ 3.33 (t, *J* = 7.6 Hz, 6H), 1.76 (quin., *J* = 7.6 Hz, 6H), 1.51 (sext., *J* = 7.6 Hz, 6H), 0.97 (t, *J* = 7.6 Hz, 9H). **^{13}C NMR (126 MHz, $CDCl_3$)** δ 39.9, 26.7, 21.8, 13.4. **^{11}B NMR (160 MHz, acetone-d_6)** δ -1.05. **^{19}F NMR (471 MHz, acetone-d_6)** δ -150.6 (q, *J* = 1.2 Hz). **HRMS (ESI$^+$):** calculated for ($C_{24}H_{54}{}^{11}BF_4S_2$ [M+($S^n Bu_3$)]$^+$) = 493.3691; found 493.3723.[5] **HRMS (ESI$^+$):** calculated for ($C_{12}H_{27}S$ [M-BF_4]$^+$) = 203.1828; found 203.1850.

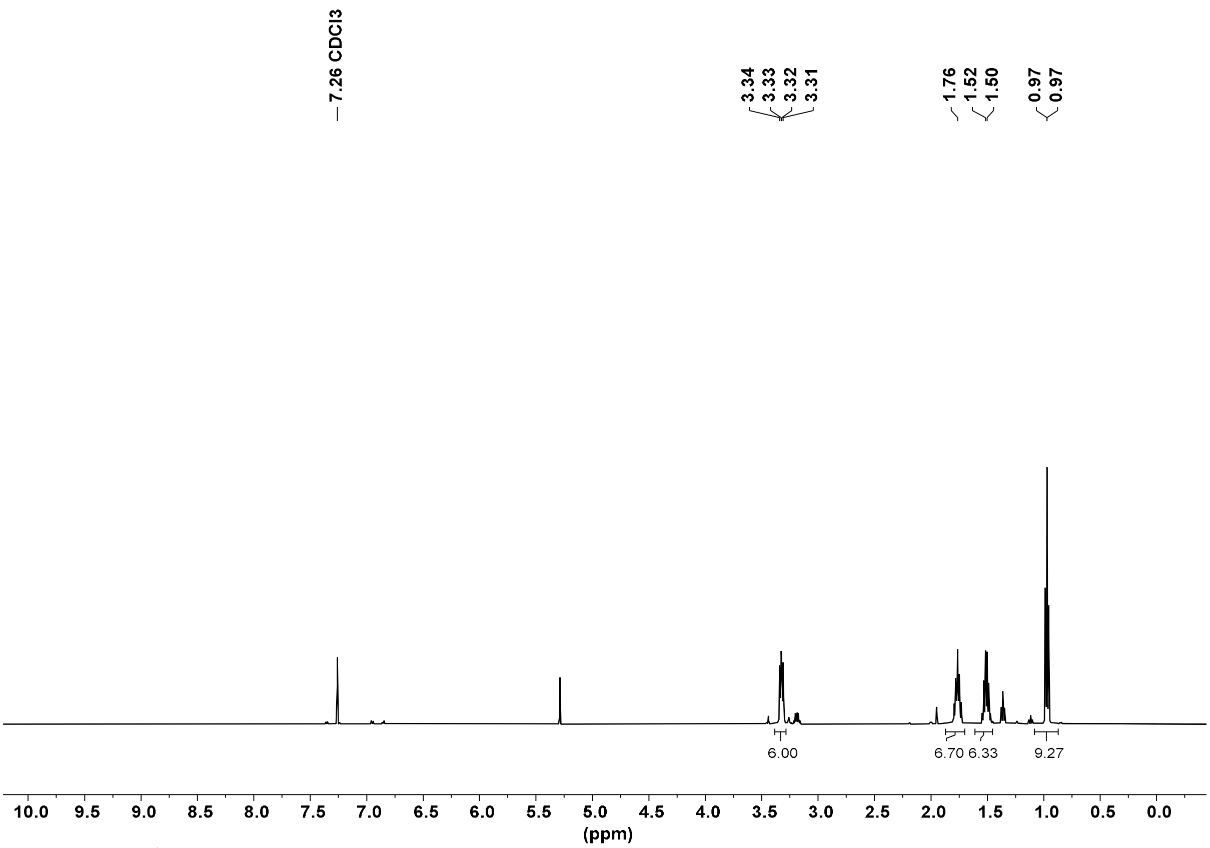

Figure S2. ^{1}H NMR spectra of tri-*n*-butylsulfonium BF_4 salt

[5]M. J. Van Stipdonk, M. P. Ince, B. A. Perera and J. A. Martin, *Rapid Commun. Mass Spectrom.*, 2002, **16**, 355-363.

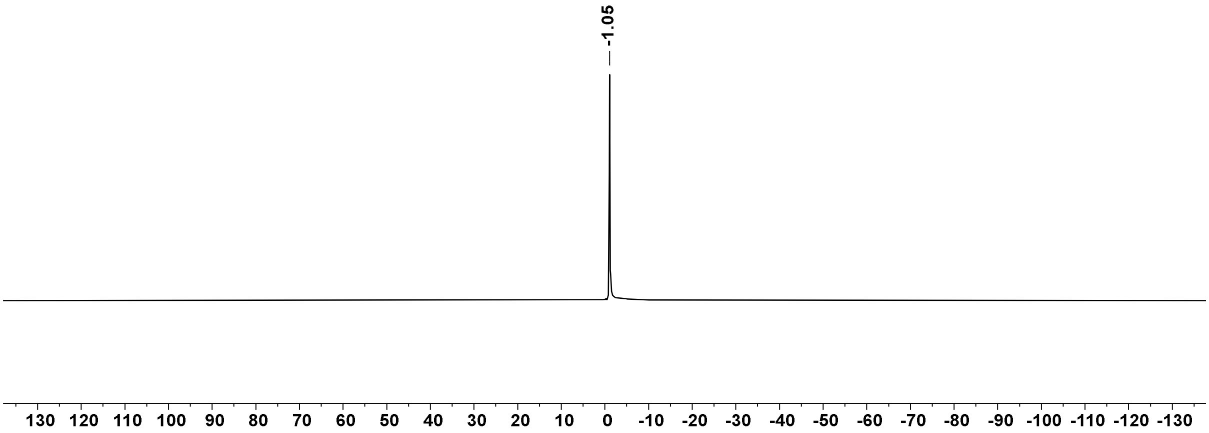

Figure S3. ^{11}B NMR spectra of tri-*n*-butylsulfonium BF_4 salt

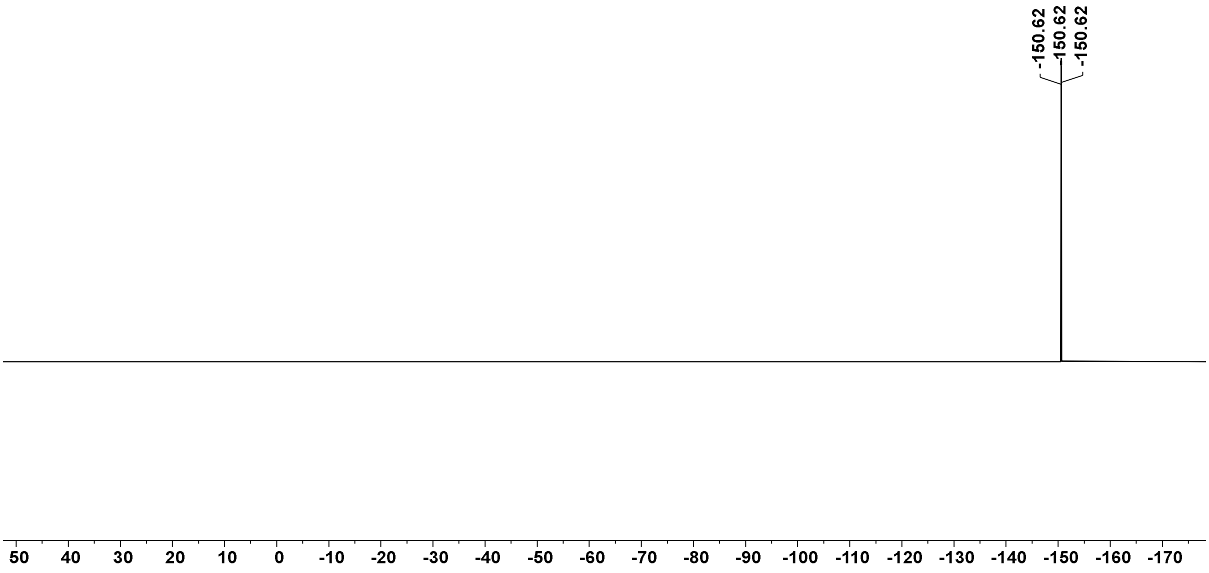

Figure S4. ^{19}F NMR spectra of tri-*n*-butylsulfonium BF_4 salt

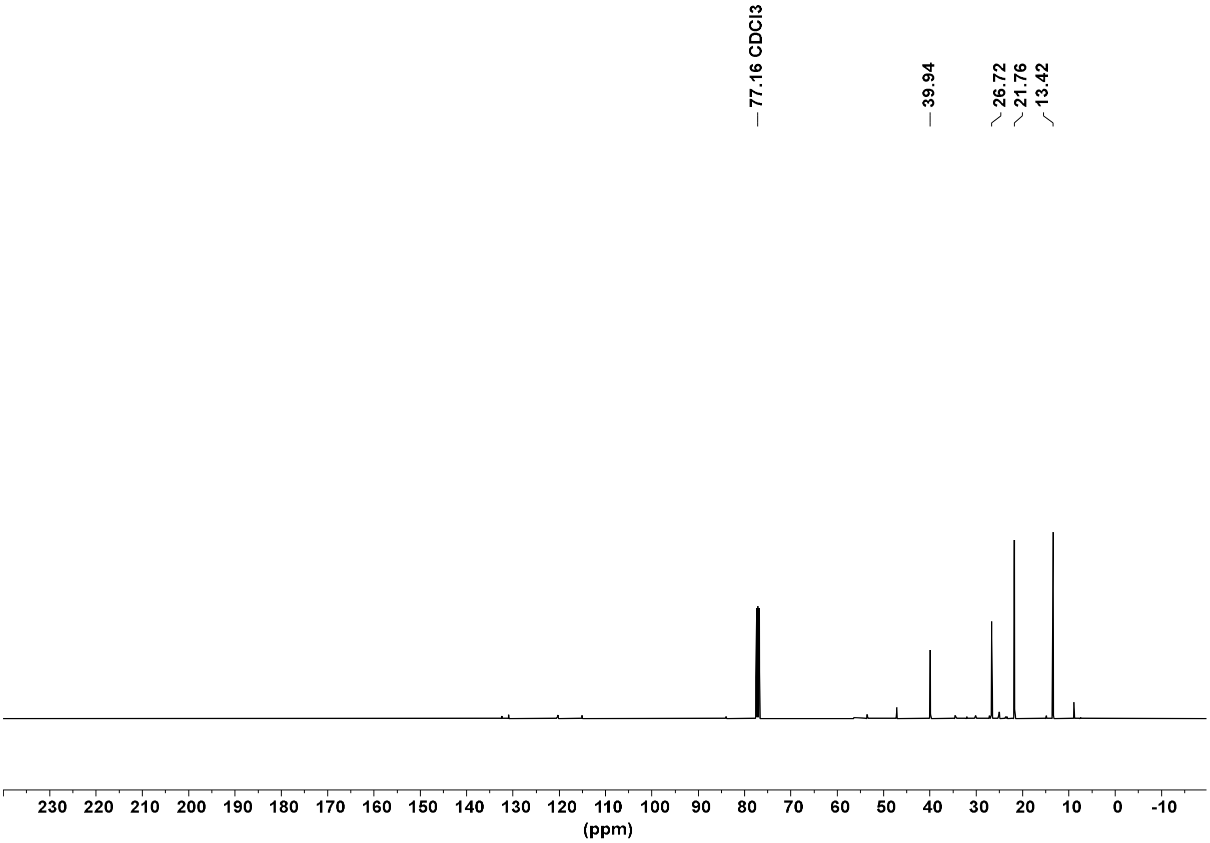

Figure S5. ^{13}C NMR spectra of tri-*n*-butylsulfonium BF_4 salt

Scheme S1. Thioboration of phenyl acetylene

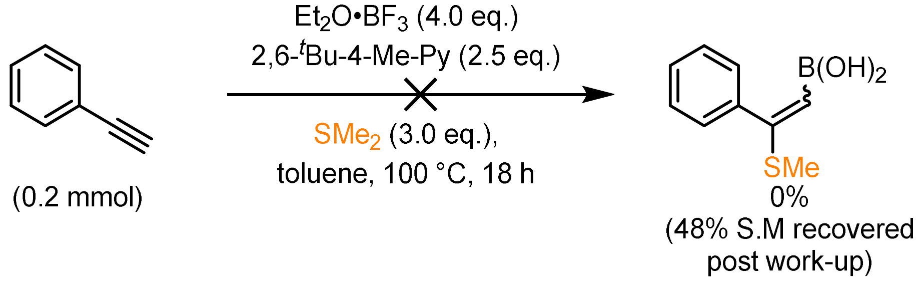

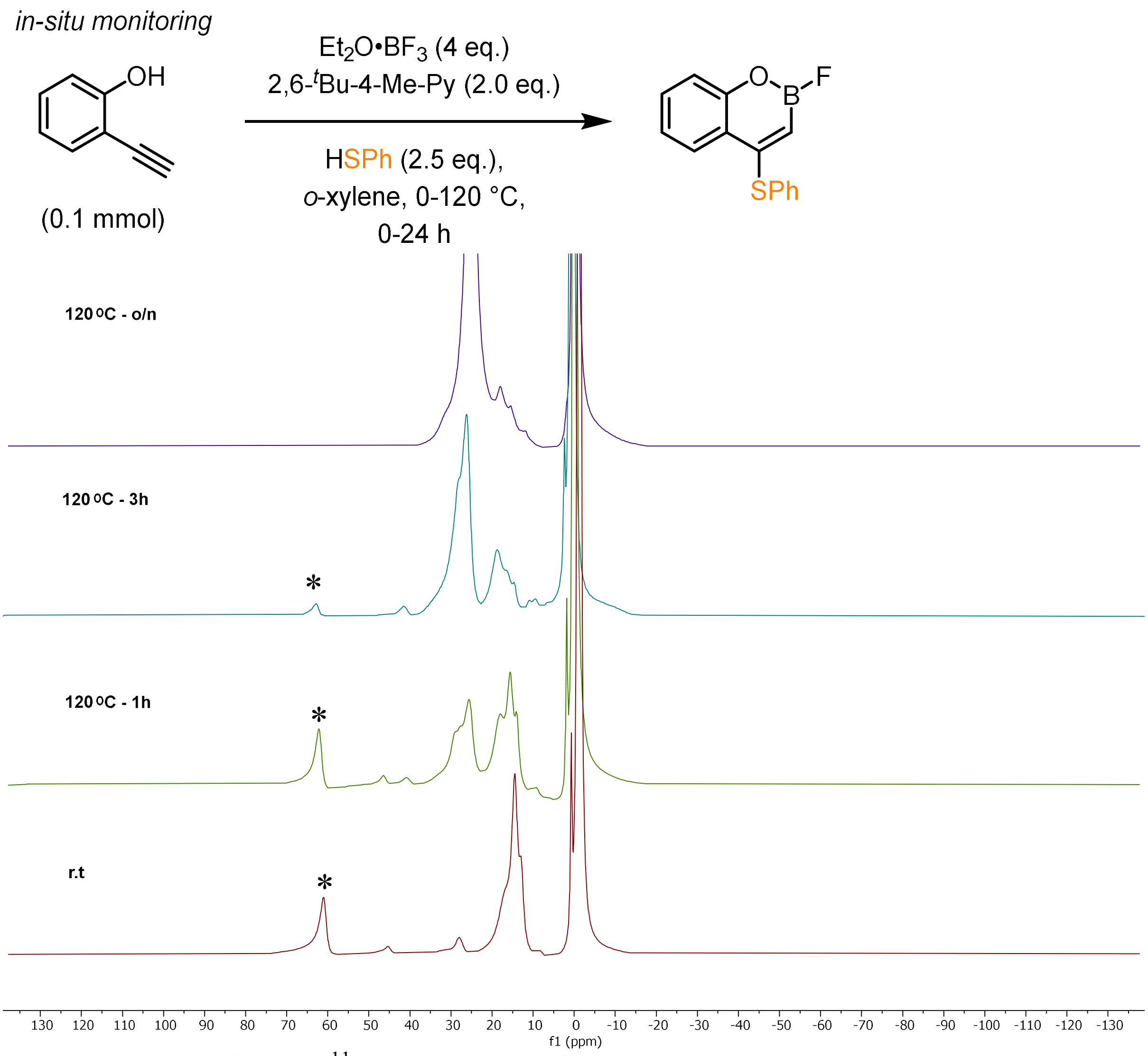

Figure S6. Stacked in situ ^{11}B NMR spectra showing the presence of $B(SPh)_3$ (asterisked) alongside the formation of thioboration product over time.

Synthesis of trithiophenylborane

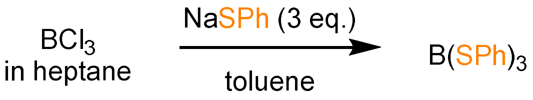

According to a modified literature procedure,[6] to an oven dried J Youngs ampoule, was added NaSPh (0.896 g, 6.78 mmol) and toluene (4 mL). BCl_3 in *n*-heptane (2.3 mL, 1.0 M, 2.3 mmol) was added dropwise. The resulting suspension heated at 60 °C for 1.5 h. The reaction mixture was then filtered, washed by toluene, and evaporated under reduced pressure. The solid was washed by hexane to give the target compound as a white solid (265.0 mg, 35%).

^{1}H NMR (500 MHz, $CDCl_3$) δ 7.49-7.46 (m, 6H), 7.35-7.33 (m, 9H). **^{11}B NMR (160 MHz, $CDCl_3$)** δ 62.5. **^{13}C NMR (126 MHz, $CDCl_3$)** δ 134.8, 129.9, 128.9, 128.3. This data is in agreement with the literature.[7]

---

[6] T. Matsuyama, H. Ishida, C. Wang, K. Miyamoto, M. Nakajima, N. Toriumi, Y. Nagashima and M. Uchiyama, *JACS Au,* 2024, **4**, 4927-4933

[7] See ref 6

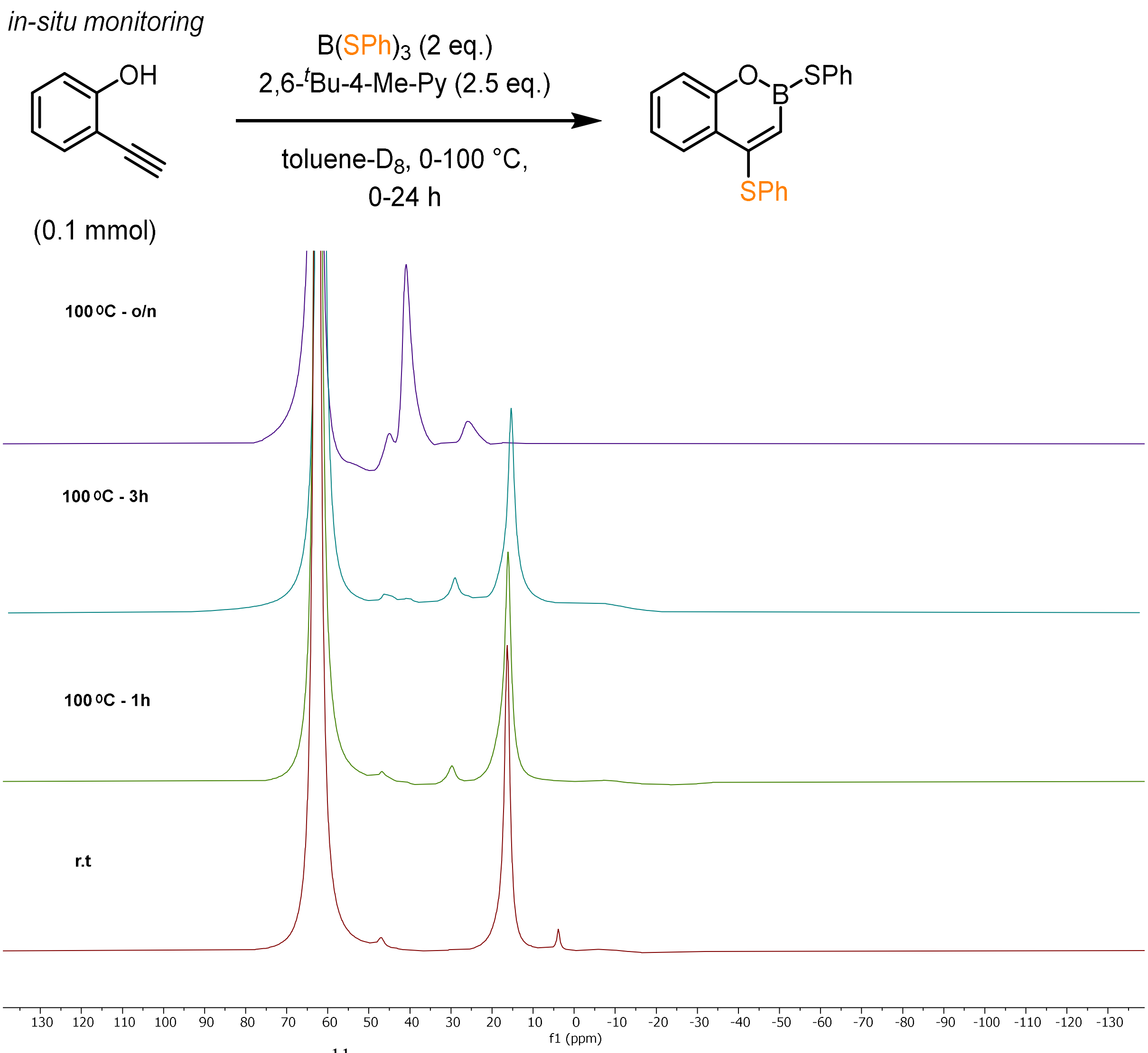

Figure S7. Stacked in situ ^{11}B NMR spectra showing the formation of thioboration product (assigned as $\delta = 41$ ppm) over time.

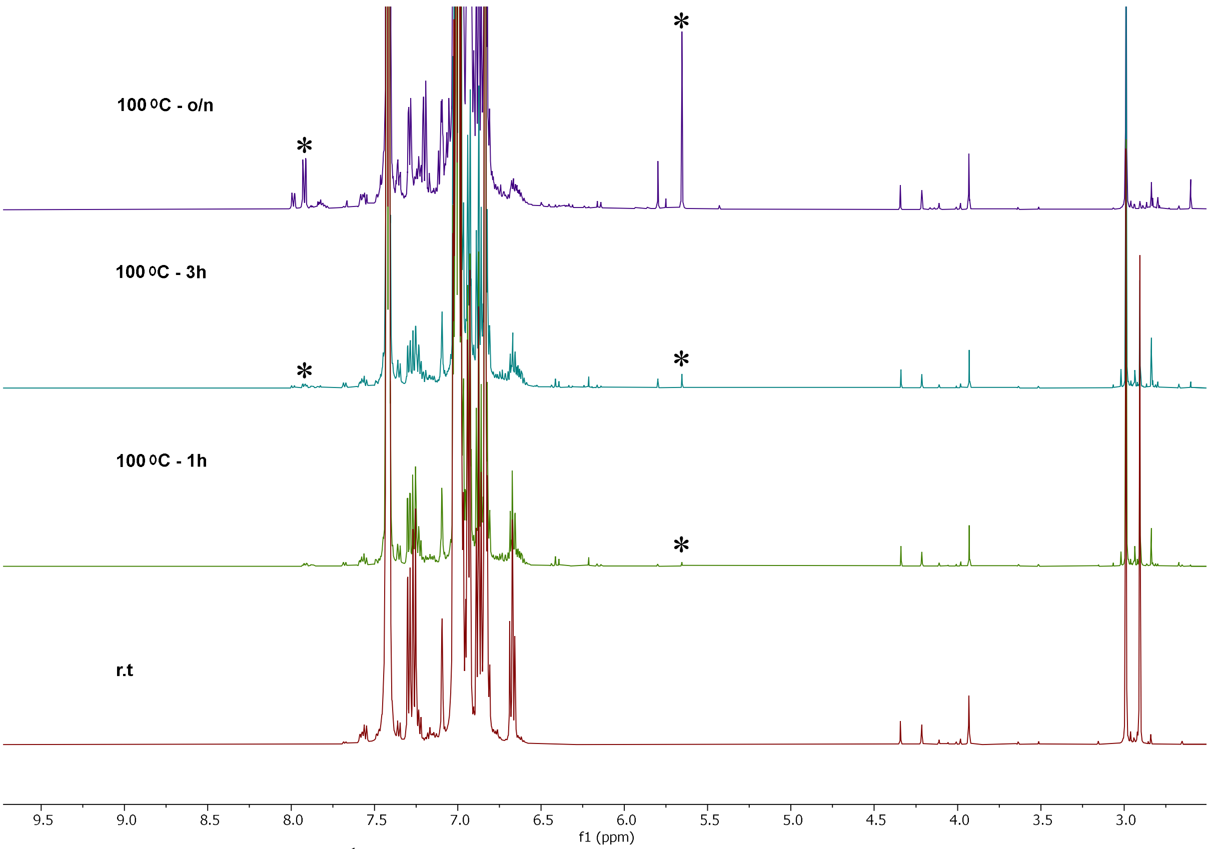

Figure S8. Stacked in situ ^{1}H NMR spectra showing the formation of thioboration product (asterisked) over time.

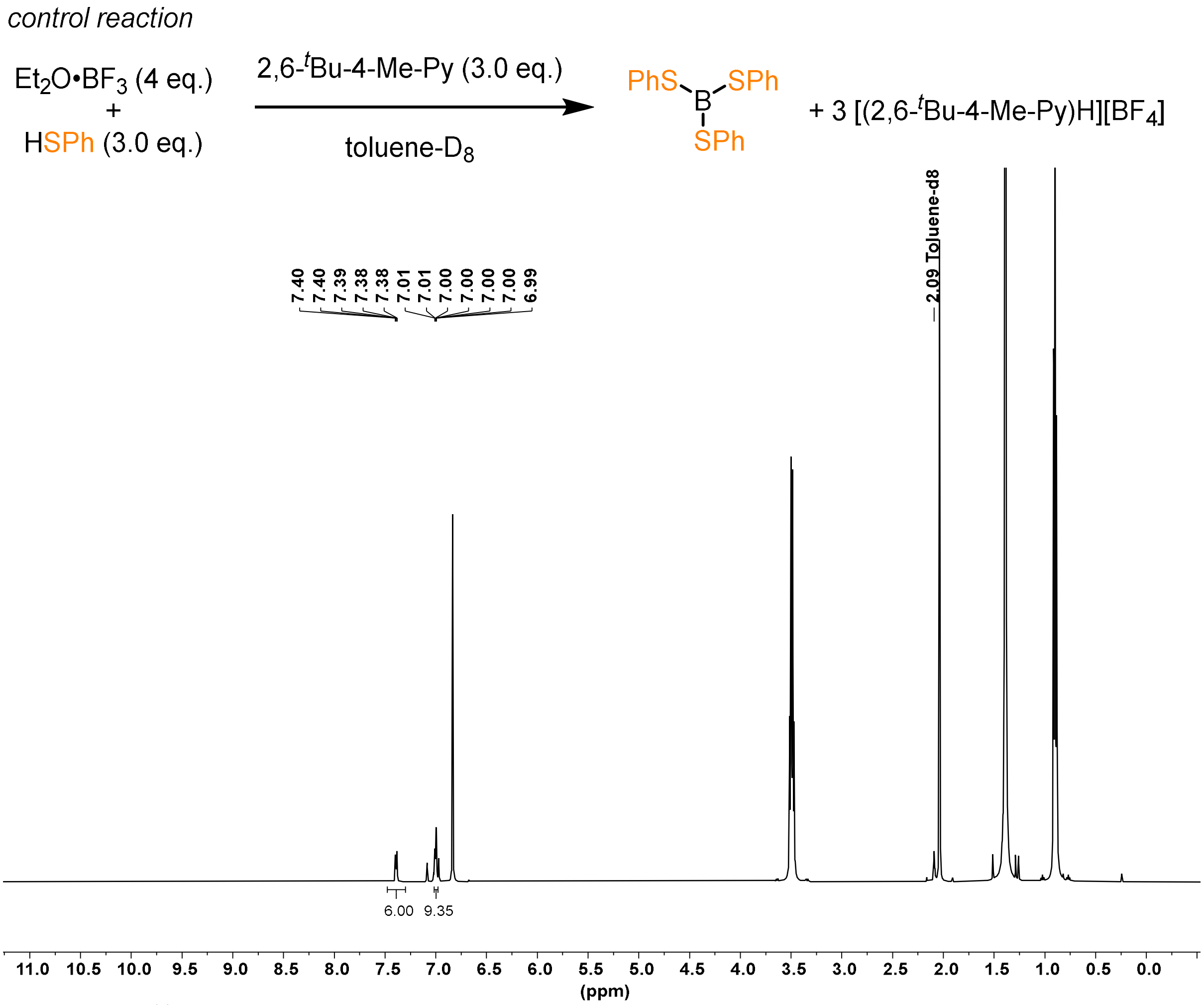

Figure S9. ^{11}H NMR spectrum showing the in-situ formation of $B(SPh)_3$.

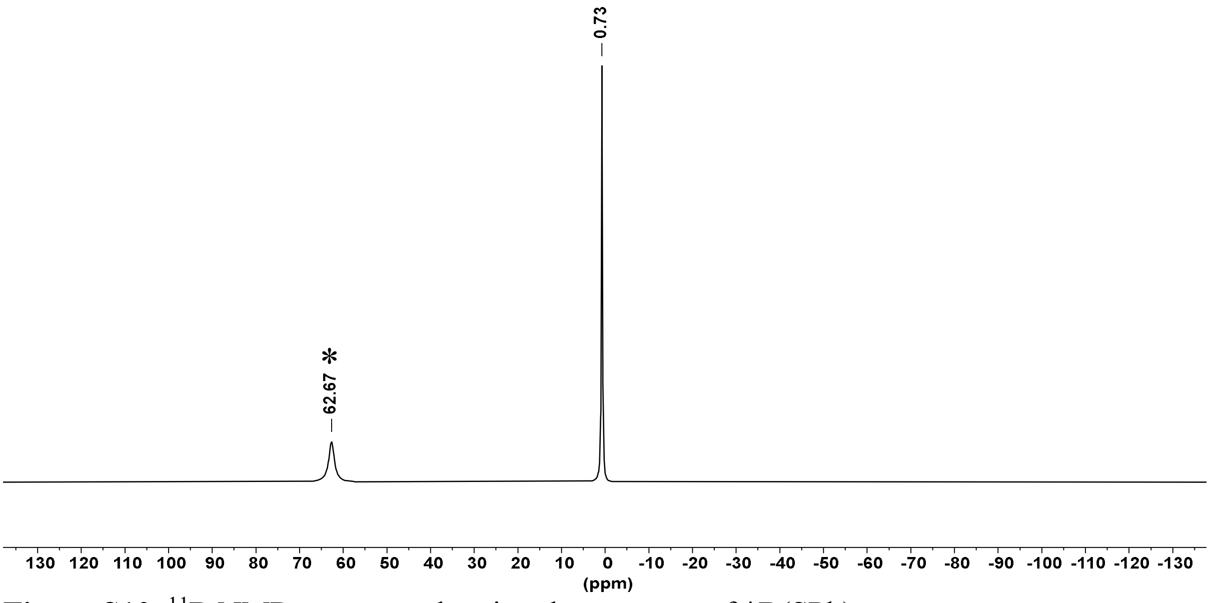

Figure S10. ^{11}B NMR spectrum showing the presence of *$B(SPh)_3$.

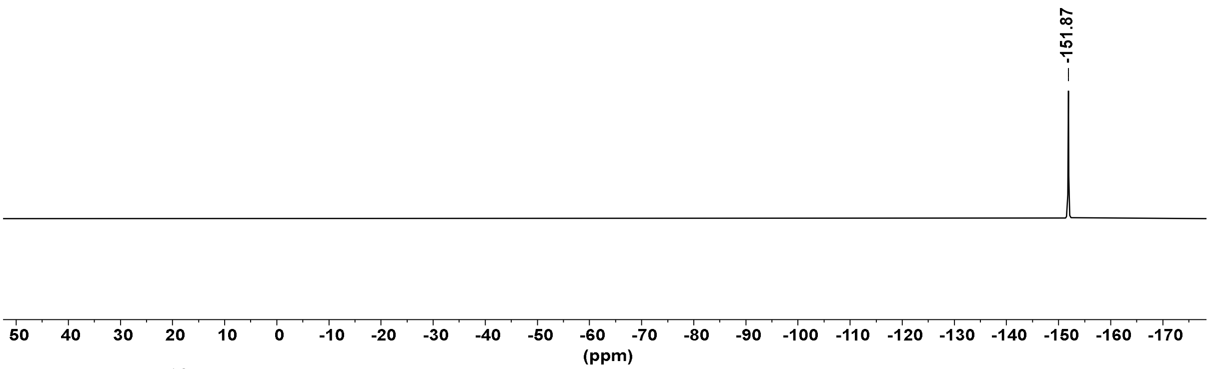

Figure S11. ^{19}F NMR spectrum.

control reaction

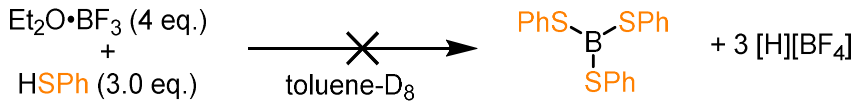

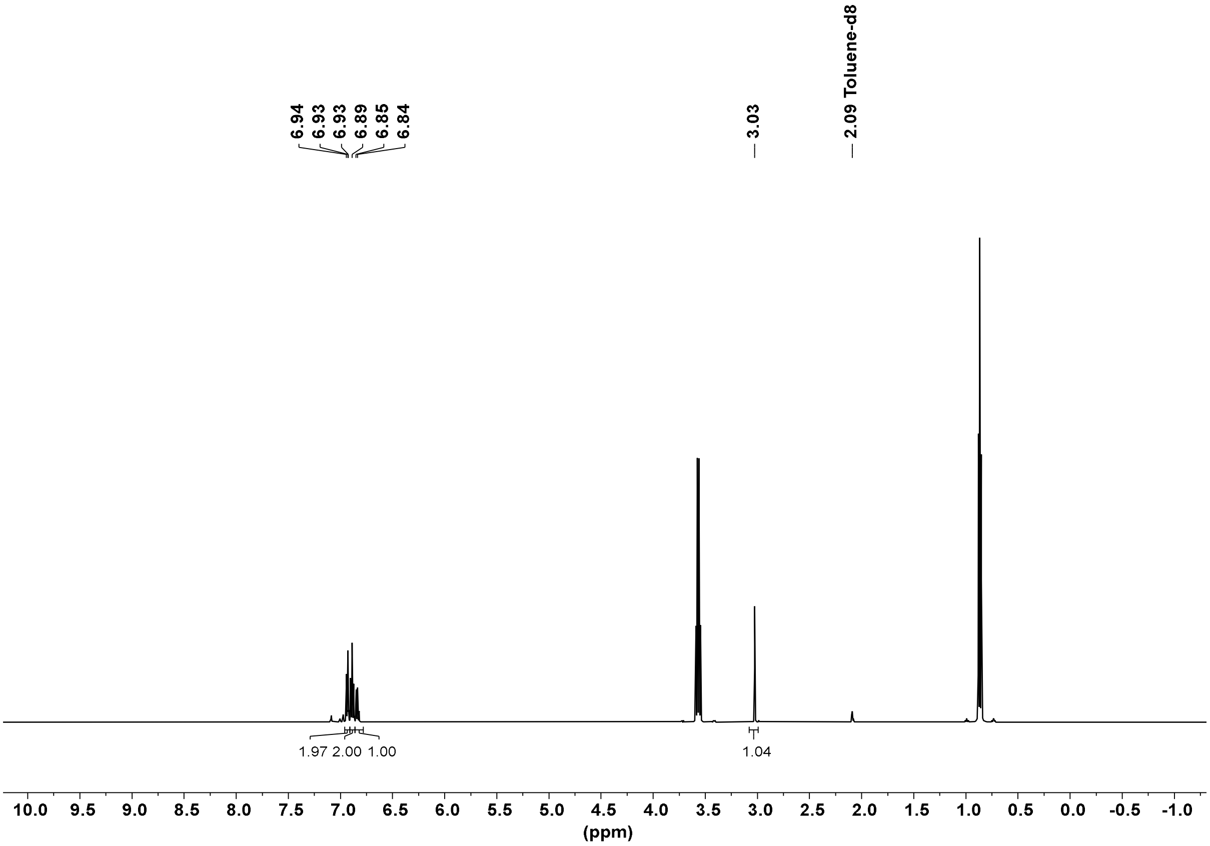

Figure S12. [11]H NMR spectrum showing thiophenol and ether.

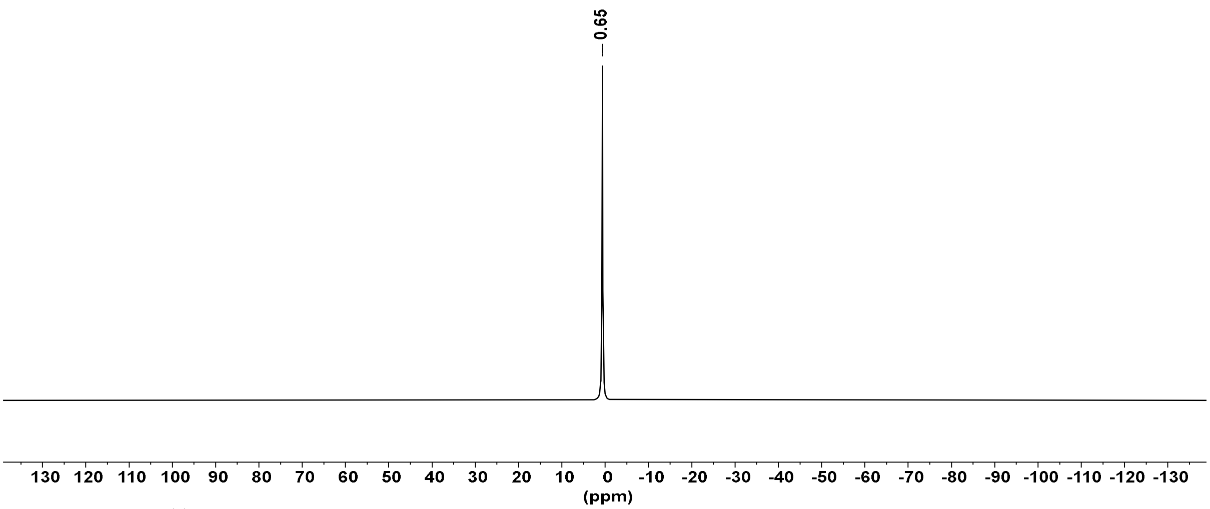

Figure S13. ^{11}B NMR spectrum showing presence of $Et_2O{\cdot}BF_3$.

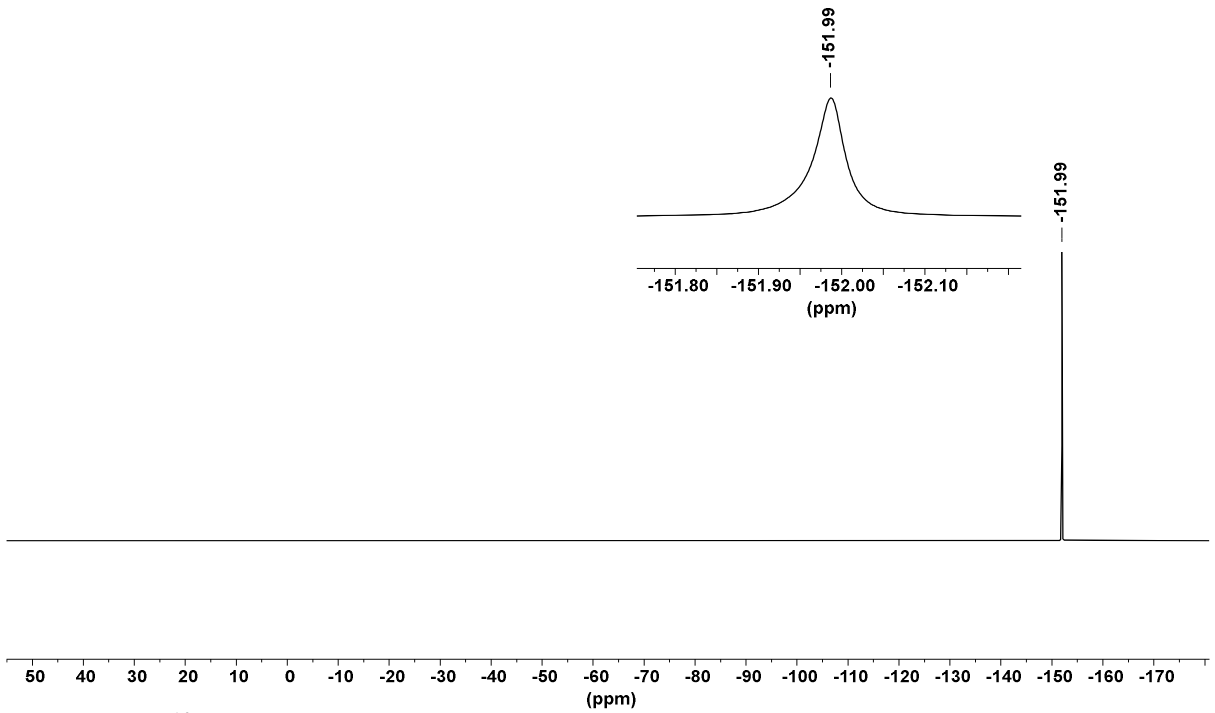

Figure S14. ^{19}F NMR spectrum showing the presence of $Et_2O{\cdot}BF_3$.

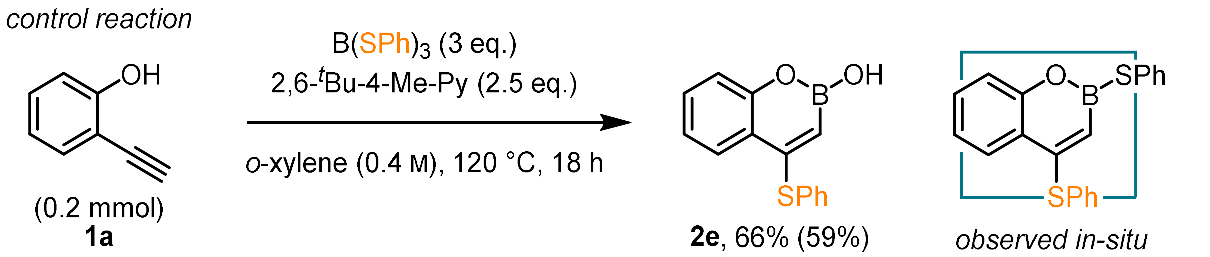

Following General Procedure 1b, using 2-ethynylphenol **1a** (24.0 mg, 0.200 mmol), 2,6-di-*tert*-butyl-4-methylpyridine (82.0 mg, 0.400 mmol) and $B(SPh)_3$ (203 mg, 0.600 mmol) in *o*-xylene at 120 °C gave title compound **2e** (66% spectroscopic yield). Purification by flash chromatography (CombiFlash Isco NextGen300+, 4 g SiO_2, CH_2Cl_2:MeOH, 100:0 to 90:10) gave the title compound **2e** as a pale yellow oil (30.0 mg, 0.12 mmol, 59%).

Data matched product **2e**.

S4. Synthesis of Starting Materials

General Procedure 4: Sonogashira coupling of iodoarenes with trimethylsilylacetylene

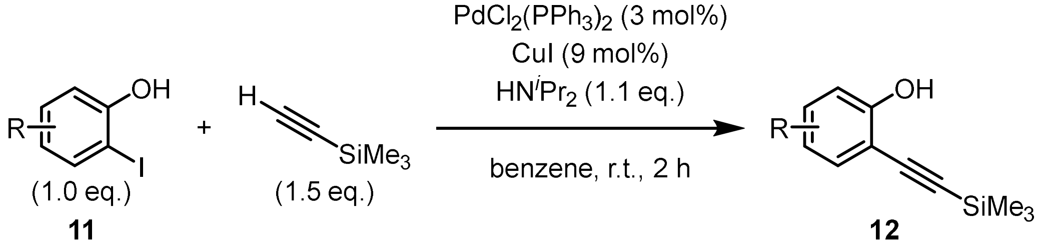

11 **12**

According to the literature procedure,[8] to a mixture of bis-(triphenylphosphine)palladium(II) dichloride (3.0 mol%), copper(I) iodide (9.0 mol%) and 2-iodophenol **11** (1.0 eq.) in benzene, was added trimethylsilylacetylene (1.5 eq.). The reaction was stirred vigorously and purged with Ar for 15 mins. Diisopropylamine (1.1 eq.) was then added slowly. The resulting solution was stirred at room temperature for 2 h. The mixture was passed through a short pad of celite and the solvent was removed *in vacuo*. The crude was dissolved in DCM and evaporated ono silica gel. The residue was purified by flash chromatography (eluent = ethyl acetate/petroleum ether mixtures) to get alkyne **12**.

General Procedure 5: Deprotection of trimethylsilane protecting group with KF

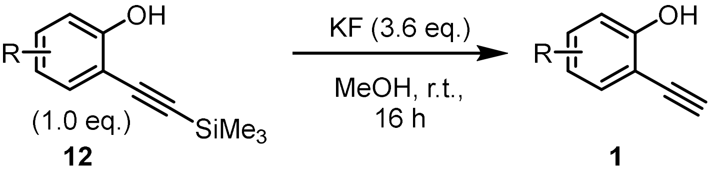

12 **1**

According to literature procedure,[9] a solution of the prepared 2-(trimethylsilylethynyl)-phenol **12** (1.0 eq.) and KF (3.6 eq.) was stirred in methanol for 16 h at r.t. The mixture was diluted with water (100 mL) and extracted with CH_2Cl_2 (3 × 50 mL). The combined organic layers were dried over $MgSO_4$, filtered, and concentrated carefully under reduced pressure, as some products were volatile. The residue was purified by flash chromatography (eluent = DCM/petroleum ether mixtures) and purified products **1** were stored in the freezer.

General Procedure 6: Synthesis of TBDMS-protected phenols

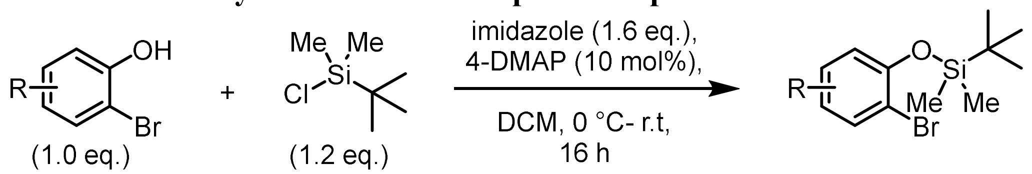

13 **14**

According to literature procedure,[10] in a two-necked flask, imidazole (1.6 eq.) and 4-DMAP (10 mol%). Phenol **13** (1.0 eq.) was dissolved in DCM and added before the solution was cooled to 0 °C, then TBDMSCl (1.2 eq.) was added in one portion. The suspension was allowed to warm to r.t and stirred overnight. The solution was filtered and the filtrate was evaporated under reduced pressure. The oil was re-dissolved in ether and the pH was adjusted to 1.0 with 2% $HCl_{(aq)}$. The organic layer was separated and washed with brine (2 × 50 mL) before it was dried over $MgSO_4$, filtered and concentrated under reduced pressure. The crude product **14** was used without further purification.

---

[8] M. Kimura, A. Ezoe, M. Mori and Y. Tamaru, *J. Am. Chem. Soc.* 2005, **127**, 201–209.
[9] A. Arcadi, S. Cacchi, M. D. Rosario, G. Fabrizi and F. Marinelli, *J. Org. Chem.* 1996, **61**, 9280-9288.
[10] M. S. Baker and S. T. Phillips, *J. Am. Chem. Soc.* 2011, **133**, 5170–5173.

General Procedure 7: Sonogashira coupling of bromoarenes with trimethylsilylacetylene

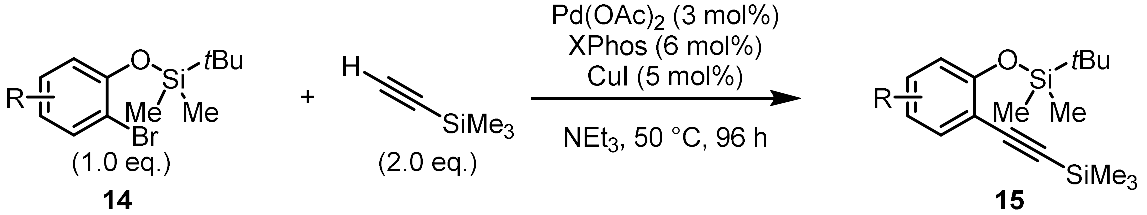

According to literature procedure,[11] to a J Youngs ampoule, was added bromoarene **14** (1.0 eq), copper(I) iodide (5.0 mol%), $Pd(OAc)_2$ (3.0 mol%) and XPhos (6.0 mol%). Dry and degassed triethylamine (7 mL) and trimethylsilylacetylene (2.0 eq.) were added sequentially. The mixture was heated to 50 °C for 96 h. After cooling to room temperature, the volatiles were removed in *vacuo*. The crude mixture was dissolved in DCM, evaporated onto Celite and the product **15** was purified by flash column chromatography (eluent = petroleum ether).

General Procedure 8: Deprotection of silyl groups with TBAF

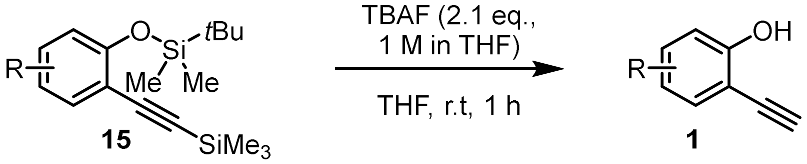

To a solution of silane protected alkynes **15** (1.0 eq.) in THF was added tetra-*n*-butylammonium fluoride (2.1 eq., 1 M in THF) at r.t. The reaction was stirred for 1 h. Then the reaction was quenched with saturated aqueous NH_4Cl solution and extracted with diethyl ether. The reaction mixture was washed with aqueous NH_4Cl and water. The organic layer was dried over $MgSO_4$ and concentrated carefully under reduced pressure, as some products were volatile. The desired product **1** was purified by flash column chromatography (eluent = DCM/petroleum ether mixtures) and stored in the freezer. Any residual silane impurities were removed by washing with pentane.

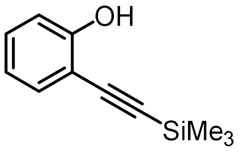

2-((Trimethylsilyl)ethynyl)phenol 12a

Following General Procedure 4 with 2-iodophenol (4.4 g, 20 mmol), copper(I) iodide (343 mg, 1.8 mmol, 0.09 eq.) $Pd(PPh_3)_2Cl_2$ (421 mg, 0.6 mmol, 0.03 eq.) and trimethylsilylacetylene (4.2 mL, 30 mmol, 1.5 eq.) in benzene (75 mL). The residue was purified by flash chromatography on silica gel (3% EtOAc/petroleum ether) to get the desired compound **12a** as an orange oil (3.75 g, 99%).

^{1}H NMR (500 MHz, $CDCl_3$) δ 7.34 (dd, J = 7.6, 1.6 Hz, 1H), 7.24 (ddd, J = 8.6, 8.2, 1.6 Hz, 1H), 6.96 (dd, J = 8.2, 1.0 Hz, 1H), 6.88, (ddd, J = 8.6, 7.6 1.0 Hz, 1H), 5.83 (s, 1H), 0.28 (s, 9H). **^{13}C NMR (126 MHz, $CDCl_3$)** δ 157.2, 131.7, 130.8, 120.4, 114.7, 109.7, 102.5, 99.1, 0.11. This data is in agreement with the literature.[12]

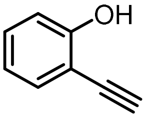

---

[11] J. Bucher, T. Wurm, K. S. Nalivela, M. Rudolph, F. Rominger and A. S. K. Hashmi, *Angew. Chem. Int. Ed.* 2014, **53**, 3854 –3858

[12] G. W. Kabalka, L. Wang, R. M. Pagni, *Tetrahedron,* 2001, **57**, 8017-8028.

2-Ethynylphenol 1a
Following General Procedure 5 with 2-((trimethylsilyl)ethynyl)phenol **12a** (2.06 g, 10.8 mmol, 1 eq.) and KF (2.25 g, 38.9 mmol, 3.6 eq.) in MeOH (60 mL). The residue was purified by flash chromatography on silica gel (30% DCM/petroleum ether) to get the desired compound **1a** as a pale brown oil (1.02 g, 80%).

^{1}H NMR (500 MHz, $CDCl_3$) δ 7.38 (dd, *J* = 7.6, 1.6 Hz, 1H), 7.28 (ddd, *J* = 8.6, 8.4, 1.6 Hz, 1H), 6.96 (dd, *J* = 8.4, 1.0 Hz, 1H), 6.88, (ddd, *J* = 8.6, 7.6 Hz, 1.0, 1H), 5.78 (s, 1H), 3.47 (s, 1H). **^{13}C NMR (126 MHz, $CDCl_3$)** δ 157.5, 132.2, 131.1, 120.5, 115.0, 108.4, 84.5, 78.4. This data is in agreement with the literature.[13]

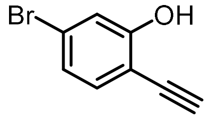

5-Bromo-2-ethynylphenol 1k
Following General Procedure 4 with 2-iodo-5-bromophenol (1.00 g, 3.35 mmol, 1 eq.), copper(I) iodide (12.8 mg, 0.07 mmol, 0.02 eq.), $Pd(PPh_3)_2Cl_2$ (23.5 mg, 0.03 mmol, 0.01 eq.) and trimethylsilylacetylene (0.56 mL, 4.0 mmol, 1.2 eq.) in THF (10 mL) and NEt_3 (4 mL). The silylated alkyne was confirmed via NMR analysis and used in the next step as a crude material.

NMR of TMS-alkyne derivative. ^{1}H NMR (500 MHz, $CDCl_3$) δ 7.19 (d, *J* = 8.2 Hz, 1H), 7.14 (d, *J* = 1.8 Hz, 1H), 7.00 (dd, *J* = 8.2, 1.8 Hz, 1H), 5.85 (s, 1H), 0.28 (s, 9H).

Following General Procedure 8 with 5-bromo-2-((trimethylsilyl)ethynyl)phenol (0.40 g, 1.49 mmol, 1 eq.) and TBAF (1.86 mL, 1.86 mmol, 1 M in THF, 1.25 eq.) in THF (4 mL). The residue was purified by flash chromatography on silica gel (30% DCM/petroleum ether) to get the desired compound **1k** as a white solid (0.26 g, 89%).

^{1}H NMR (500 MHz, $CDCl_3$) δ 7.23 (d, *J* = 8.3 Hz, 1H), 7.15 (d, *J* = 2.0 Hz, 1H), 7.03 (dd, *J* = 8.3, 2.0 Hz, 1H), 5.82 (s, 1H), 3.51 (s, 1H). **^{13}C NMR (126 MHz, $CDCl_3$)** δ 158.0, 133.0, 124.5, 123.9, 118.5, 107.6, 85.6, 77.4. This data is in agreement with the literature.[14]

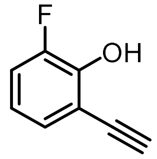

6-Fluoro-2-ethynylphenol 1l
Following General Procedure 4 with 2-iodo-6-fluorophenol (0.500 g, 2.10 mmol, 1 eq.), copper(I) iodide (36.0 mg, 0.19 mmol, 0.09 eq.), $Pd(PPh_3)_2Cl_2$ (44.2 mg, 0.06 mmol, 0.03 eq.) and trimethylsilylacetylene (0.44 mL, 3.15 mmol, 1.5 eq.) in benzene (10 mL). The silylated alkyne was confirmed via NMR analysis and used in the next step as a crude material.

NMR data of TMS-alkyne derivative. ^{1}H NMR (500 MHz, $CDCl_3$) δ 7.13 (dt, *J* = 7.9, 1.5 Hz, 1H), 7.06 (ddd, *J* = 9.9, 8.4, 1.5 Hz, 1H), 6.81-6.75 (m, 1H), 5.68 (s, 1H), 0.28 (s, 9H). **^{19}F NMR (471 MHz, $CDCl_3$)** δ -137.0 (dd, *J* = 10.5, 4.9 Hz).

---
[13] See ref 11
[14] K. Yuan, M. J. Ingleson, *Angew. Chem. Int. Ed.* 2023, **62**, e202301463

Following General Procedure 8, with TBAF (2.1 mL, 2.1 mmol, 1 M in THF, 1.0 eq.) in THF (3 mL). The residue was purified by flash chromatography on silica gel (30% DCM/petroleum ether) to get the desired compound **1l** as a clear oil (67.6 mg, 24%).

^{1}H NMR (500 MHz, CDCl$_3$) δ 7.18 (dt, *J* = 7.9, 1.4 Hz, 1H), 7.10 (ddd, *J* = 9.7, 8.3, 1.4 Hz, 1H), 6.84-6.79 (m, 1H), 5.62 (s, 1H), 3.47 (s, 1H). **^{19}F NMR (471 MHz, CDCl$_3$)** δ -137.4 (dd, *J* = 10.3, 4.6 Hz). **^{13}C NMR (126 MHz, CDCl$_3$)** δ 150.8 (d, *J* = 242.4 Hz), 145.8 (d, *J* = 13.5 Hz), 128.0 (d, *J* = 3.5 Hz), 120.4 (d, *J* = 7.5 Hz), 117.4 (d, *J* = 18.3 Hz), 111.0 (d, *J* = 3.3 Hz), 84.5, 77.4. This data is in agreement with the literature.[15]

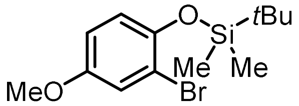

(2-Bromo-4-(methoxy)phenoxy)(*tert*-butyl)dimethylsilane 14a

Following General Procedure 6 with 2-bromo-4-methoxyphenol (2.00 g, 9.85 mmol, 1 eq.), 4-dimethylaminopyridine (120 mg, 0.99 mmol, 0.1 eq.), imidazole (1.07 g, 15.8 mmol, 1.6 eq.) and *tert*-butyldimethylsilyl chloride (1.78 g, 11.8 mmol, 1.2 eq) in DCM (50 mL). The crude product **14a** was a colourless oil (3.31 g) and used without further purification.

^{1}H NMR (500 MHz, CDCl$_3$) δ 7.07 (d, *J* = 3.0 Hz, 1H), 6.79 (d, *J* = 8.9 Hz, 1H), 6.73 (dd, *J* = 8.9, 3.0 Hz, 1H), 3.75 (s, 3H), 1.03 (s, 9H), 0.22 (s, 6H). This data is in agreement with the literature.[16]

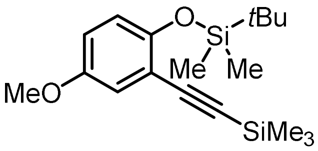

***tert*-Butyl(4-methoxy-2-((trimethylsilyl)ethynyl)phenoxy)dimethylsilane 15a**

Following General Procedure 7 with (2-bromo-4-(methoxy)phenoxy)(*tert*-butyl)dimethylsilane **14a** (2.30 g, 7.25 mmol, 1 eq.), copper(I) iodide (69.0 mg, 0.36 mmol, 0.05 eq.), Pd(OAc)$_2$ (48.8 mg, 0.22 mmol, 0.03 eq.), XPhos (207 mg, 0.44 mmol, 0.06 eq.) and trimethylsilylacetylene (2.00 mL, 14.5 mmol, 2 eq.) in NEt$_3$ (40 mL). The residue was purified by flash chromatography on silica gel (petroleum ether) to get the desired compound **15a** as a brown oil (1.46 g, 60%).

^{1}H NMR (500 MHz, CDCl$_3$) δ 6.91 (d, *J* = 3.1 Hz, 1H), 6.75 (dd, *J* = 8.8, 3.1 Hz, 1H), 6.71 (d, *J* = 8.8 Hz, 1H), 3.75 (s, 3H), 1.03 (s, 9H), 0.24 (s, 9H), 0.21 (s, 6H). **^{13}C NMR (126 MHz, CDCl$_3$)** δ 153.6, 151.0, 120.7, 117.6, 116.8, 115.8, 102.5, 97.8, 55.9, 26.0, 18.4, 0.2, -4.1. This data is in agreement with the literature.[17]

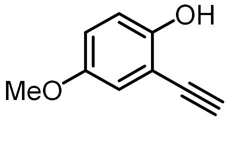

4-Methoxy-2-ethynylphenol 1j

Following General Procedure 8 with *tert*-butyl(4-methoxy-2-((trimethylsilyl)ethynyl)phenoxy)dimethylsilane **15a** (1.46 g, 4.37 mmol, 1 eq.) and TBAF (10.1 mL, 1 M in THF, 10.1 mmol, 2.3 eq.) in THF (10 mL) at r.t for 1 h. The residue was

[15] See ref 14

[16] See ref 11

[17] See ref 11

purified by flash chromatography on silica gel (30% DCM/petroleum ether) to get the desired compound **1j** as an orange oil (0.36 g, 55%).

^{1}H NMR (500 MHz, $CDCl_3$) δ 6.89-6.88 (m, 1H), 6.87-6.86 (m, 2H), 5.46 (s (br), 1H), 3.75 (s, 3H), 3.45 (s, 1H). **^{13}C NMR (126 MHz, $CDCl_3$)** δ 153.1, 151.9, 118.2, 115.9, 115.8, 108.4, 84.2, 78.6, 56.0.

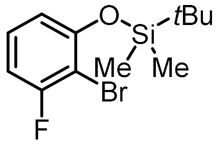

(2-Bromo-3-(fluoro)phenoxy)(*tert*-butyl)dimethylsilane 14b

Following General Procedure 6 with 2-bromo-3-fluorophenol (3.00 g, 15.7 mmol, 1 eq.), 4-dimethylaminopyridine (192 mg, 1.57 mmol, 0.1 eq.), imidazole (1.71 g, 25.1 mmol, 1.6 eq.) and *tert*-butyldimethylsilyl chloride (2.84 g, 18.8 mmol, 1.2 eq) in DCM (50 mL). The crude product **14b** was a colourless oil (4.75 g) and used without further purification.

^{1}H NMR (500 MHz, $CDCl_3$) δ 7.12 (td, *J* = 8.4, 6.5 Hz, 1H), 6.74 (td, *J* = 8.4, 1.3 Hz, 1H), 6.66 (dt, *J* = 8.4, 1.3 Hz, 1H), 1.05 (s, 9H), 0.26 (s, 6H). **^{19}F NMR (471 MHz, $CDCl_3$)** δ -104.5 (dd, *J* = 6.5, 1.3 Hz). **^{13}C NMR (126 MHz, $CDCl_3$)** δ 160.5 (d, *J* = 246.0 Hz), 154.5 (d, *J* = 3.6 Hz), 128.1 (d, *J* = 9.6 Hz), 115.6 (d, *J* = 3.0 Hz), 109.1 (d, *J* = 22.8 Hz), 103.4, (d, *J* = 21.0 Hz), 25.8, 18.5, -4.1. **HRMS (ESI$^+$):** calculated for ($C_{12}H_{19}BrFOSi$ $[M+H]^+$) = 305.0367; found 305.0367.

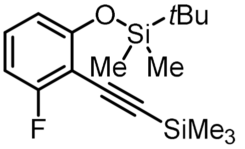

***tert*-Butyl(3-fluoro-2-((trimethylsilyl)ethynyl)phenoxy)dimethylsilane 15b**

Following General Procedure 7 with (2-bromo-3-(fluoro)phenoxy)(*tert*-butyl)dimethylsilane **14b** (4.75 g, 15.6 mmol, 1 eq.), copper(I) iodide (148 mg, 0.78 mmol, 0.05 eq.), $Pd(OAc)_2$ (105 mg, 0.47 mmol, 0.03 eq.), XPhos (446 mg, 0.94 mmol, 0.06 eq.) and trimethylsilylacetylene (4.30 mL, 31.2 mmol, 2 eq.) in NEt_3 (50 mL). The residue was purified by flash chromatography on silica gel (petroleum ether) to get the desired compound **15b** as a pale yellow oil (1.50 g, 30%).

^{1}H NMR (500 MHz, $CDCl_3$) δ 7.11 (td, *J* = 8.4, 6.5 Hz, 1H), 6.67 (td, *J* = 8.4, 0.9 Hz, 1H), 6.59 (dt, *J* = 8.4, 0.9 Hz, 1H), 1.04 (s, 9H), 0.25 (s, 15H). **^{19}F NMR (471 MHz, $CDCl_3$)** δ -108.0 (app t, *J* = 6.5 Hz). **^{13}C NMR (126 MHz, $CDCl_3$)** δ 164.3 (d, *J* = 251.3 Hz), 158.4 (d, *J* = 5.2 Hz), 129.5 (d, *J* = 10.5 Hz), 115.2 (d, *J* = 3.7 Hz), 108.2 (d, *J* = 21.7 Hz), 105.5, (d, *J* = 16.5 Hz), 103.7 (d, *J* = 3.5 Hz), 95.1 (d, *J* = 1.0 Hz), 25.9, 18.4, 0.07, -4.1. **HRMS (LDI$^+$):** calculated for ($C_{17}H_{28}FOSi_2$ $[M+H]^+$) = 323.1657; found 323.1659. **HRMS (LDI$^+$):** calculated for ($C_{17}H_{27}FOSi_2Na$ $[M+Na]^+$) = 345.1477; found 345.1479.

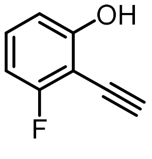

3-Fluoro-2-ethynylphenol 1g

Following General Procedure 8 with *tert*-butyl(3-fluoro-2-((trimethylsilyl)ethynyl)phenoxy)dimethylsilane **15b** (1.50 g, 4.66 mmol, 1 eq.) and TBAF (9.30 mL, 1 M in THF, 9.32 mmol, 2.0 eq.) in THF (6 mL) at r.t for 1 h. The residue was

purified by flash chromatography on silica gel (30% DCM/petroleum ether) and the solid washed with pentane to get the desired compound **1g** as a white fluffy solid (0.38 g, 60%).

^{1}H NMR (500 MHz, CDCl$_3$) δ 7.22 (td, *J* = 8.4, 6.5 Hz, 1H), 6.76 (dt, *J* = 8.4, 0.9 Hz, 1H), 6.66 (td, *J* = 8.4, 0.9 Hz, 1H), 5.84 (s, 1H), 3.67 (s, 1H). **^{19}F NMR (471 MHz, CDCl$_3$)** δ -109.1 (dd, *J* = 8.5, 6.5 Hz). **^{13}C NMR (126 MHz, CDCl$_3$)** δ 163.3 (d, *J* = 252.3 Hz), 158.7 (d, *J* = 3.9 Hz), 131.1 (d, *J* = 10.2 Hz), 110.7 (d, *J* = 3.1 Hz), 107.4 (d, *J* = 20.3 Hz), 98.6, (d, *J* = 18.4 Hz), 89.0 (d, *J* = 3.1 Hz), 72.6 (d, *J* = 1.9 Hz). **HRMS (ESI$^+$):** calculated for (C_8H_5FO [M]$^+$) = 136.0319; found 136.0320. **HRMS (ESI$^+$):** calculated for (C_8H_6FO [M+H]$^+$) = 137.0397; found 137.0398.

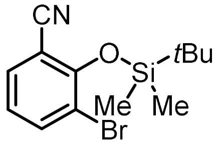

(2-Bromo-6-(cyano)phenoxy)(*tert*-butyl)dimethylsilane 14c

Following General Procedure 6 with 3-bromo-2-hydroxybenzonitrile (2.00 g, 9.85 mmol, 1 eq.), 4- dimethylaminopyridine (120 mg, 0.99 mmol, 0.1 eq.), imidazole (1.07 g, 15.8 mmol, 1.6 eq.) and *tert*-butyldimethylsilyl chloride (1.78 g, 11.8 mmol, 1.2 eq) in DCM (50 mL). The crude product **14c** was a colourless oil (3.31 g) and used without further purification.

^{1}H NMR (500 MHz, CDCl$_3$) δ 7.75 (dd, *J* = 7.9, 1.7 Hz, 1H), 7.50 (dd, *J* = 7.9, 1.7 Hz, 1H), 6.91 (t, *J* = 7.9 Hz, 1H), 1.09 (s, 9H), 0.38 (s, 6H). **^{13}C NMR (126 MHz, CDCl$_3$)** δ 155.2, 138.5, 133.2, 122.9, 117.0, 116.6, 107.1, 26.0, -2.6. **HRMS (LDI$^+$):** calculated for ($C_{13}H_{18}{}^{81}BrNOSiNa$ [M+Na]$^+$) = 336.0213; found 336.0214.

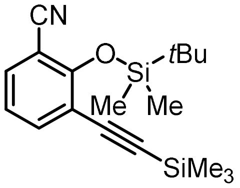

***tert*-Butyl(6-cyano-2-((trimethylsilyl)ethynyl)phenoxy)dimethylsilane 15c**

Following General Procedure 7 with (2-bromo-6-(cyano)phenoxy)(*tert*-butyl)dimethylsilane **14c** (1.45 g, 9.7 mmol, 1 eq.), copper(I) iodide (44.3 mg, 0.23 mmol, 0.05 eq.), Pd(OAc)$_2$ (31.3 mg, 0.14 mmol, 0.03 eq.), XPhos (134 mg, 0.28 mmol, 0.06 eq.) and trimethylsilylacetylene (1.30 mL, 9.30 mmol, 2 eq.) in NEt$_3$ (40 mL). The residue was purified by flash chromatography on silica gel (petroleum ether) to get the desired compound **15c** as a pale yellow oil (0.96 g, 62%).

^{1}H NMR (500 MHz, CDCl$_3$) δ 7.62 (dd, *J* = 7.8, 1.9 Hz, 1H), 7.48 (dd, *J* = 7.8, 1.9 Hz, 1H), 6.97 (t, *J* = 7.8 Hz, 1H), 1.09 (s, 9H), 0.36 (s, 6H), 0.25 (s, 9H). **^{13}C NMR (126 MHz, CDCl$_3$)** δ 158.5, 139.3, 134.0, 121.8, 117.7, 117.2, 106.5, 100.9, 100.8, 25.9, 18.7, -0.08, -2.9. **HRMS (LDI$^+$):** calculated for ($C_{13}H_{28}NOSi_2$ [M]$^+$) = 330.1704; found 330.1711. **HRMS (LDI$^+$):** calculated for ($C_{13}H_{27}NOSi_2Na$ [M+Na]$^+$) = 352.1523; found 352.1528.

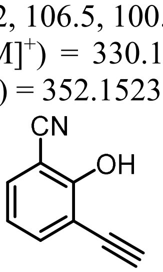

3-Ethynyl-2-hydroxybenzonitrile 1i

Following General Procedure 8 with *tert*-butyl(6-cyano-2-((trimethylsilyl)ethynyl)phenoxy)dimethylsilane **15c** (0.36 g, 1.10 mmol, 1 eq.) and TBAF (2.3

mL, 1 M in THF, 2.3 mmol, 2.1 eq.) in THF (2 mL) at r.t for 1 h. The residue was purified by flash chromatography on silica gel (100% DCM) to get the desired compound **1i** as a white solid (61.4 mg, 39%).

^{1}H NMR (500 MHz, CDCl$_3$) δ 7.60 (dd, *J* = 7.8, 1.5 Hz, 1H), 7.55 (dd, *J* = 7.8, 1.5 Hz, 1H), 6.98 (t, *J* = 7.8 Hz, 1H), 6.38 (s, 1H), 3.59 (s, 1H). **^{13}C NMR (126 MHz, CDCl$_3$)** δ 159.3, 136.7, 134.3, 121.0, 115.4, 110.2, 100.2, 86.7, 76.3. **HRMS (LDI$^+$):** calculated for (C_9H_5NONa [M+Na]$^+$) = 166.0263; found 166.0263.

N-Benzylation of 1,2,3,4-tetrahydroquinoline

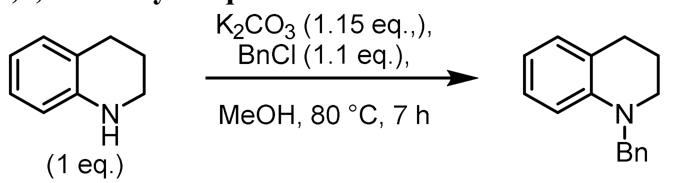

According to literature procedure,[18] to a one-necked flask charged with a stir bar, was added 1,2,3,4-tetrahydroquinoline (1.00 g, 8.39 mmol), K_2CO_3 (1.33 g, 9.65 mmol) and MeOH (10 mL). BnCl (1.10 mL, 9.23 mmol) was added under stirring. The flask was sealed and heated at 80 °C for 7 h. The reaction mixture was then filtered through Celite, washed with EtOAc and the volatiles removed under reduced pressure. The residue was redissolved in EtOAc (10 mL) and separated with water (50 mL). The aqueous phase was extracted with EtOAc (3 x 50 mL). The combined organic layers were washe with water (30 mL), brine (30 mL), dried over $MgSO_4$, filtered and solvent removed in *vacuo*. The residue was purified by flash chromatography (eluent = 10% DCM/petroleum ether) to get the target compound as a clear oil (1.26 g, 5.36 mmol, 67%).

^{1}H NMR (500 MHz, CDCl$_3$) δ 7.35-7.30 (m, 2H), 7.28-7.22 (m, 3H), 6.99-6.95 (m, 2H), 6.58 (app t, *J* = 7.2 Hz, 1H), 6.51 (app d, *J* = 8.2 Hz, 1H), 4.48 (s, 2H), 3.37 (t, *J* = 6.5 Hz, 2H), 2.82 (t, *J* = 6.5 Hz, 2H), 2.02 (app quin., *J* = 6.5 Hz, 2H). **^{13}C NMR (126 MHz, CDCl$_3$)** δ 145.8, 139.1, 129.1, 128.7, 127.3, 126.9, 126.7, 122.4, 116.0, 111.1, 55.3, 50.0, 28.4, 22.5. This data is in agreement with the literature.[19]

Monobenzylation of aniline

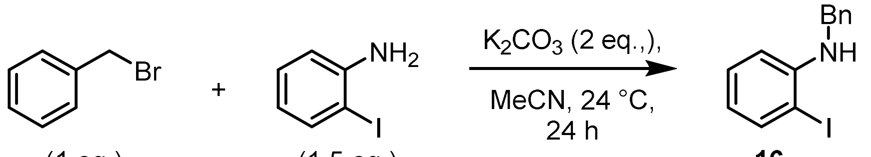

16

According to literature procedure,[20] to a one-necked flask charged with a stir bar, was added aniline (1.92 g, 8.77 mmol), K_2CO_3 (1.62 g, 11.7 mmol) and MeCN (15 mL). BnBr (1.00 g, 5.85 mmol) was added slowly under stirring. The flask was sealed and warmed to 24 °C for 24 h. The reaction mixture was then filtered through Celite, washed with EtOAc and the volatiles removed under reduced pressure. The residue was purified by flash chromatography (eluent = 10% DCM/petroleum ether) to get the target compound **16** as a clear oil (0.970 g, 3.14 mmol, 54%).

^{1}H NMR (500 MHz, CDCl$_3$) δ 7.68 (dd, *J* = 7.9, 1.6 Hz, 1H), 7.38-7.36 (m, 4H), 7.30-7.27 (m, 1H), 7.16 (ddd, *J* = 8.4, 7.7, 1.3 Hz, 1H), 6.54 (dd, *J* = 8.4, 1.5 Hz, 1H), 6.45 (ddd, *J* = 8.8,

---

[18] A. Noble and D. W. C. MacMillan, *J. Am. Chem. Soc.* 2014, **136**, 11602−11605.
[19] See ref 18
[20] L. Yang, Z. Qiu, J. Wu, J. Zhao, T. Shen, X. Huang and Z.-Q. Liu, *Org. Lett.* 2021, **23**, 3207−3210.

7.7, 1.4 Hz, 1H), 4.63 (s (br), 1H), 4.41 (d, J = 5.5 Hz, 2H). **^{13}C NMR (126 MHz, CDCl$_3$)** δ 147.2, 139.2, 138.8, 129.6, 128.9, 127.5, 127.3, 119.0, 111.1, 85.5, 48.5. This data is in agreement with the literature.[21]

Sonogashira coupling of iodoaniline derivative with trimethylsilylacetylene

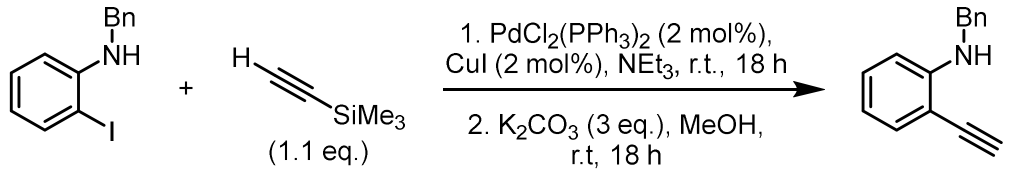

16 **3**

According to literature procedure,[22] to a mixture of bis-(triphenylphosphine)palladium(II) dichloride (44.0 mg, 0.06 mmol), copper(I) iodide (12.0 mg, 0.06 mmol) and *N*-benzyl-2-iodoaniline **16** (0.970 g, 3.14 mmol) in NEt_3 (14 mL), was added trimethylsilylacetylene (0.49 mL, 3.44 mmol) slowly. The resulting solution was stirred at room temperature for 18 h. The reaction was then quenched with NH_4Cl and the layers separated. The aqueous layer was extracted with EtOAc (3 x 50 mL). The combined organic layers were washed with water (30 mL), brine (30 mL), dried over $MgSO_4$, filtered and solvent removed in *vacuo*. The crude was redissolved in MeOH (15 mL) and K_2CO_3 (1.3019 g, 9.42 mmol) was added. The mixture was allowed to stir at r.t overnight before it was diluted with hexane, filtered and solvent removed in *vacuo*. The residue was purified by flash chromatography (eluent = 3% ethyl acetate/petroleum ether) to get the target compound **3** as a yellow oil (0.406 g, 1.96 mmol, 62%).

^{1}H NMR (500 MHz, CDCl$_3$) δ 7.39-7.33 (m, 5H), 7.30-7.27 (m, 1H), 7.17 (ddd, J = 8.5, 7.7, 1.3 Hz, 1H), 6.63 (ddd, J = 8.5, 7.5, 1.2 Hz, 1H), 6.57 (d (br), J = 8.4 Hz, 1H), 5.08 (s (br), 1H), 4.43 (d, J = 5.9 Hz, 2H), 3.40 (s, 1H). **^{13}C NMR (126 MHz, CDCl$_3$)** δ 149.5, 139.1, 132.8, 130.5, 128.8, 127.4, 127.3, 116.6, 110.1, 106.4, 83.1, 80.9, 47.8. This data is in agreement with the literature.[23]

Corey-Fuchs preparation of alkyne

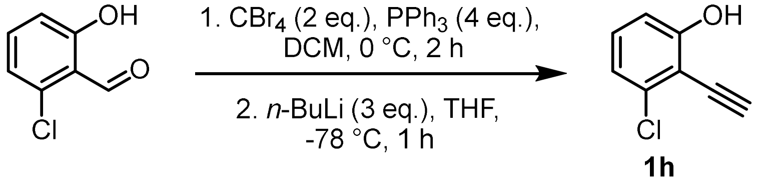

1h

According to the literature procedure,[24] to a stirred solution of 3-chlorosalicylaldehyde (1.57 g, 10.0 mmol) and carbon tetrabromide (6.63 g, 20.0 mmol) in DCM (10 mL) was added PPh_3 (10.49 g, 40.0 mmol) slowly in portions at 0 °C. The reaction was stirred at 0 °C for 1 h before it was quenched with water (50 mL) and the layers separated. The aqueous layer was extracted with DCM (3 x 50 mL). The combined organic layers were washed with water (30 mL), brine (30 mL), dried over $MgSO_4$, filtered and solvent removed in *vacuo*. The residue was purified by flash column chromatography (eluent = EtOAc:petroleum ether 0:100 to 100:0) to give dibromoalkene (1.21 g, 3.88 mmol). The product was dissolved in THF (15 mL) before *n*-BuLi

[21] See ref 20

[22] D. S. Casadio, S. Aikonen, A. Lenarda, M. Nieger, T. Hu, S. Tauber, D. Sundholm., M. Muuronen, T. Wirtanen and J. Helaja, *Chem. Eur. J.* 2021, **27**, 5283 – 5291.

[23] M.-X. Fu, J.-H. Lin, J-C. Xiao, *Org. Lett.* 2024, **26**, 6065−6069.

[24] Z. Rong, W. Hu, N. Dai and G. Qian, *Org. Lett.* 2020, **22**, 3286−3290.

(4.7 mL, 2.5 M in hexanes, 11.6 mmol) was added dropwise at -78 °C. The reaction was stirred at -78 °C for 1 h before it was quenched with saturated aqueous NH_4Cl (50 mL). The aqueous layer was extracted with DCM (3 x 50 mL). The combined organic layers were washed with water (30 mL), brine (30 mL), dried over $MgSO_4$, filtered and solvent removed in *vacuo*. The residue was purified by flash column chromatography (eluent = 30% DCM/petroleum ether) to give 3-chloro-2-ethynylphenol **1h** as an orange oil (174.5 mg, 1.14 mmol, 29%).

^{1}H NMR (500 MHz, $CDCl_3$) δ 7.18 (t, *J* = 8.2, Hz, 1H), 6.97 (dd, *J* = 8.2, 1.0 Hz, 1H), 6.87 (dd, *J* = 8.2, 1.0 Hz, 1H), 6.00 (s, 1H), 3.75 (s, 1H). **^{13}C NMR (126 MHz, $CDCl_3$)** δ 158.7, 136.0, 130.9, 121.3, 113.3, 109.3, 89.2, 75.5. This data is in agreement with the literature.[25]

Synthesis of internal alkyne

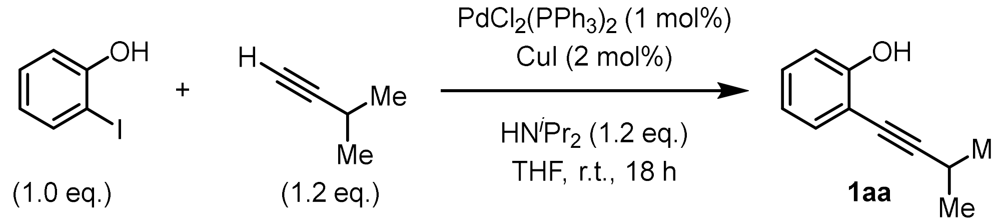

To a mixture of bis-(triphenylphosphine)palladium(II) dichloride (160 mg, 0.02 mmol), copper(I) iodide (8.70 mg, 0.05 mmol) and 2-iodophenol (500 mg, 2.27 mmol) in THF (11 mL), was added 3-methyl-1-butyne (0.28 mL, 2.72 mmol). Diisopropylamine (0.32 mL, 2.72 mmol) was then added slowly. The resulting solution was stirred at room temperature for 18 h. The mixture was passed through a short pad of celite, washed with EtOAc and the solvent was removed *in vacuo*. The crude was dissolved in DCM and evaporated ono silica gel. The residue was purified by flash chromatography (eluent = 10% DCM/petroleum ether) to give **1aa** as a pale-yellow oil (226 mg, 1.41 mmol, 62%).

^{1}H NMR (500 MHz, $CDCl_3$) δ 7.29 (dd, *J* = 7.7, 1.6 Hz, 1H), 7.20 (ddd, *J* = 8.4, 7.6, 1.6 Hz, 1H), 6.93 (m, 1H), 6.84, (ddd, *J* = 8.4, 7.7 1.2 Hz, 1H), 5.77 (s, 1H), 2.85 (sept, *J* = 6.9 Hz, 1H) 1.30 (d, *J* = 6.9 Hz, 6H). **^{13}C NMR (126 MHz, $CDCl_3$)** δ 156.5, 131.5, 129.8, 120.3, 114.4, 110.3, 103.6, 73.8, 23.2, 21.5. **HRMS (LDI$^+$):** calculated for ($C_{11}H_{13}O$ $[M+H]^+$) = 161.0961; found 161.0954.

[25] See ref 24

S5. NMR Spectra

Note, is some cases D_2O (40 uL) was added to break-up the B-O-B anhydride. In these cases, the B–OH resonance is absent. The resonance for HDO is observed and in most cases, methanol is observed in the spectra. Some residual traces of DCM can be observed in a few cases.

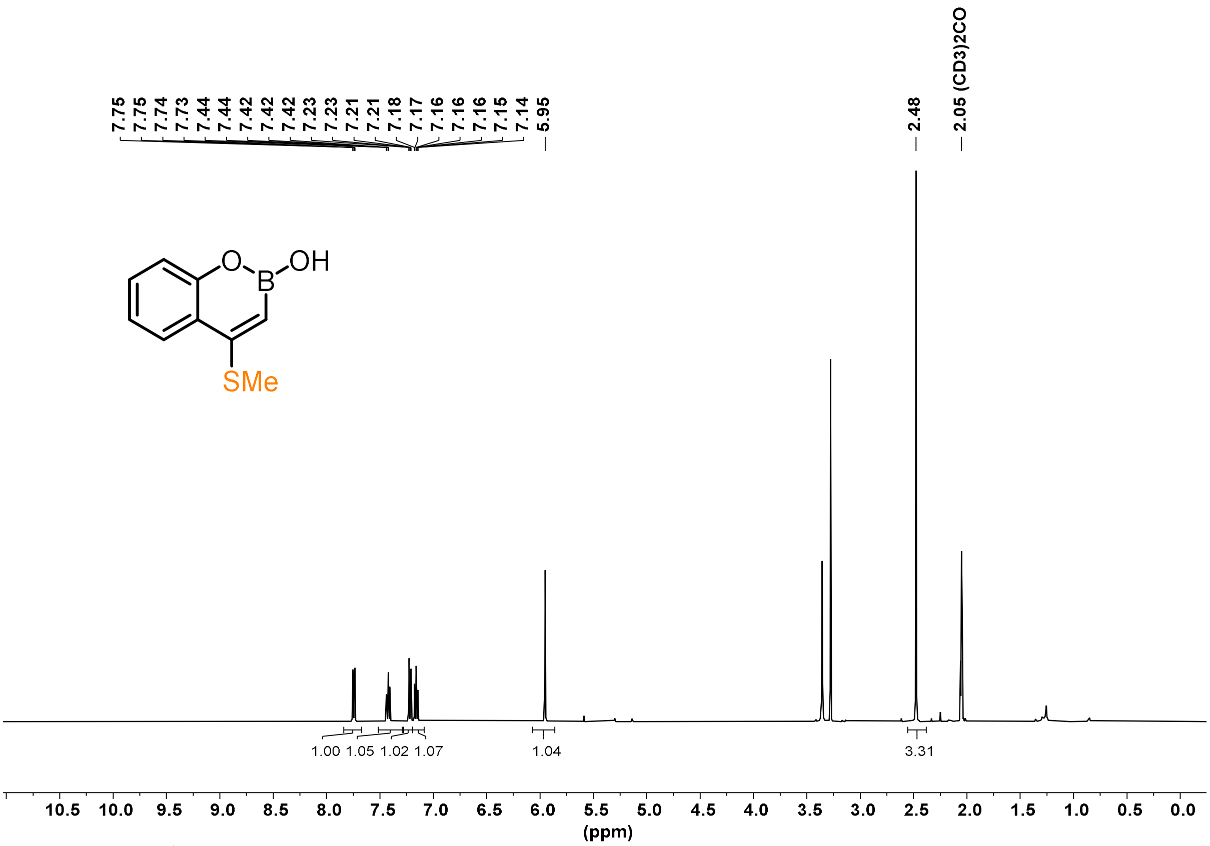

Figure S15. ^{1}H NMR spectrum of compound **2a** in acetone-D_6.

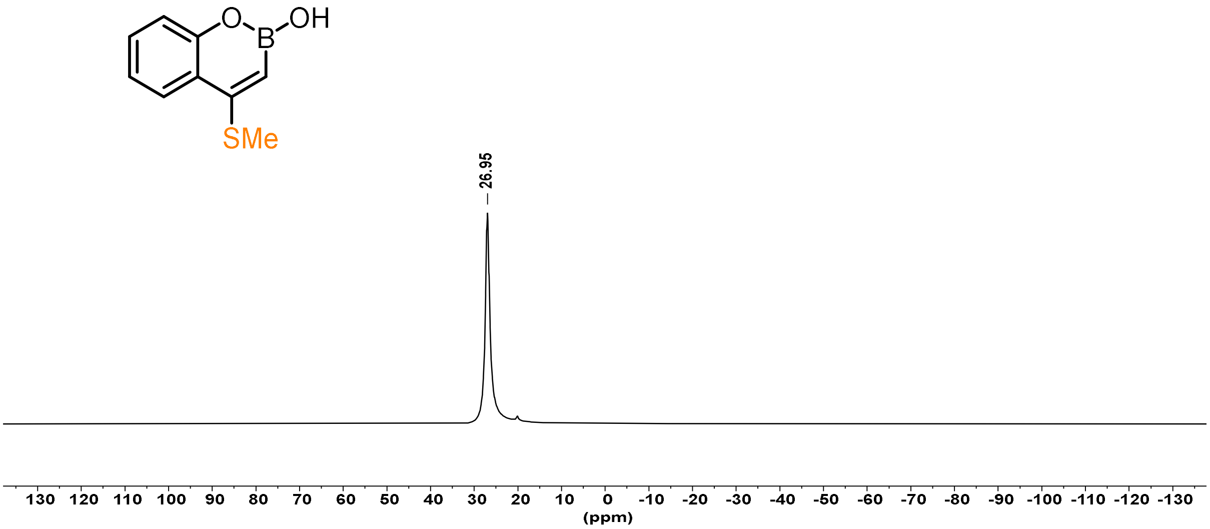

Figure S16. ^{11}B NMR spectrum of compound **2a** in acetone-D_6.

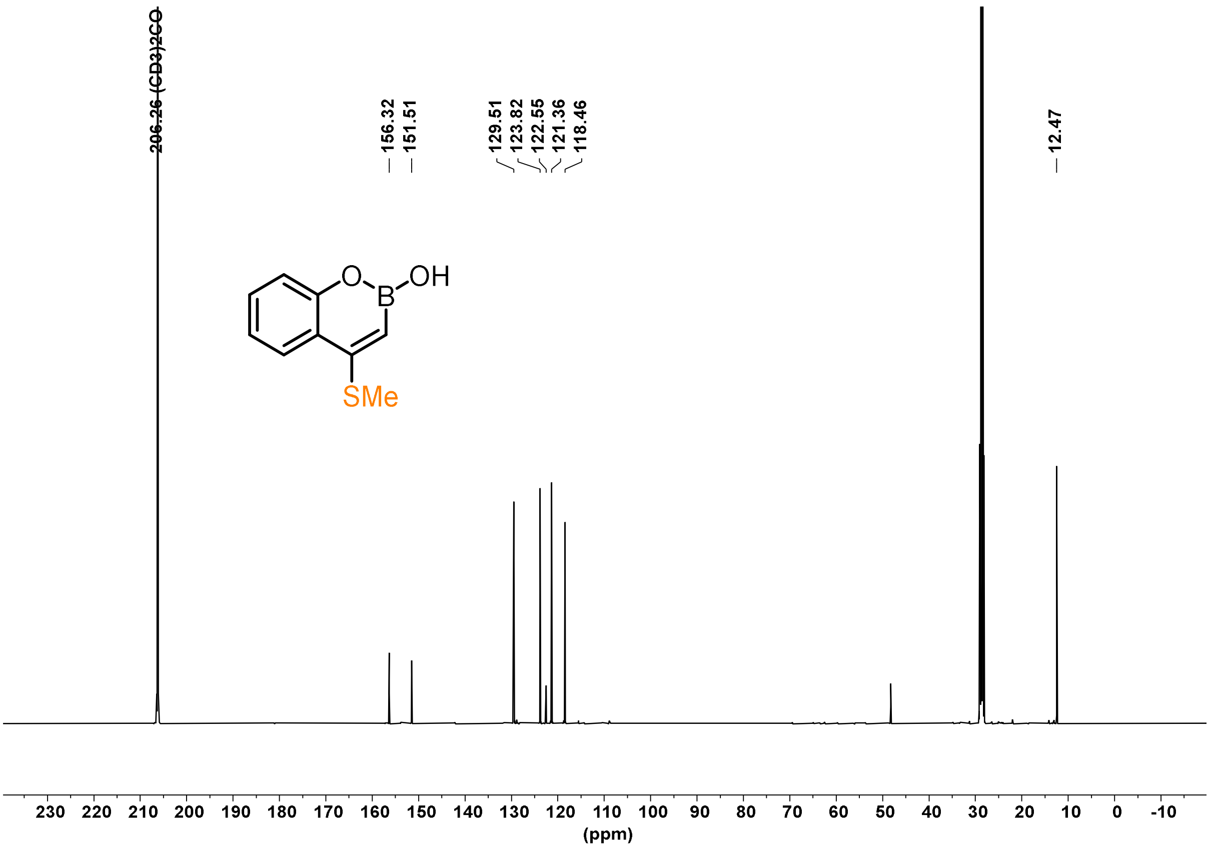

Figure S17. $^{13}C\{^{1}H\}$ NMR spectrum of compound **2a** in acetone-D6.

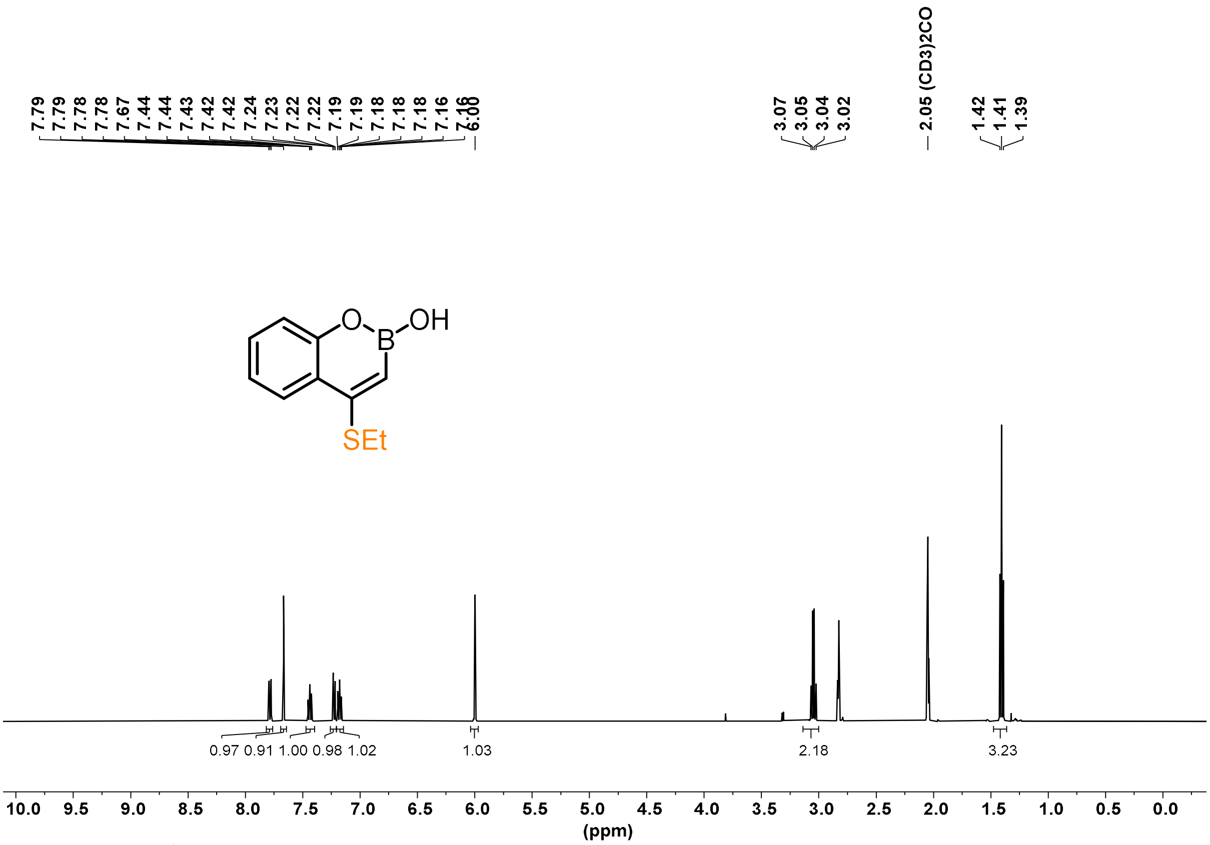

Figure S18. ^{1}H NMR spectrum of compound **2b** in acetone-D6.

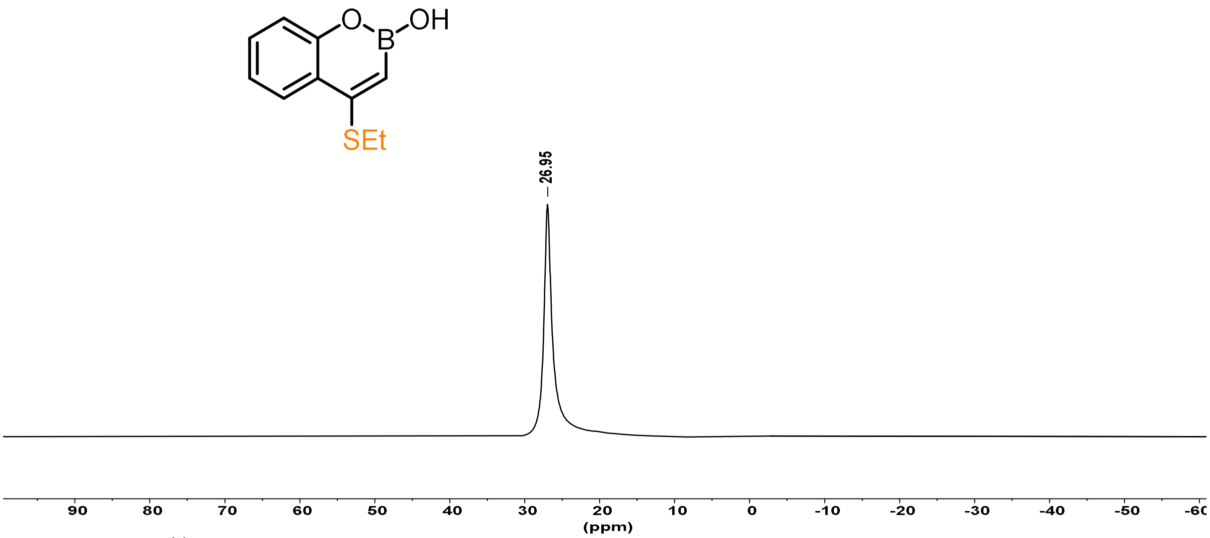

Figure S19. ^{11}B NMR spectrum of compound **2b** in acetone-D6.

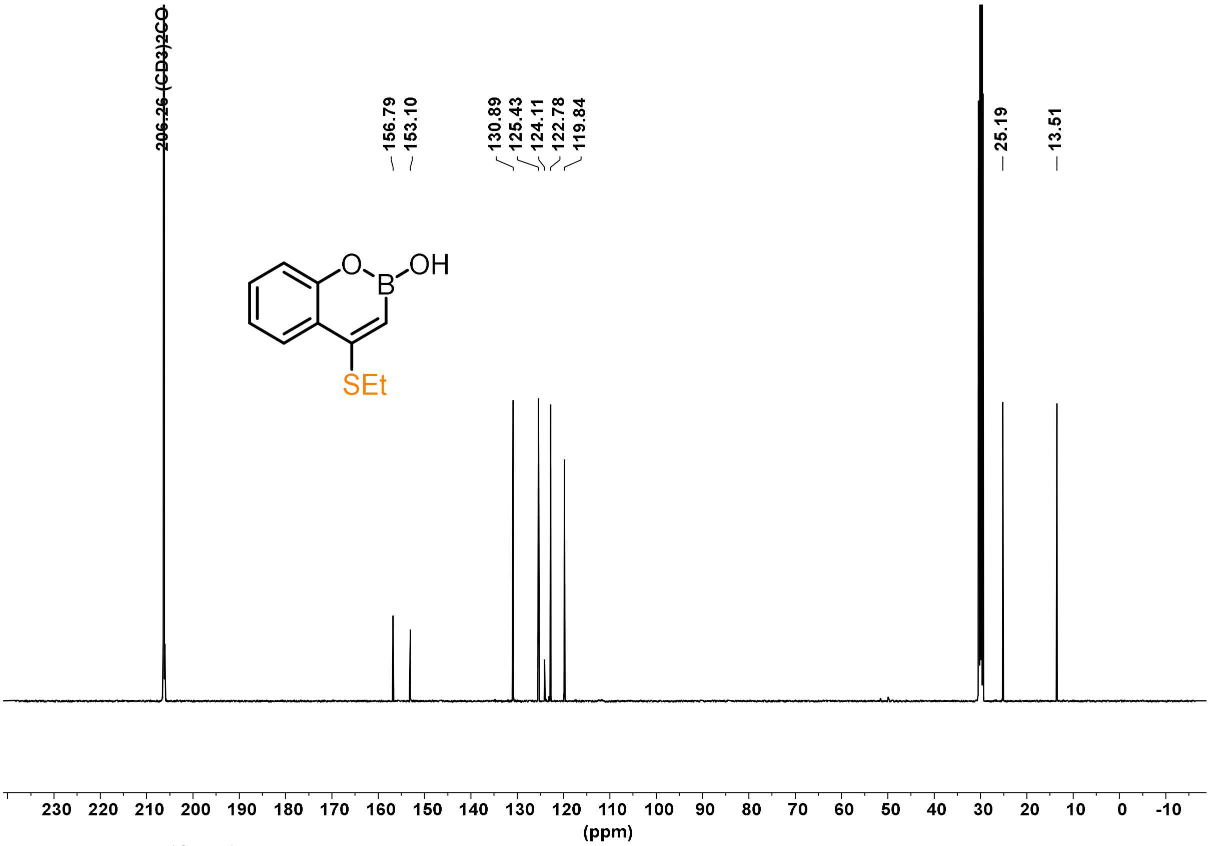

Figure S20. ^{13}C{^{1}H} NMR spectrum of compound **2b** in acetone-D6.

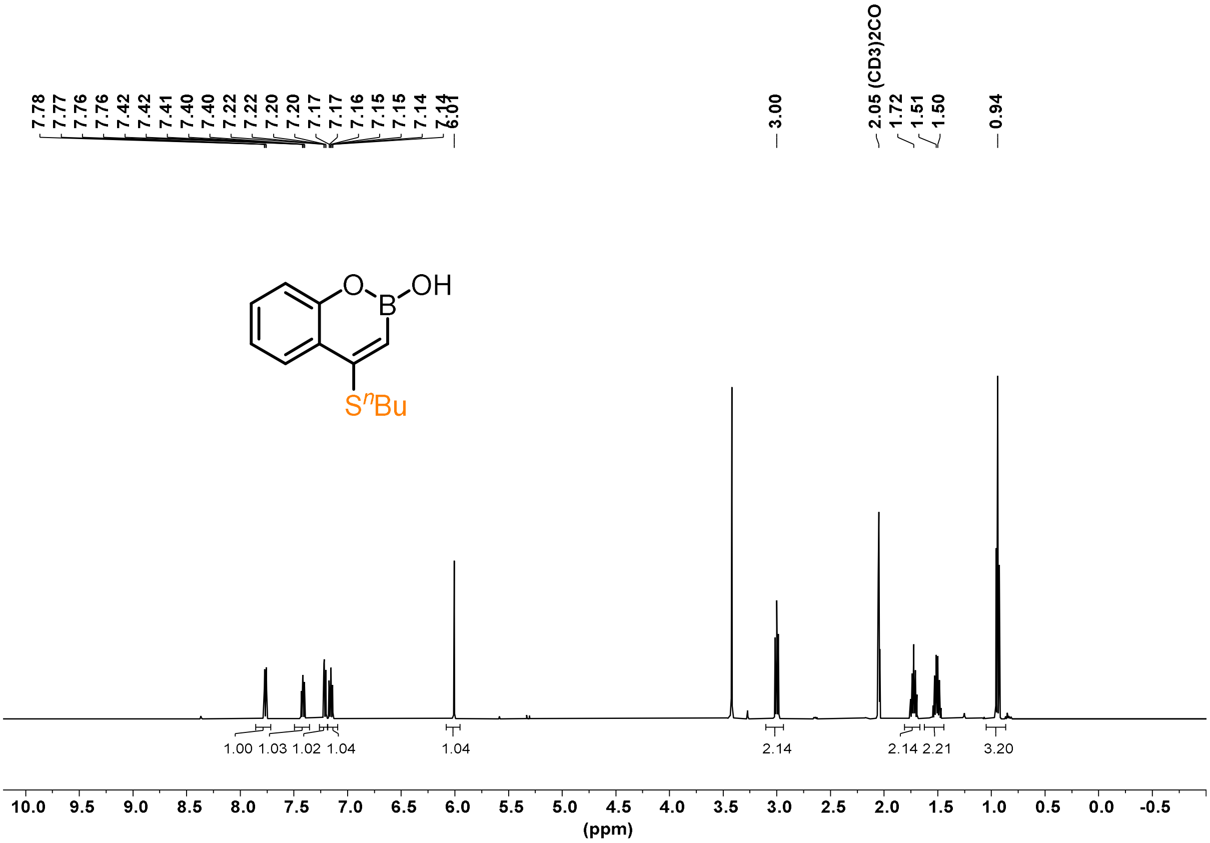

Figure S21. ^{1}H NMR spectrum of compound **2c** in acetone-D$_6$ + 40 uL D$_2$O.

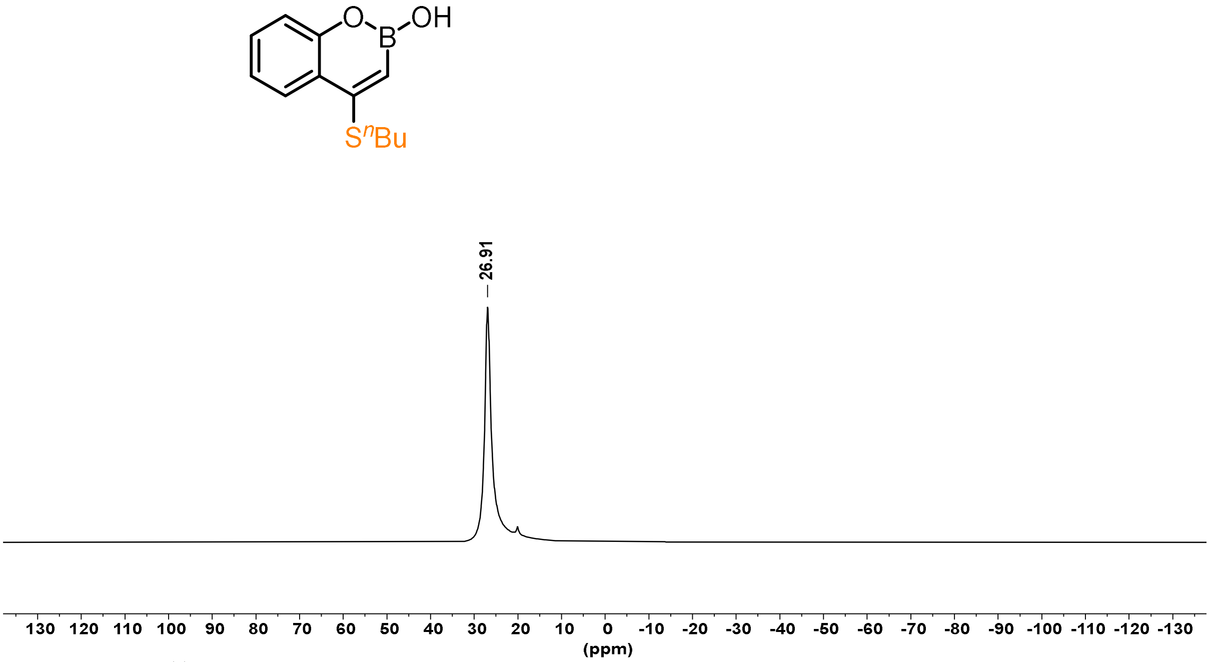

Figure S22. ^{11}B NMR spectrum of compound **2c** in acetone-D$_6$.

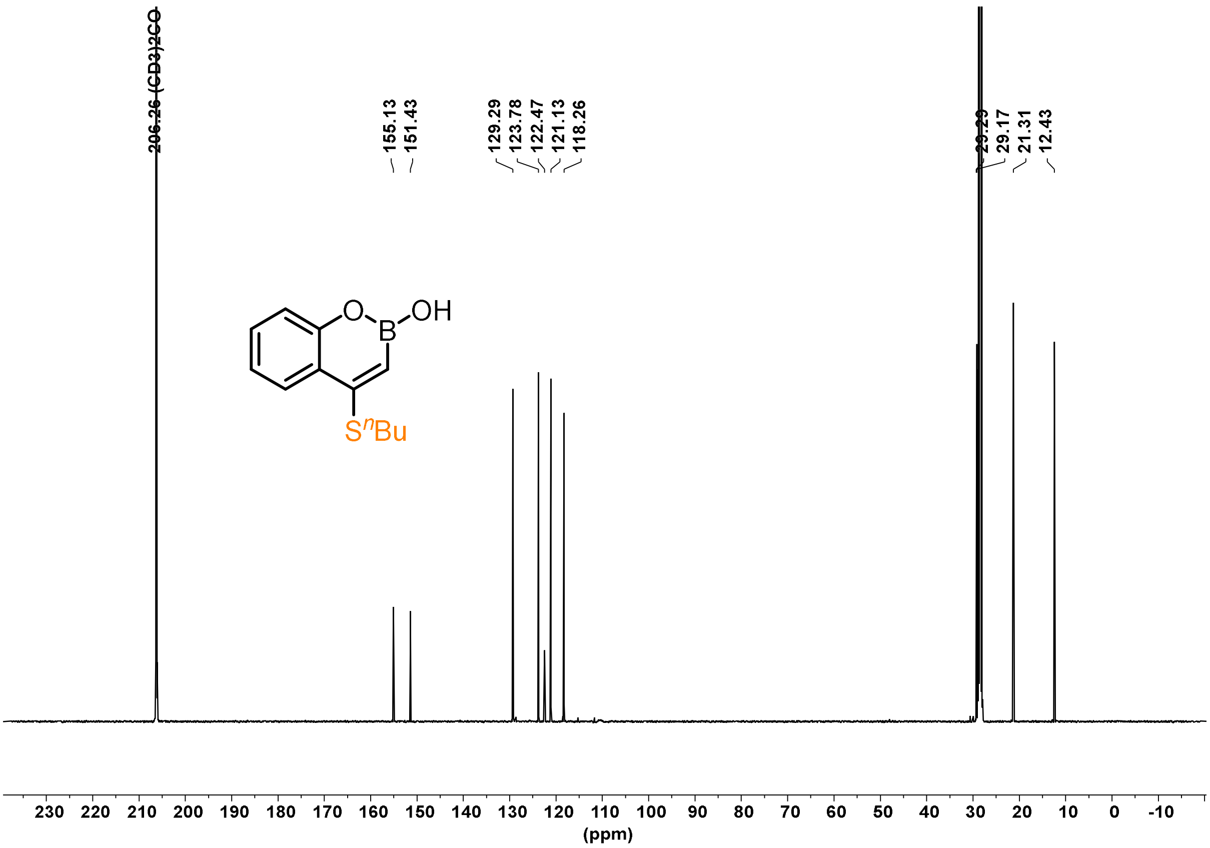

Figure S23. $^{13}C\{^{1}H\}$ NMR spectrum of compound **2c** in acetone-D_6 + 40 uL D_2O.

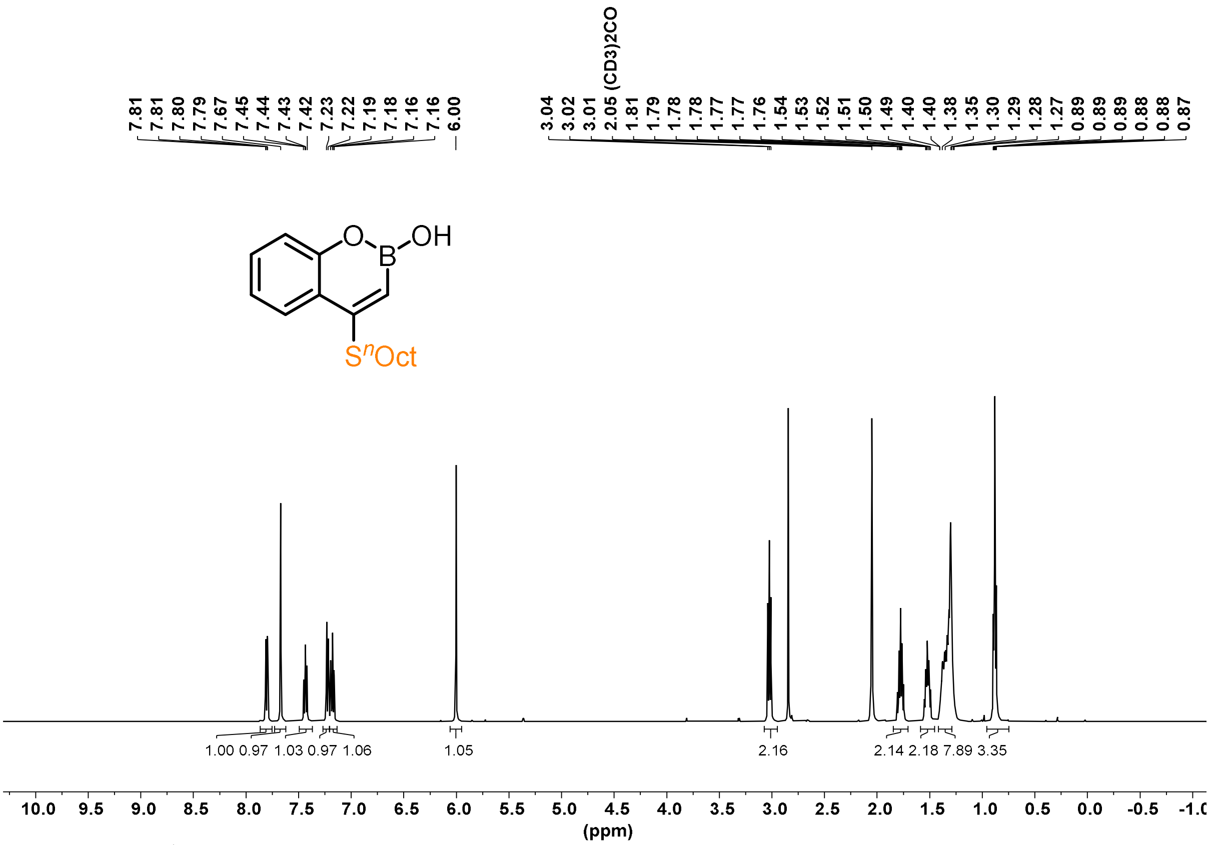

Figure S24. ^{1}H NMR spectrum of compound **2d** in acetone-D_6.

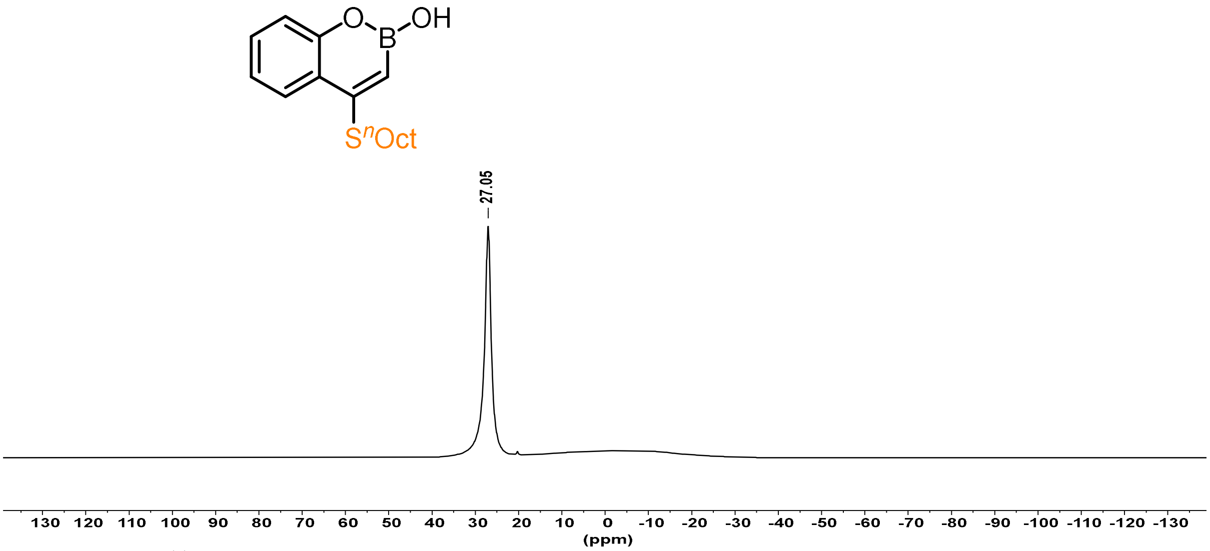

Figure S25. ^{11}B NMR spectrum of compound **2d** in acetone-D$_6$.

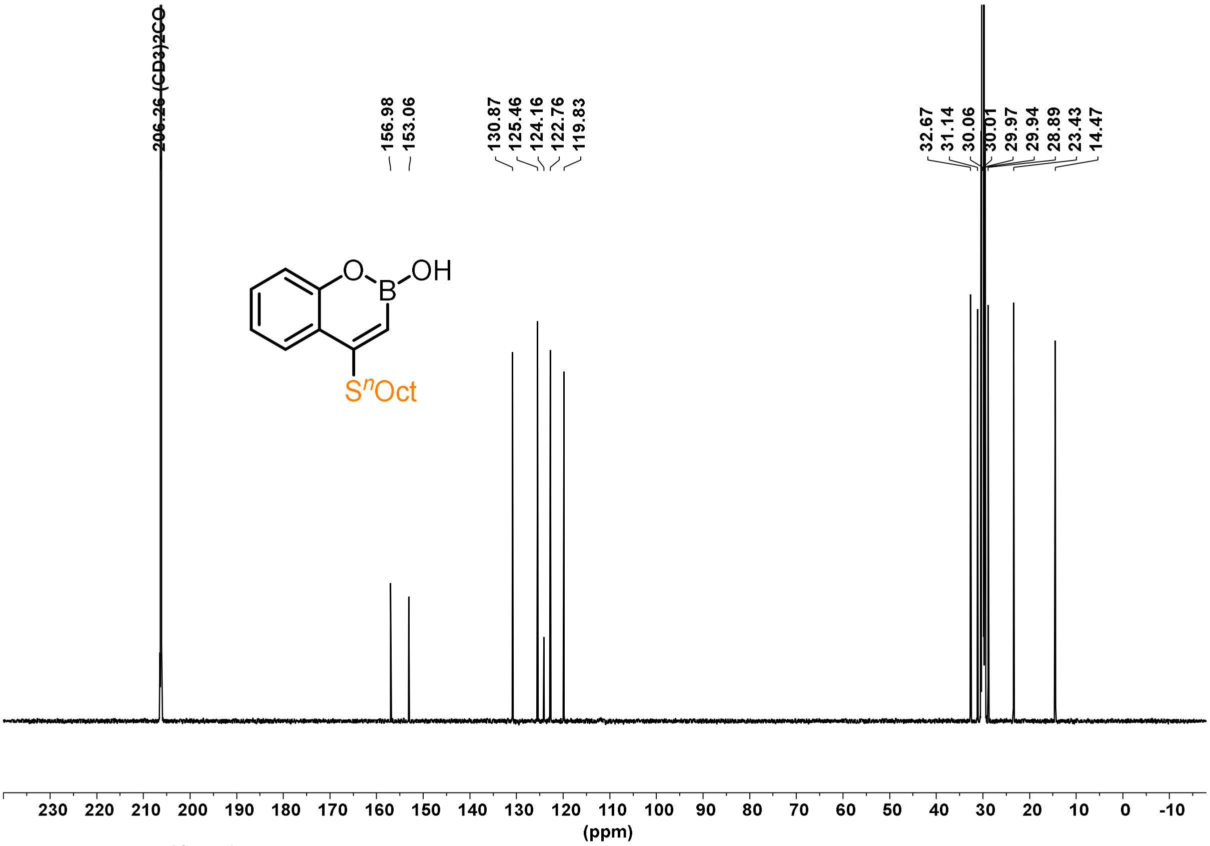

Figure S26. ^{13}C{^{1}H} NMR spectrum of compound **2d** in acetone-D$_6$.

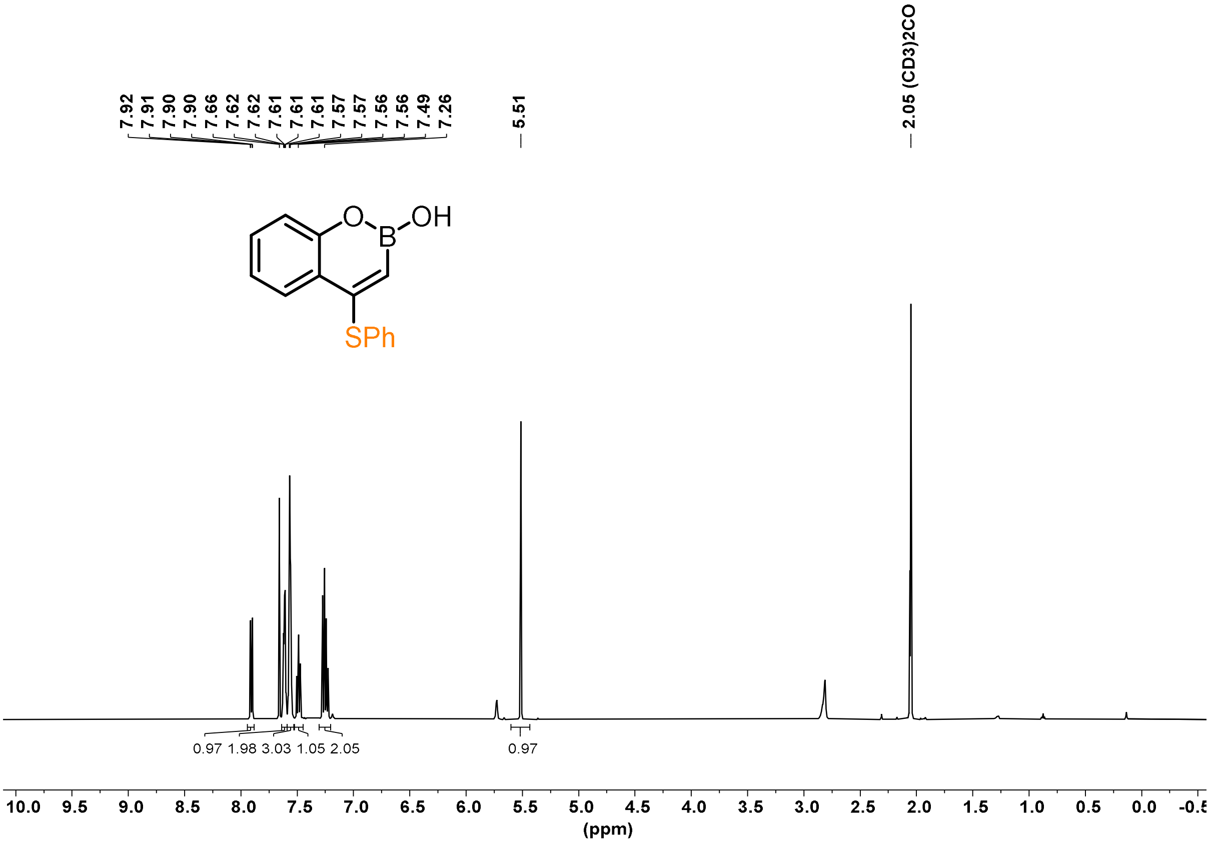

Figure S27. ^{1}H NMR spectrum of compound **2e** in acetone-D_6 + 40 uL D_2O.

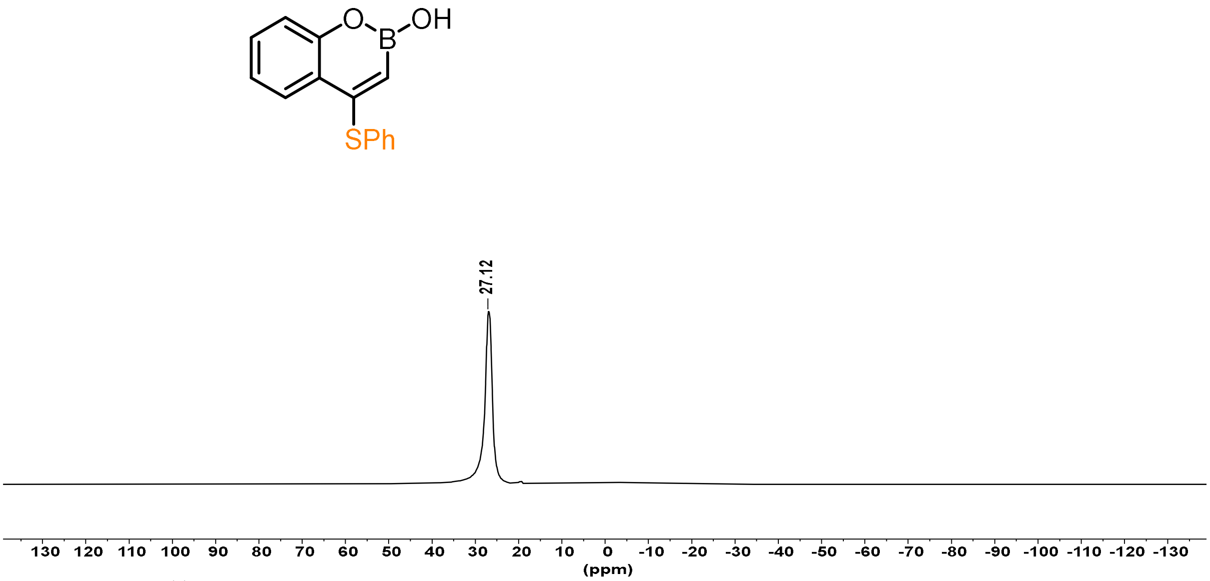

Figure S28. ^{11}B NMR spectrum of compound **2e** in acetone-D_6.

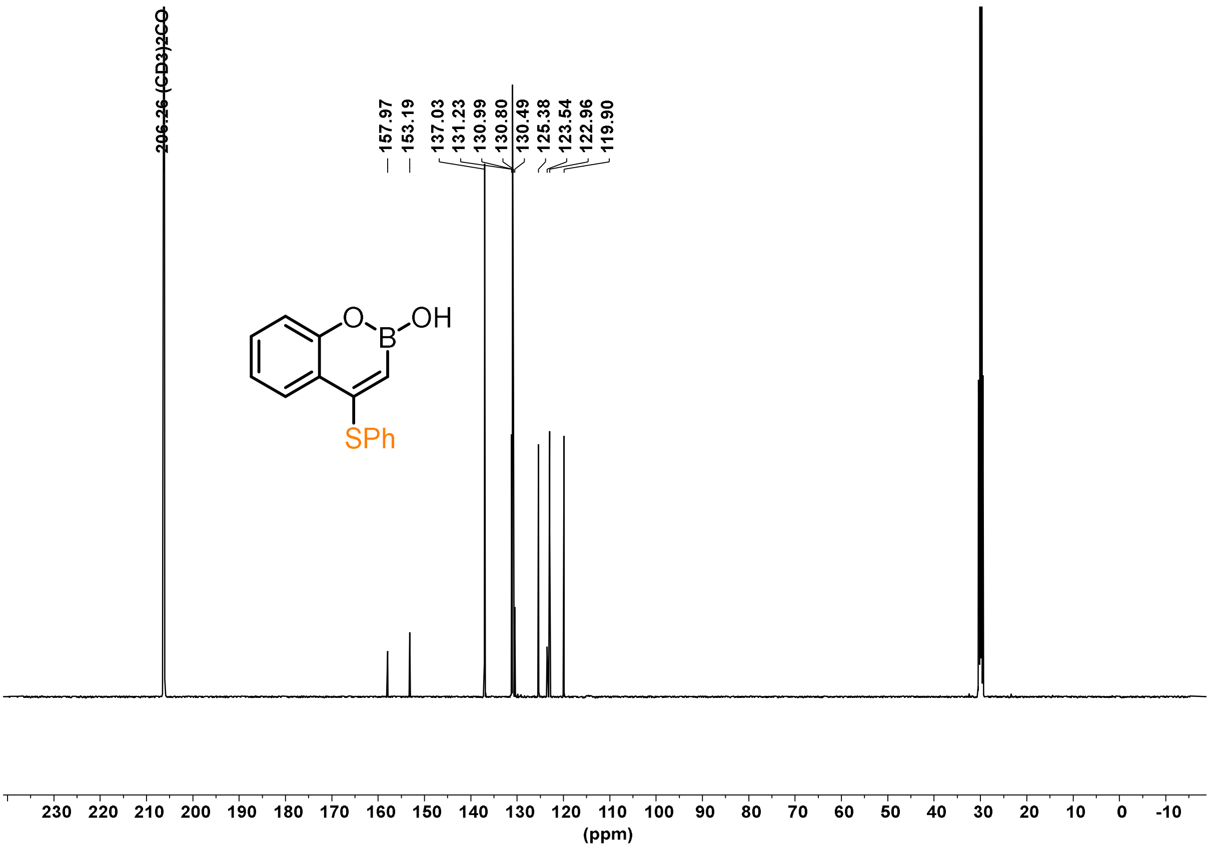

Figure S29. $^{13}C\{^{1}H\}$ NMR spectrum of compound **2e** in acetone-D_6 + 40 uL D_2O.

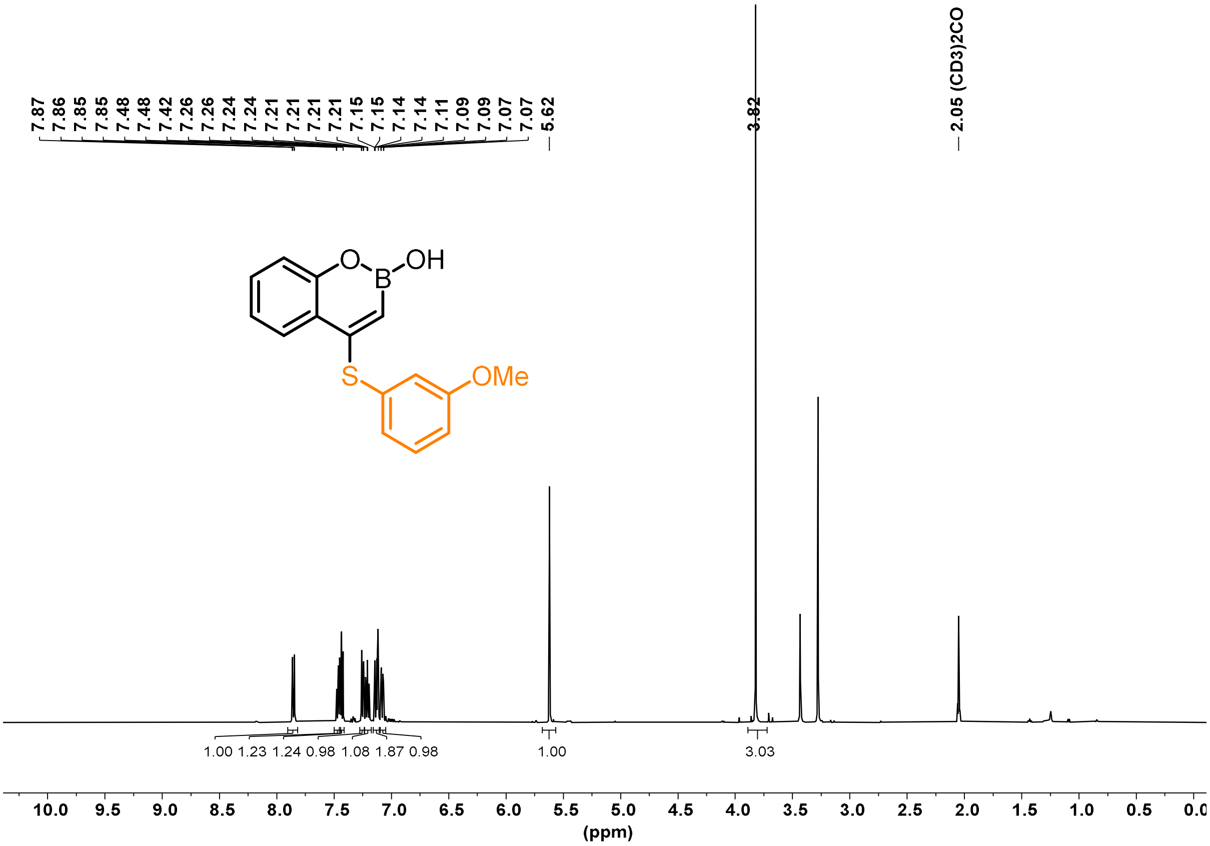

Figure S30. ^{1}H NMR spectrum of compound **2f** in acetone-D_6 + 40 uL D_2O.

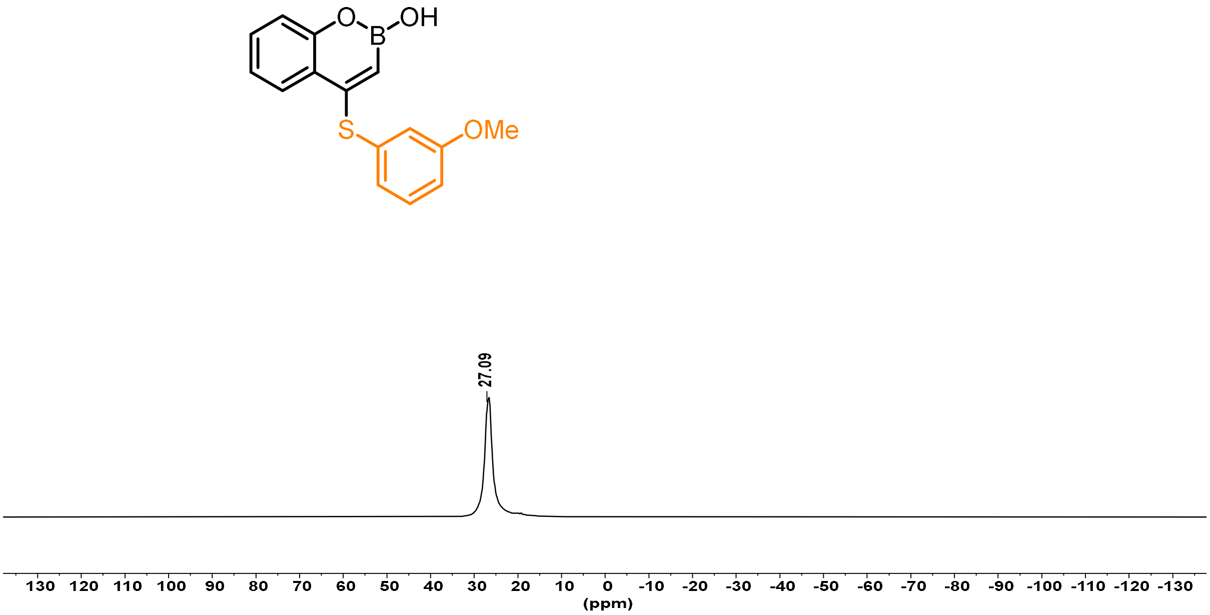

Figure S31. ^{11}B NMR spectrum of compound **2f** in acetone-D$_6$.

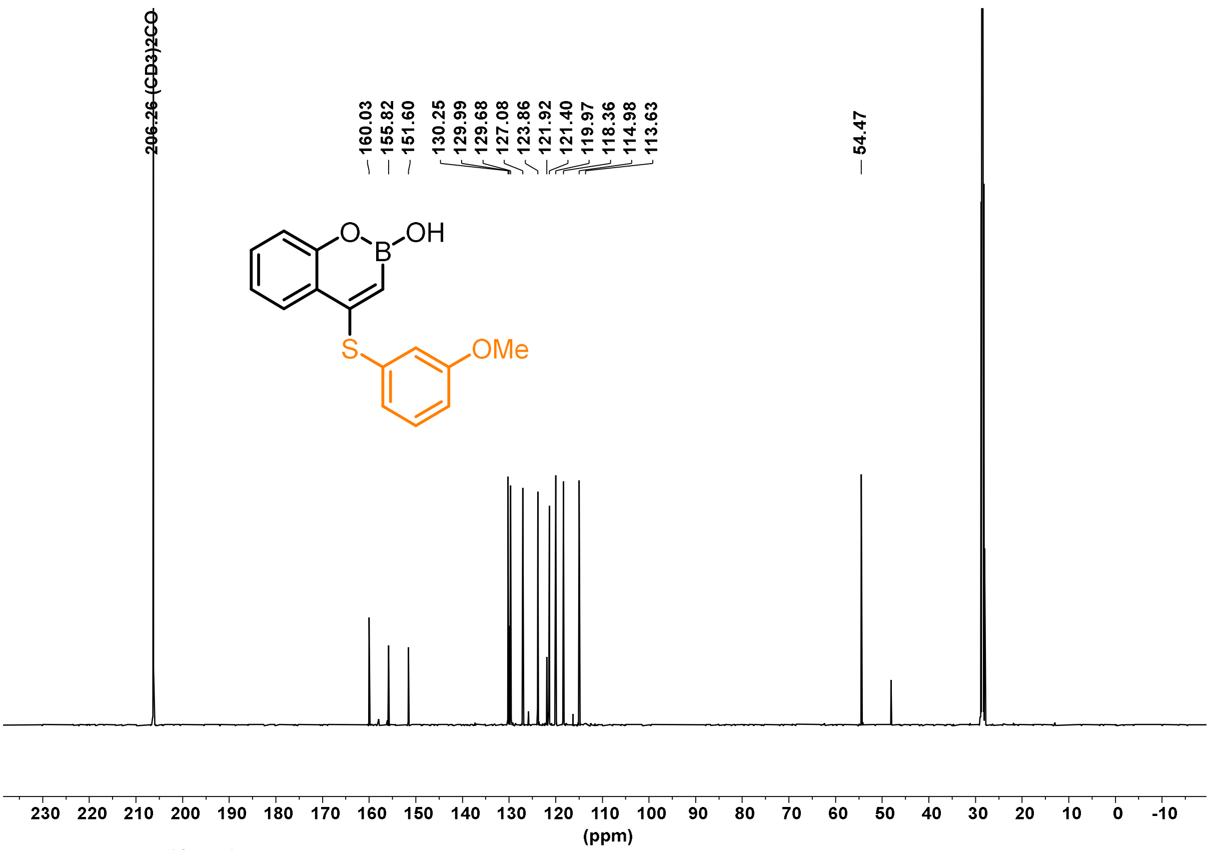

Figure S32. $^{13}C\{^{1}H\}$ NMR spectrum of compound **2f** in acetone-D$_6$ + 40 uL D_2O.

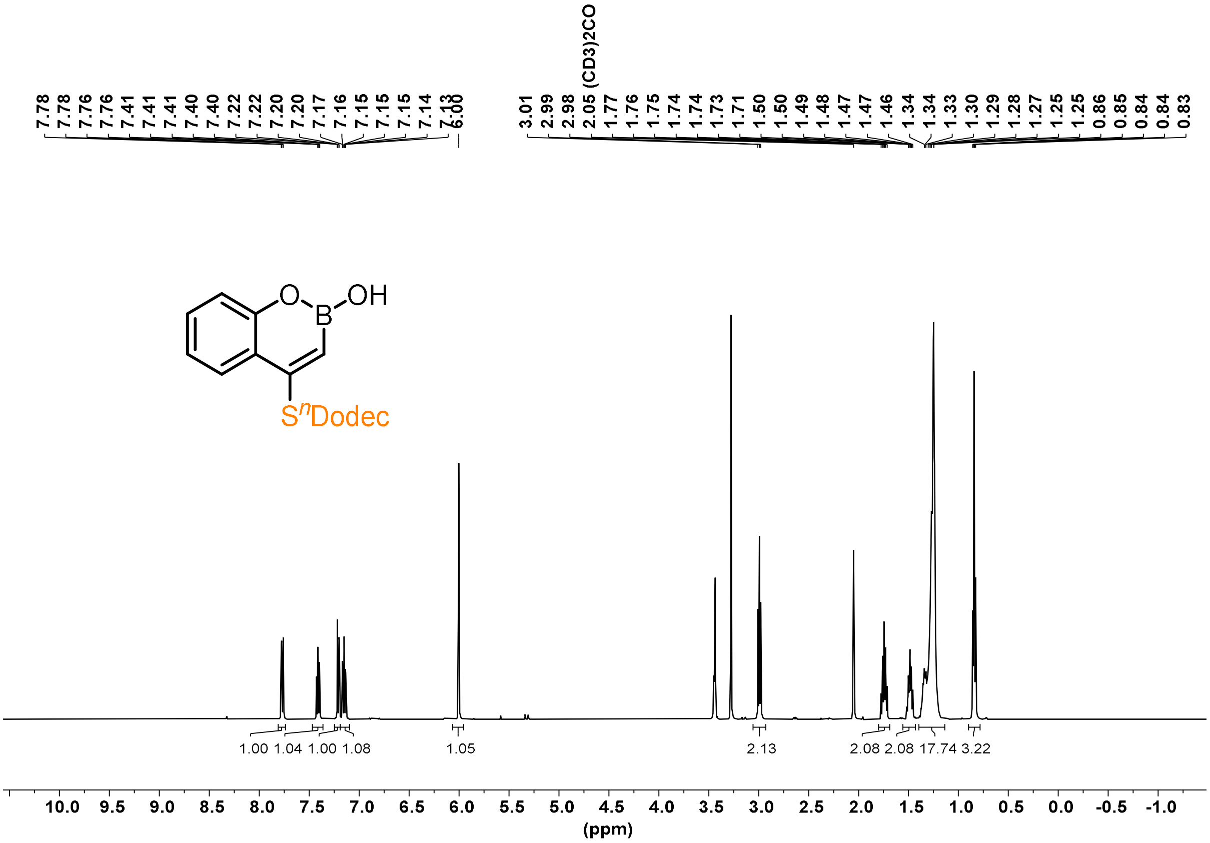

Figure S33. ^{1}H NMR spectrum of compound **2g** in acetone-D_6 + 40 uL D_2O.

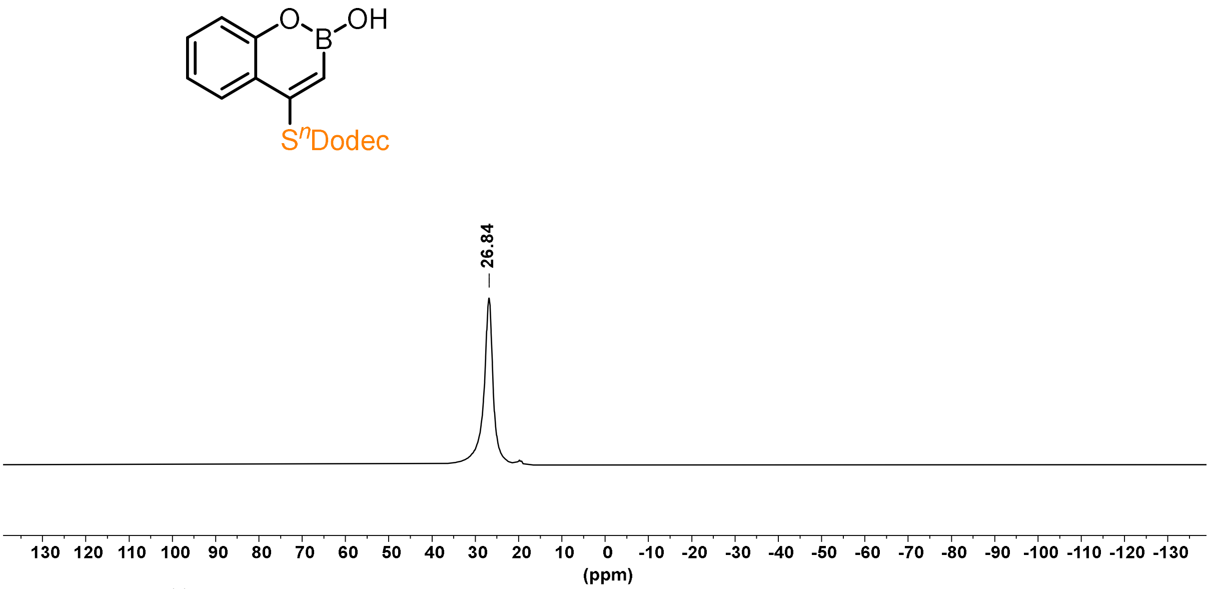

Figure S34. ^{11}B NMR spectrum of compound **2g** in acetone-D_6.

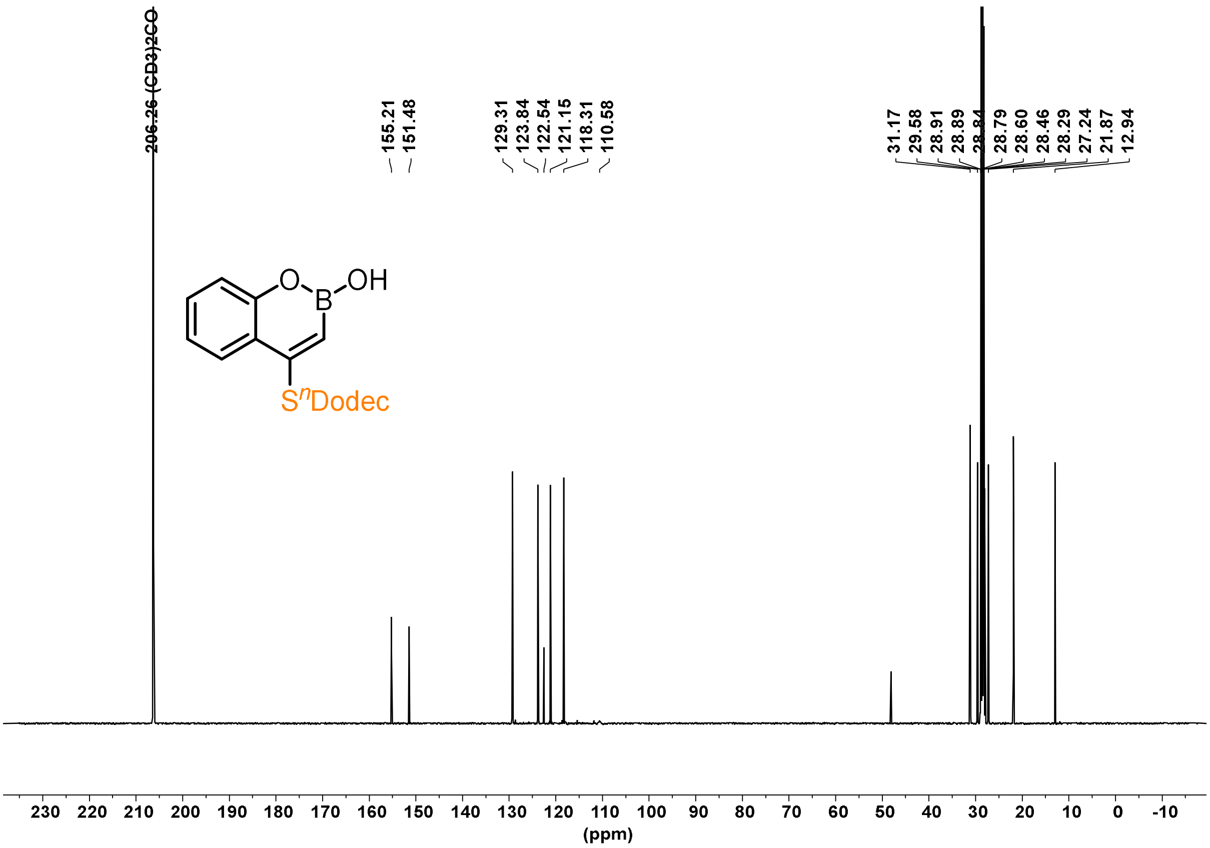

Figure S35. $^{13}C\{^1H\}$ NMR spectrum of compound **2g** in acetone-D$_6$ + 40 uL D_2O.

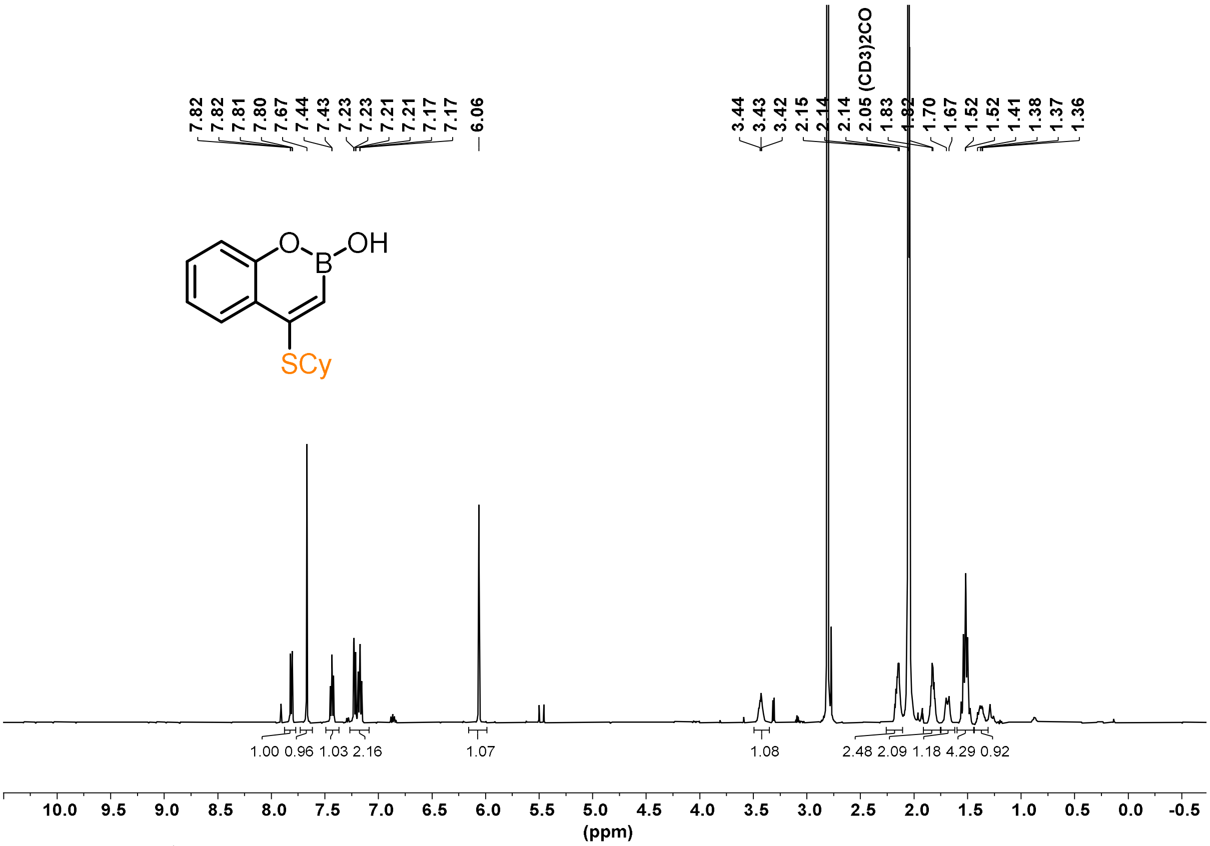

Figure S36. ^1H NMR spectrum of compound **2h** in acetone-D$_6$ + 40 uL D_2O.

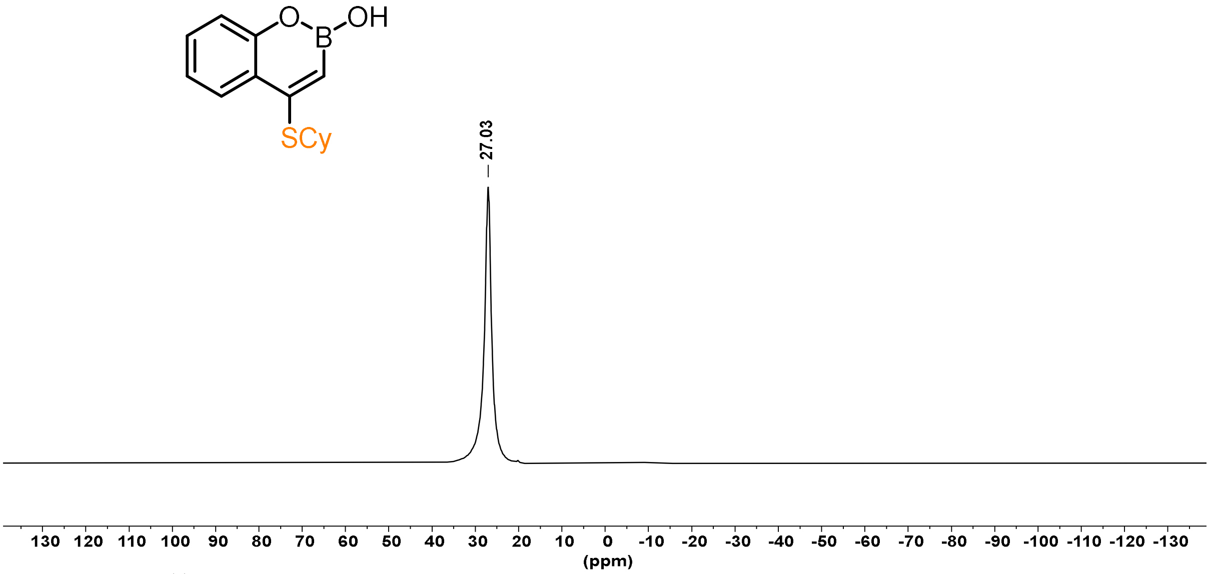

Figure S37. ^{11}B NMR spectrum of compound **2h** in acetone-D_6.

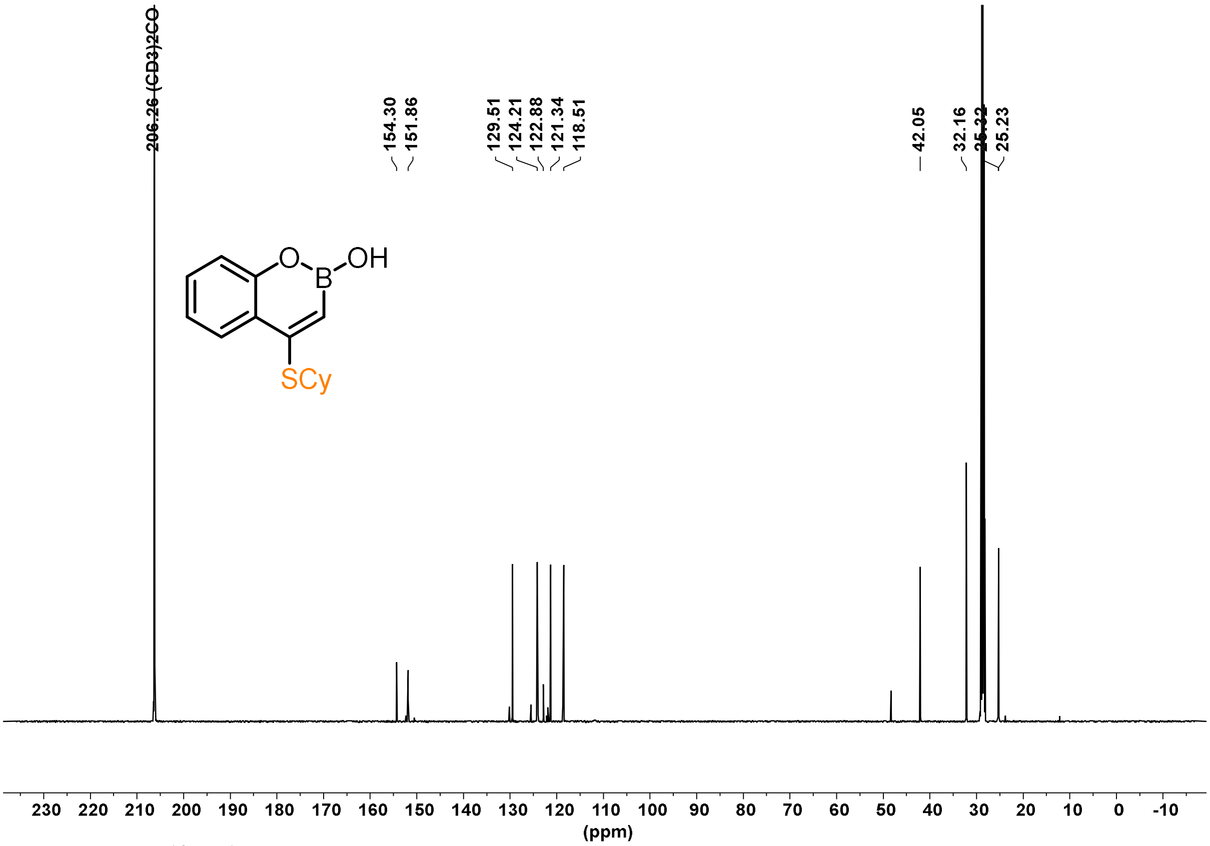

Figure S38. $^{13}C\{^1H\}$ NMR spectrum of compound **2h** in acetone-D_6 + 40 uL D_2O.

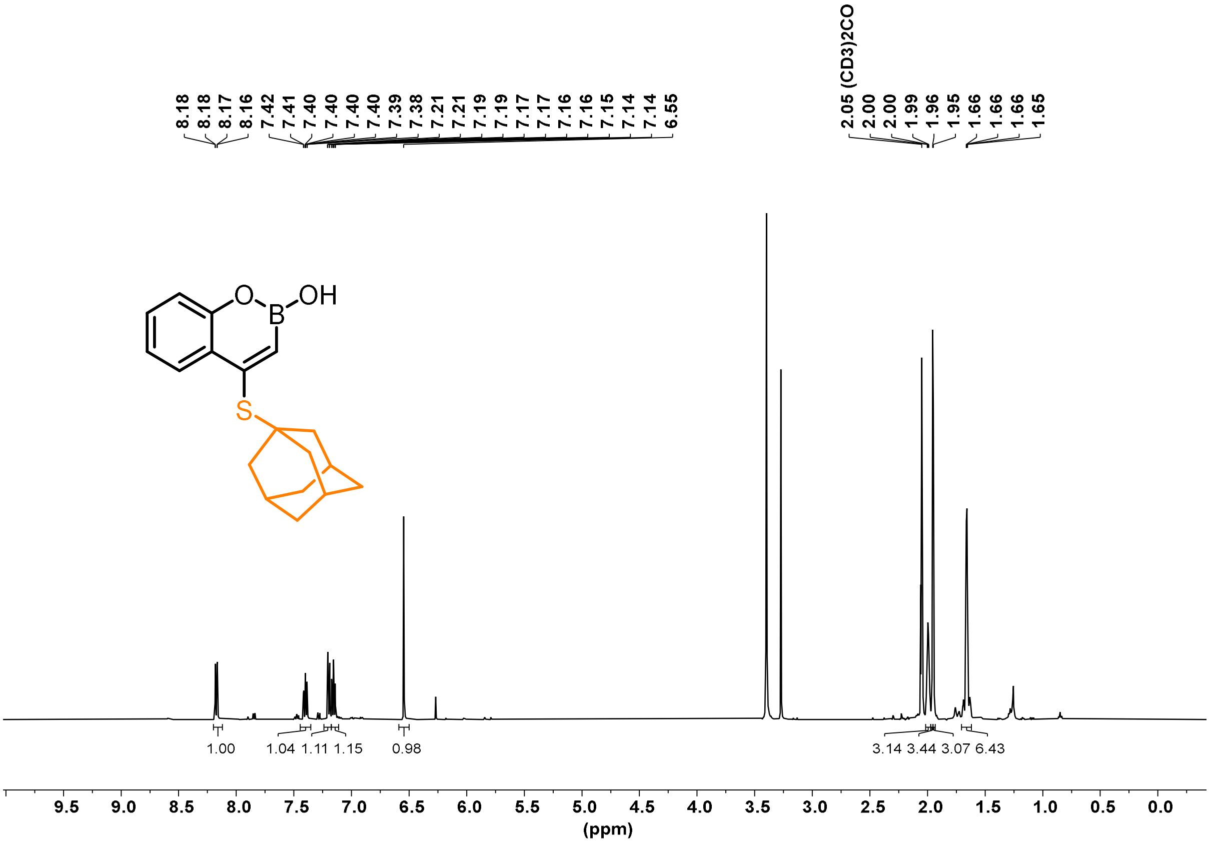

Figure S39. ^{1}H NMR spectrum of compound **2i** in acetone-D_6 + 40 uL D_2O.

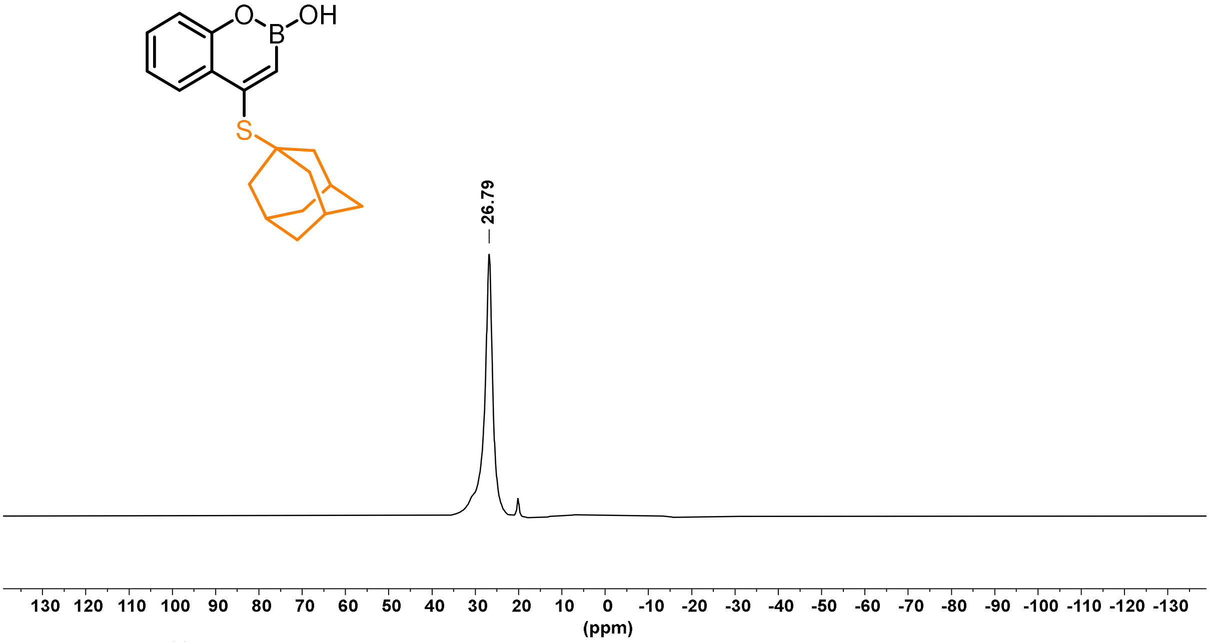

Figure S40. ^{11}B NMR spectrum of compound **2i** in acetone-D_6.

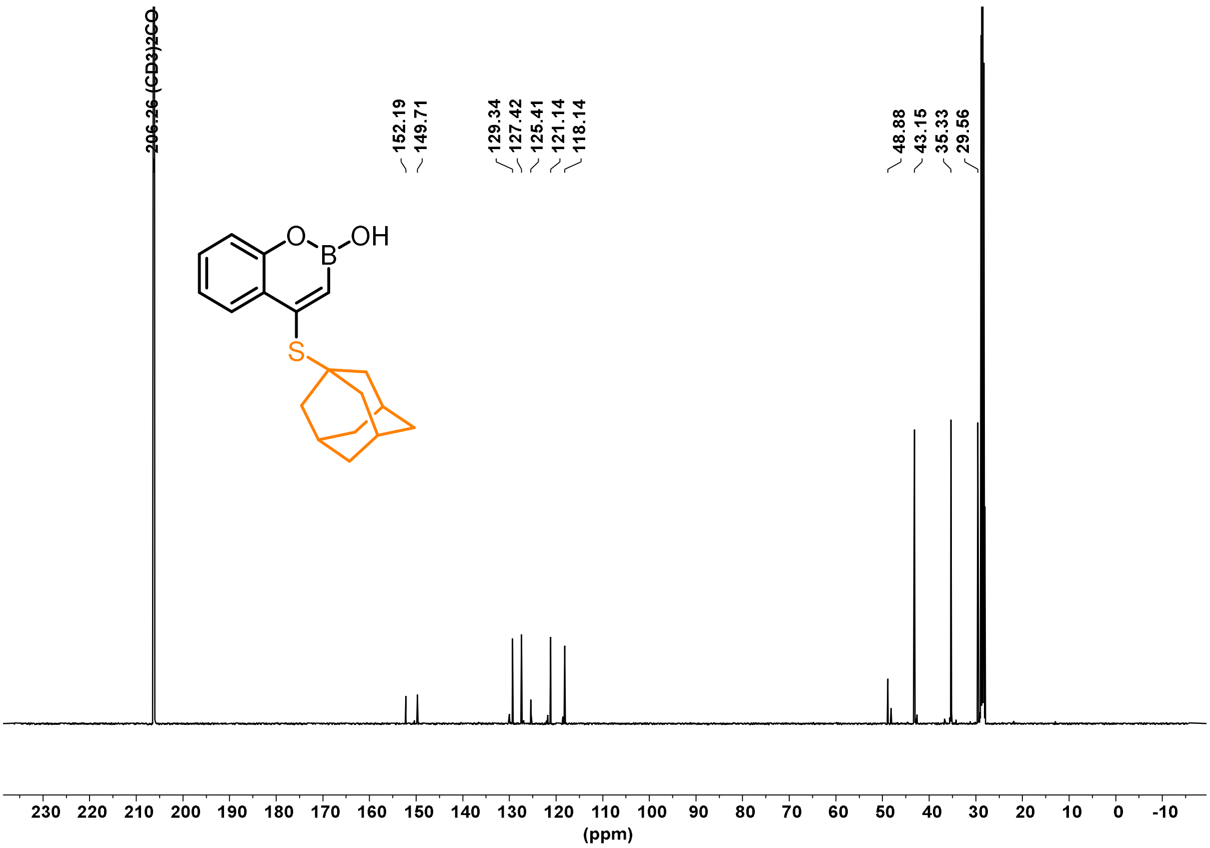

Figure S41. $^{13}C\{^{1}H\}$ NMR spectrum of compound **2i** in acetone-D6 + 40 uL D_2O.

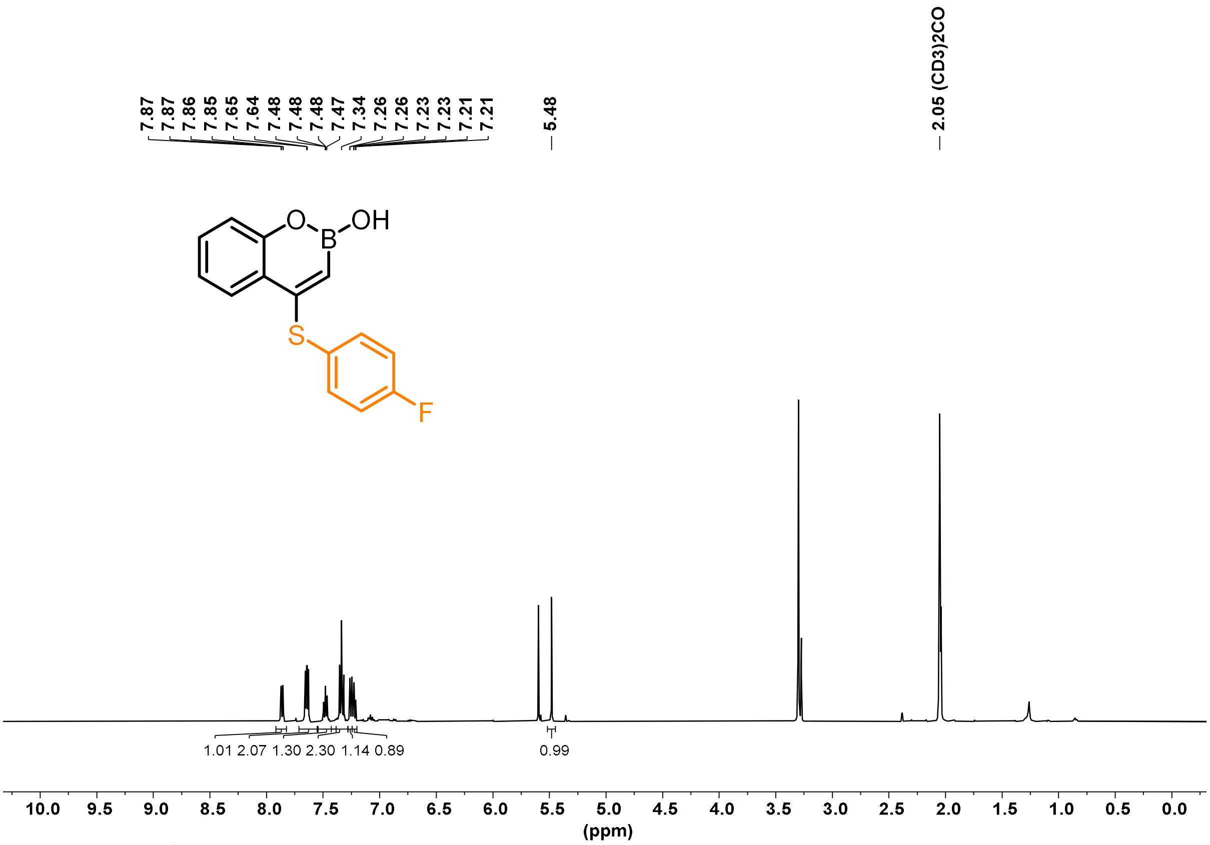

Figure S42. ^{1}H NMR spectrum of compound **2j** in acetone-D6 + 40 uL D_2O.

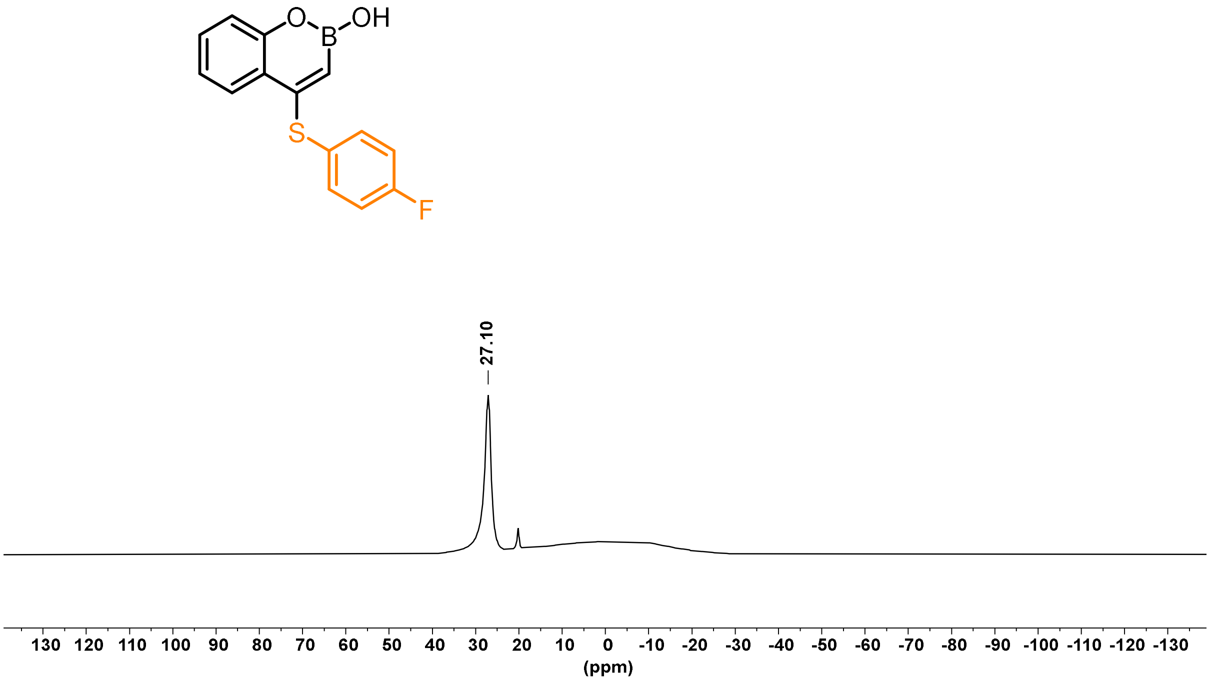

Figure S43. ^{11}B NMR spectrum of compound **2j** in acetone-D6.

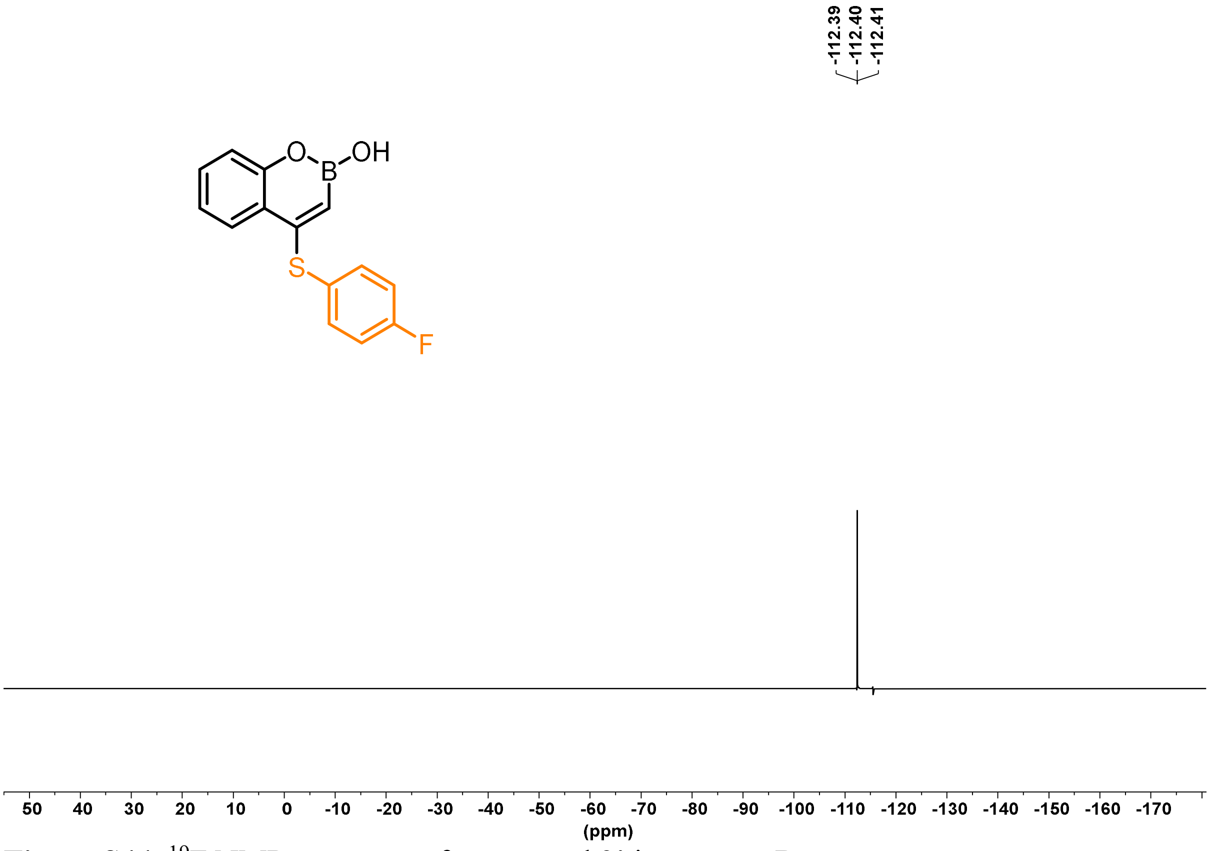

Figure S44. ^{19}F NMR spectrum of compound **2j** in acetone-D6.

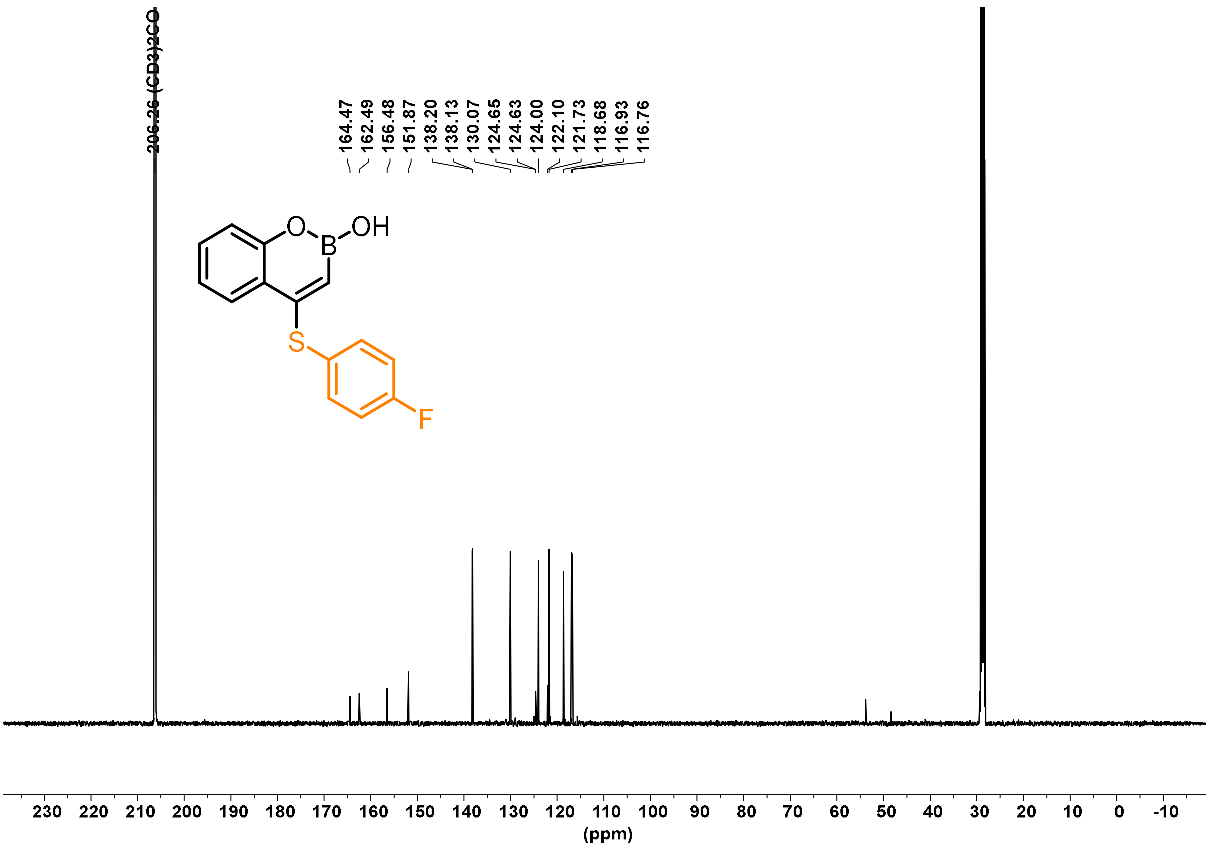

Figure S45. $^{13}C\{^{1}H\}$ NMR spectrum of compound **2j** in acetone-D_6 + 40 uL D_2O.

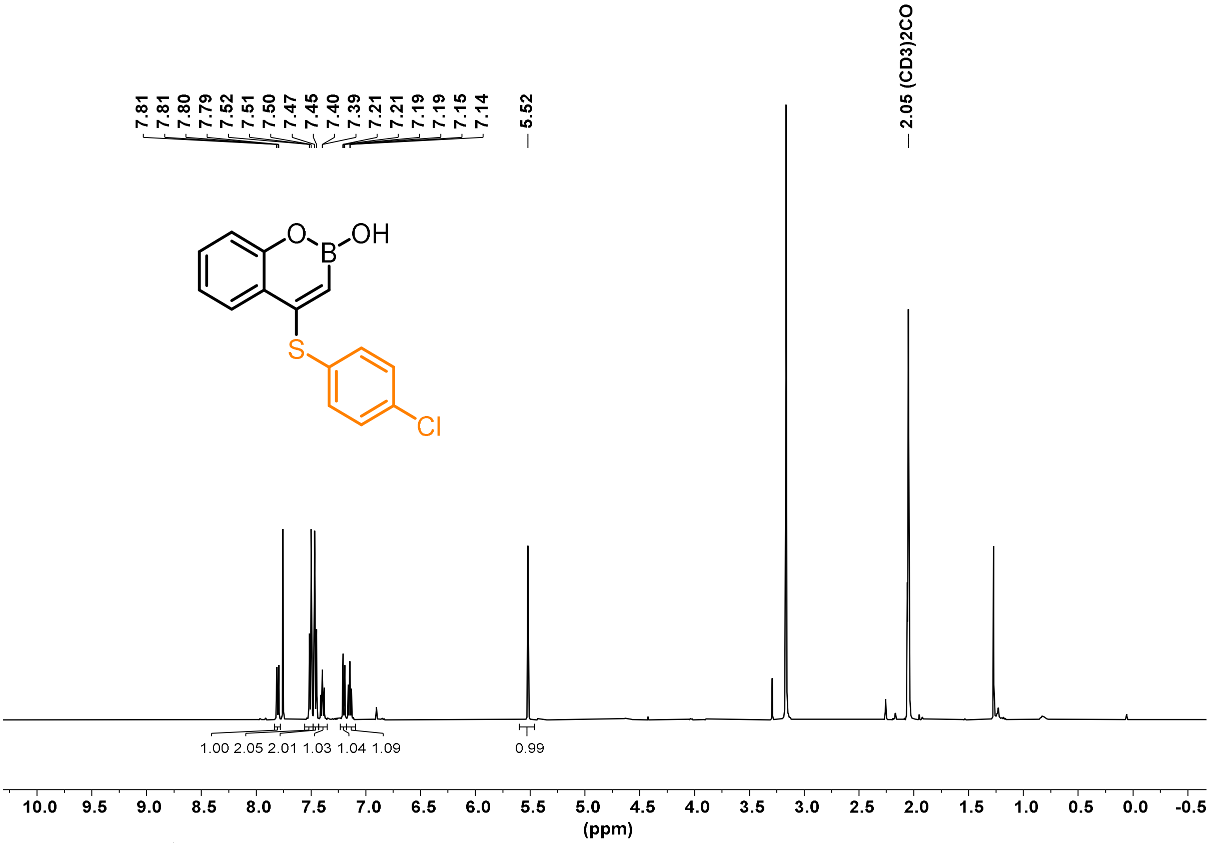

Figure S46. ^{1}H NMR spectrum of compound **2k** in acetone-D_6.

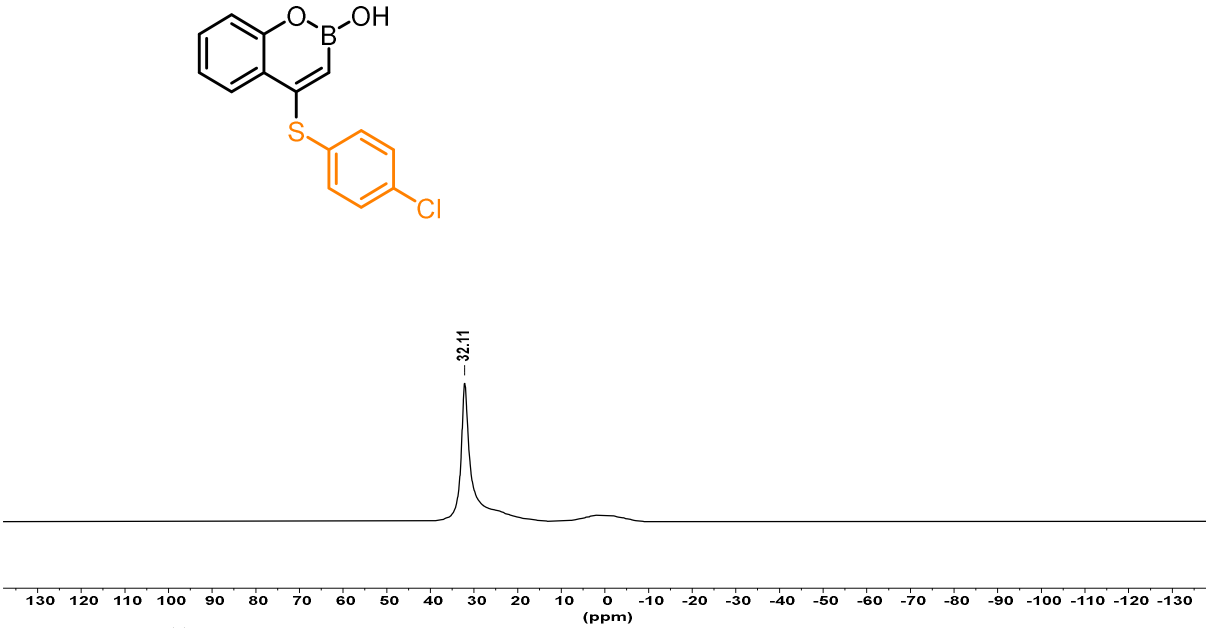

Figure S47. ^{11}B NMR spectrum of compound **2k** in acetone-D$_6$.

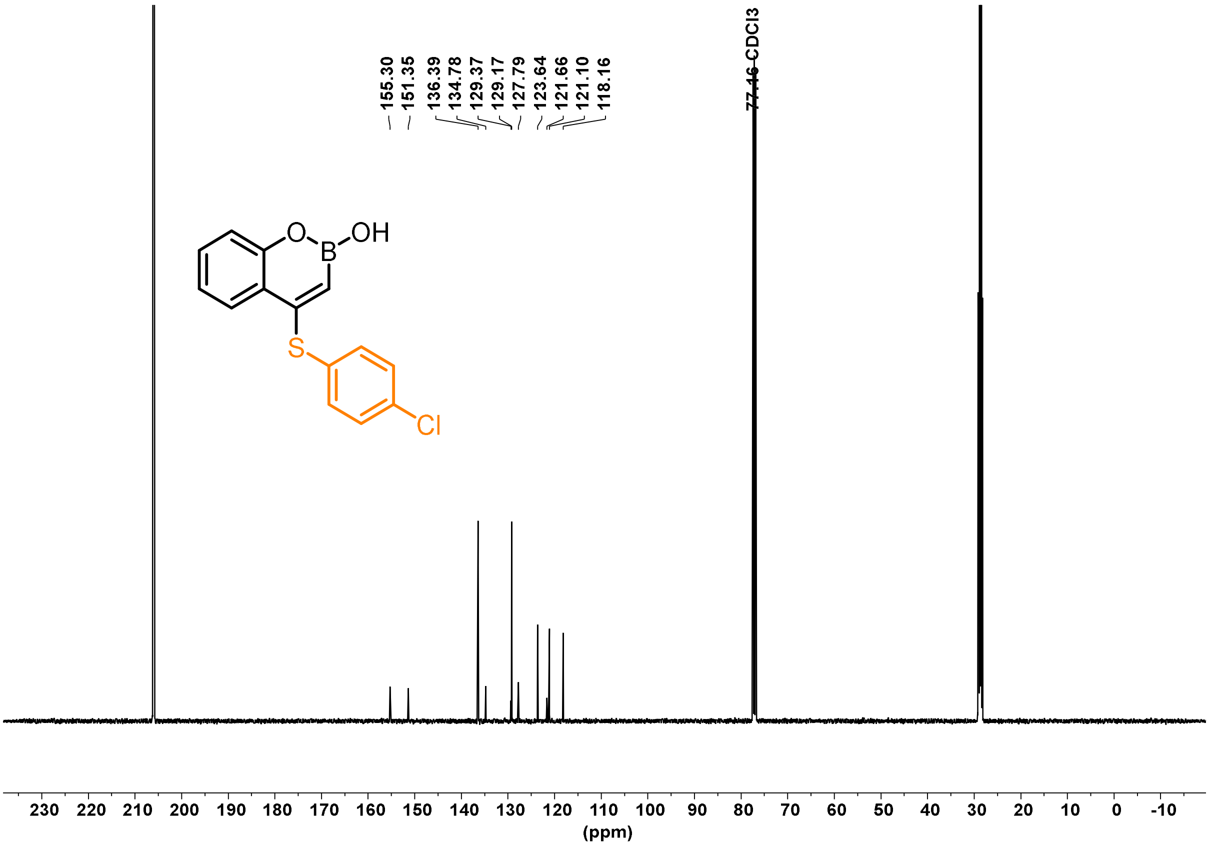

Figure S48. ^{13}C{^{1}H} NMR spectrum of compound **2k** in acetone-D$_6$.

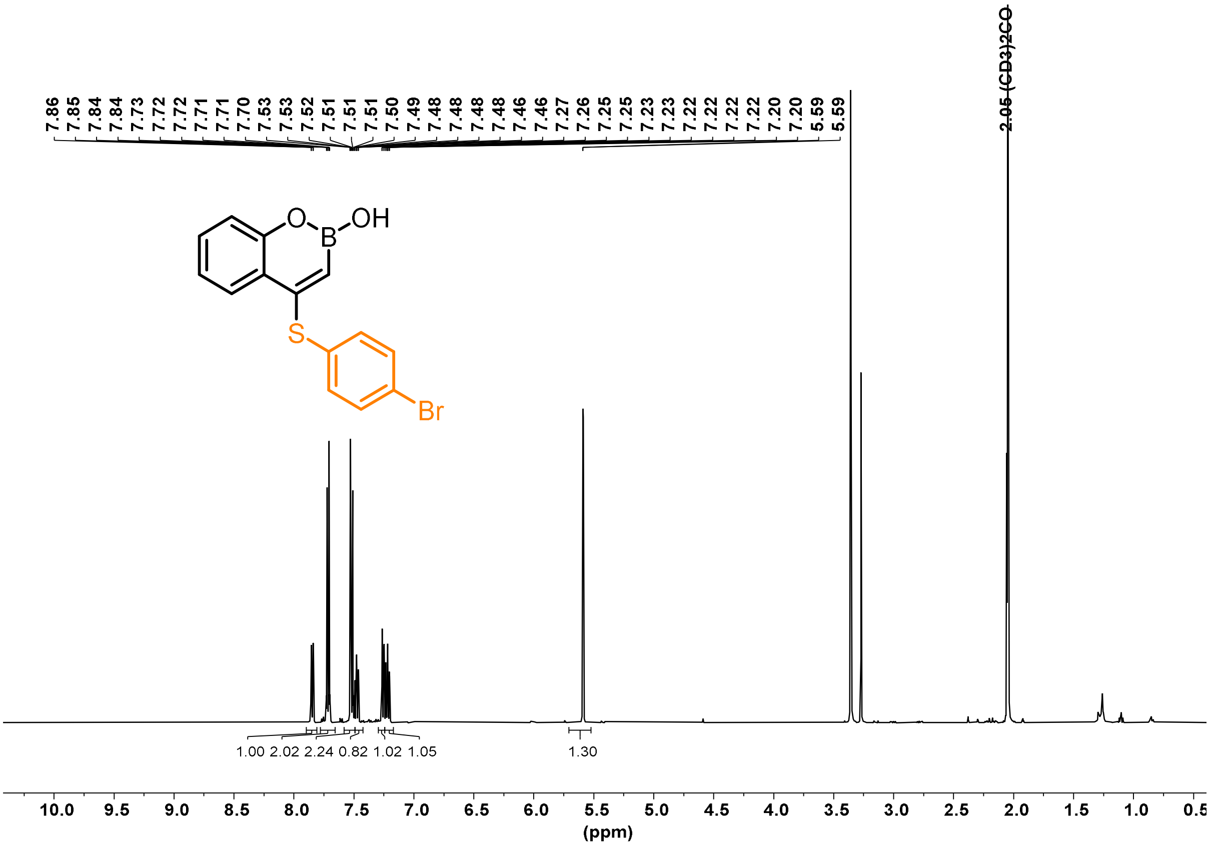

Figure S49. ^{1}H NMR spectrum of compound **2l** in acetone-D_6 + 40 uL D_2O.

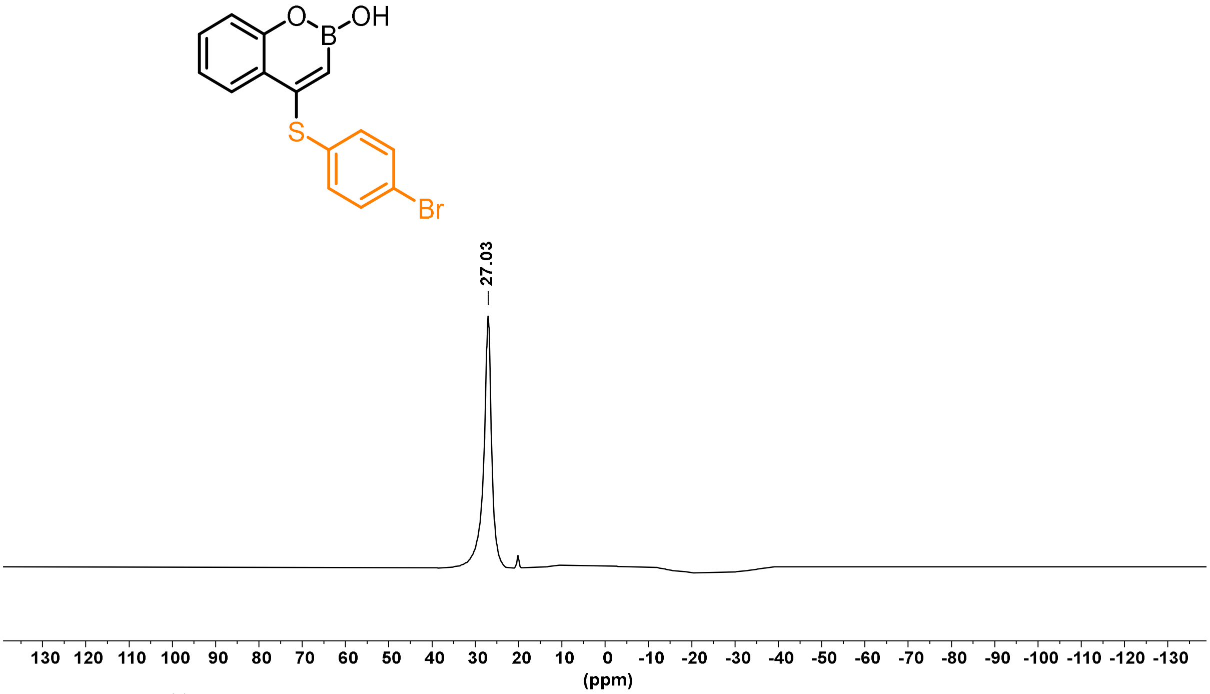

Figure S50. ^{11}B NMR spectrum of compound **2l** in acetone-D_6.

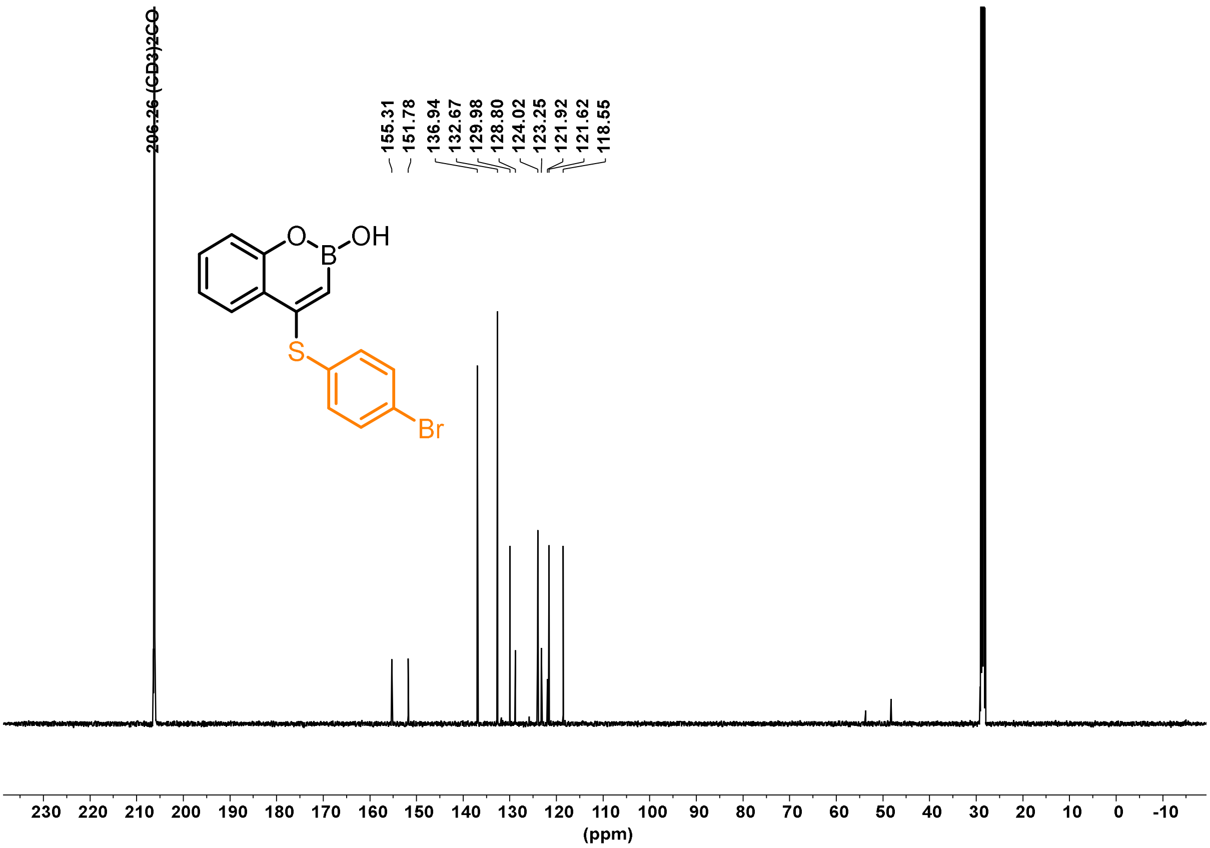

Figure S51. $^{13}C\{^{1}H\}$ NMR spectrum of compound **2l** in acetone-D_6 + 40 uL D_2O.

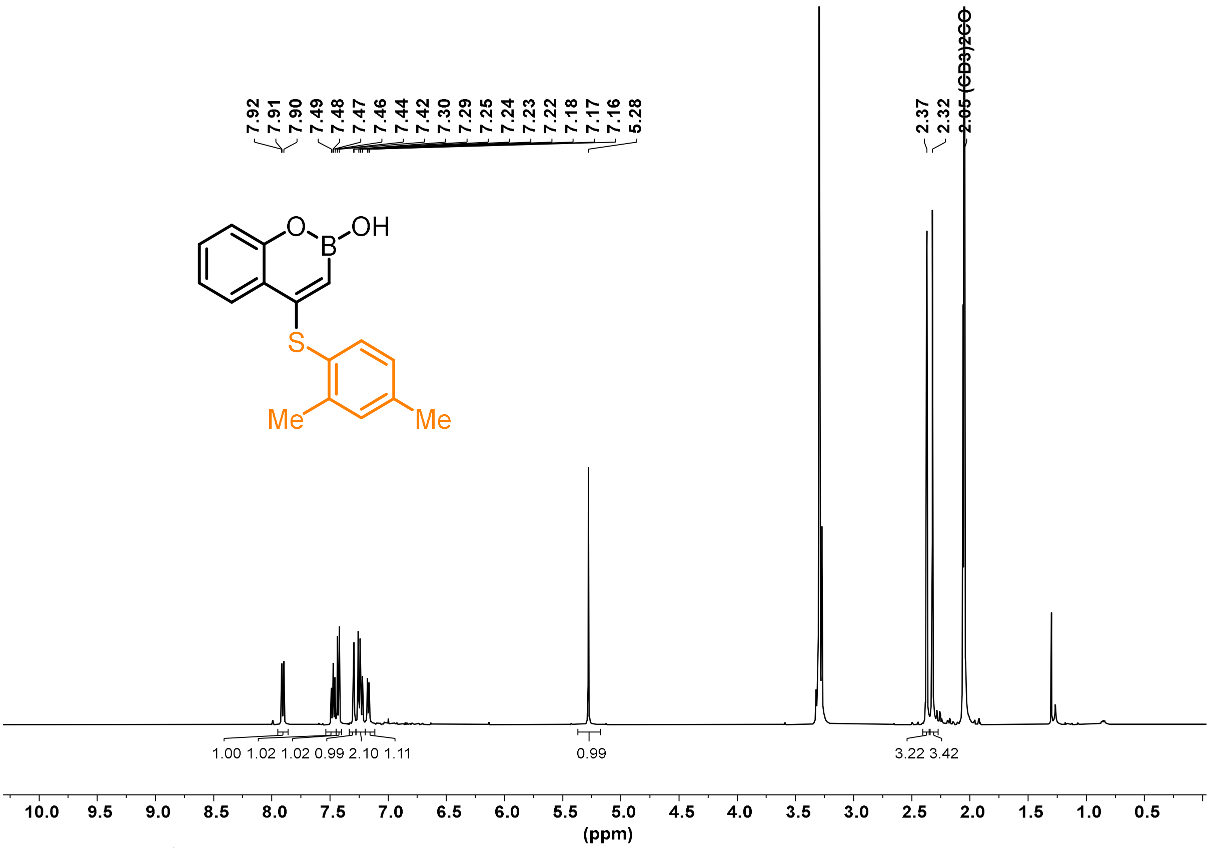

Figure S52. ^{1}H NMR spectrum of compound **2m** in acetone-D_6 + 40 uL D_2O.

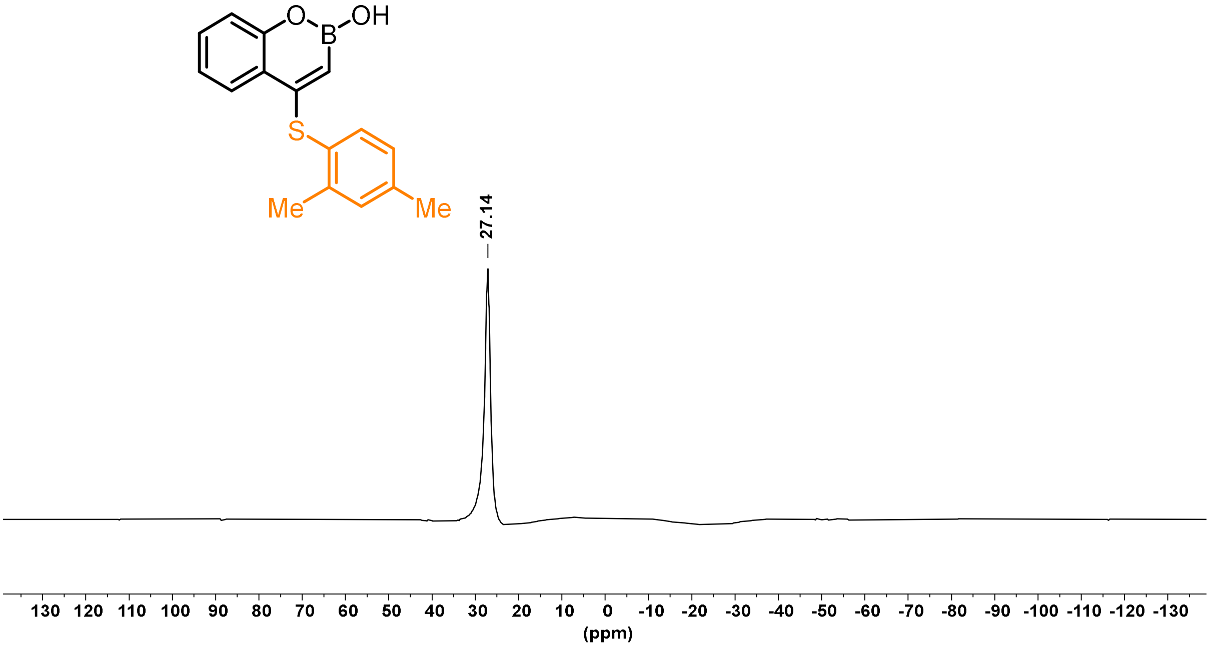

Figure S53. ^{11}B NMR spectrum of compound **2m** in acetone-D_6.

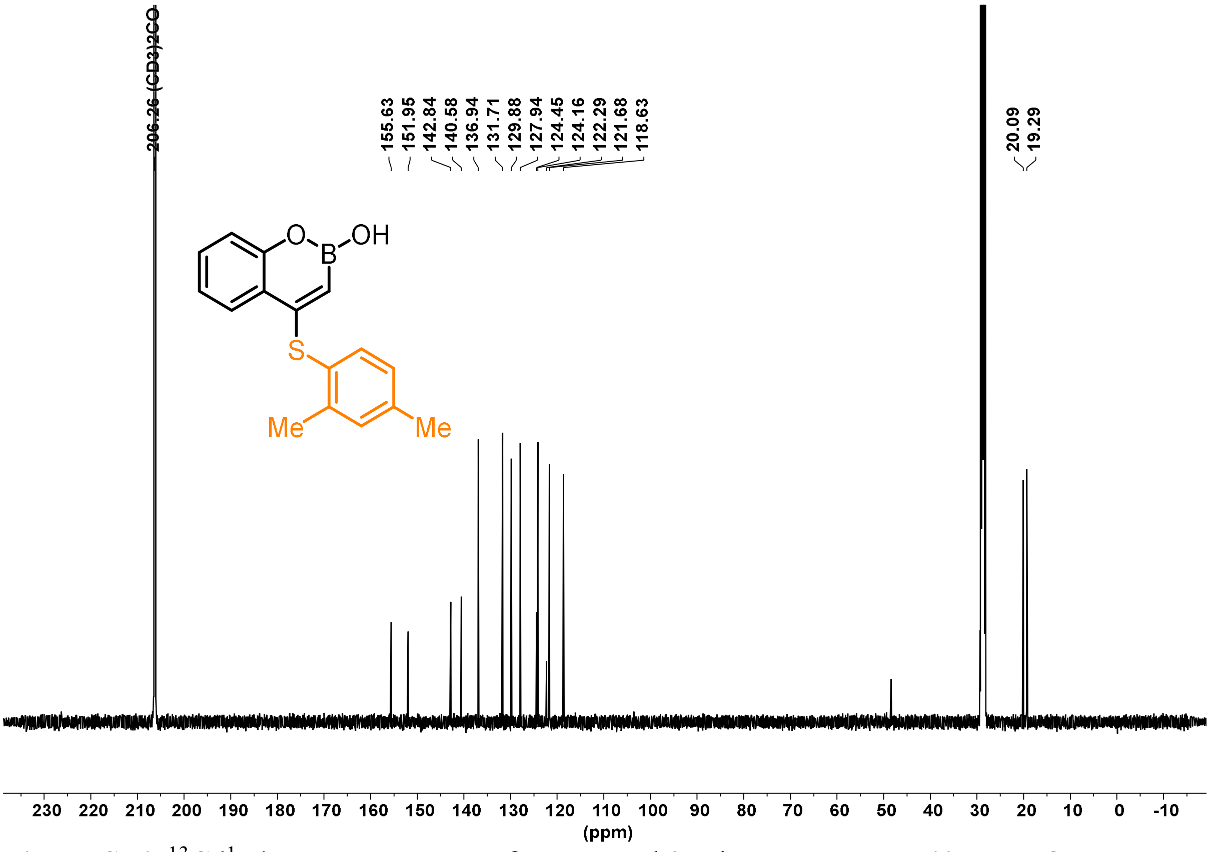

Figure S54. $^{13}C\{^1H\}$ NMR spectrum of compound **2m** in acetone-D_6 + 40 uL D_2O.

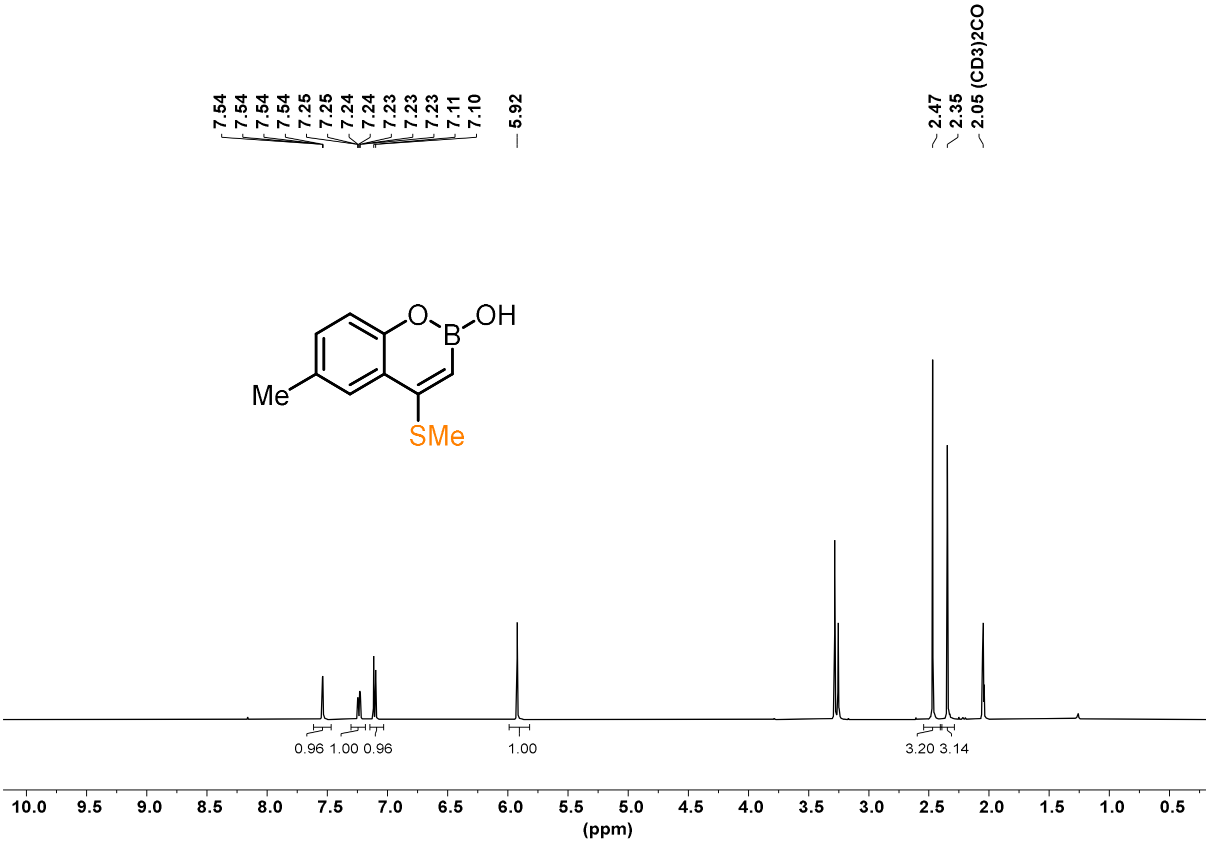

Figure S55. ^{1}H NMR spectrum of compound **2n** in acetone-D_6 + 40 uL D_2O.

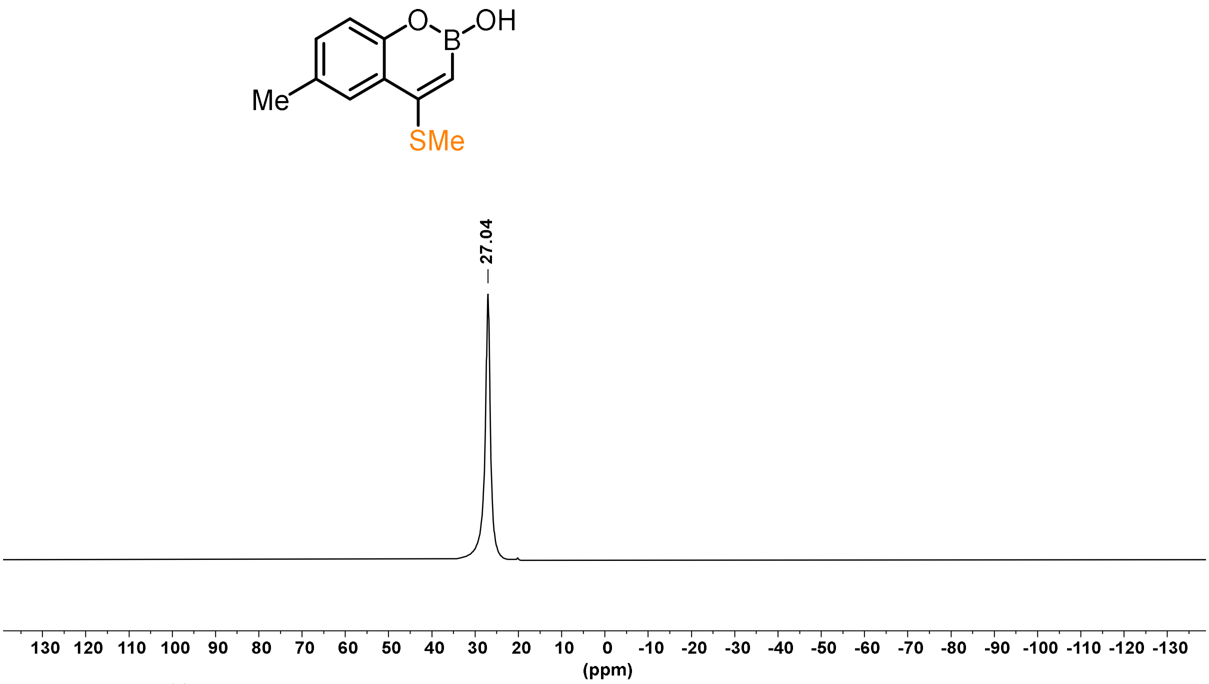

Figure S56. ^{11}B NMR spectrum of compound **2n** in acetone-D_6.

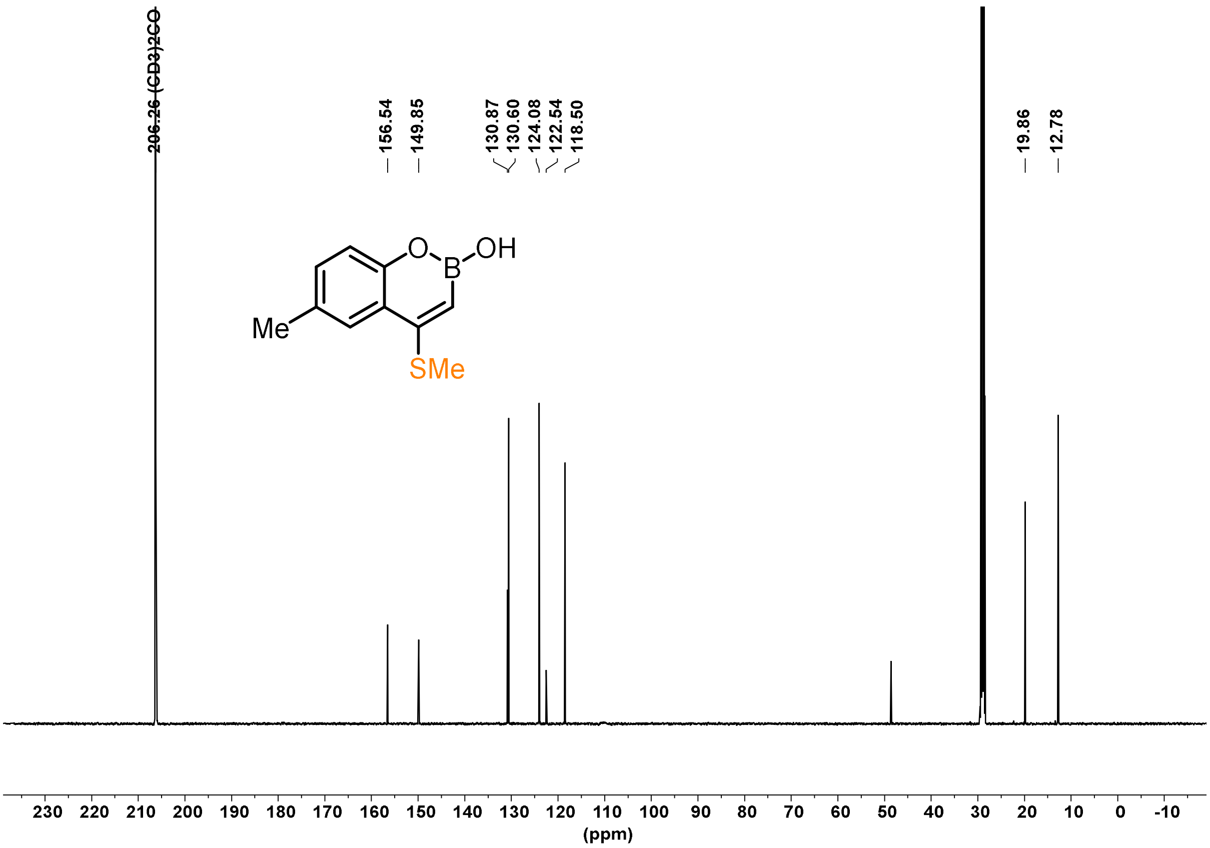

Figure S57. $^{13}C\{^1H\}$ NMR spectrum of compound **2n** in acetone-D_6 + 40 uL D_2O.

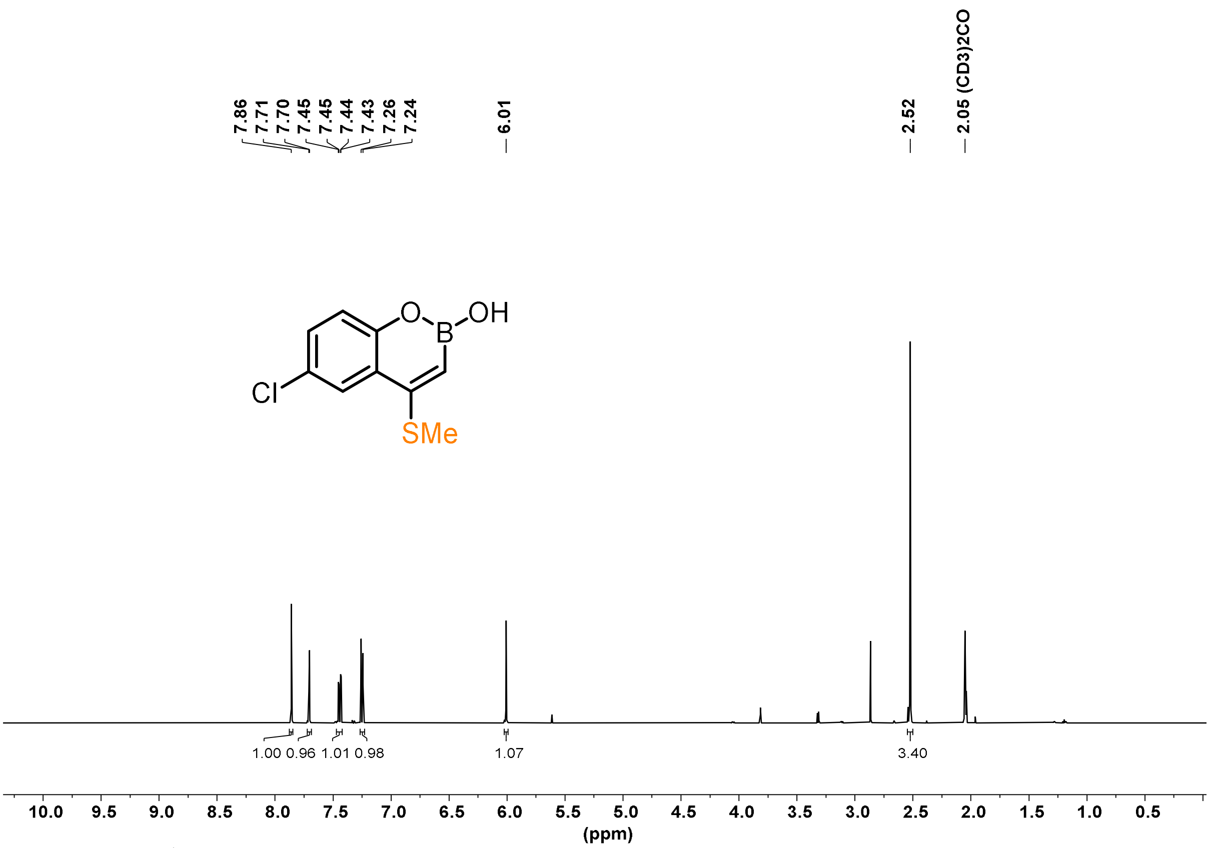

Figure S58. ^1H NMR spectrum of compound **2o** in acetone-D_6.

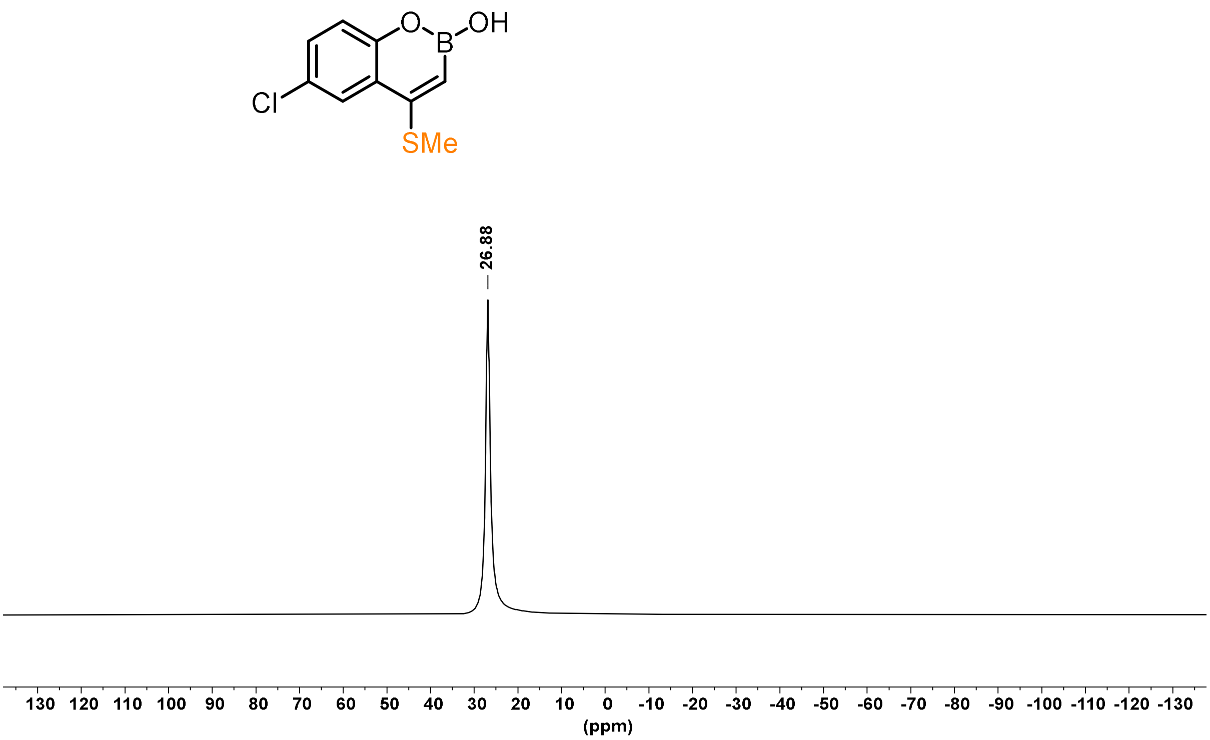

Figure S59. ^{11}B NMR spectrum of compound **2o** in acetone-D_6.

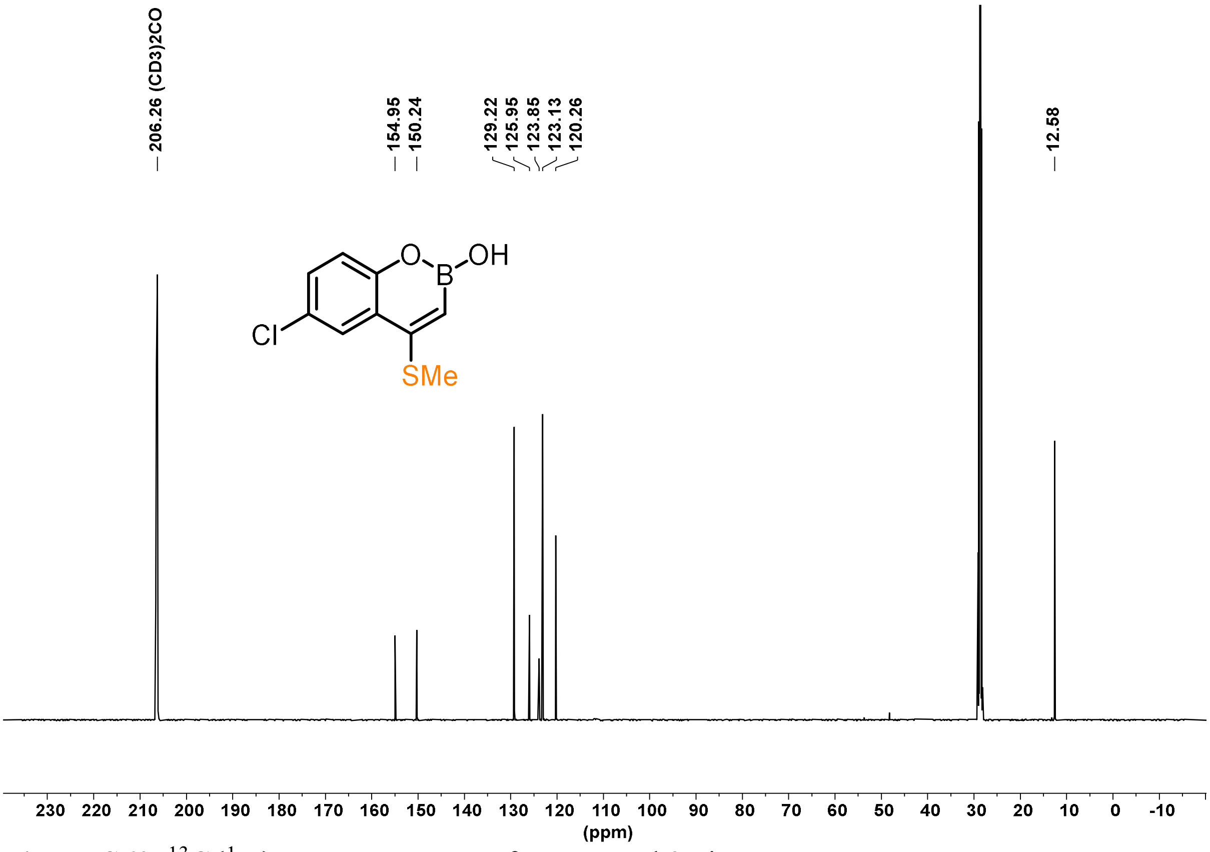

Figure S60. $^{13}C\{^{1}H\}$ NMR spectrum of compound **2o** in acetone-D_6.

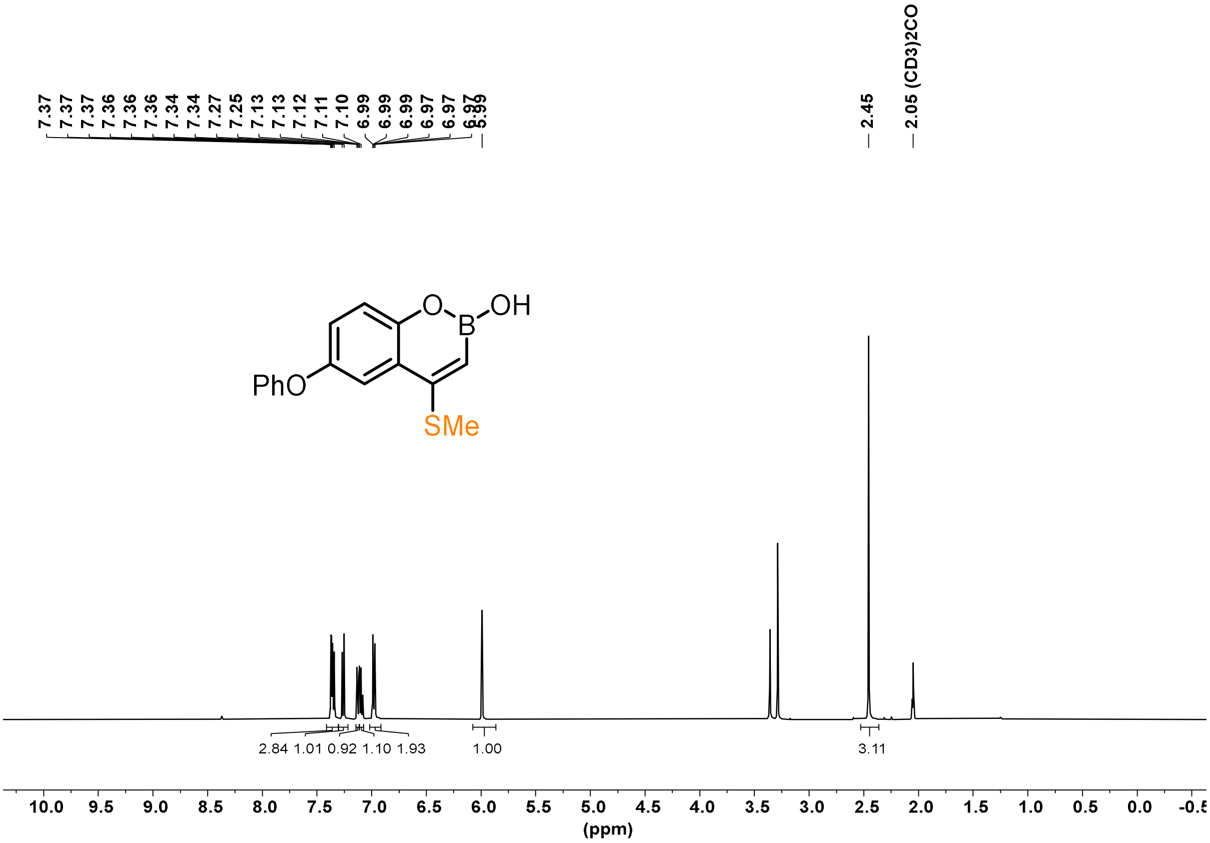

Figure S61. ^{1}H NMR spectrum of compound **2p** in acetone-D_6 + 40 uL D_2O.

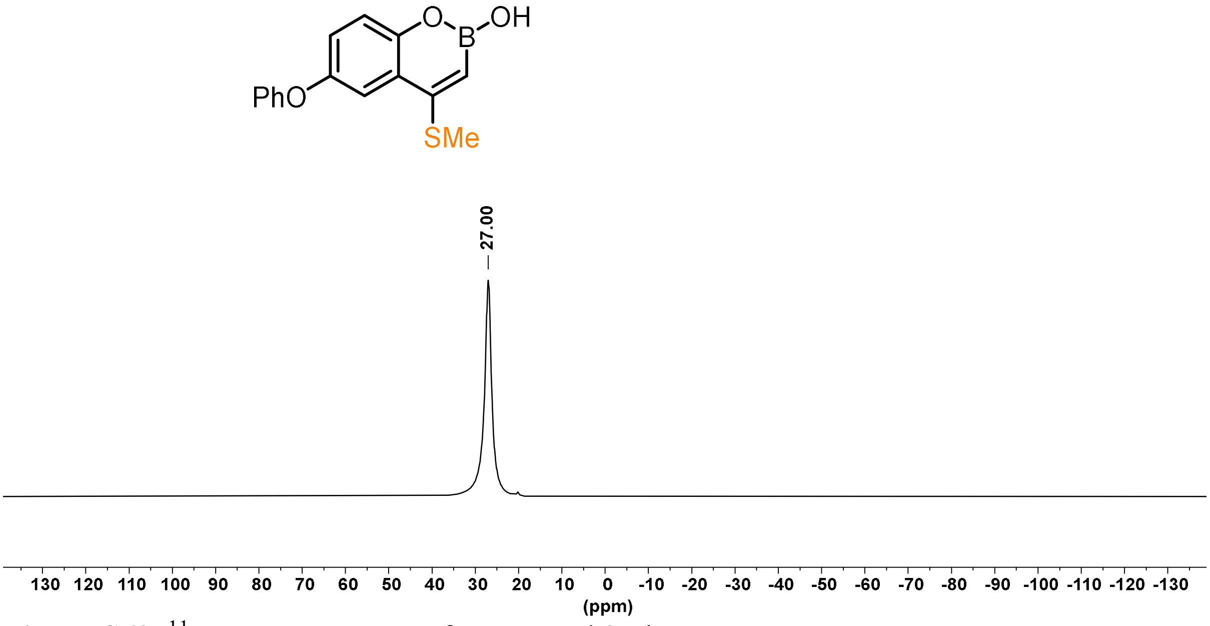

Figure S62. ^{11}B NMR spectrum of compound **2p** in acetone-D_6.

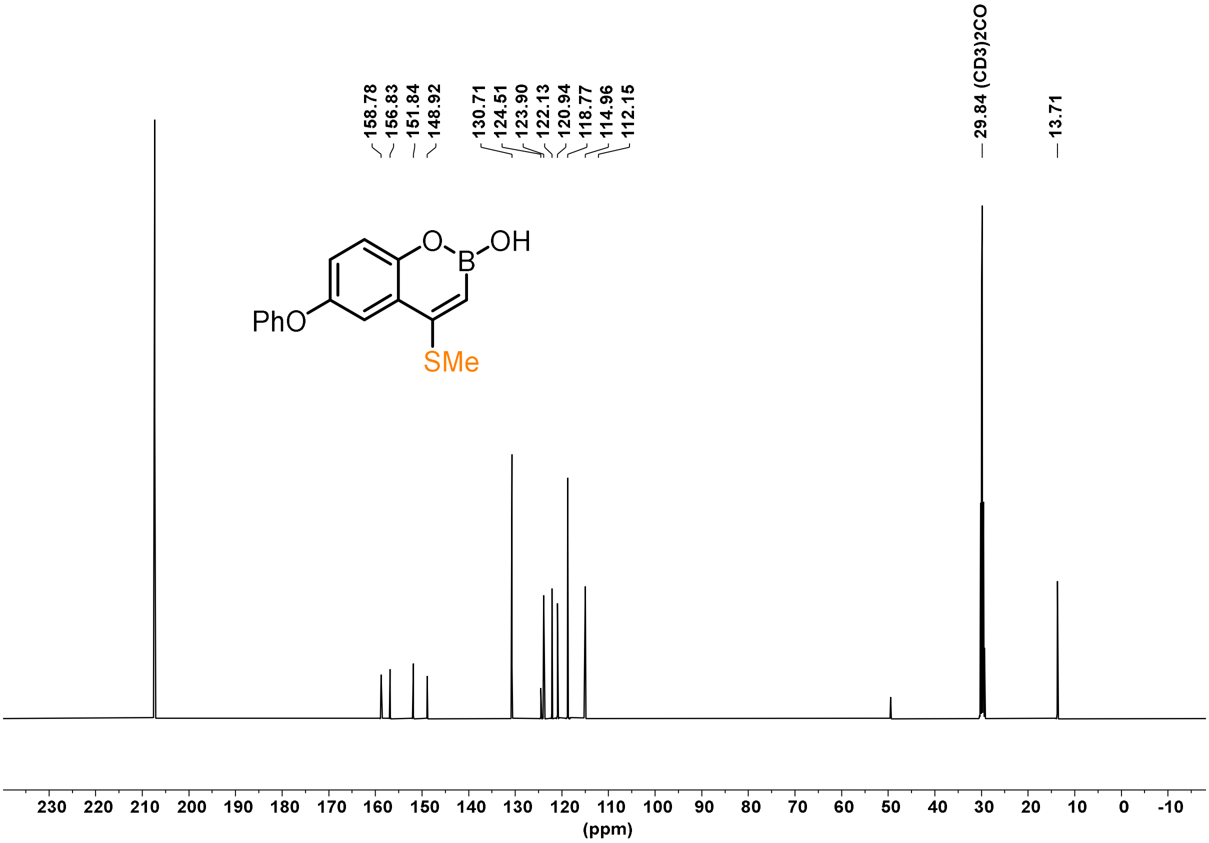

Figure S63. $^{13}C\{^{1}H\}$ NMR spectrum of compound **2p** in acetone-D_6 + 40 uL D_2O.

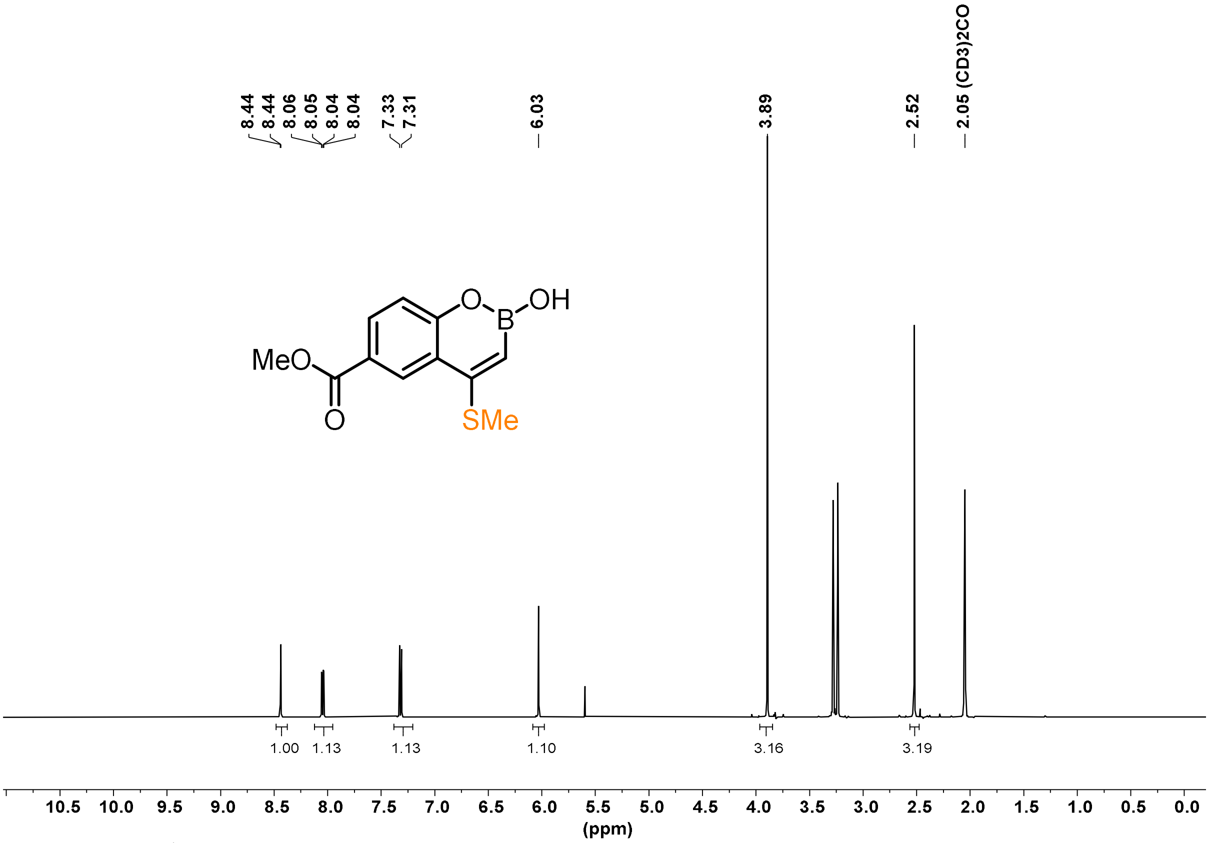

Figure S64. ^{1}H NMR spectrum of compound **2q** in acetone-D_6 + 40 uL D_2O.

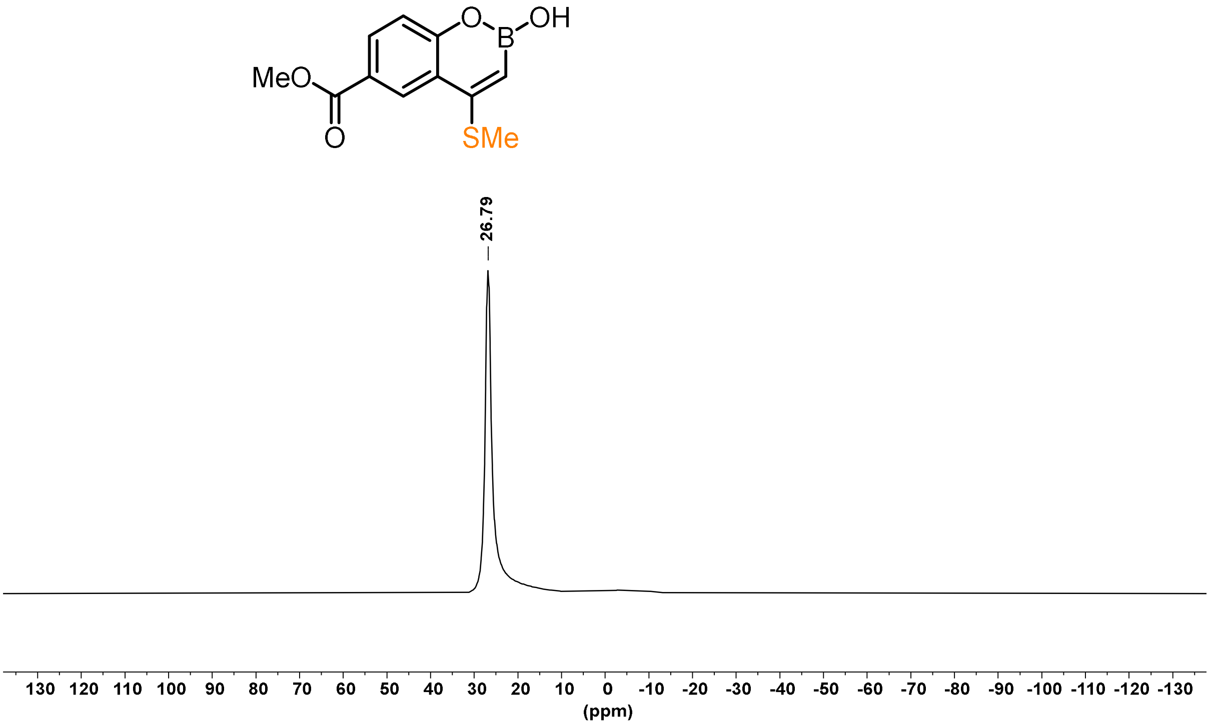

Figure S65. ^{11}B NMR spectrum of compound **2q** in acetone-D_6.

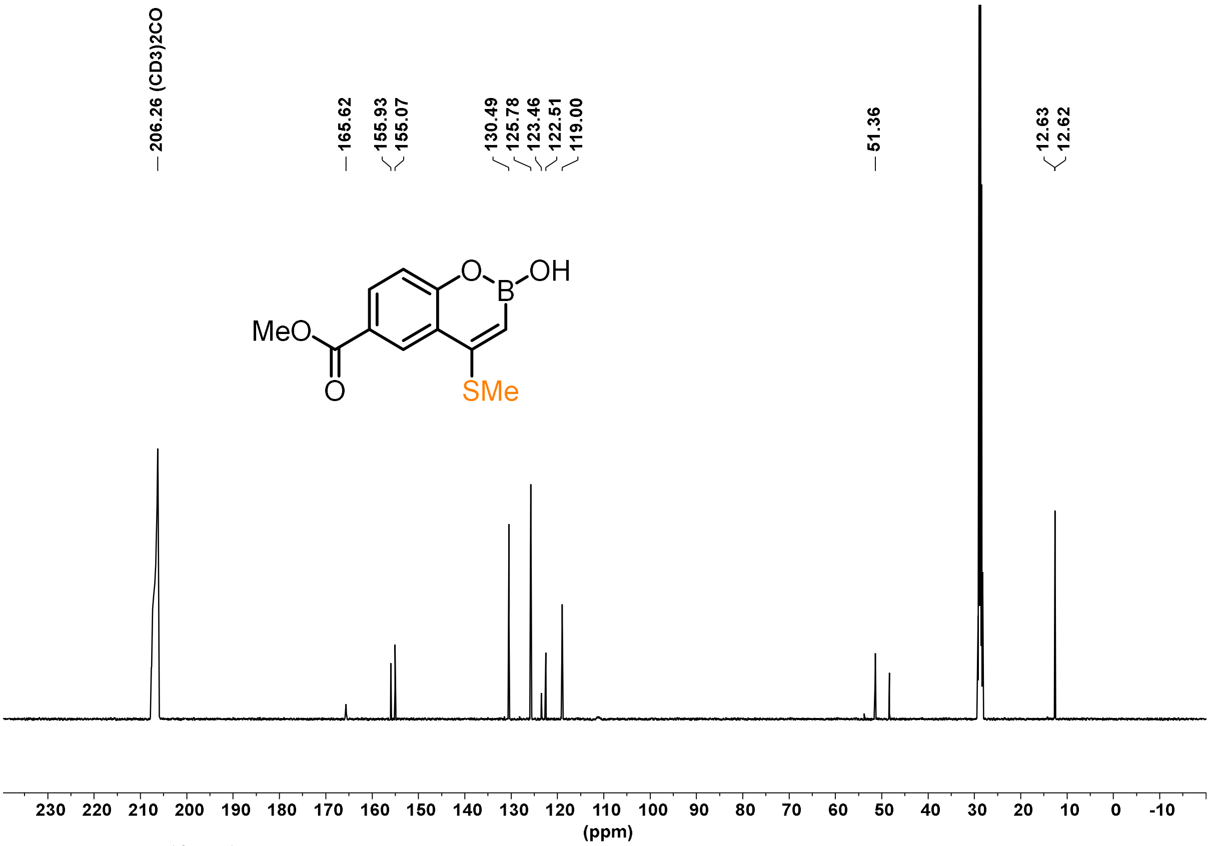

Figure S66. $^{13}C\{^{1}H\}$ NMR spectrum of compound **2q** in acetone-D_6 + 40 uL D_2O.

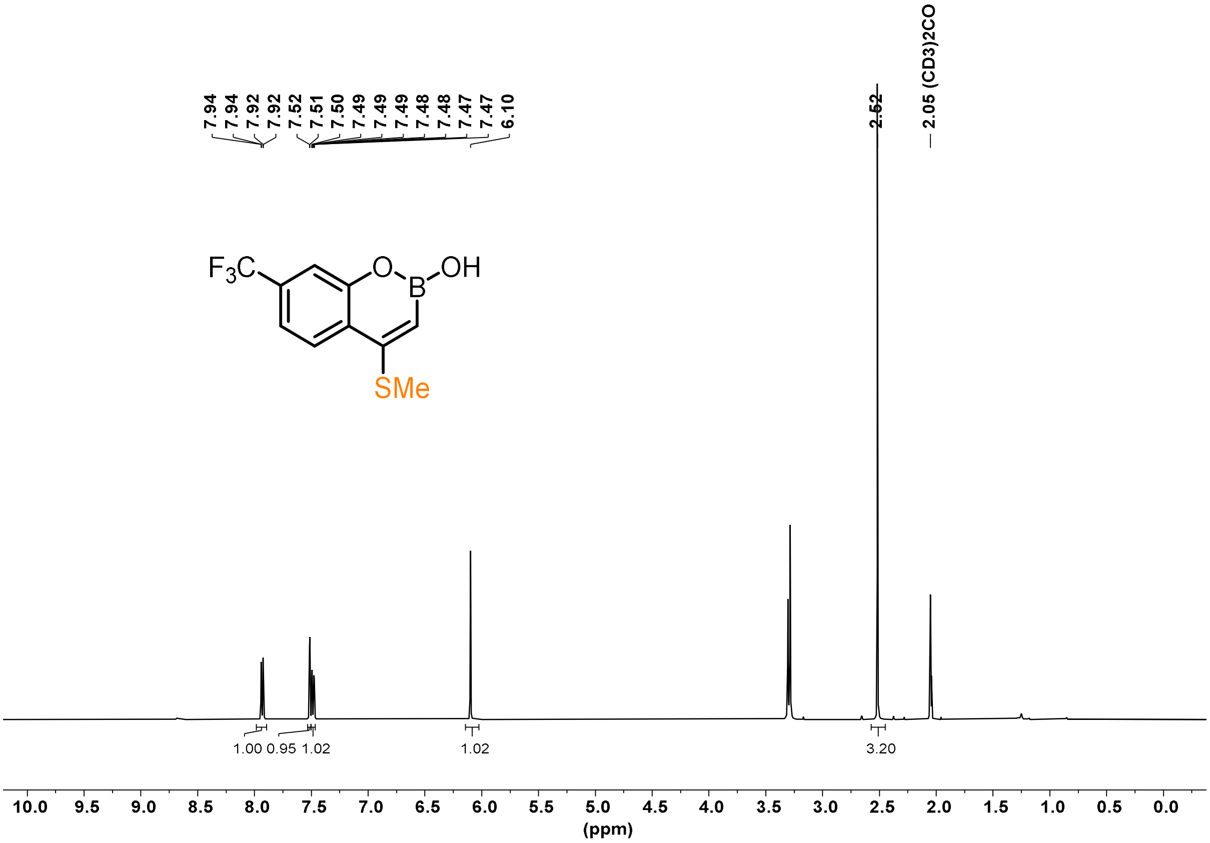

Figure S67. ^{1}H NMR spectrum of compound **2r** in acetone-D$_6$.

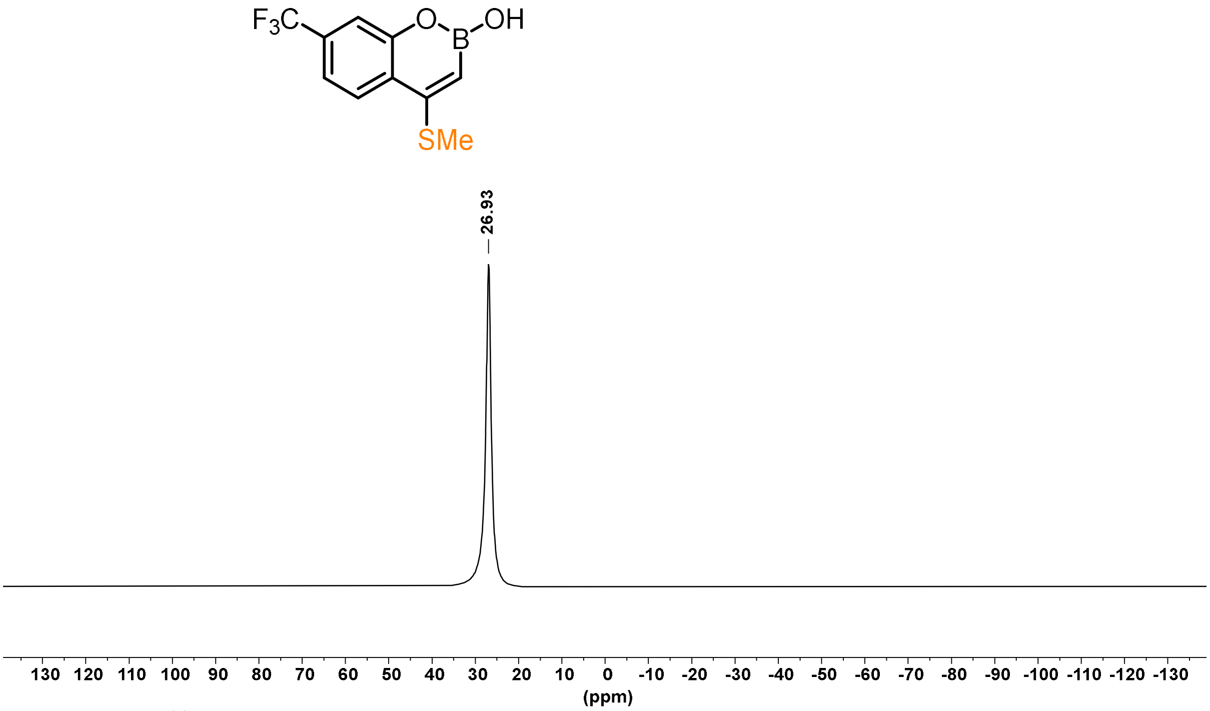

Figure S68. ^{11}B NMR spectrum of compound **2r** in acetone-D$_6$.

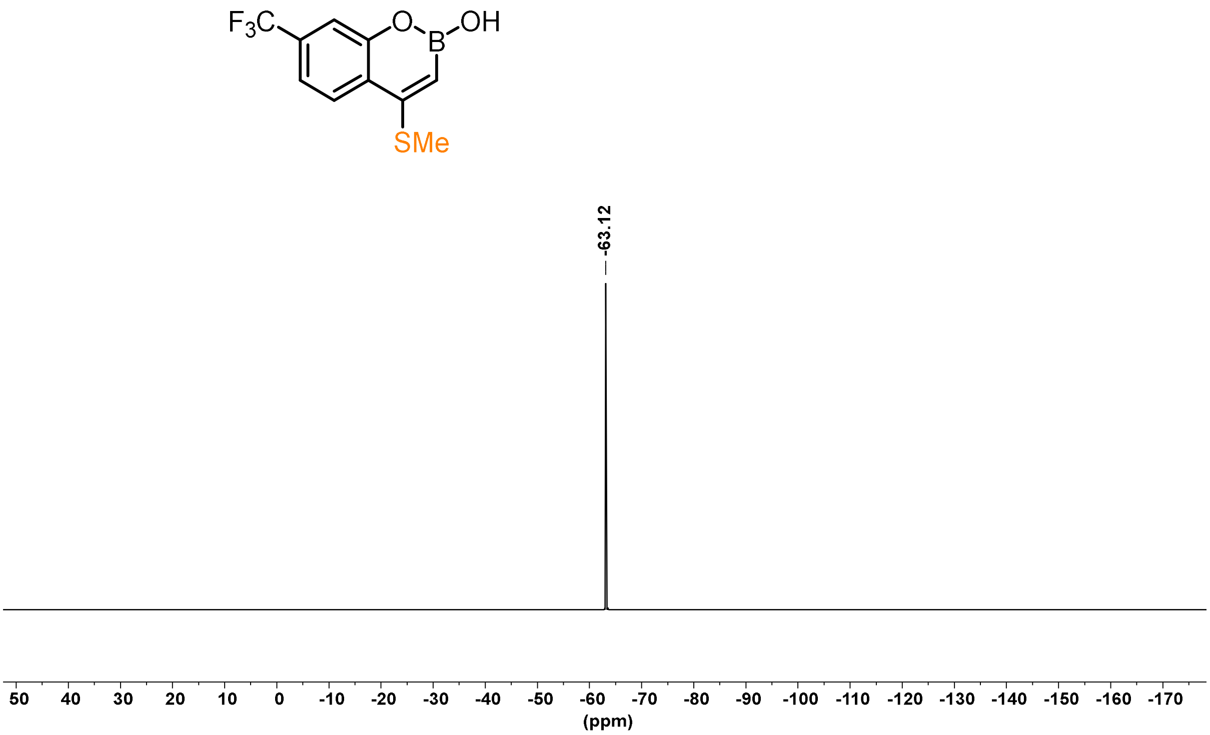

Figure S69. ^{19}F NMR spectrum of compound **2r** in acetone-D$_6$.

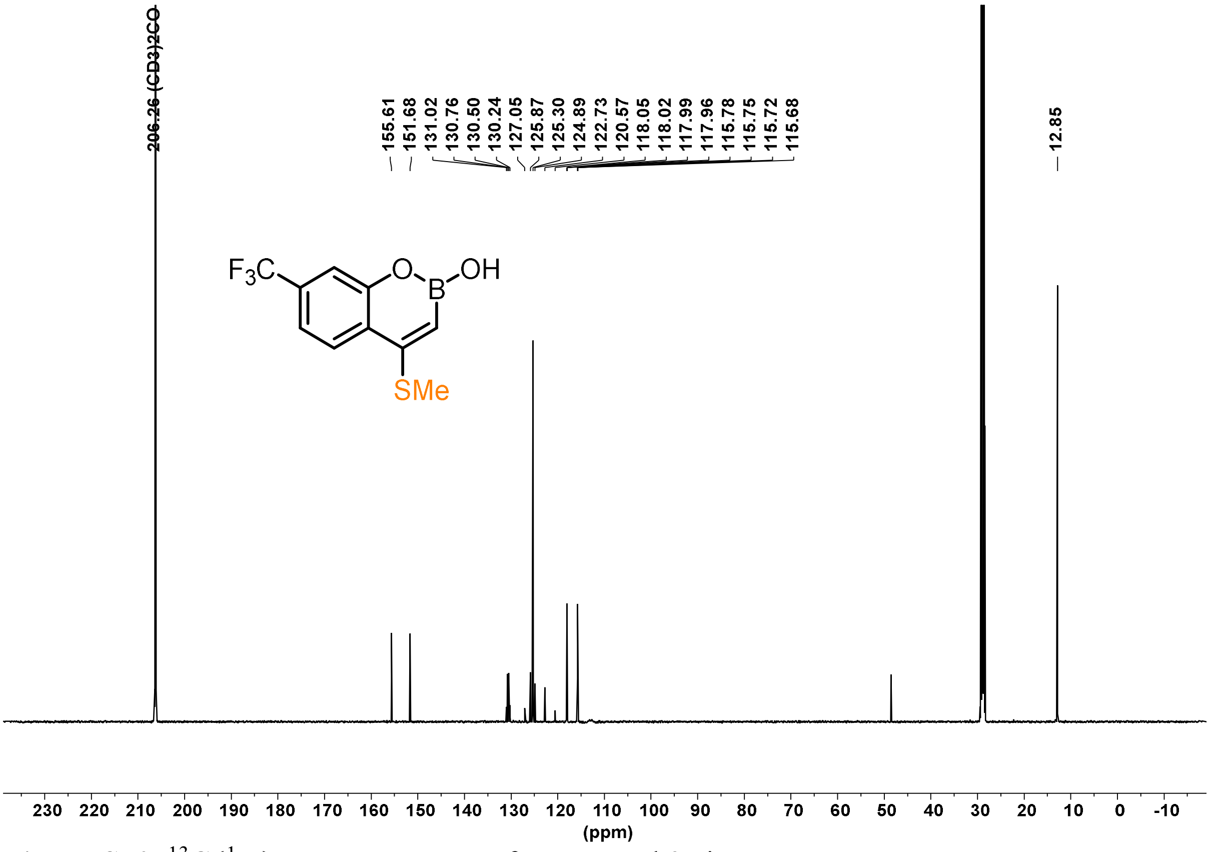

Figure S70. ^{13}C{^{1}H} NMR spectrum of compound **2r** in acetone-D$_6$.

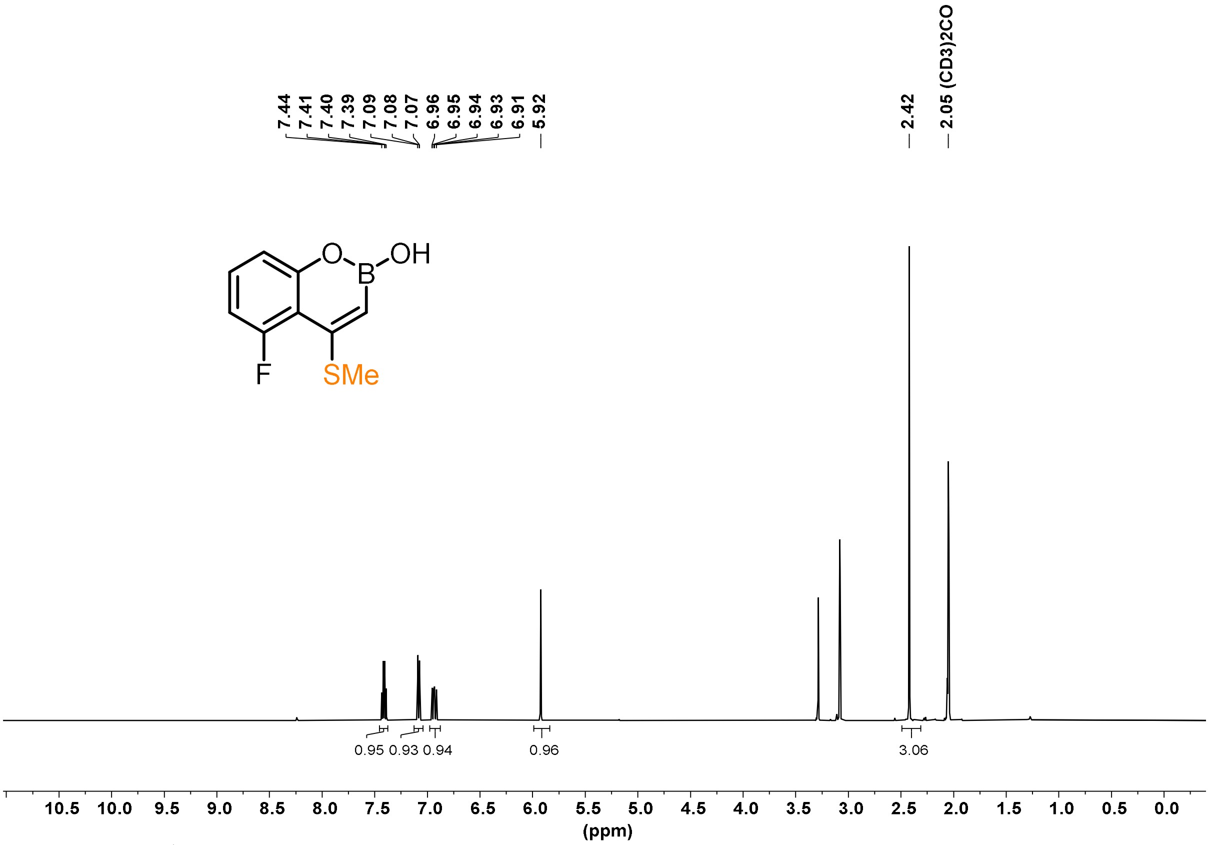

Figure S71. ^{1}H NMR spectrum of compound **2s** in acetone-D_6 + 40 uL D_2O.

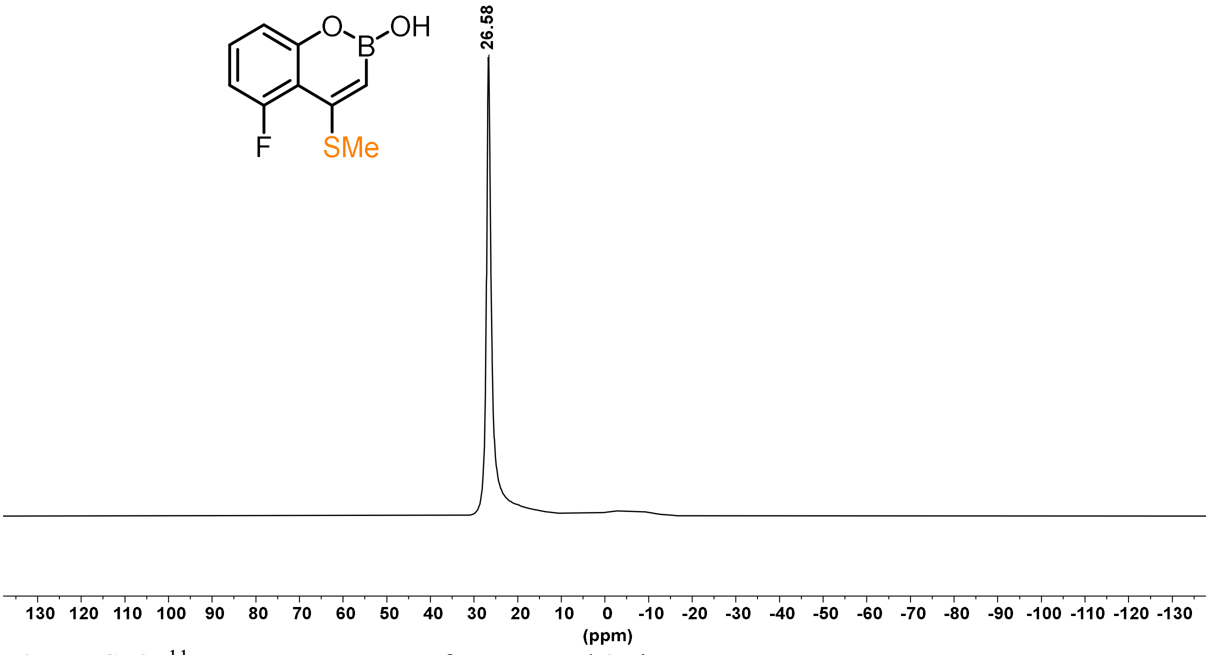

Figure S72. ^{11}B NMR spectrum of compound **2s** in acetone-D_6.

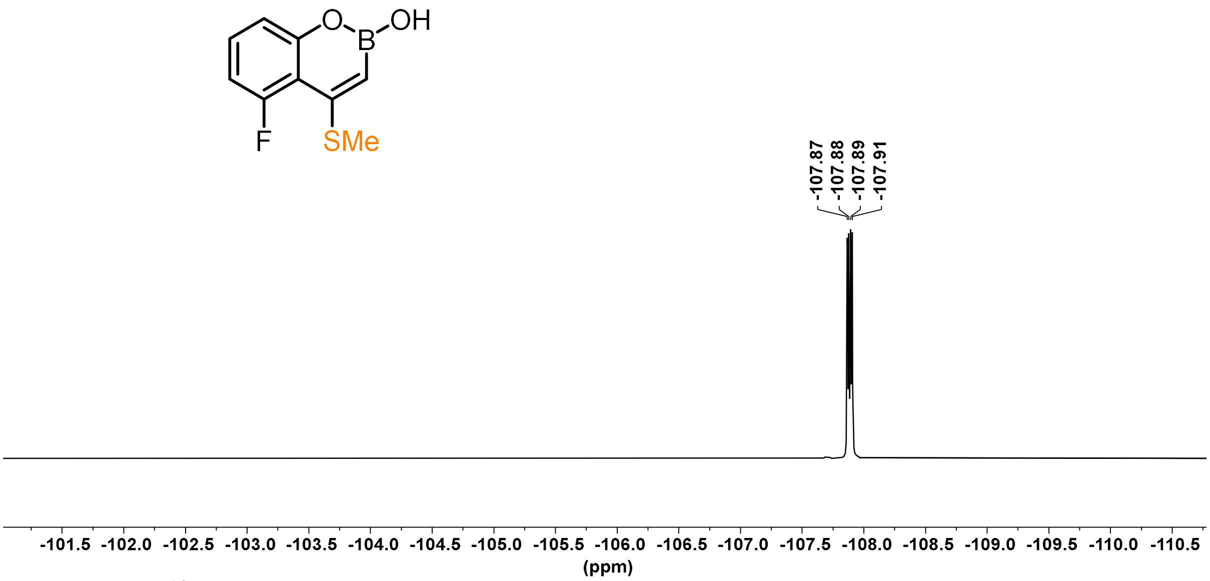

Figure S73. ^{19}F NMR spectrum of compound **2s** in acetone-D6.

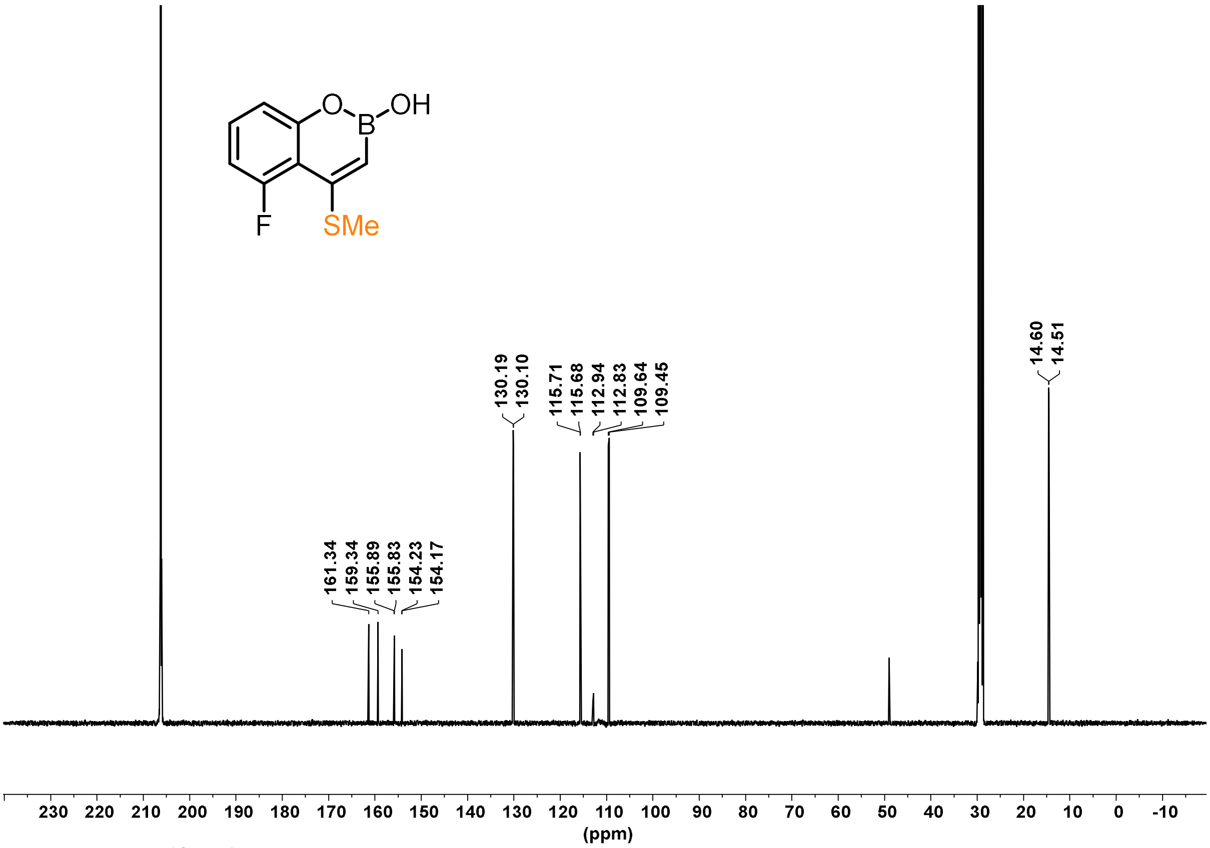

Figure S74. $^{13}C\{^1H\}$ NMR spectrum of compound **2s** in acetone-D6 + 40 uL D_2O.

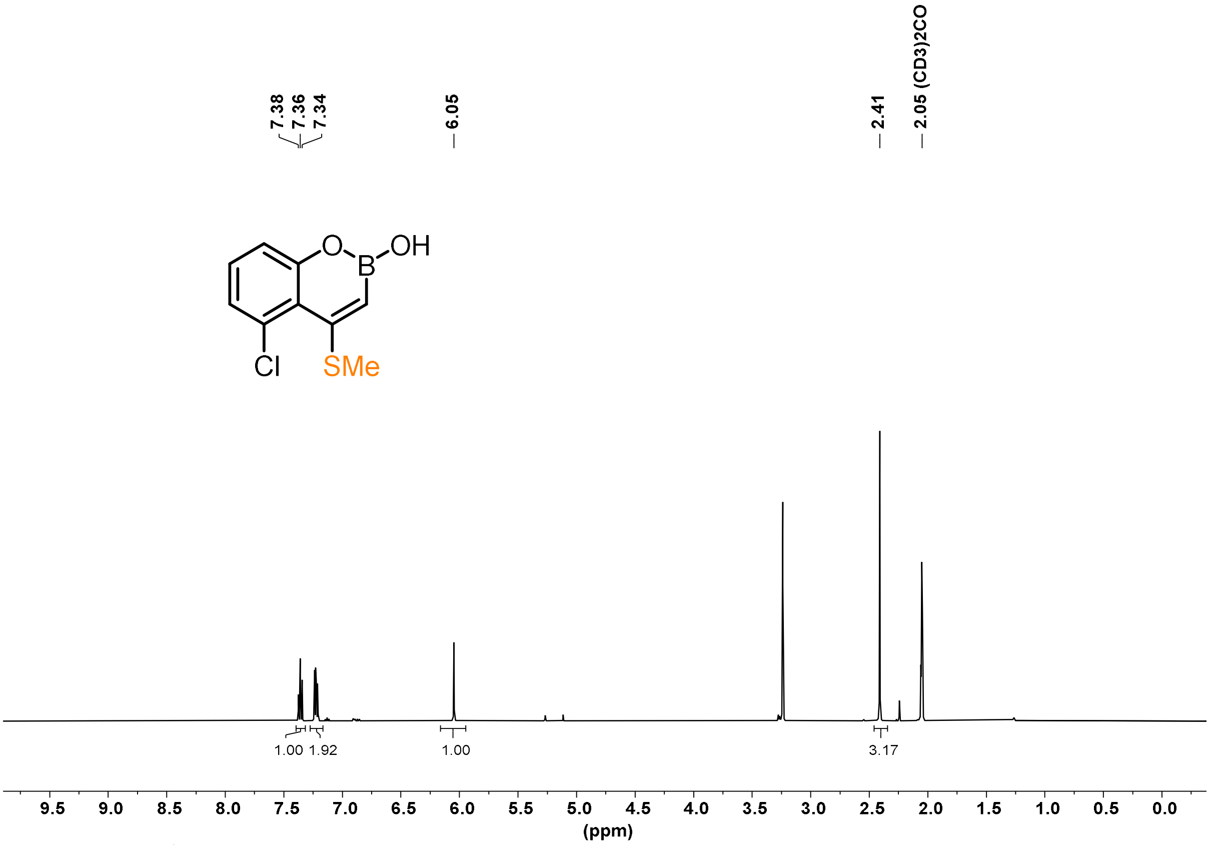

Figure S75. ^{1}H NMR spectrum of compound **2t** in acetone-D_6 + 40 uL D_2O.

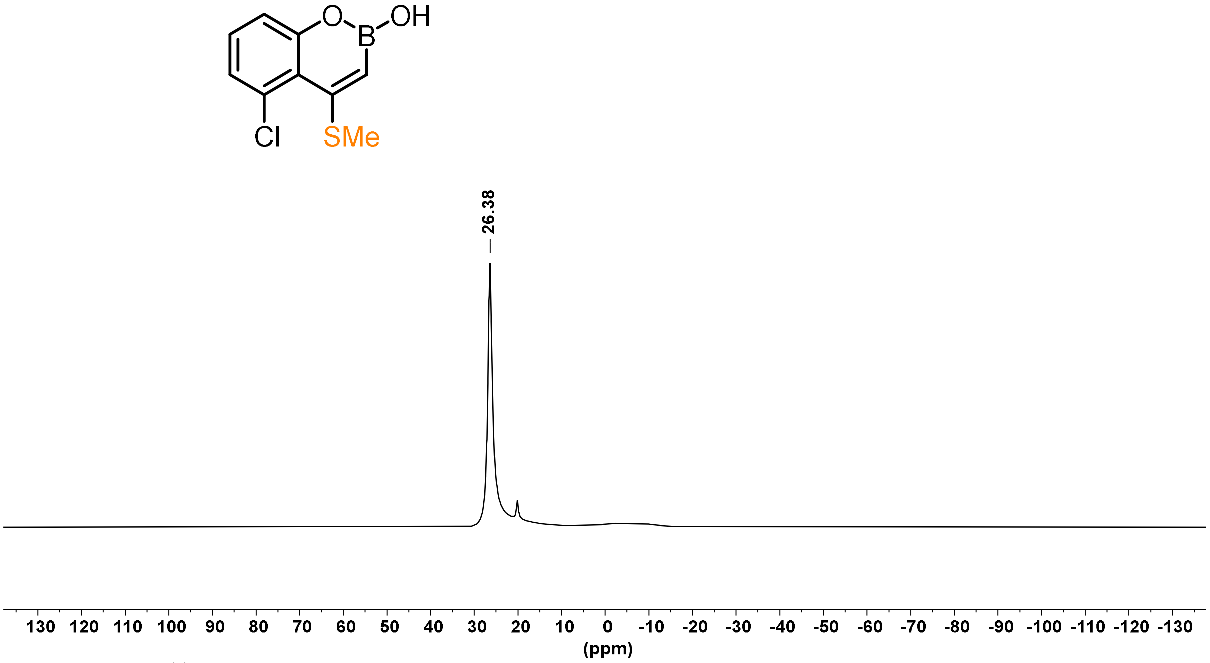

Figure S76. ^{11}B NMR spectrum of compound **2t** in acetone-D_6.

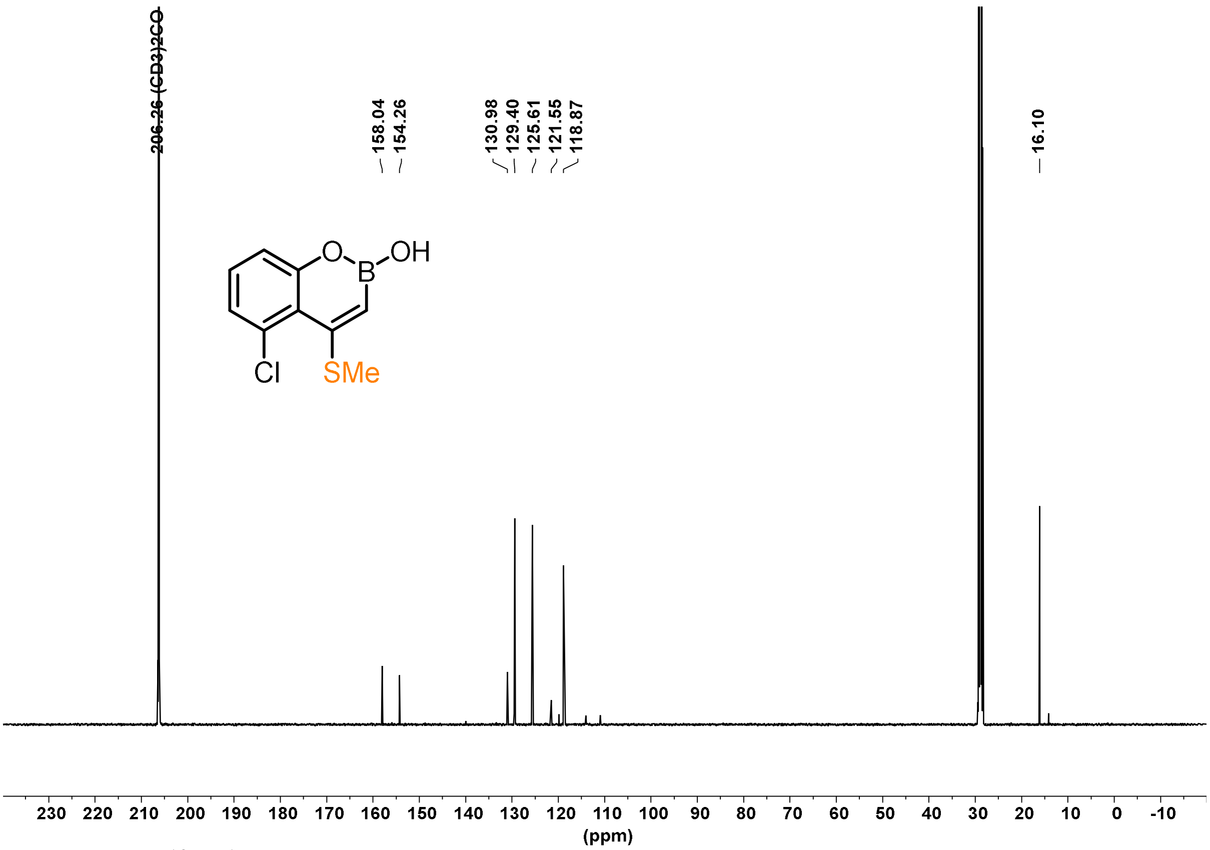

Figure S77. $^{13}C\{^{1}H\}$ NMR spectrum of compound **2t** in acetone-D_6 + 40 uL D_2O.

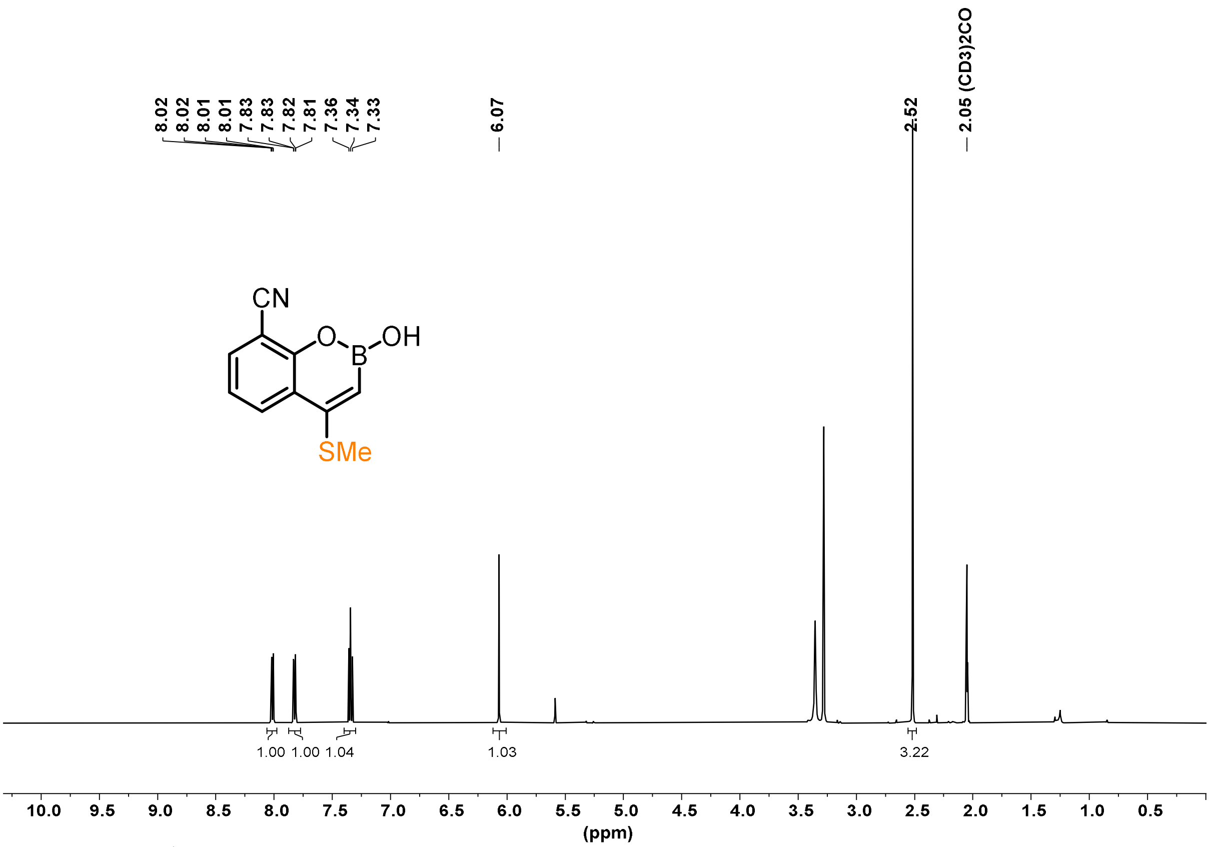

Figure S78. ^{1}H NMR spectrum of compound **2u** in acetone-D_6 + 40 uL D_2O.

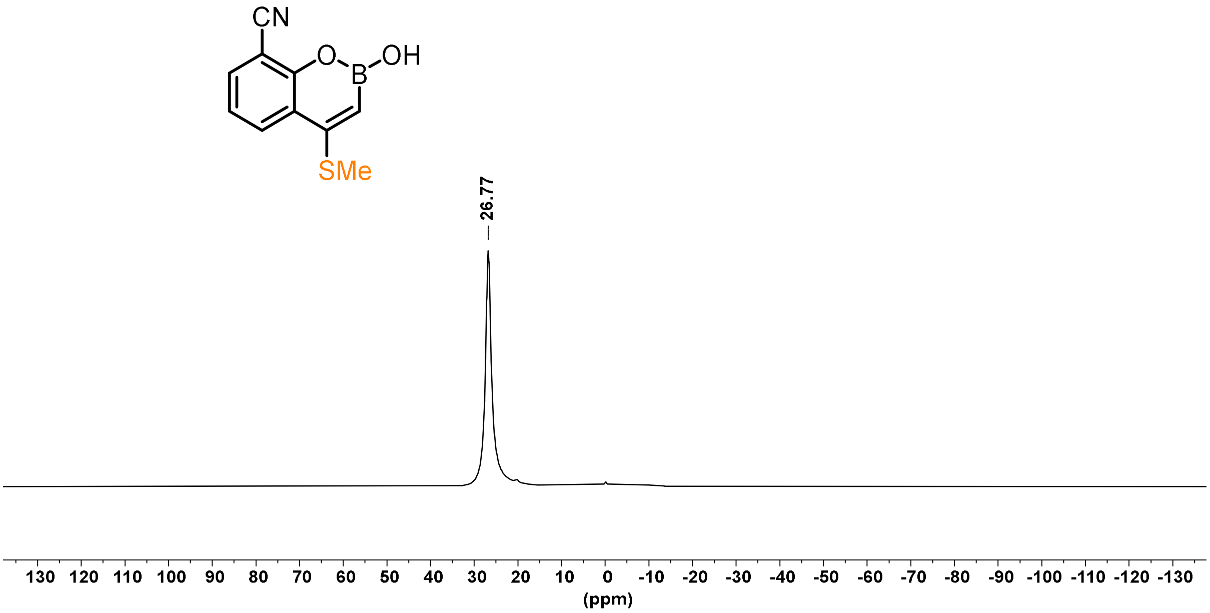

Figure S79. ^{11}B NMR spectrum of compound **2u** in acetone-D$_6$.

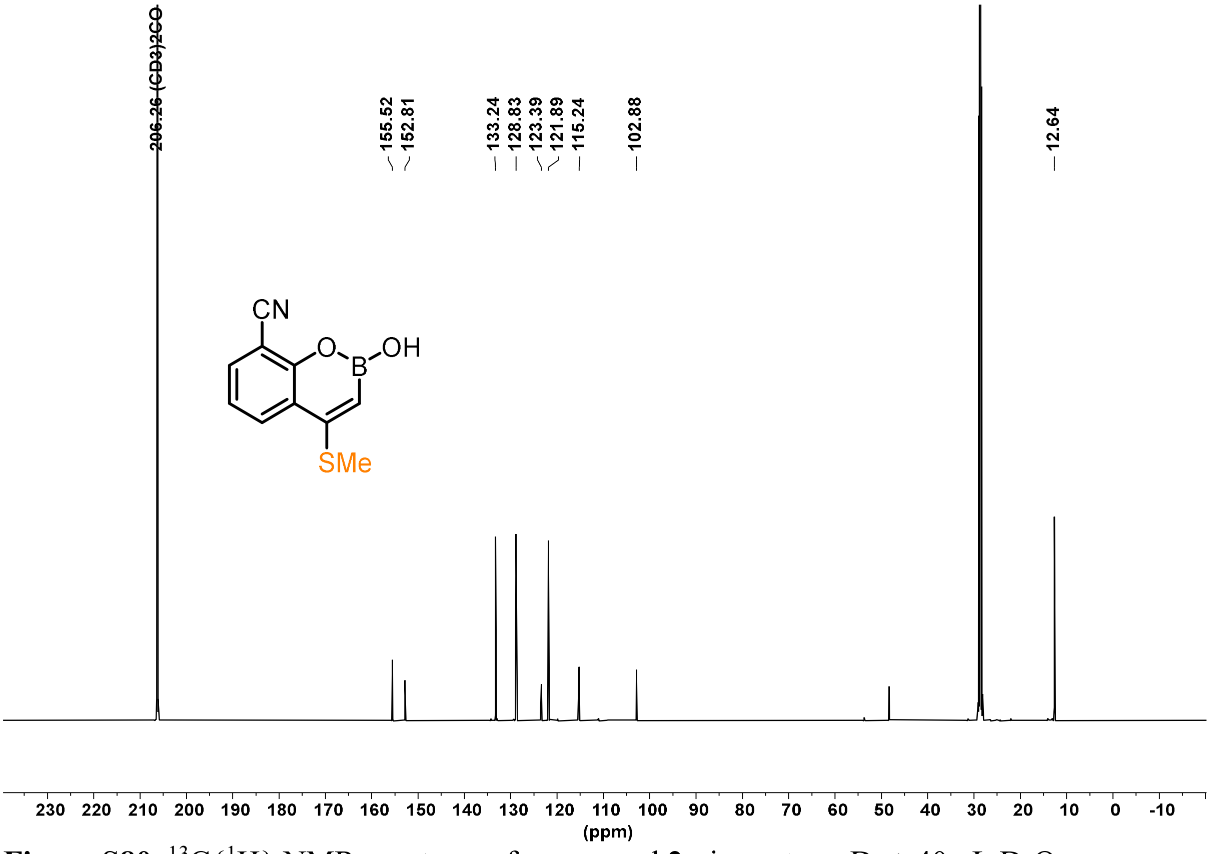

Figure S80. ^{13}C{^{1}H} NMR spectrum of compound **2u** in acetone-D$_6$ + 40 uL D$_2$O.

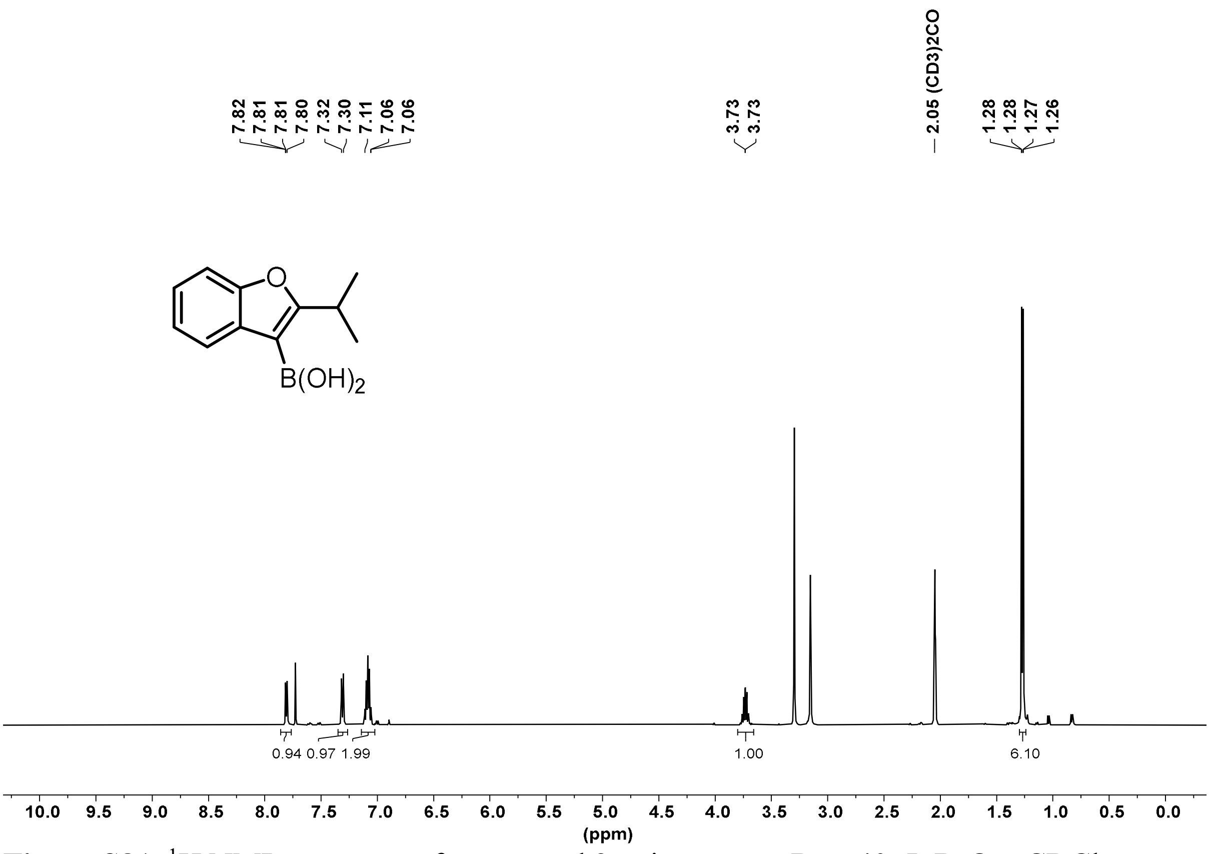

Figure S81. ^{1}H NMR spectrum of compound **2aa** in acetone-D_6 + 40μL D_2O + $CDCl_3$.

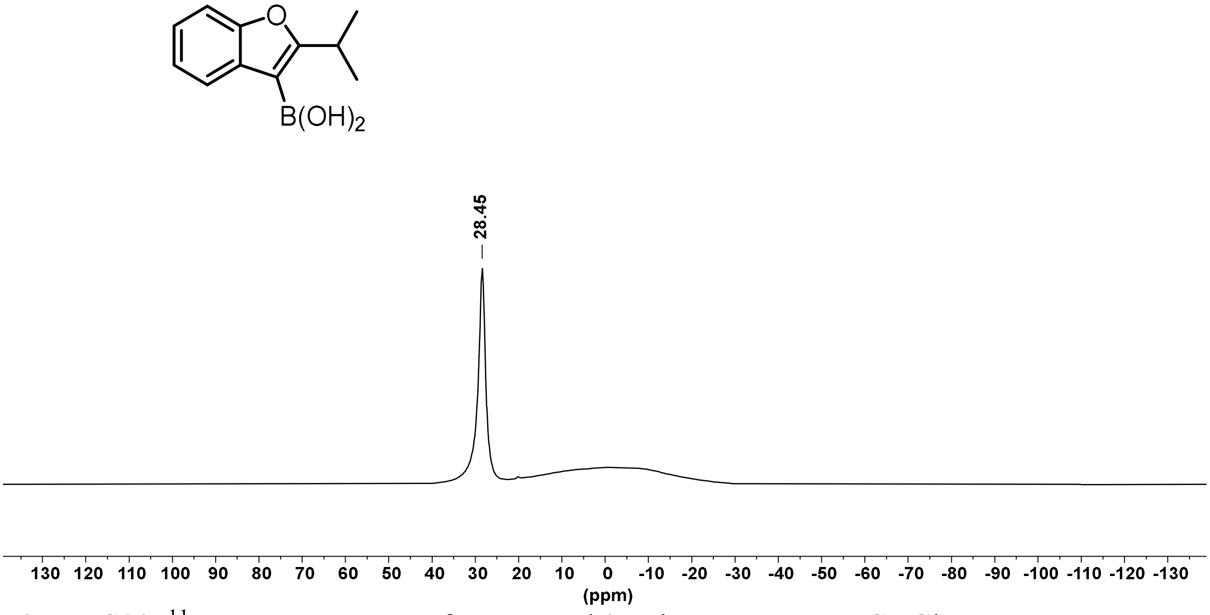

Figure S82. ^{11}B NMR spectrum of compound **2aa** in acetone-D_6 + $CDCl_3$.

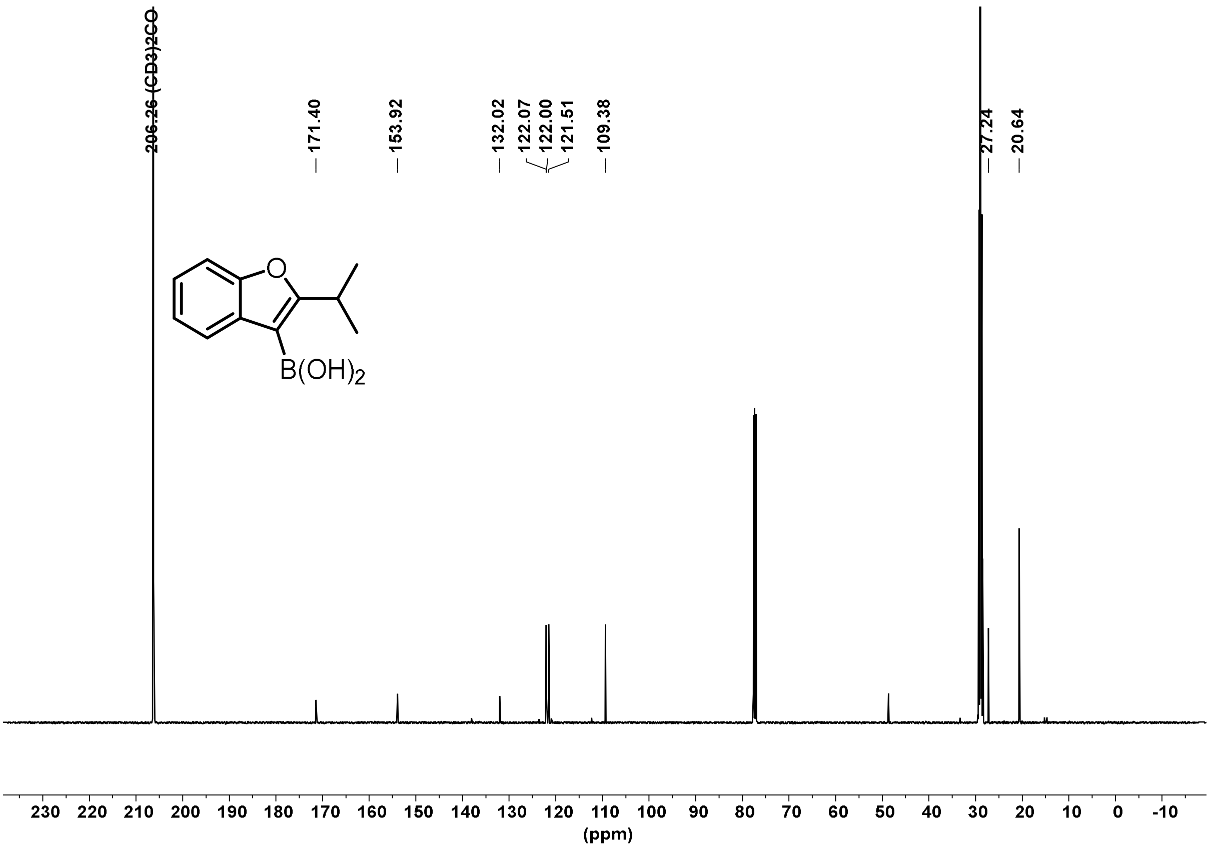

Figure S83. $^{13}C\{^1H\}$ NMR spectrum of compound **2aa** in acetone-D_6 + 40 uL D_2O + $CDCl_3$.

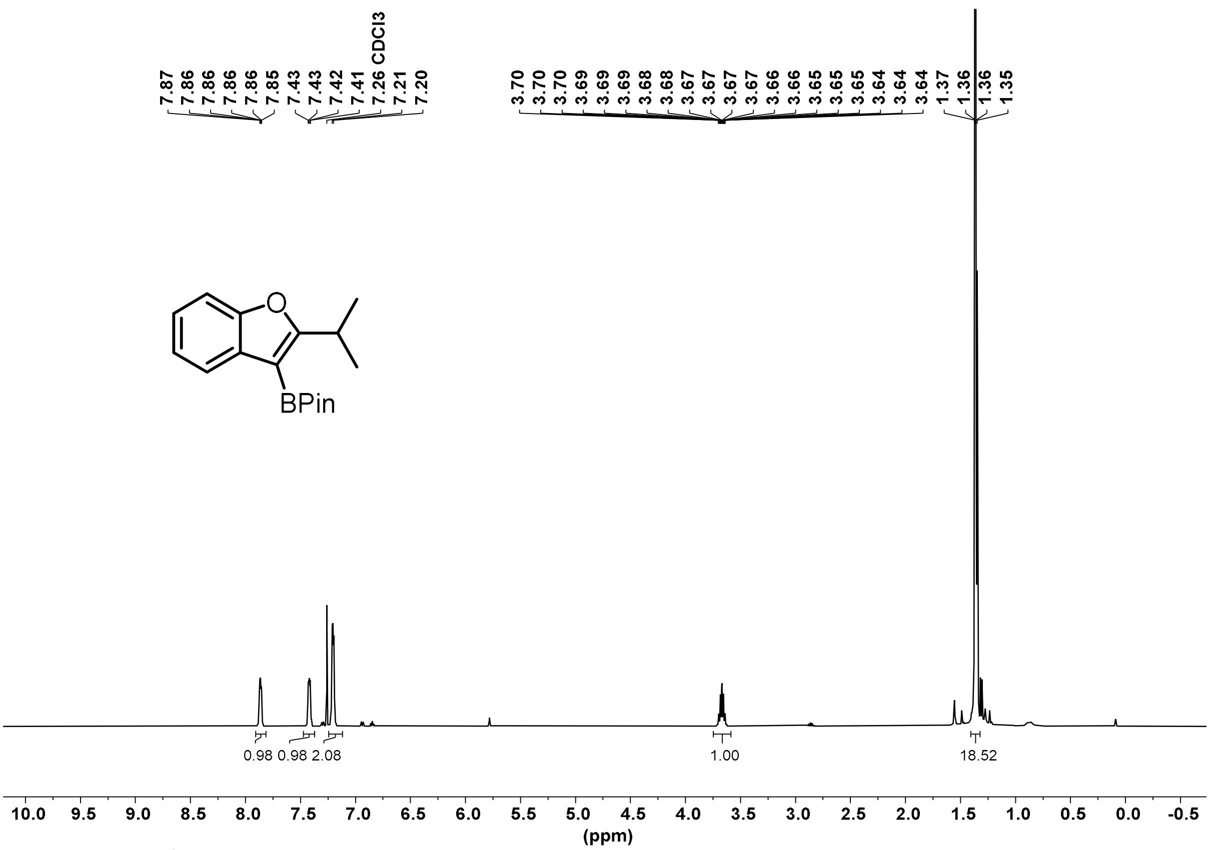

Figure S84. ^1H NMR spectrum of compound **2ab** in $CDCl_3$.

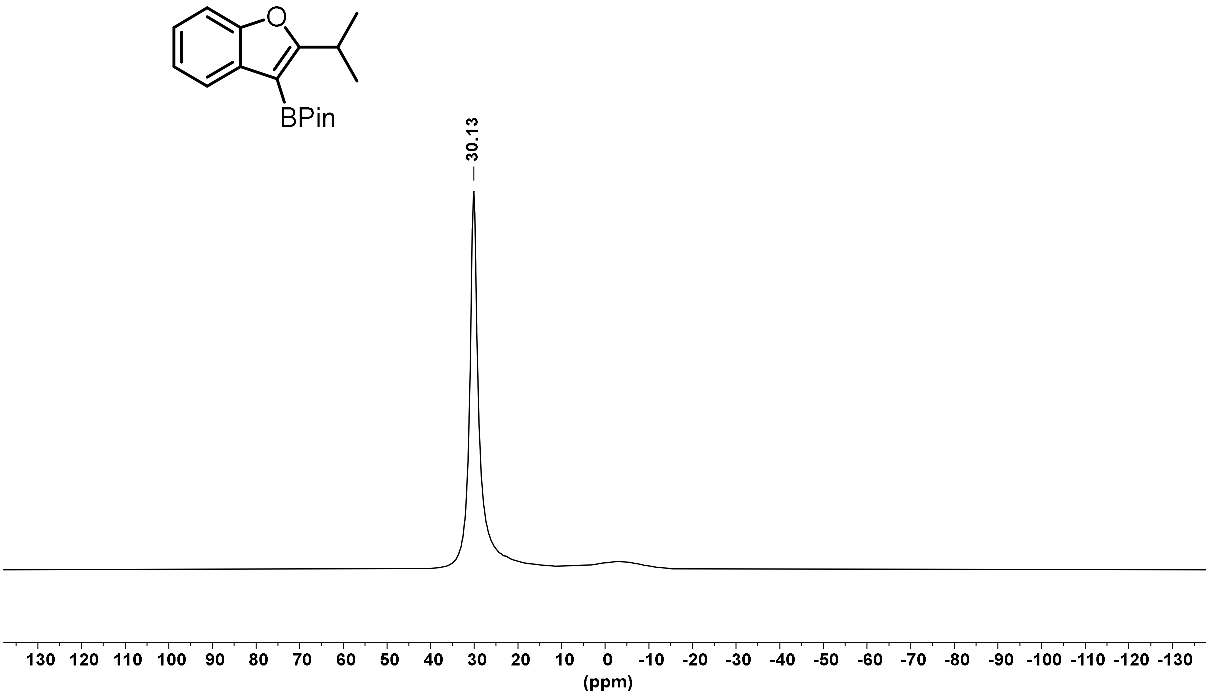

Figure S85. ^{11}B NMR spectrum of compound **2ab** in $CDCl_3$.

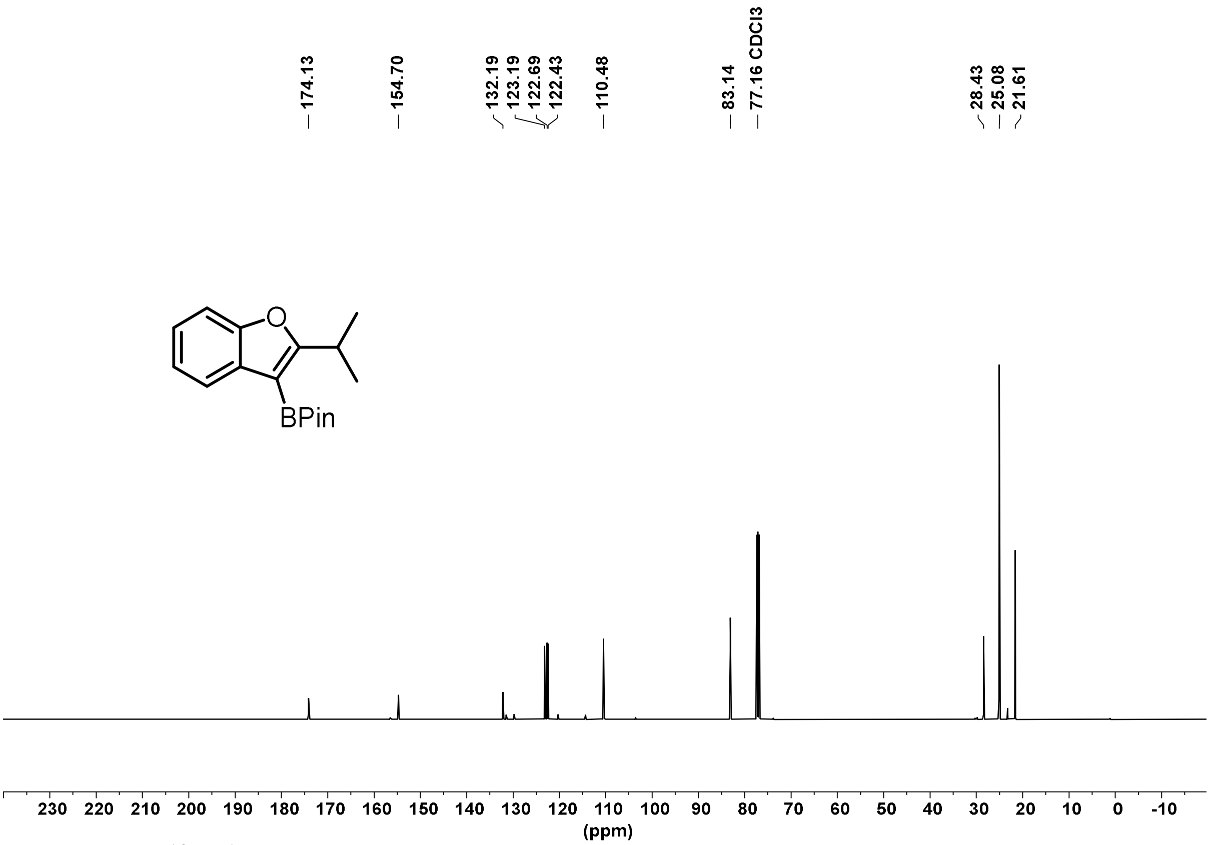

Figure S86. $^{13}C\{^1H\}$ NMR spectrum of compound **2ab** in $CDCl_3$.

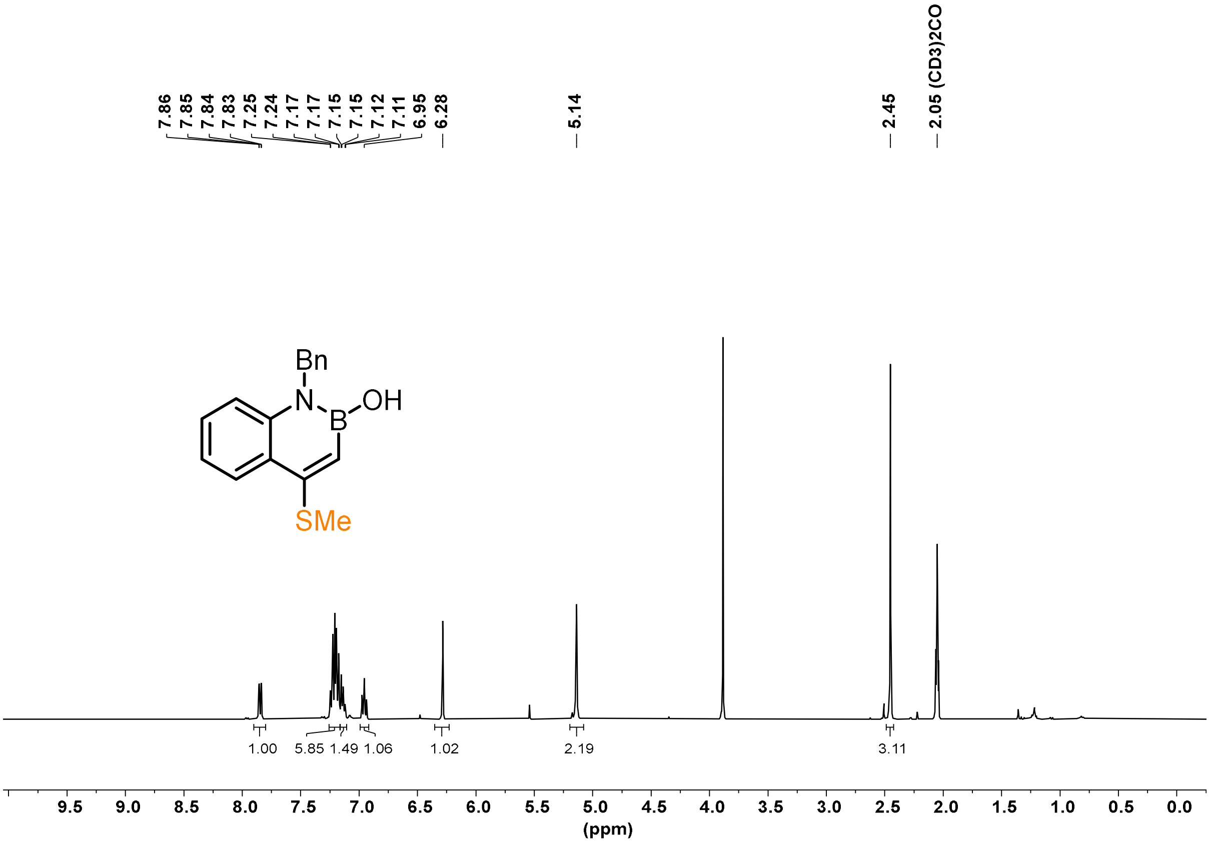

Figure S87. ^{1}H NMR spectrum of compound **4a** in acetone-D_6 + D_2O.

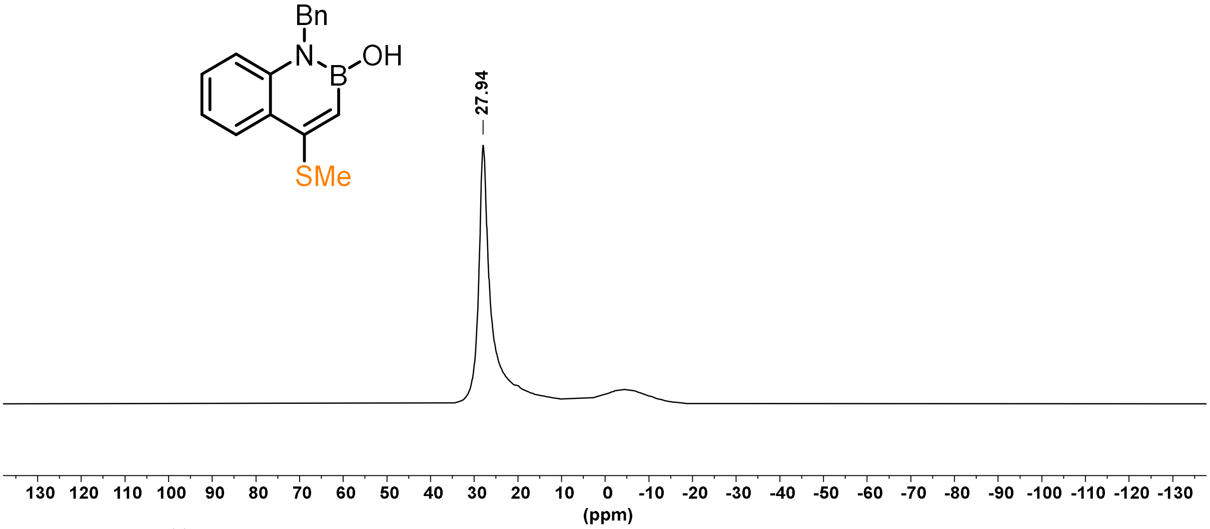

Figure S88. ^{11}B NMR spectrum of compound **4a** in acetone-D_6 + D_2O.

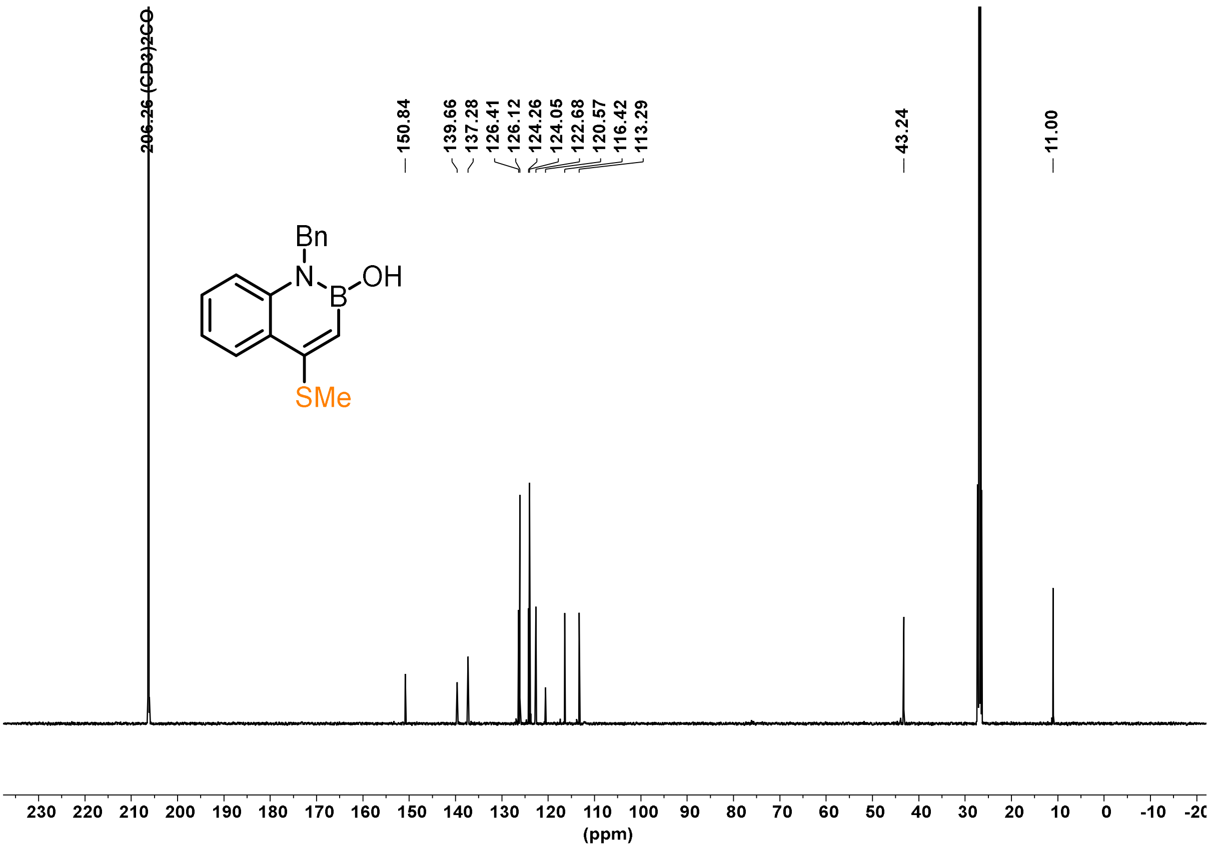

Figure S89. $^{13}C\{^1H\}$ NMR spectrum of compound **4a** in acetone-D$_6$ + D$_2$O.

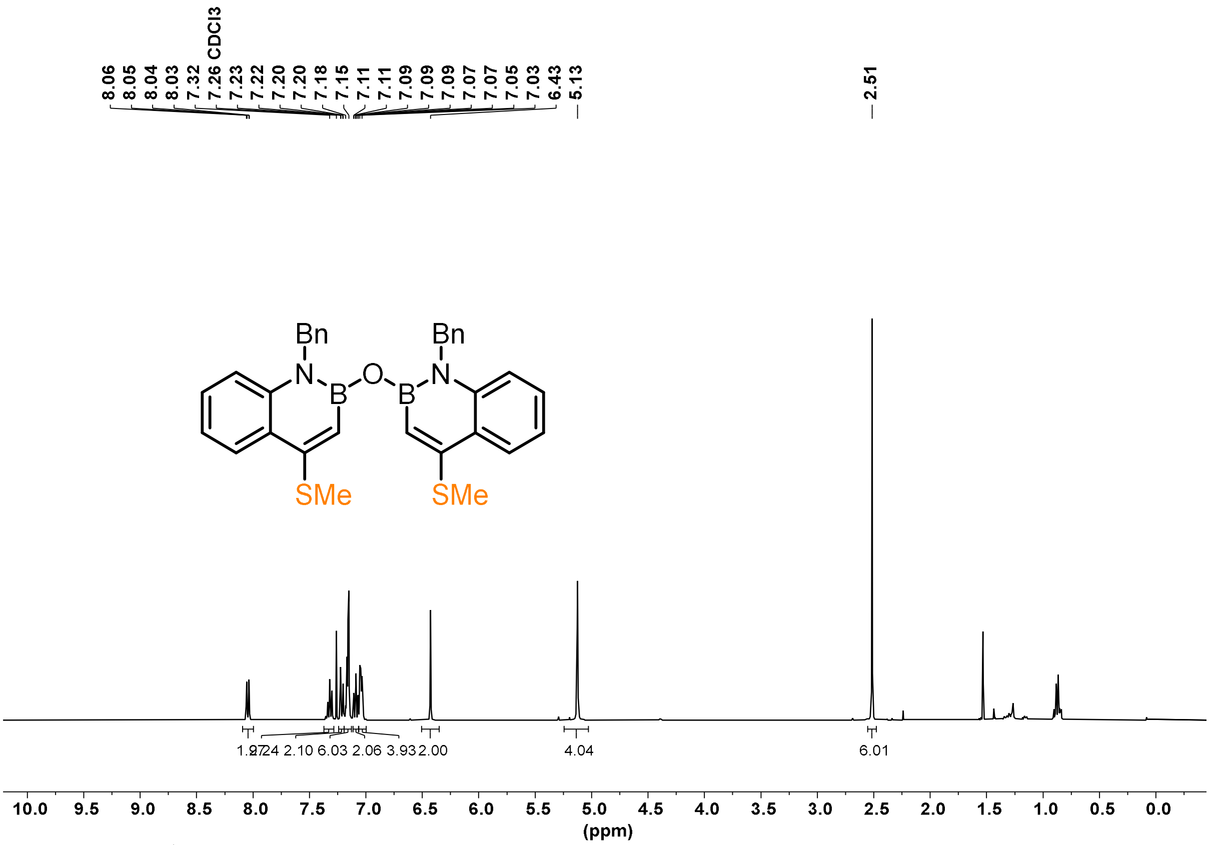

Figure S90. ^1H NMR spectrum of compound **4a-anhydride** in $CDCl_3$.

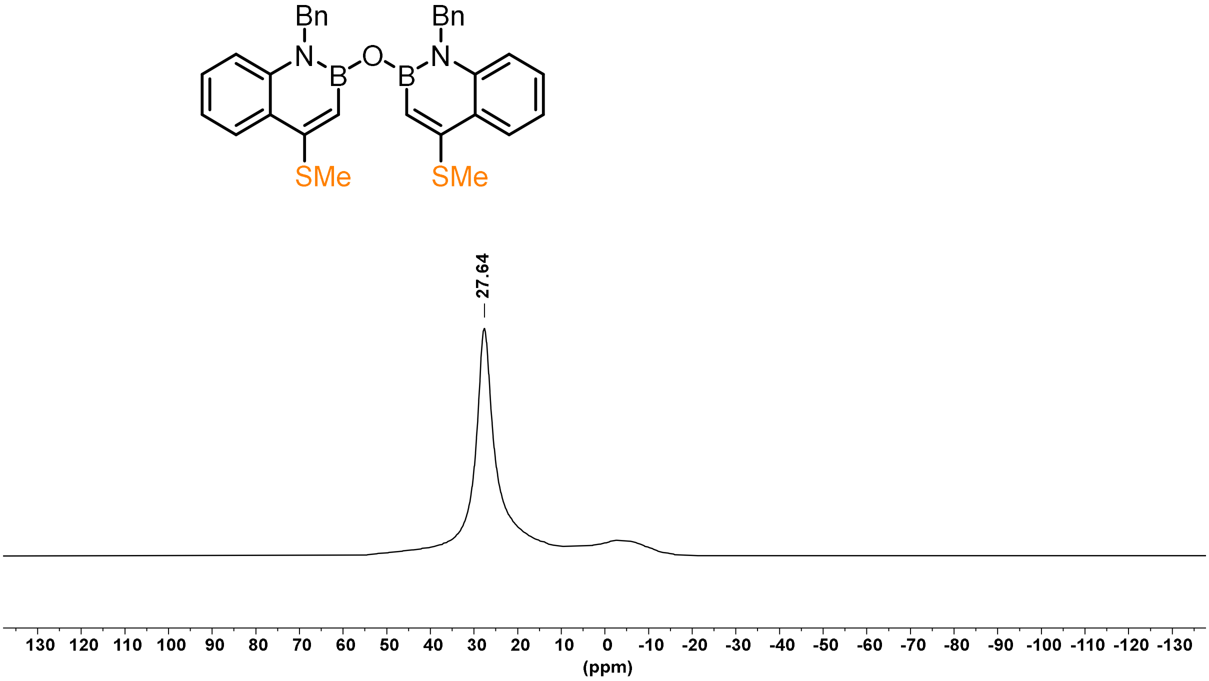

Figure S91. ^{11}B NMR spectrum of compound **4a-anhydride** in $CDCl_3$.

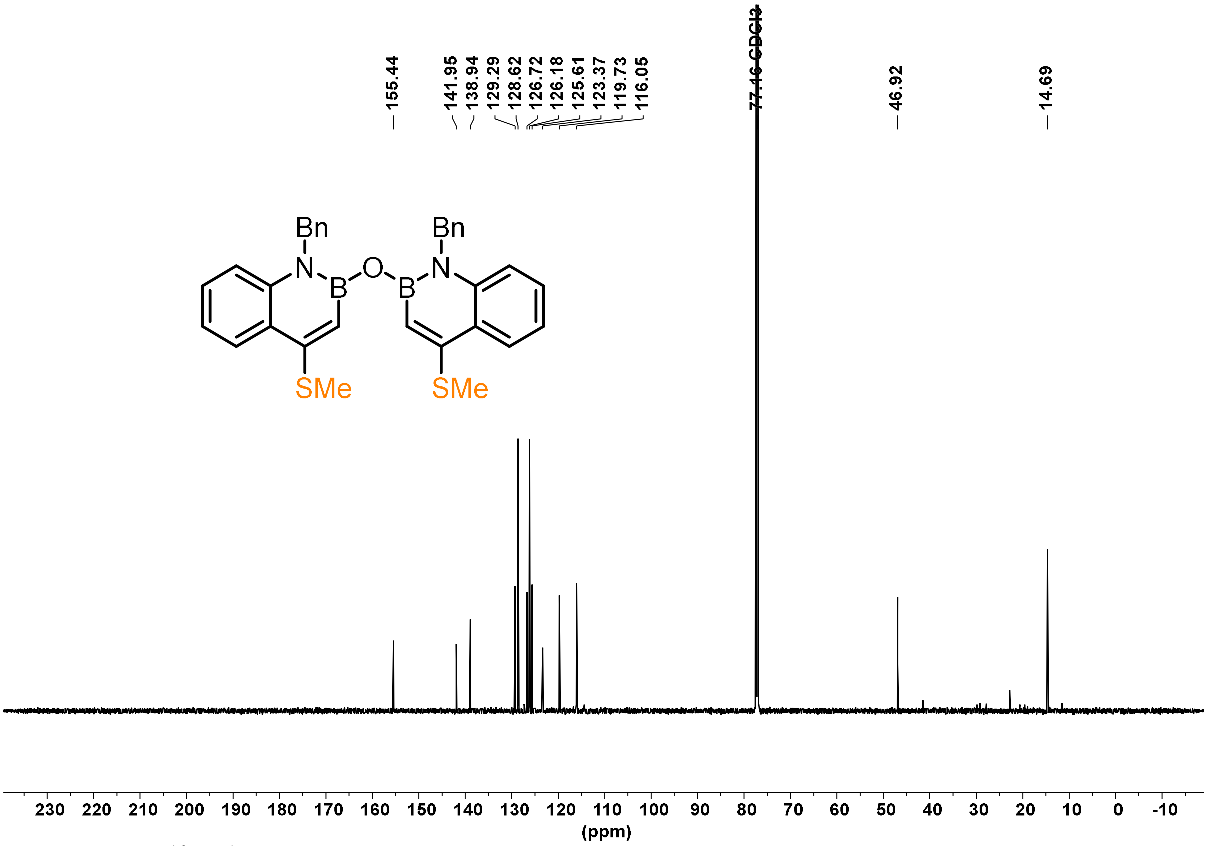

Figure S92. $^{13}C\{^1H\}$ NMR spectrum of compound **4a-anhydride** in $CDCl_3$.

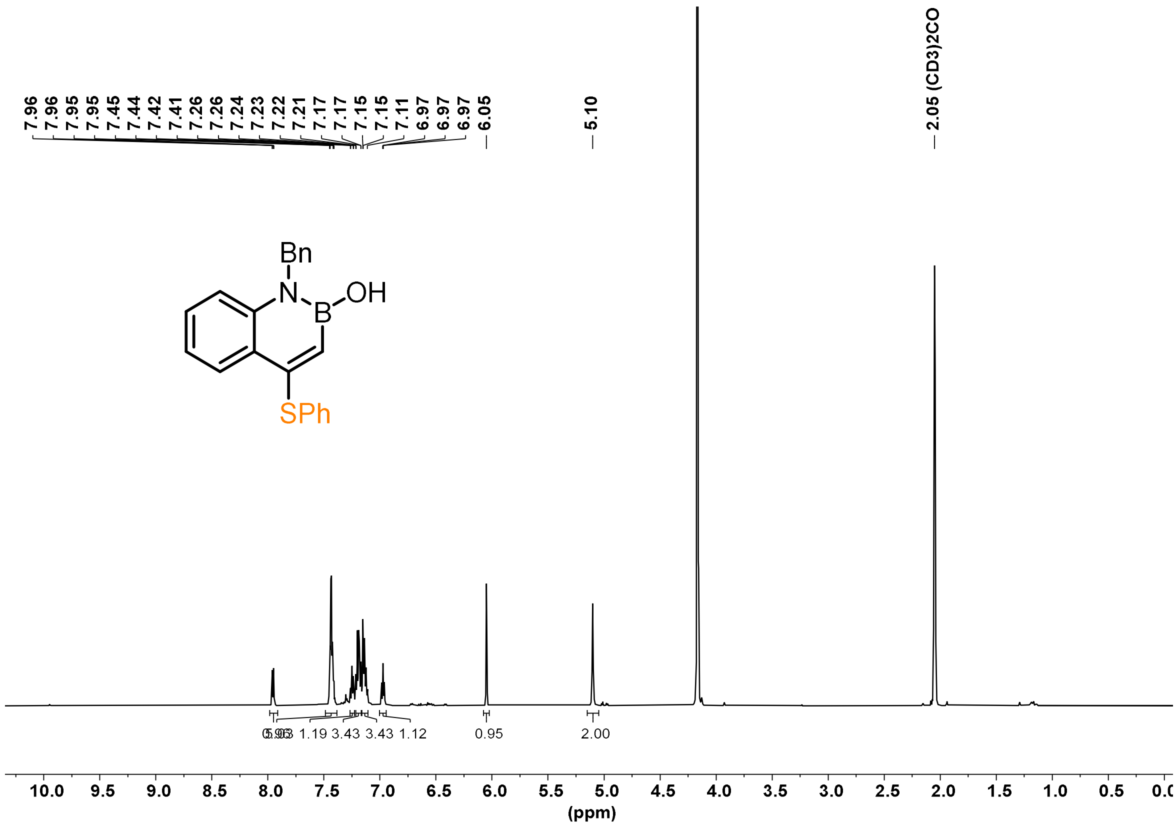

Figure S93. ^{1}H NMR spectrum of compound **4b** in acetone-D_6 + D_2O.

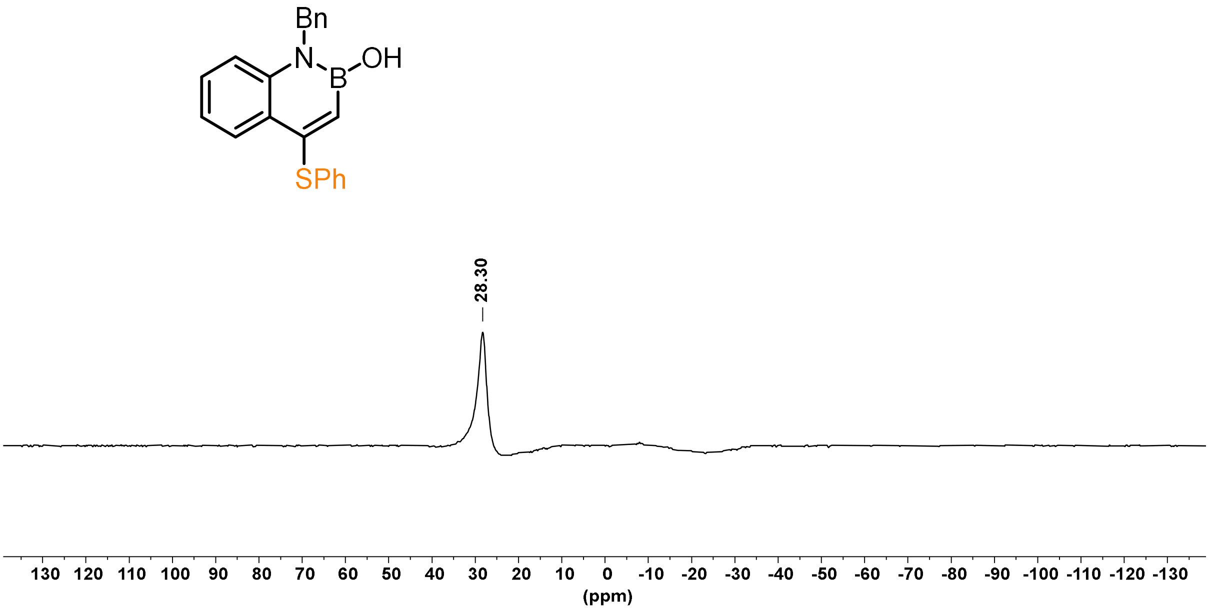

Figure S94. ^{11}B NMR spectrum of compound **4b** in acetone-D_6 + D_2O.

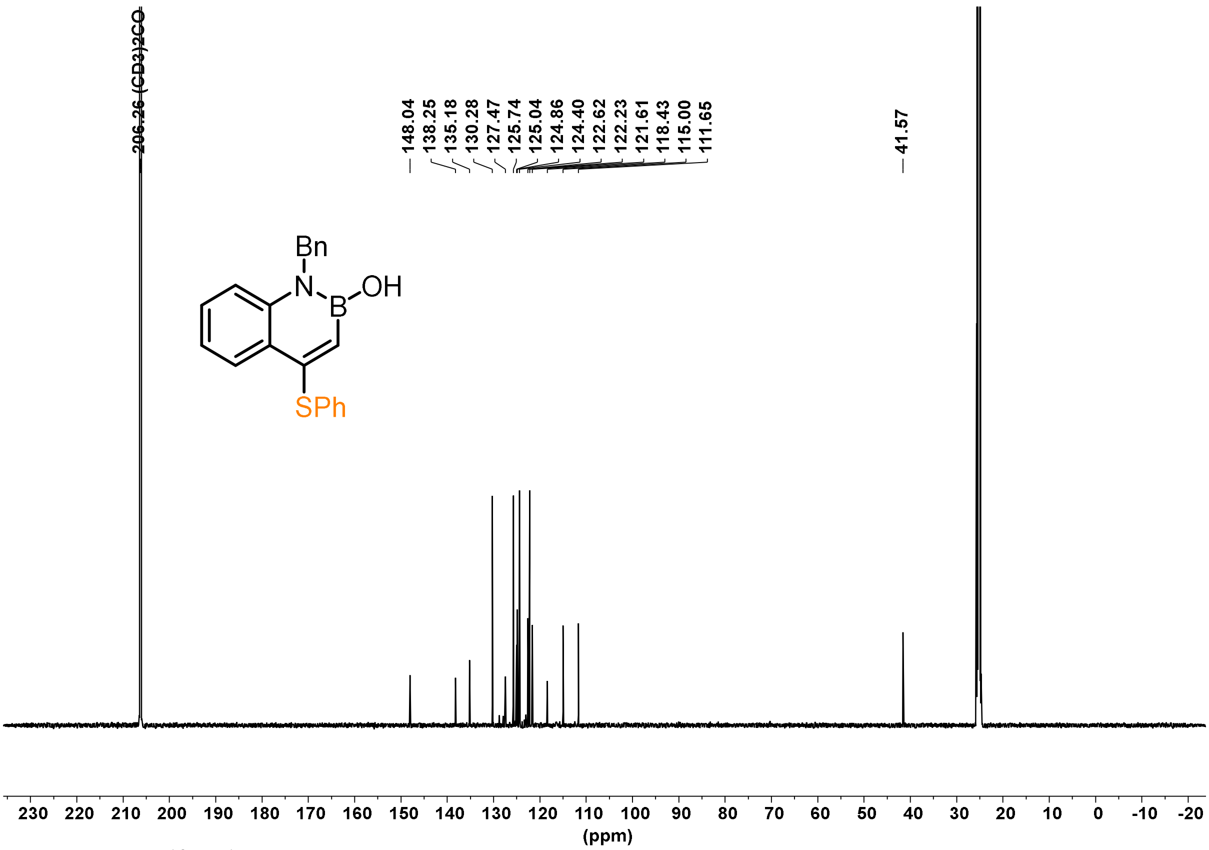

Figure S95. $^{13}C\{^{1}H\}$ NMR spectrum of compound **4b** in acetone-D_6 + D_2O.

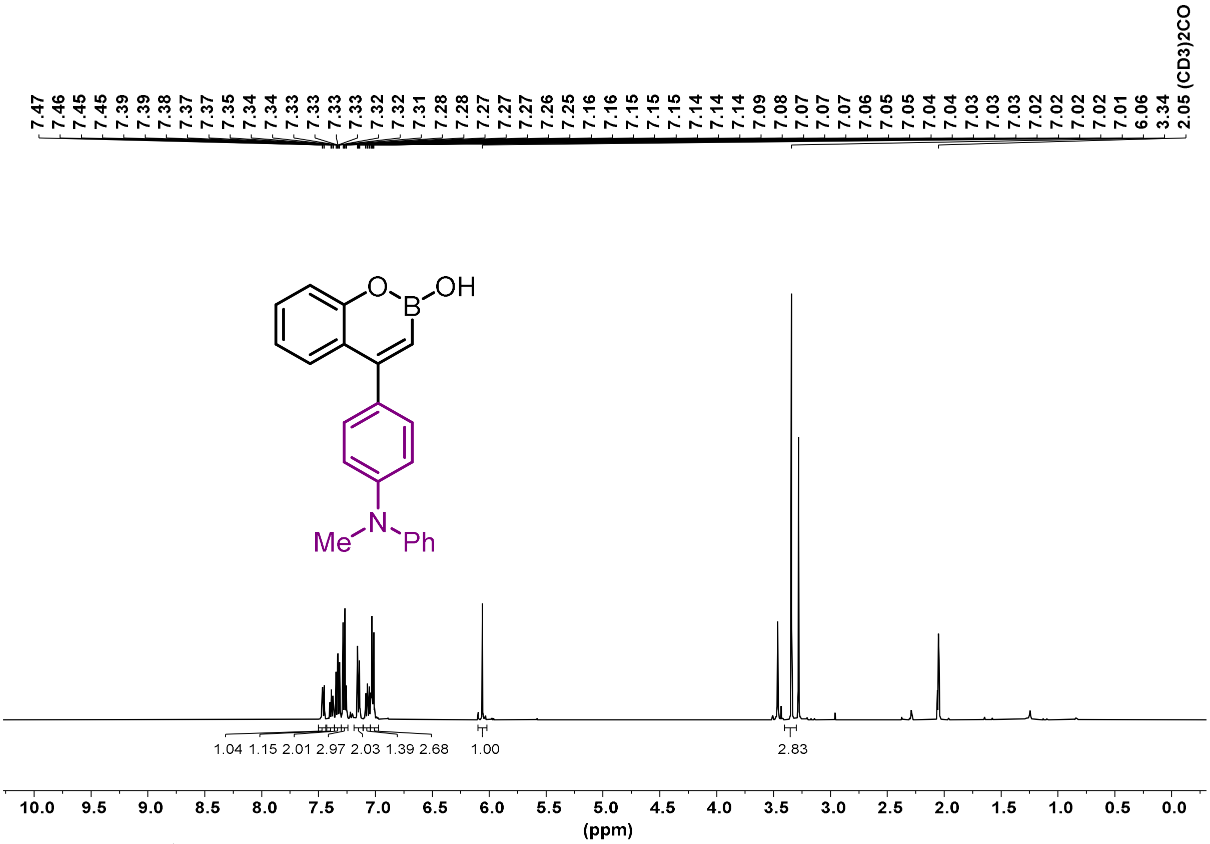

Figure S96. ^{1}H NMR spectrum of compound **5a** in acetone-D_6 + 40 uL D_2O.

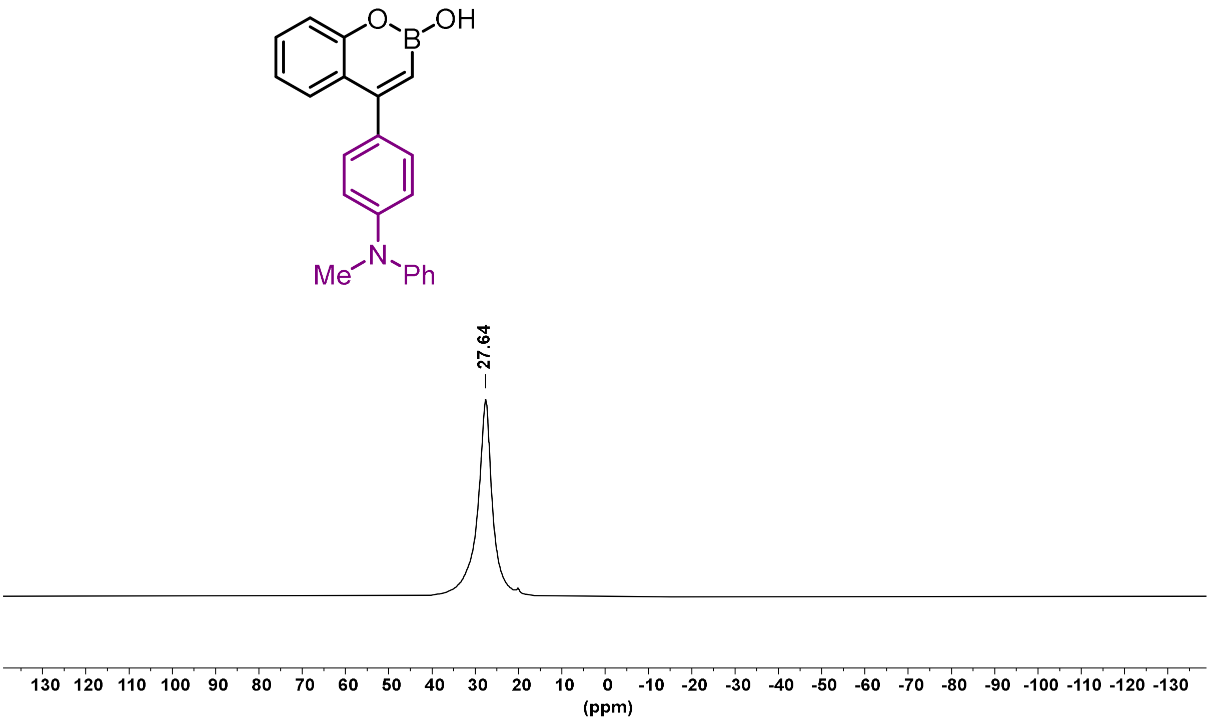

Figure S97. ^{11}B NMR spectrum of compound **5a** in acetone-D_6.

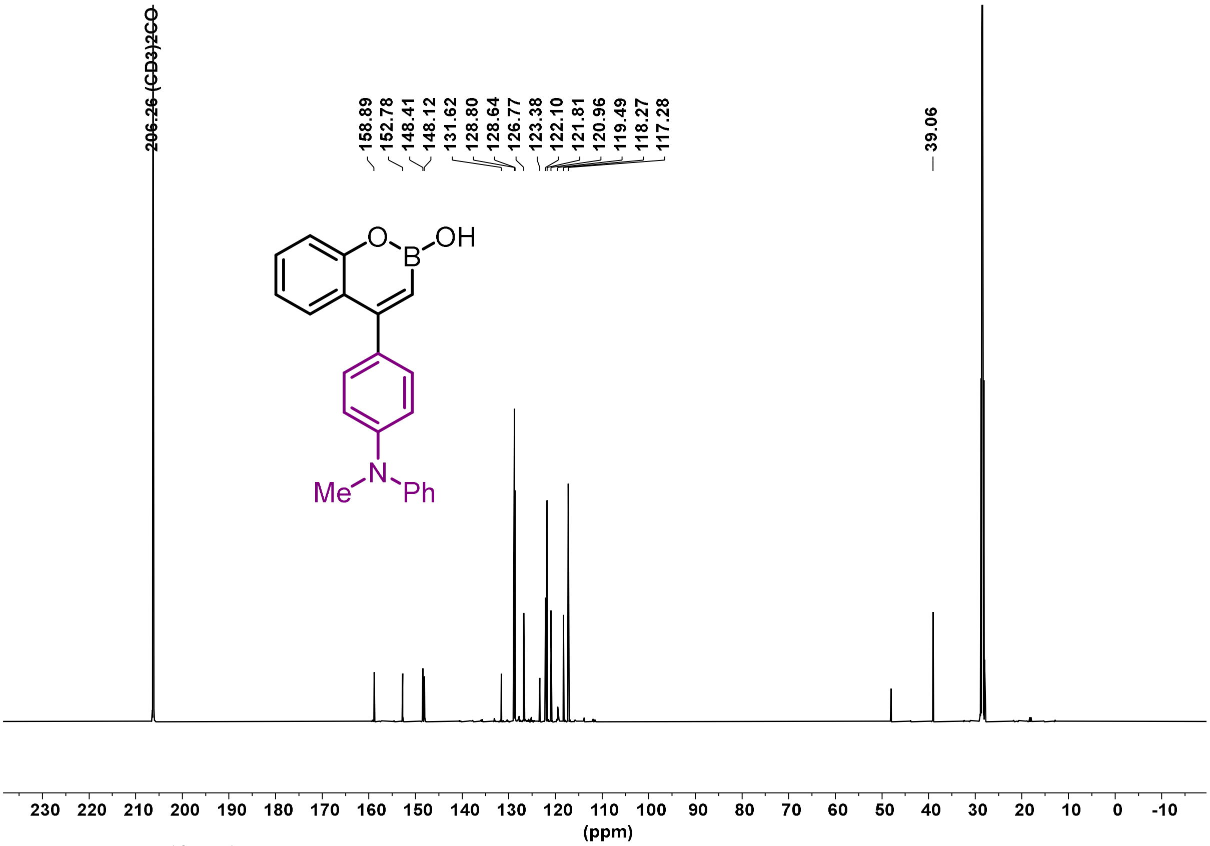

Figure S98. $^{13}C\{^{1}H\}$ NMR spectrum of compound **5a** in acetone-D_6 + 40 uL D_2O.

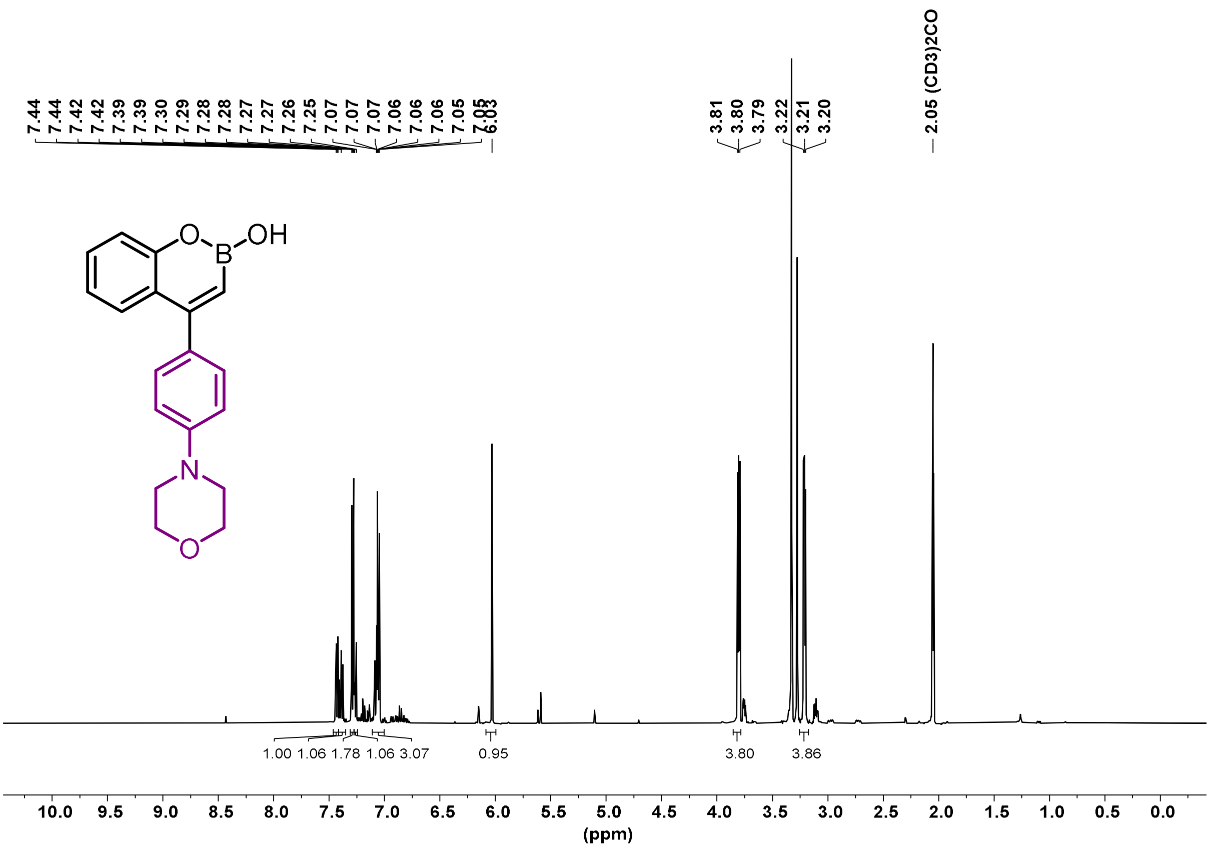

Figure S99. ^{1}H NMR spectrum of compound **5b** in acetone-D_6 + 40 uL D_2O.

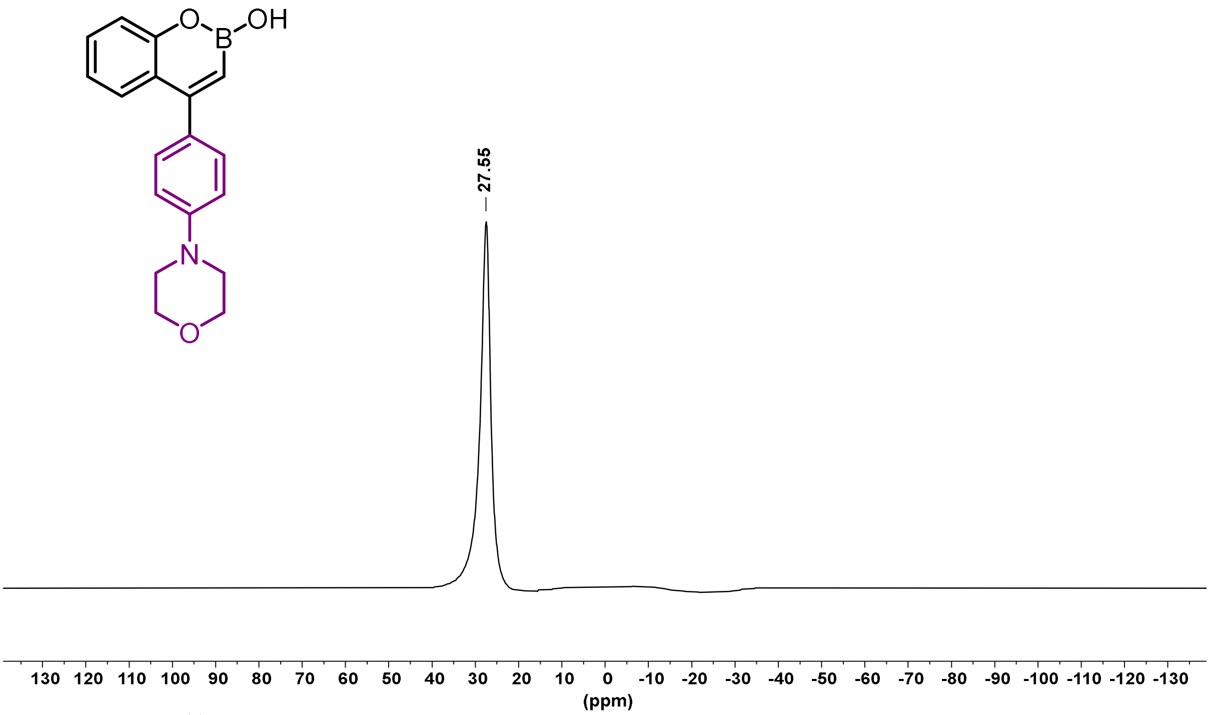

Figure S100. ^{11}B NMR spectrum of compound **5b** in acetone-D_6.

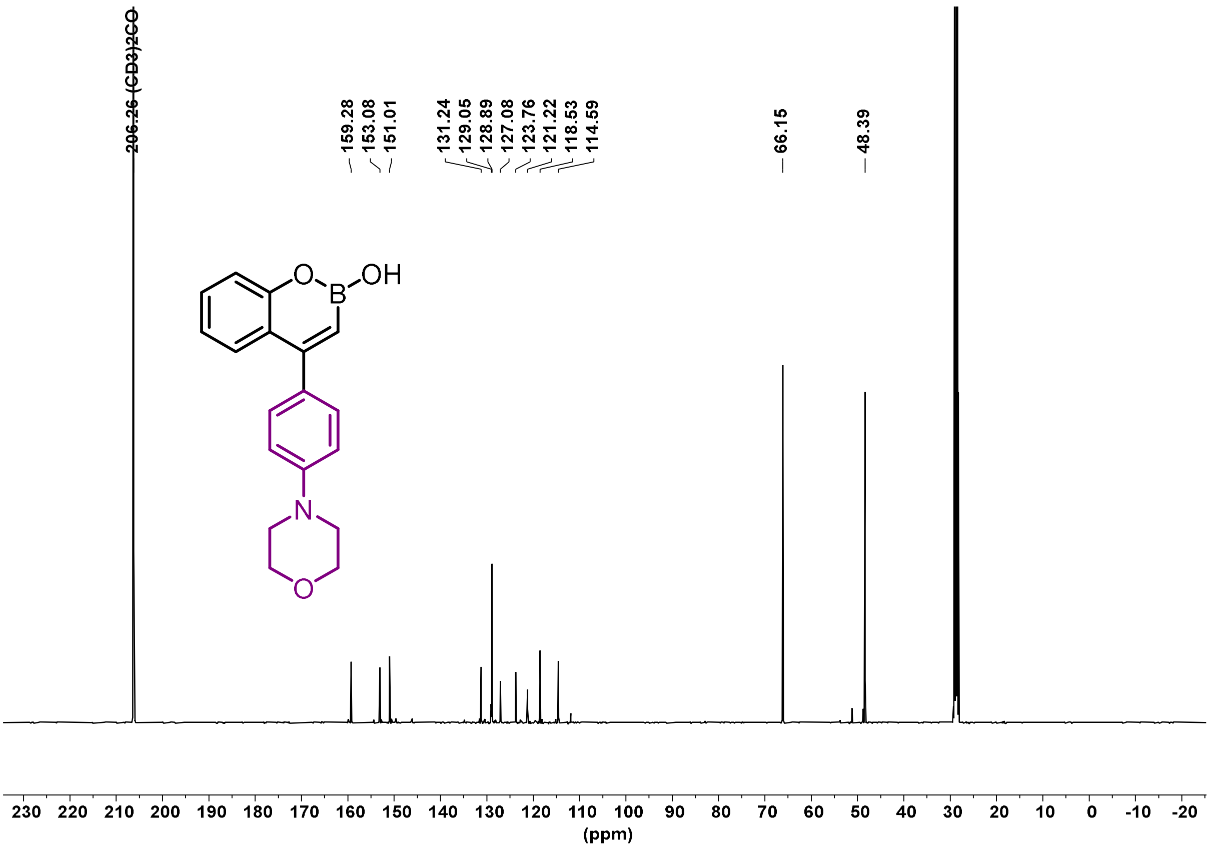

Figure S101. $^{13}C\{^1H\}$ NMR spectrum of compound **5b** in acetone-D_6 + 40 uL D_2O.

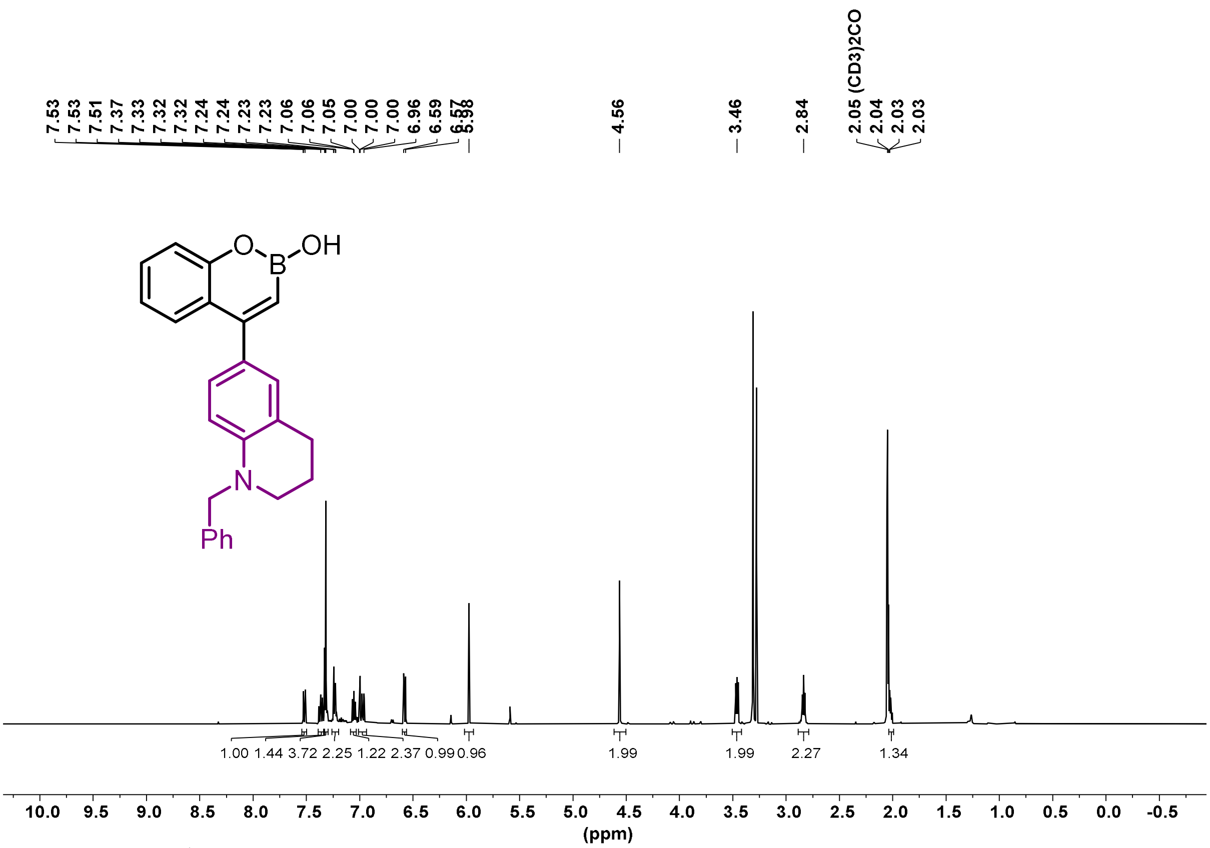

Figure S102. ^1H NMR spectrum of compound **5c** in acetone-D_6 + 40 uL D_2O.

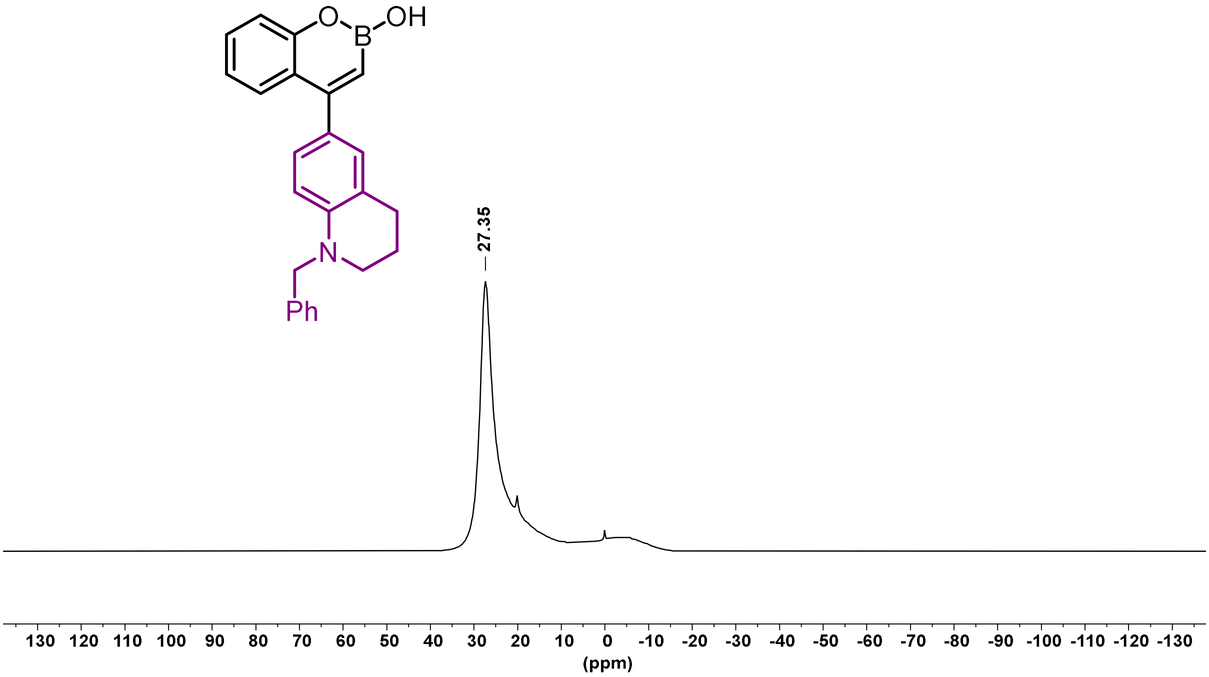

Figure S103. ^{11}B NMR spectrum of compound **5c** in acetone-D$_6$.

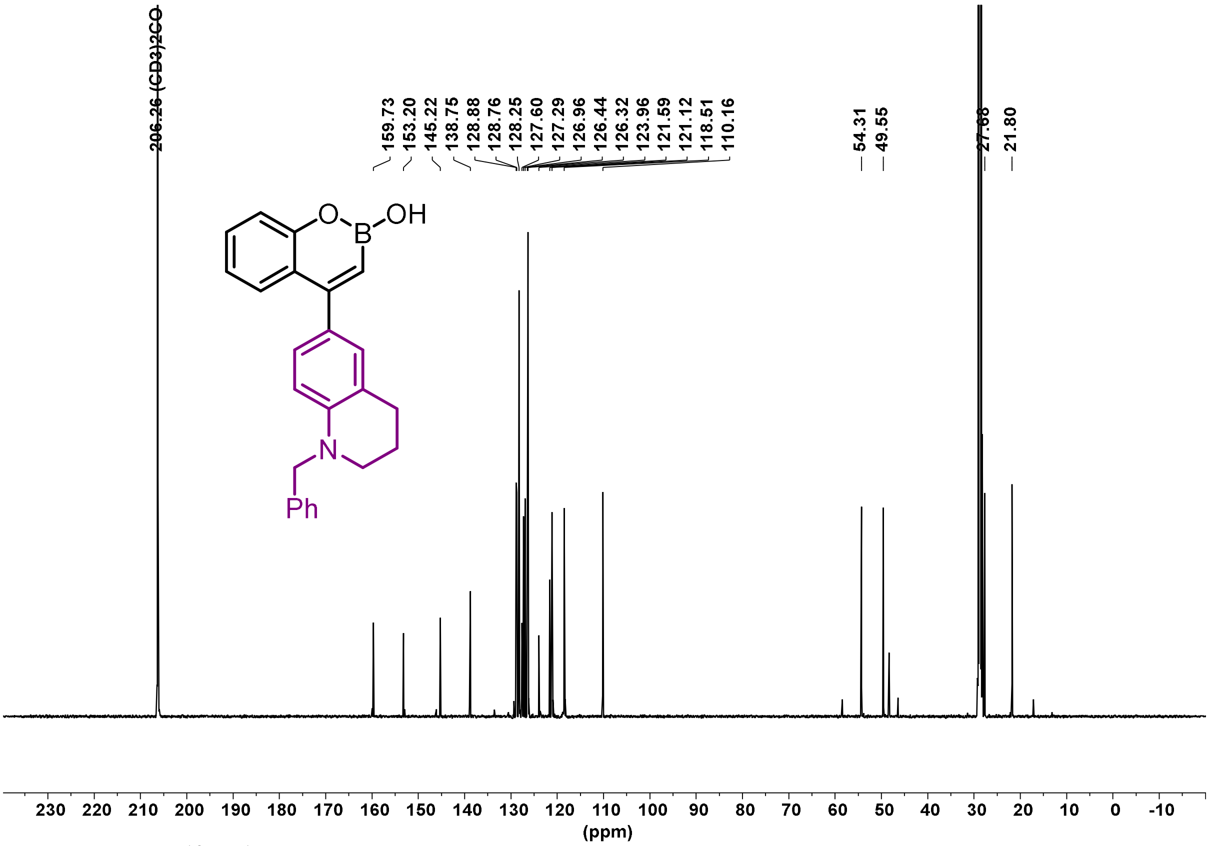

Figure S104. ^{13}C{^{1}H} NMR spectrum of compound **5c** in acetone-D$_6$ + 40 uL D$_2$O.

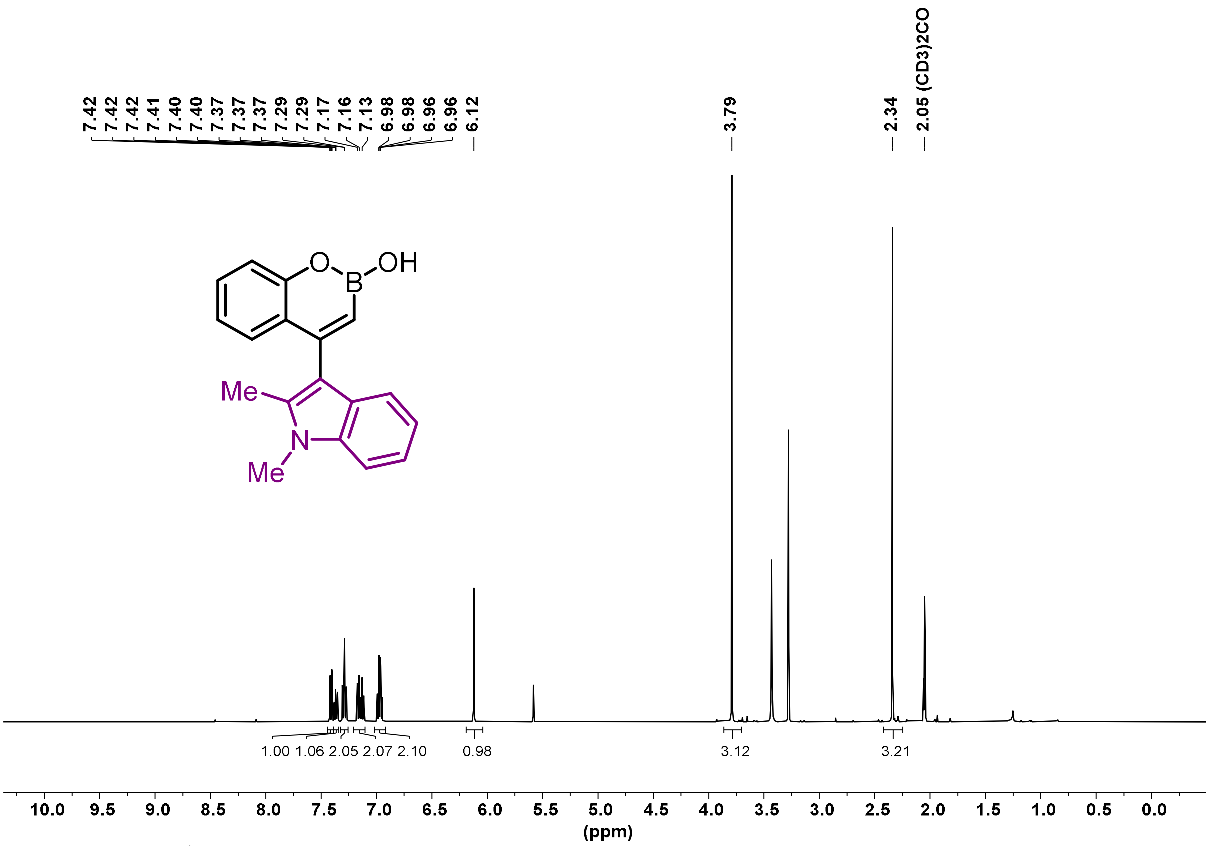

Figure S105. ^{1}H NMR spectrum of compound **5d** in acetone-D_6 + 40 uL D_2O.

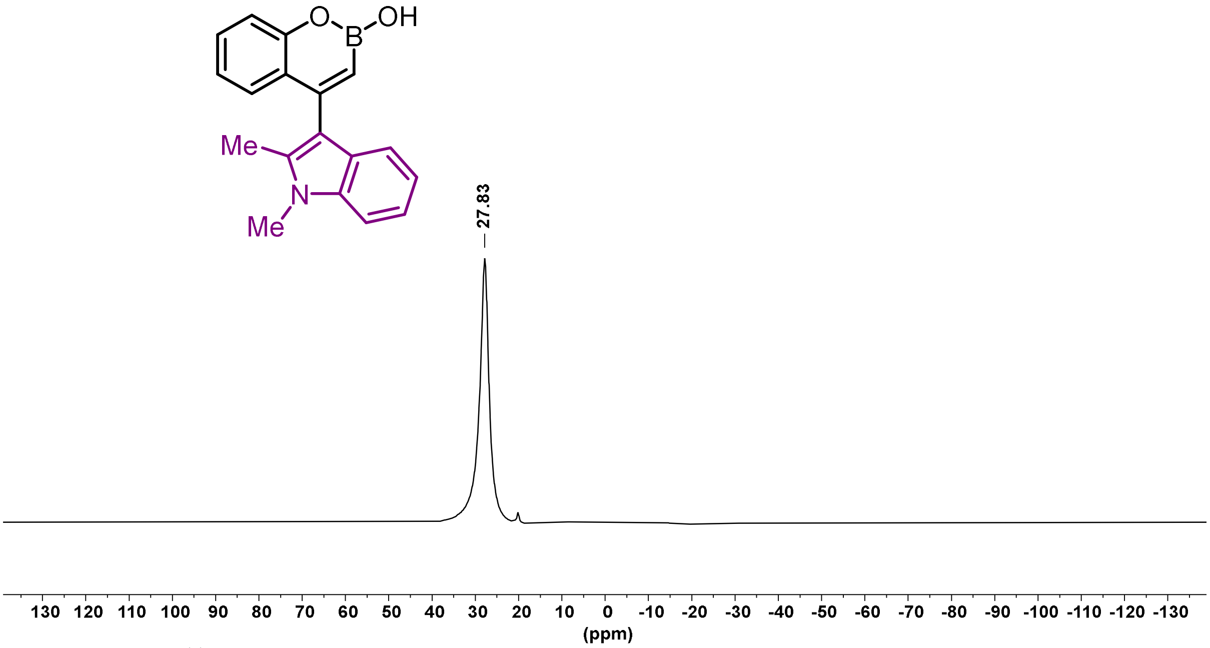

Figure S106. ^{11}B NMR spectrum of compound **5d** in acetone-D_6.

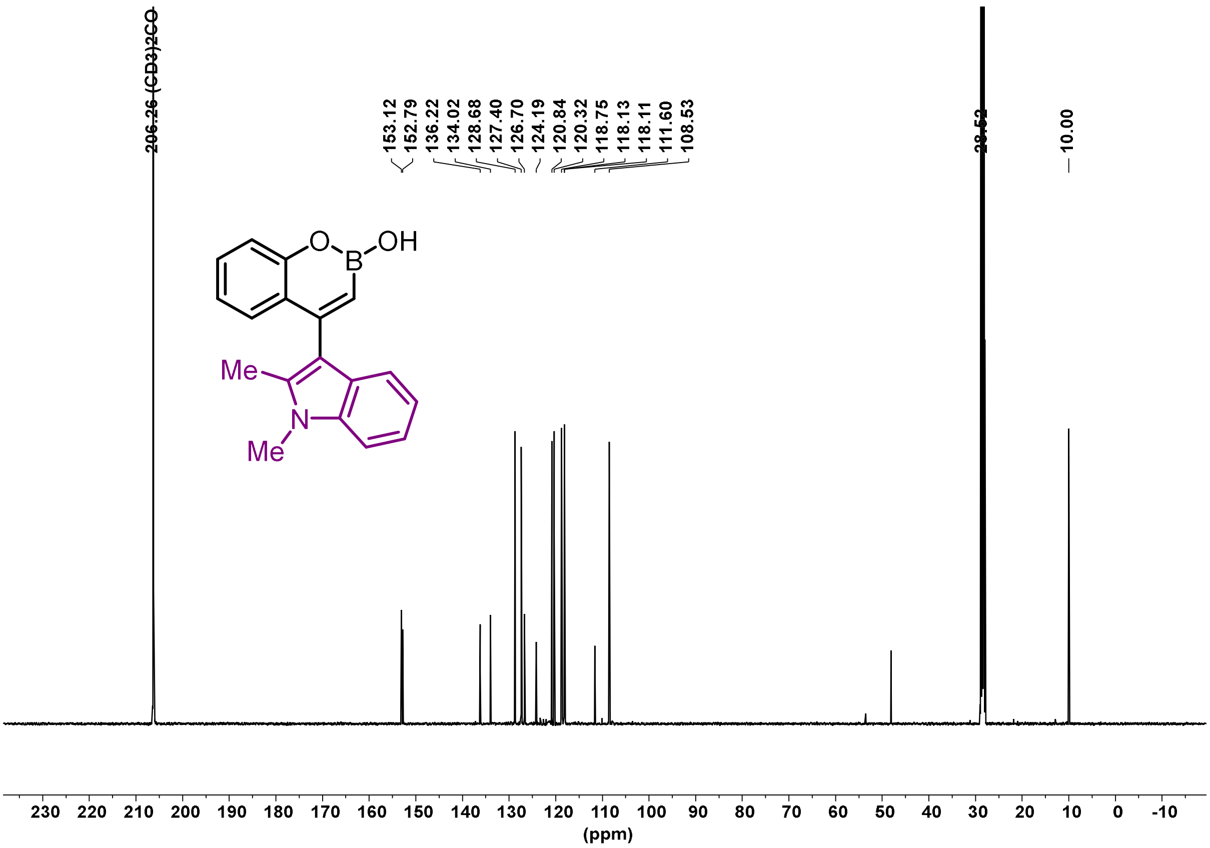

Figure S107. $^{13}C\{^{1}H\}$ NMR spectrum of compound **5d** in acetone-D_6 + 40 uL D_2O.

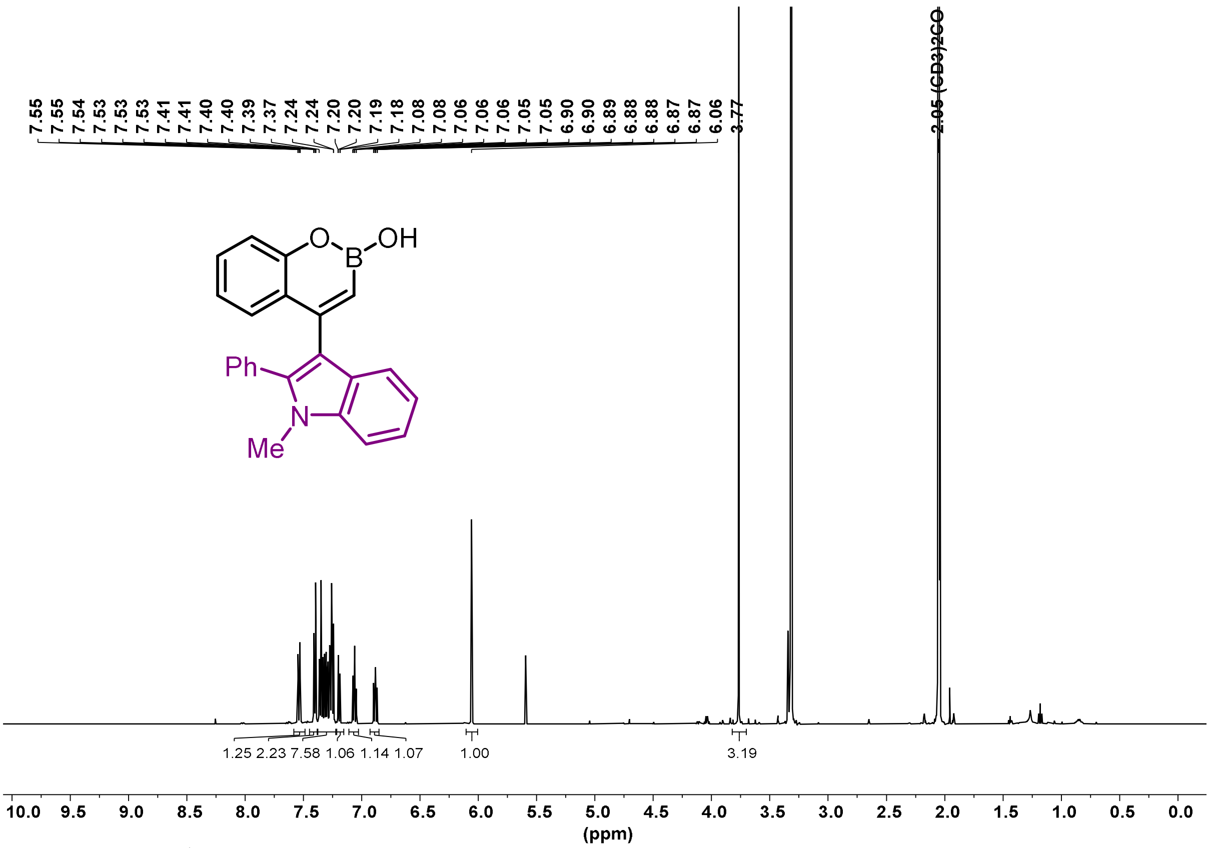

Figure S108. ^{1}H NMR spectrum of compound **5e** in acetone-D_6 + 40 uL D_2O.

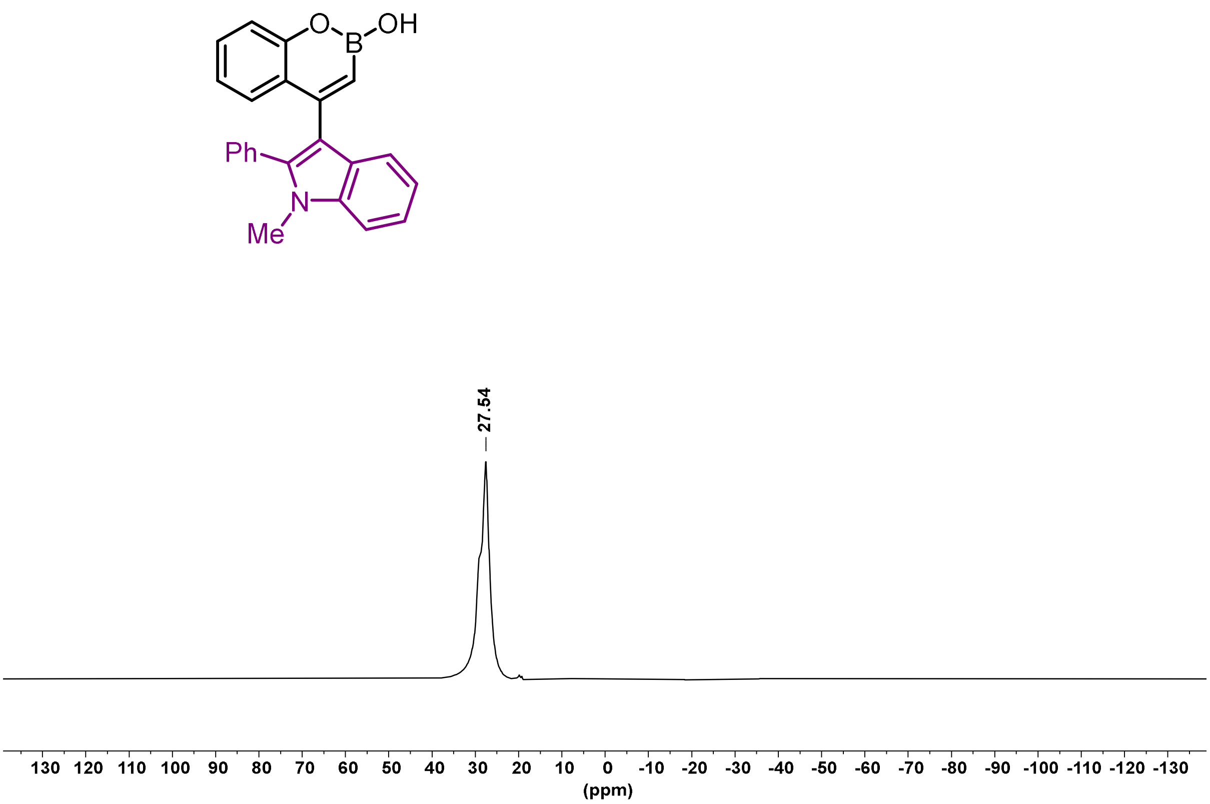

Figure S109. ^{11}B NMR spectrum of compound **5e** in acetone-D_6.

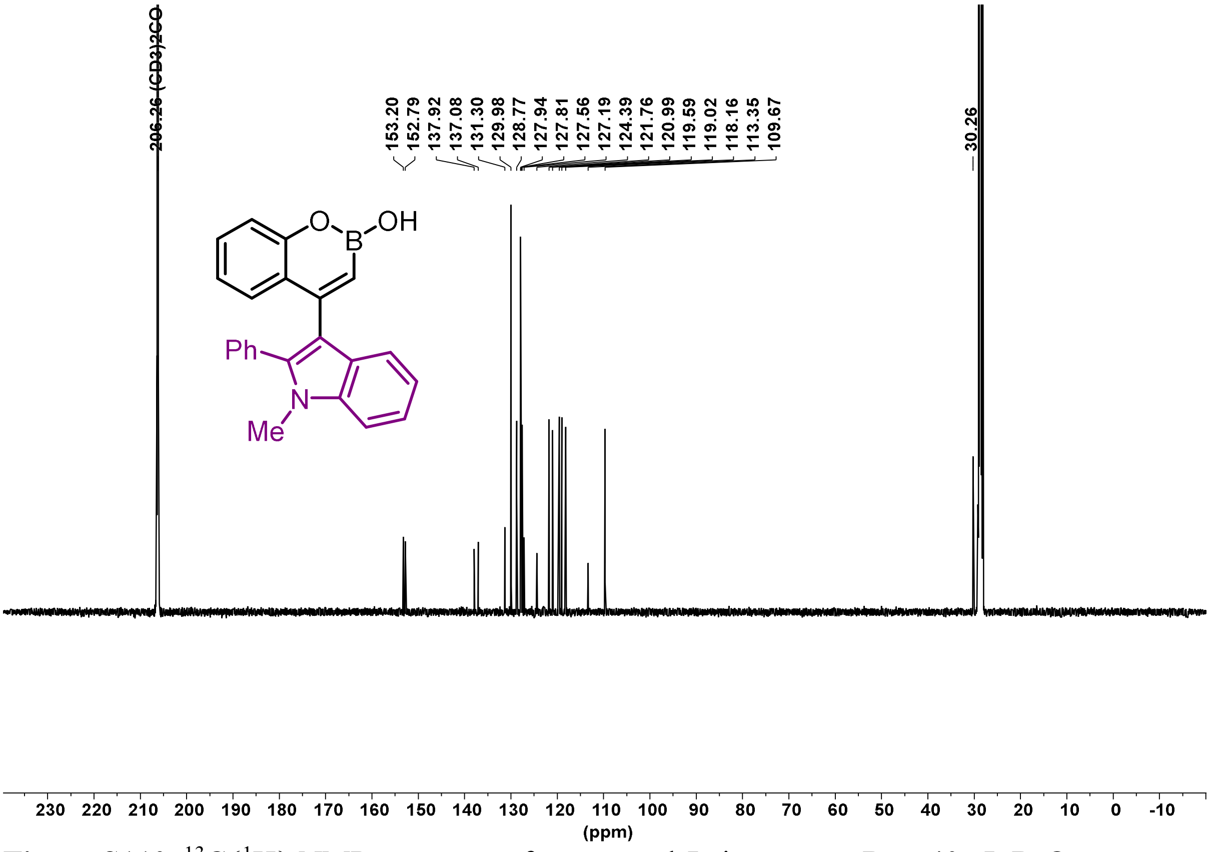

Figure S110. $^{13}C\{^1H\}$ NMR spectrum of compound **5e** in acetone-D_6 + 40 uL D_2O.

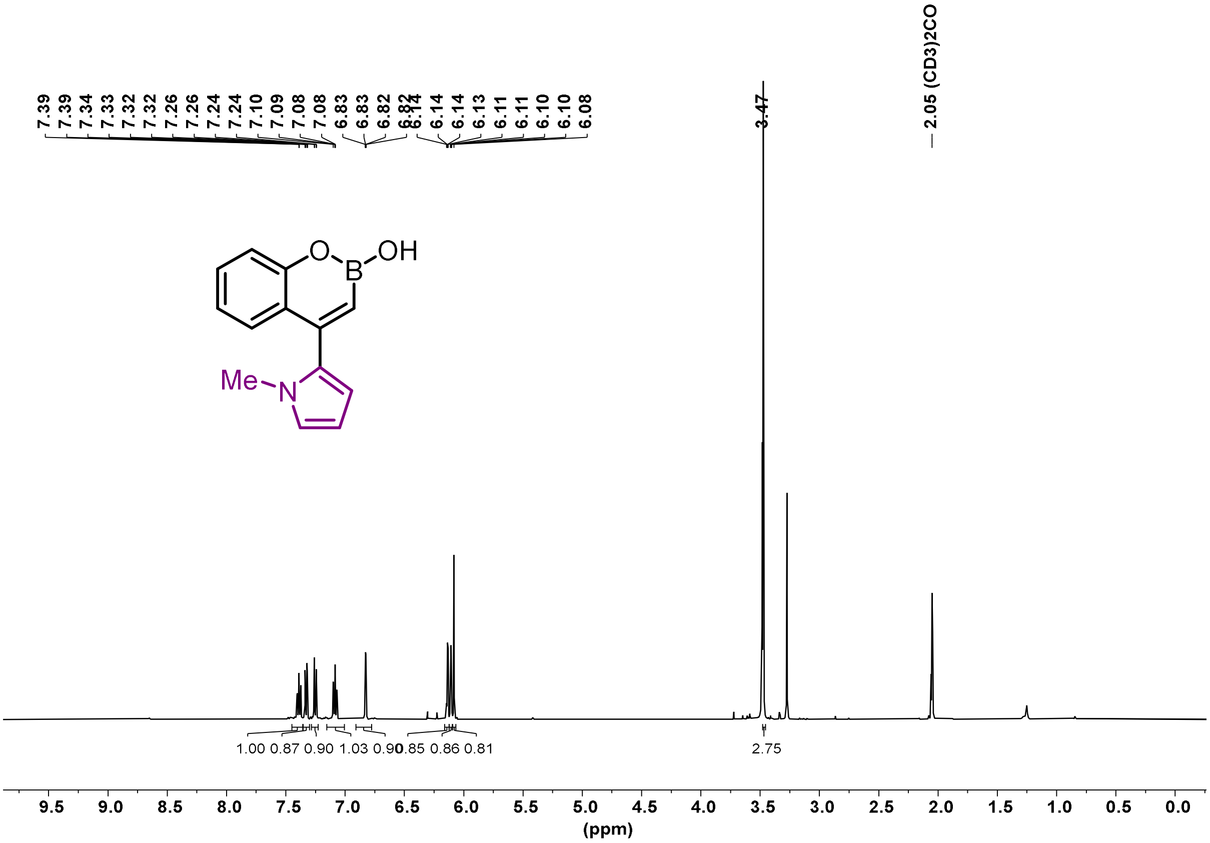

Figure S111. ^{1}H NMR spectrum of compound **5f** in acetone-D_6 + 40 uL D_2O.

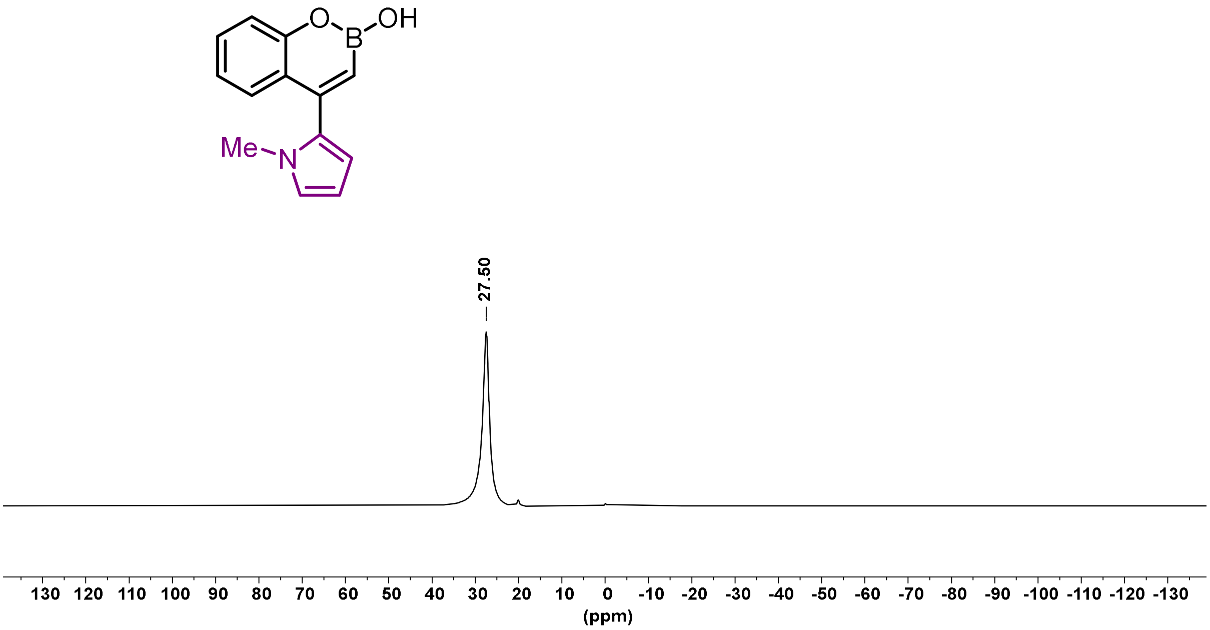

Figure S112. ^{11}B NMR spectrum of compound **5f** in acetone-D_6.

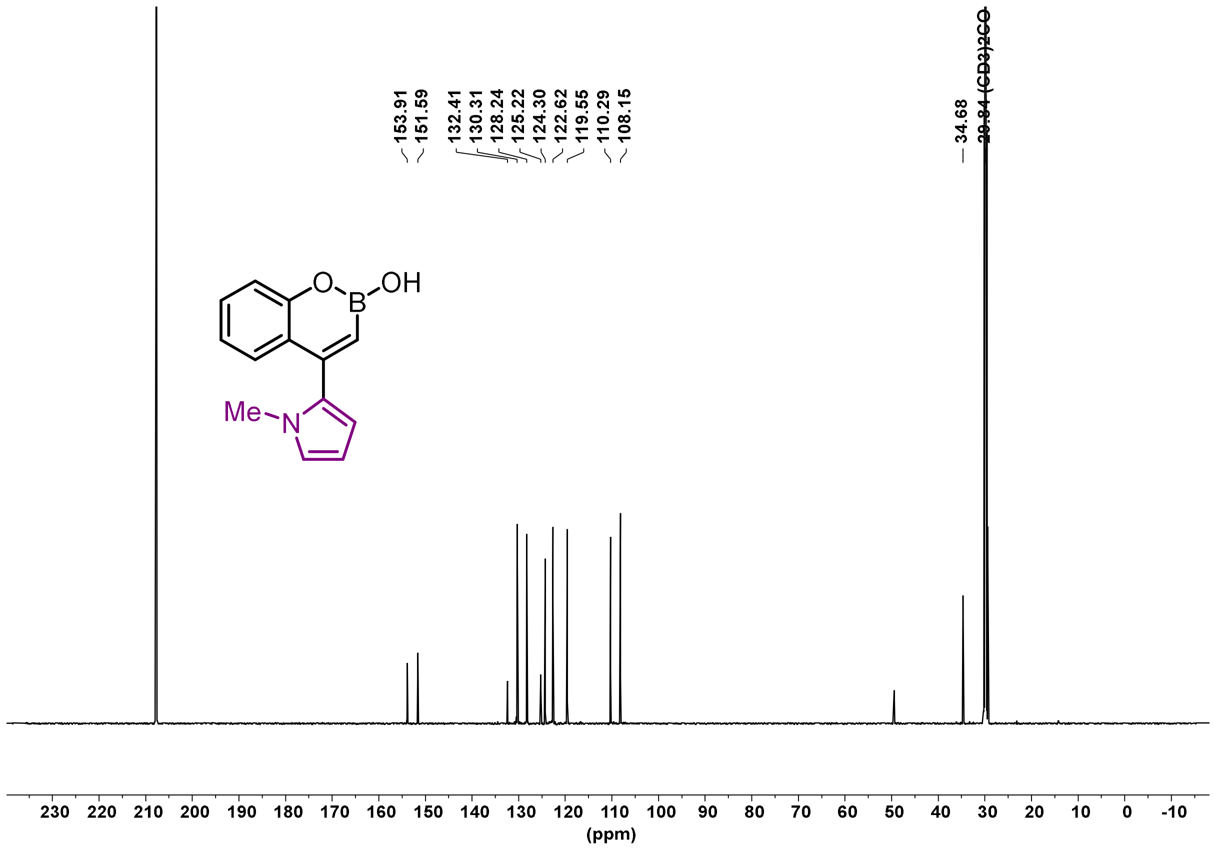

Figure S113. $^{13}C\{^{1}H\}$ NMR spectrum of compound **5f** in acetone-D_6 + 40 uL D_2O.

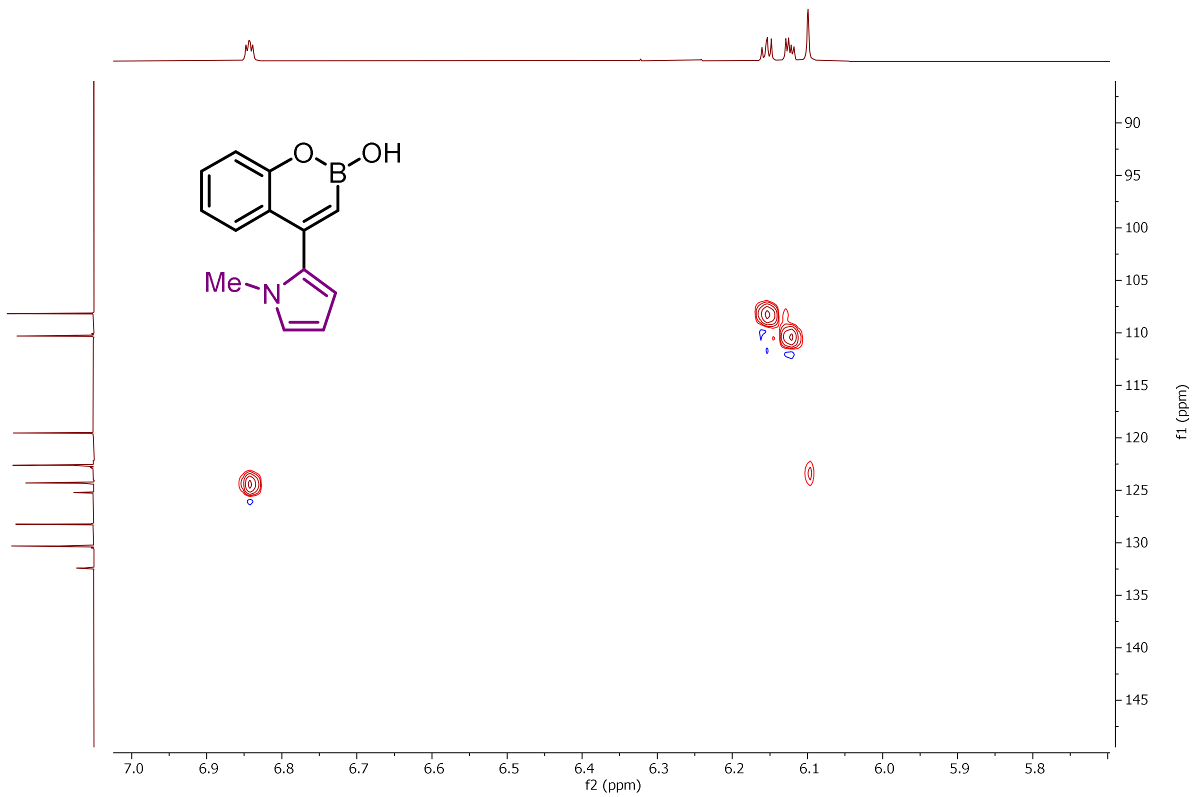

Figure S114. HSQC showing pyrrole CH environments.

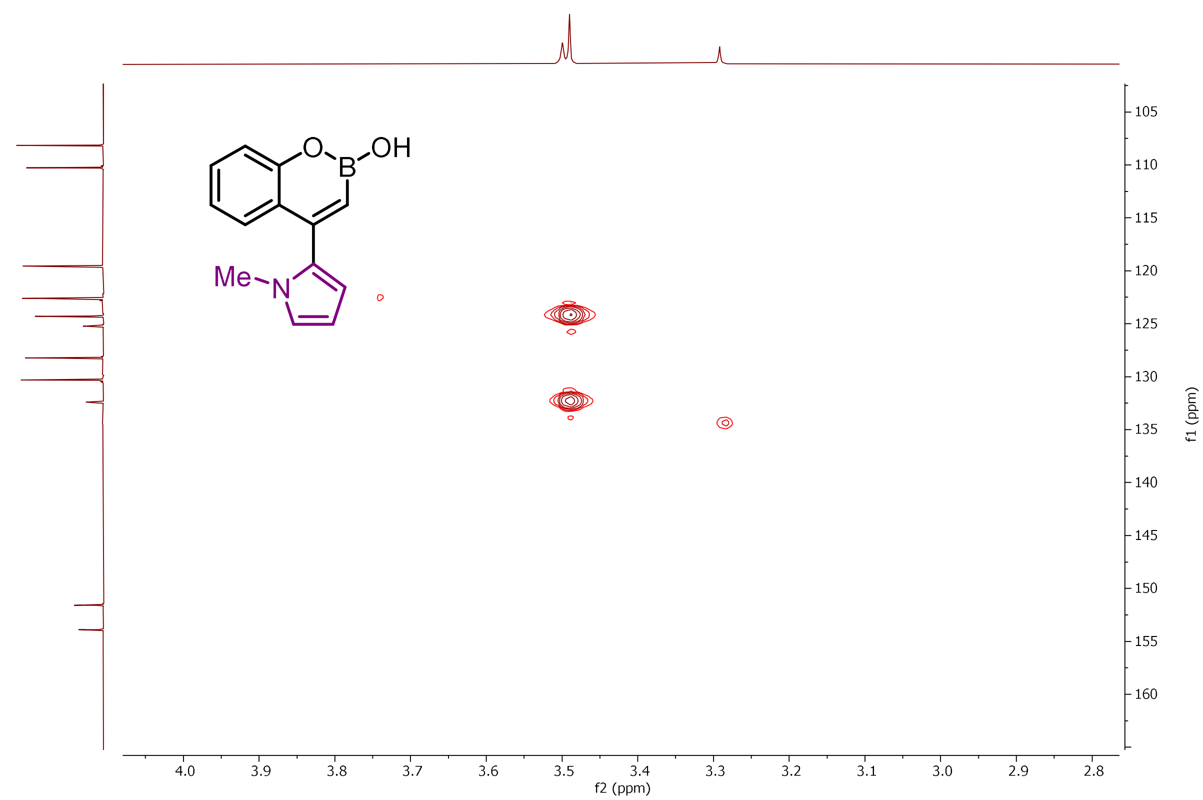

Figure S115. HMBC showing *N*-methyl association with one CH pyrrole and a quaternary C.

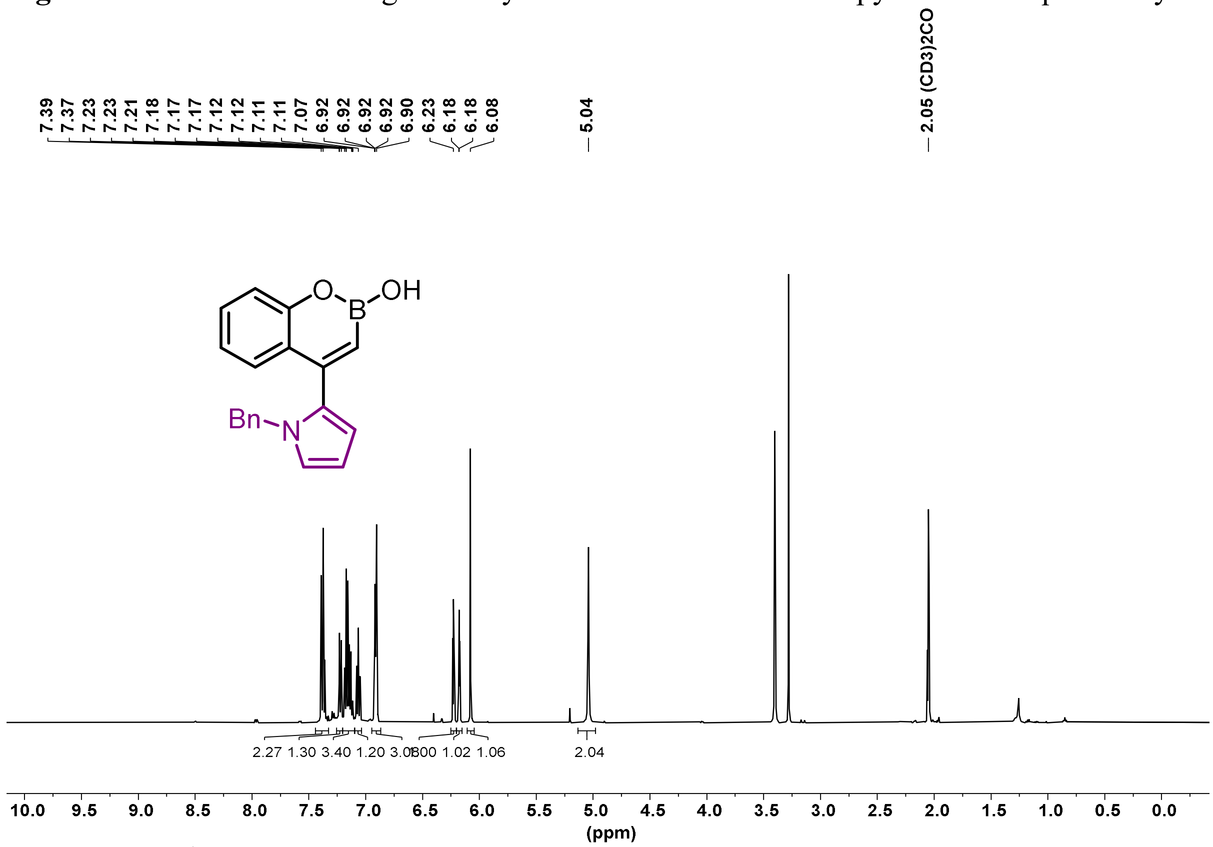

Figure S116. ^{1}H NMR spectrum of compound **5g** in acetone-D_6 + 40 uL D_2O.

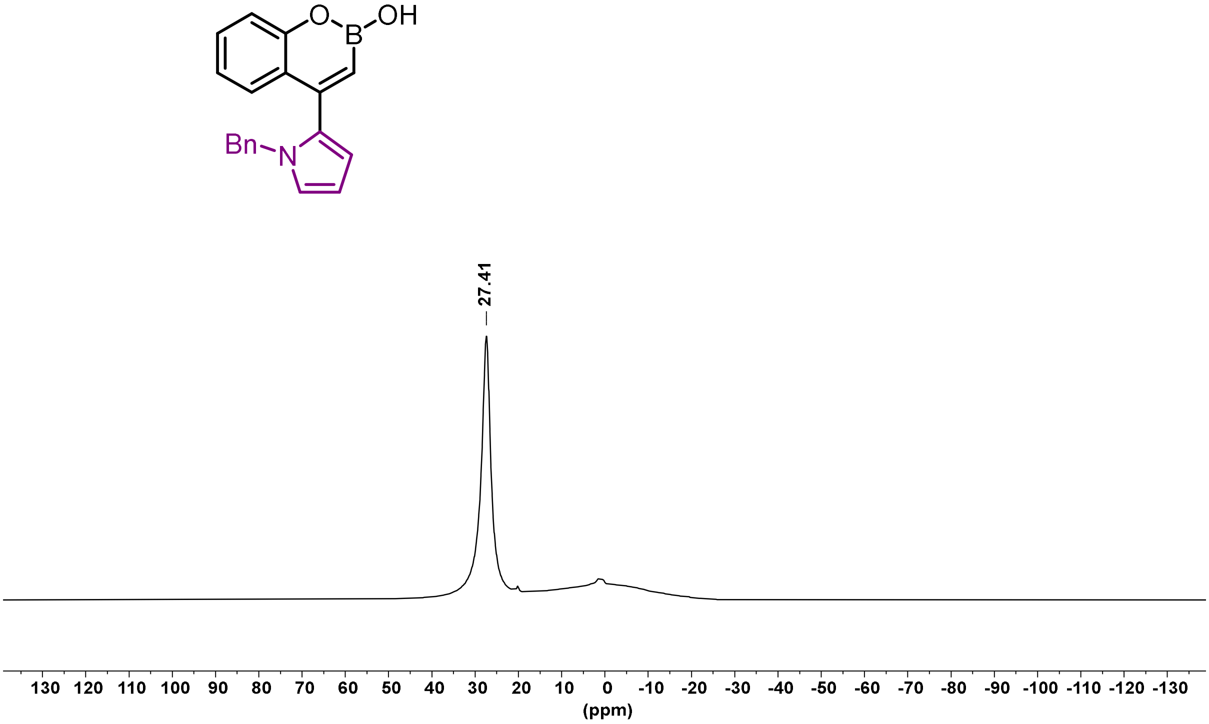

Figure S117. ^{11}B NMR spectrum of compound **5g** in acetone-D_6.

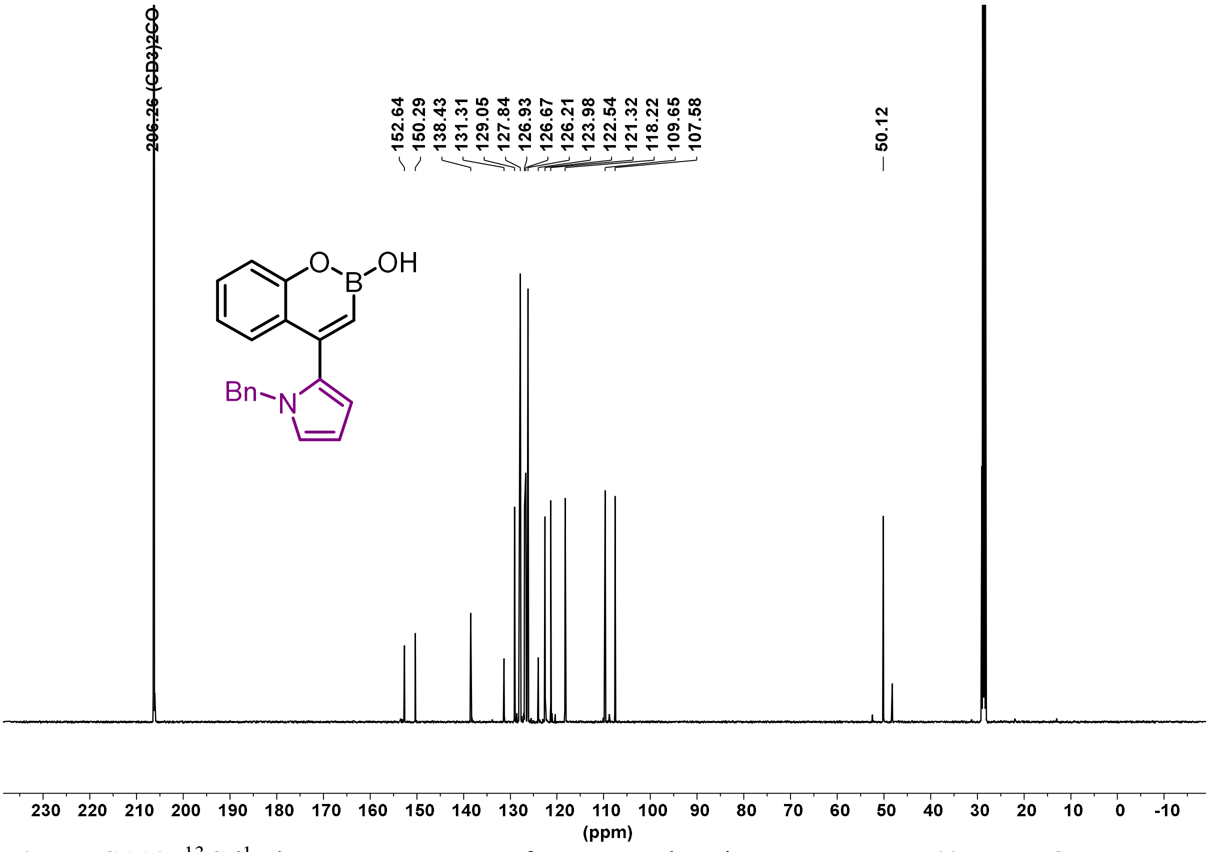

Figure S118. $^{13}C\{^1H\}$ NMR spectrum of compound **5g** in acetone-D_6 + 40 uL D_2O.

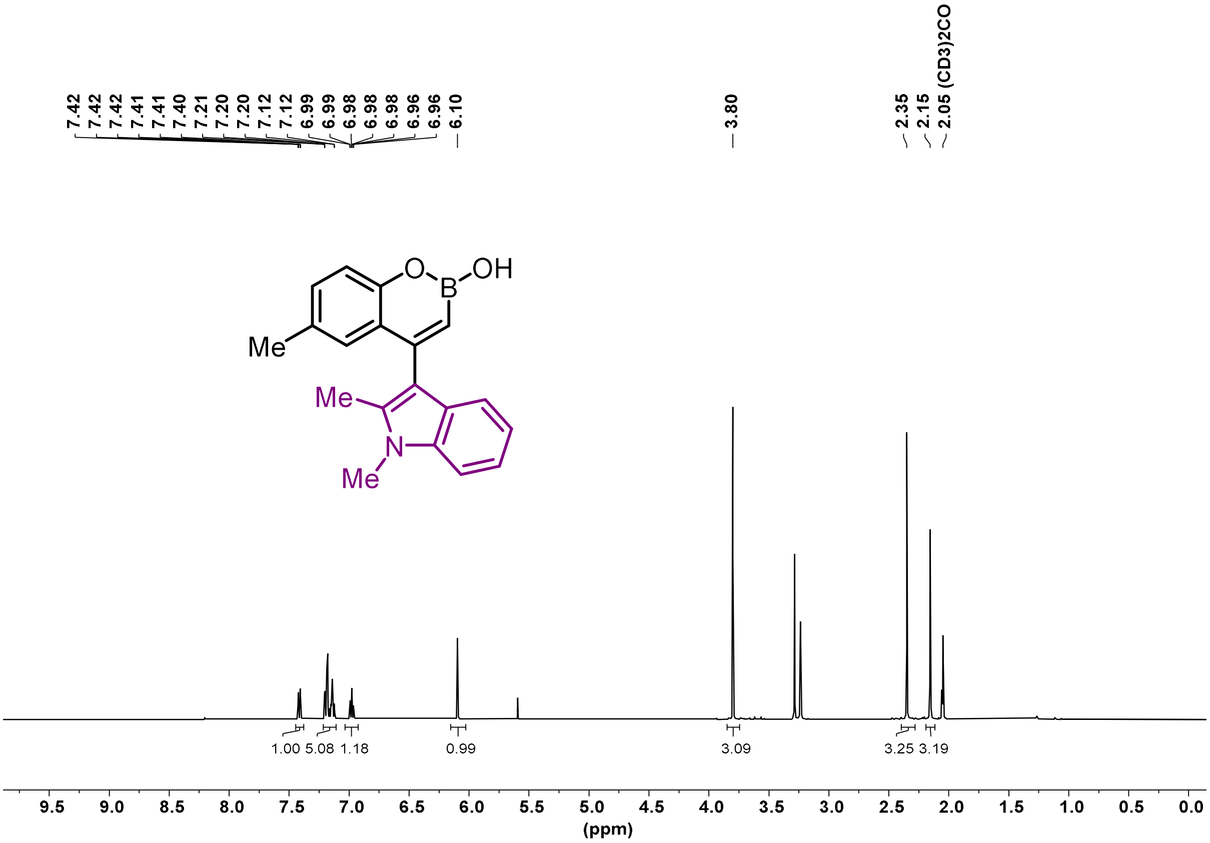

Figure S119. ^{1}H NMR spectrum of compound **5h** in acetone-D_6 + 40 uL D_2O.

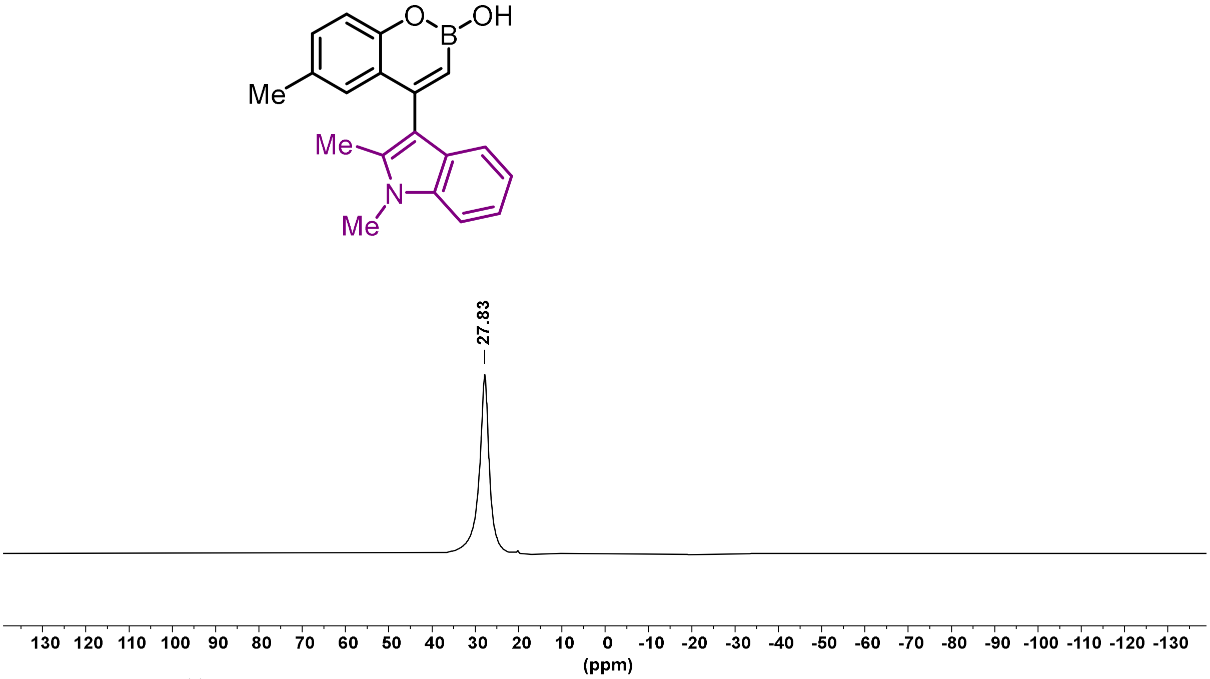

Figure S120. ^{11}B NMR spectrum of compound **5h** in acetone-D_6.

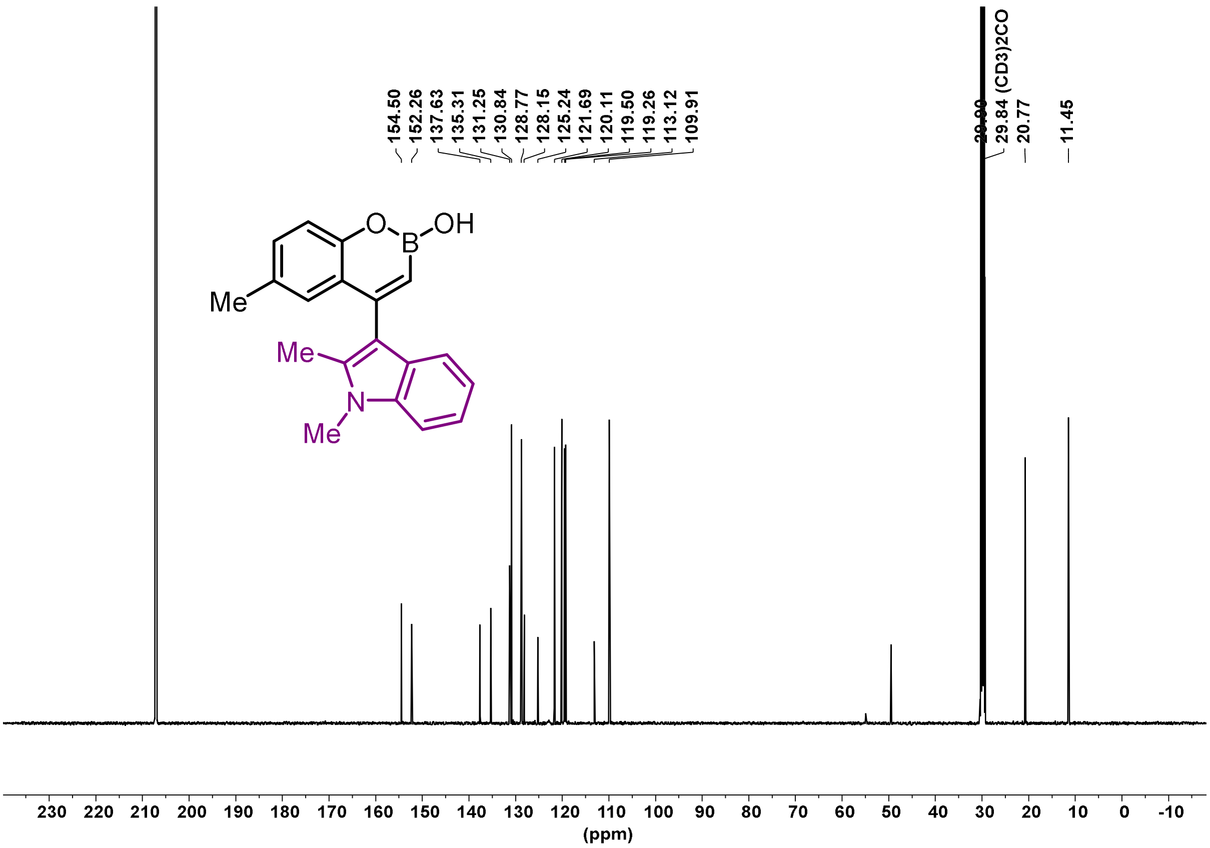

Figure S121. $^{13}C\{^{1}H\}$ NMR spectrum of compound **5h** in acetone-D_6 + 40 uL D_2O.

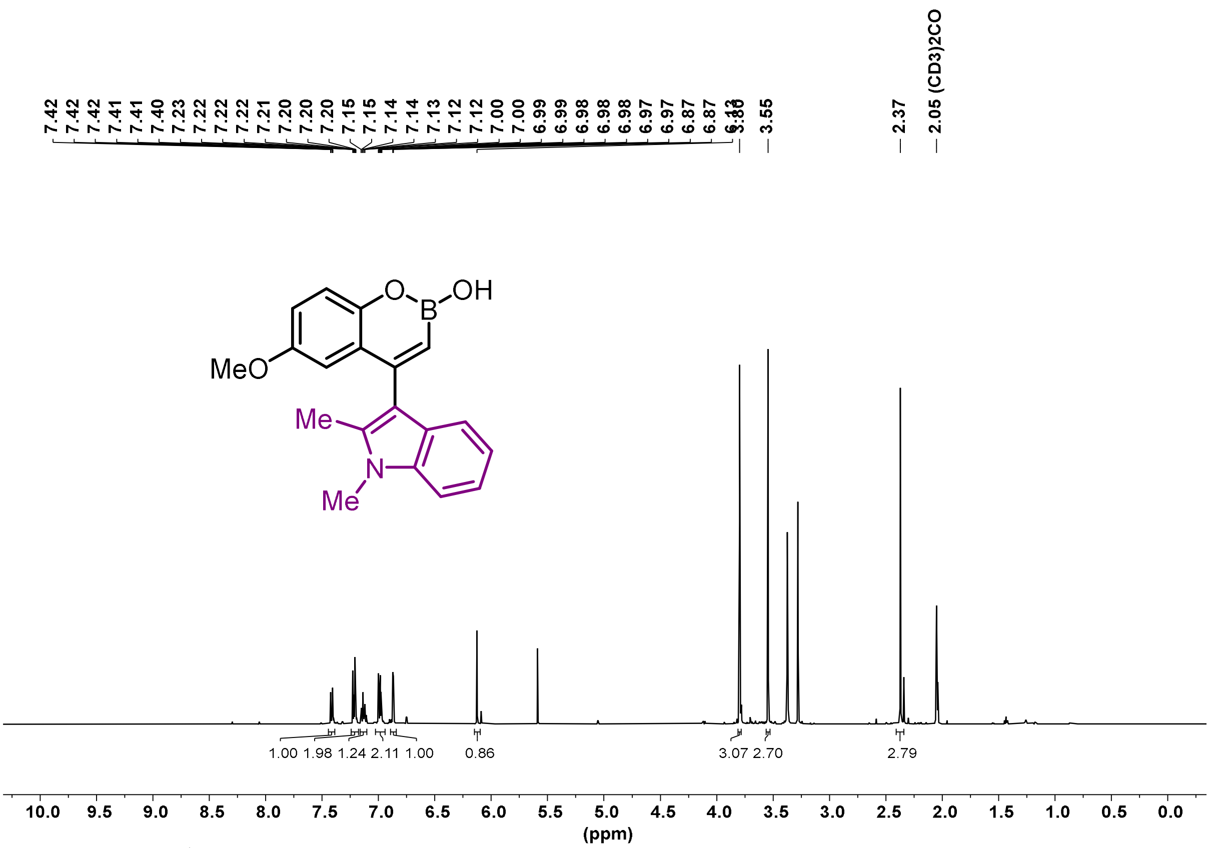

Figure S122. ^{1}H NMR spectrum of compound **5i** in acetone-D_6 + 40 uL D_2O.

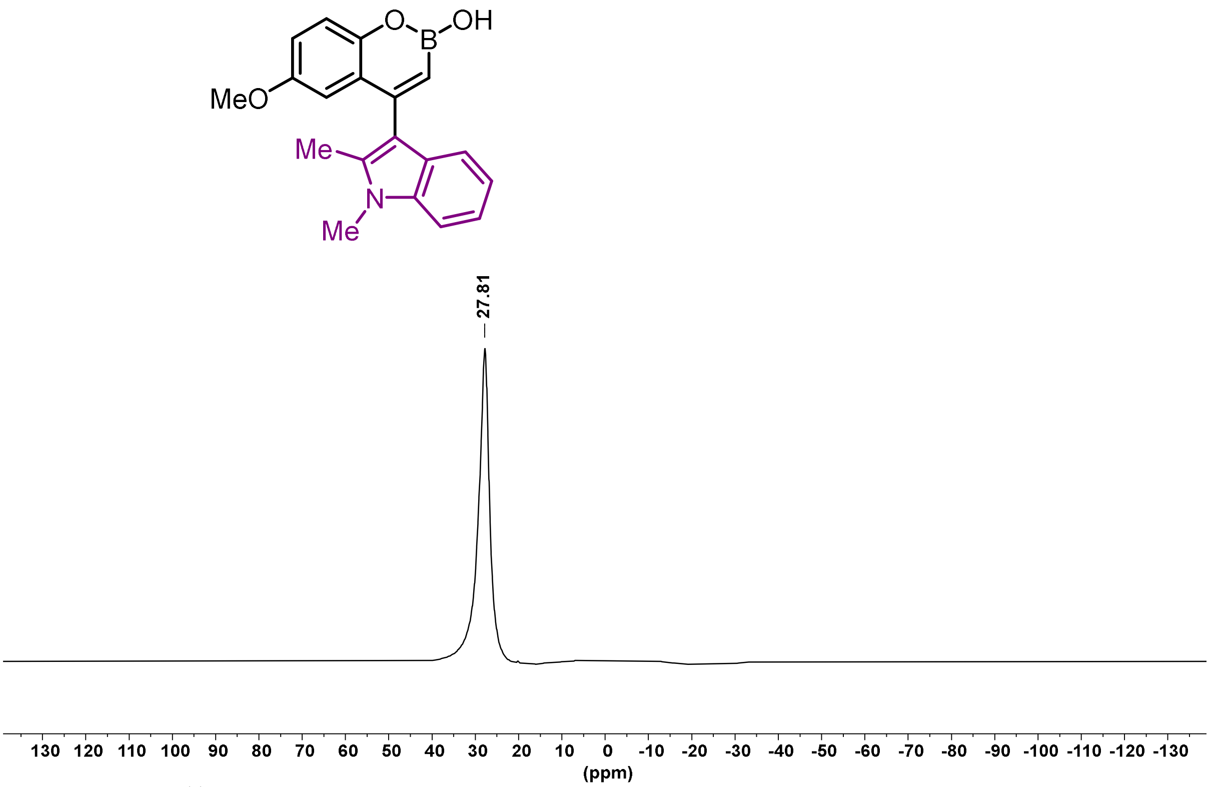

Figure S123. ^{11}B NMR spectrum of compound **5i** in acetone-D$_6$.

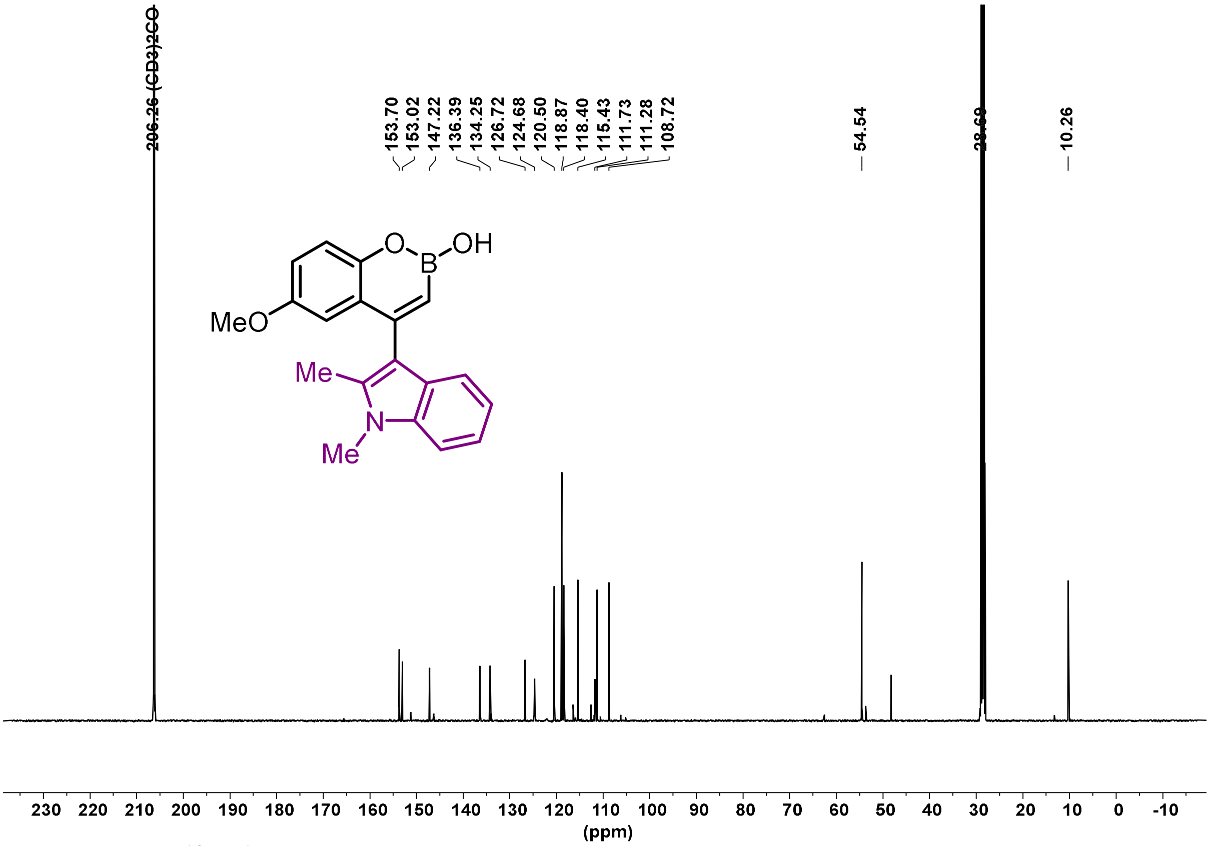

Figure S124. $^{13}C\{^1H\}$ NMR spectrum of compound **5i** in acetone-D$_6$ + 40 uL D$_2$O.

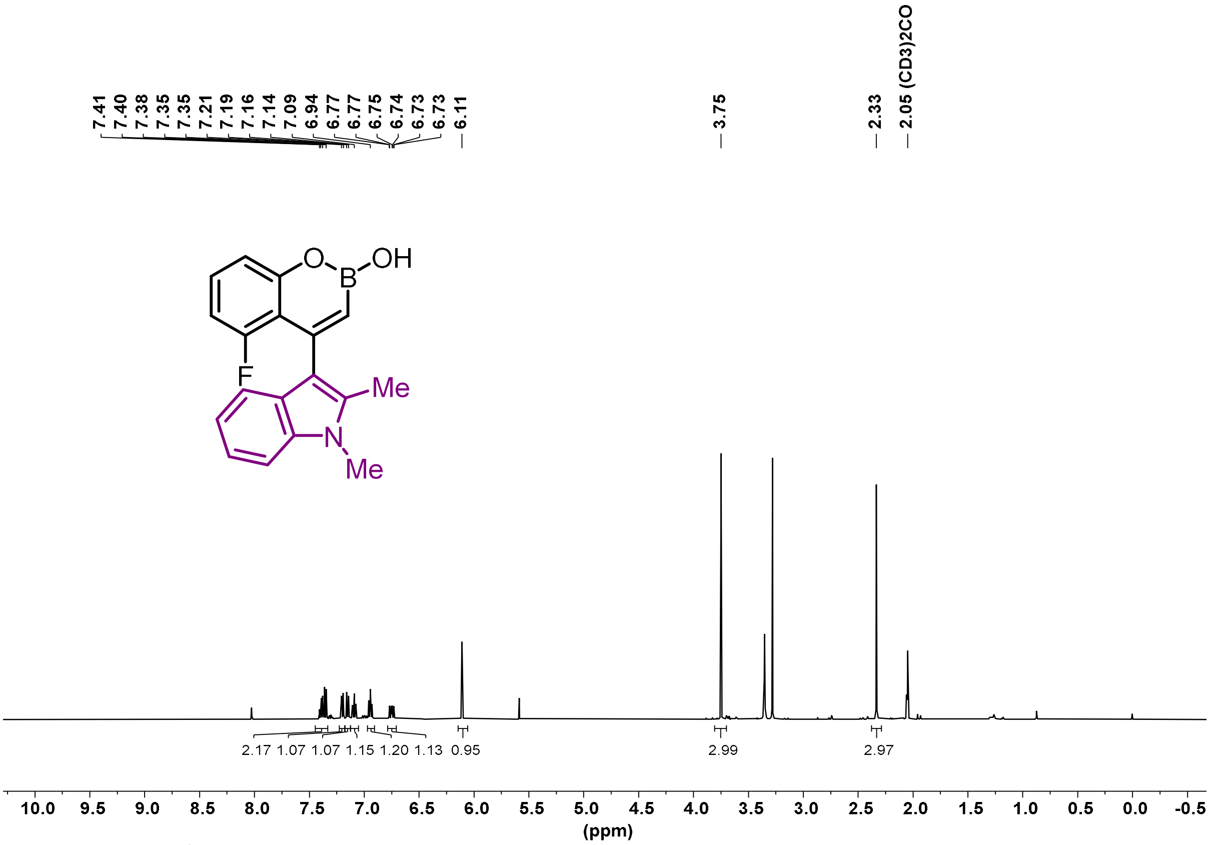

Figure S125. ^{1}H NMR spectrum of compound **5j** in acetone-D_6 + 40 uL D_2O.

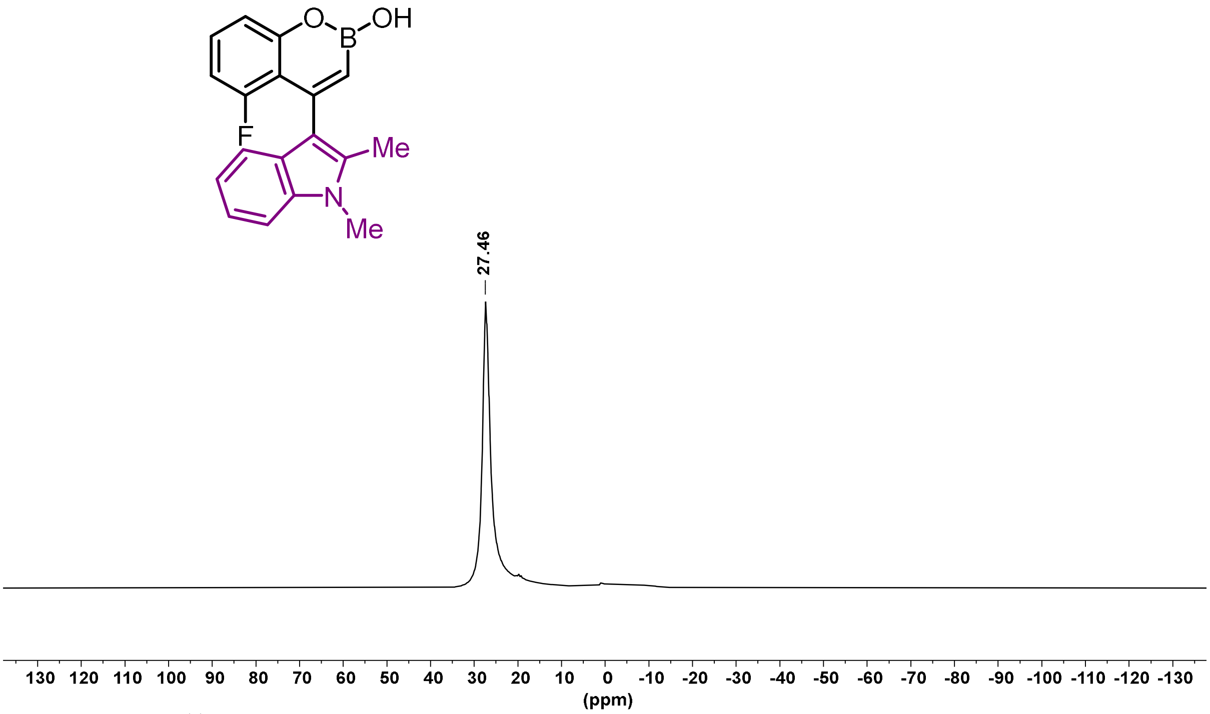

Figure S126. ^{11}B NMR spectrum of compound **5j** in acetone-D_6.

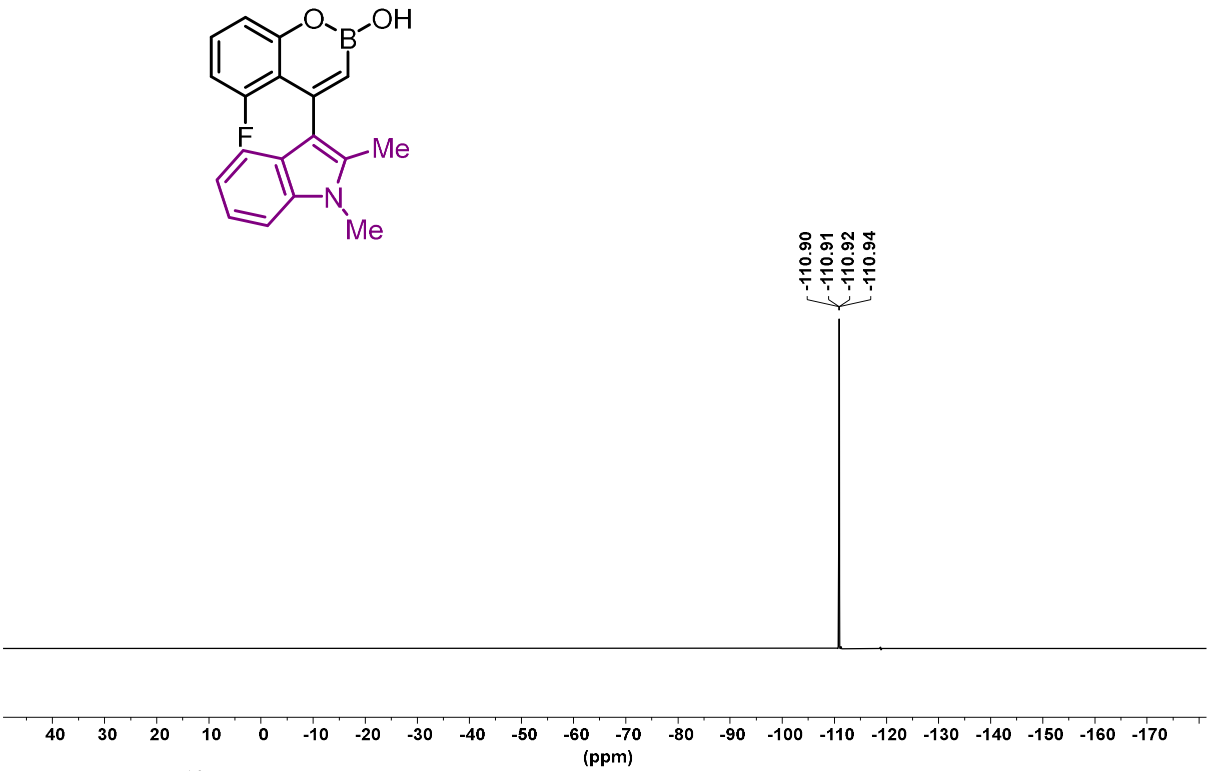

Figure S127. ^{19}F NMR spectrum of compound **5j** in acetone-D6.

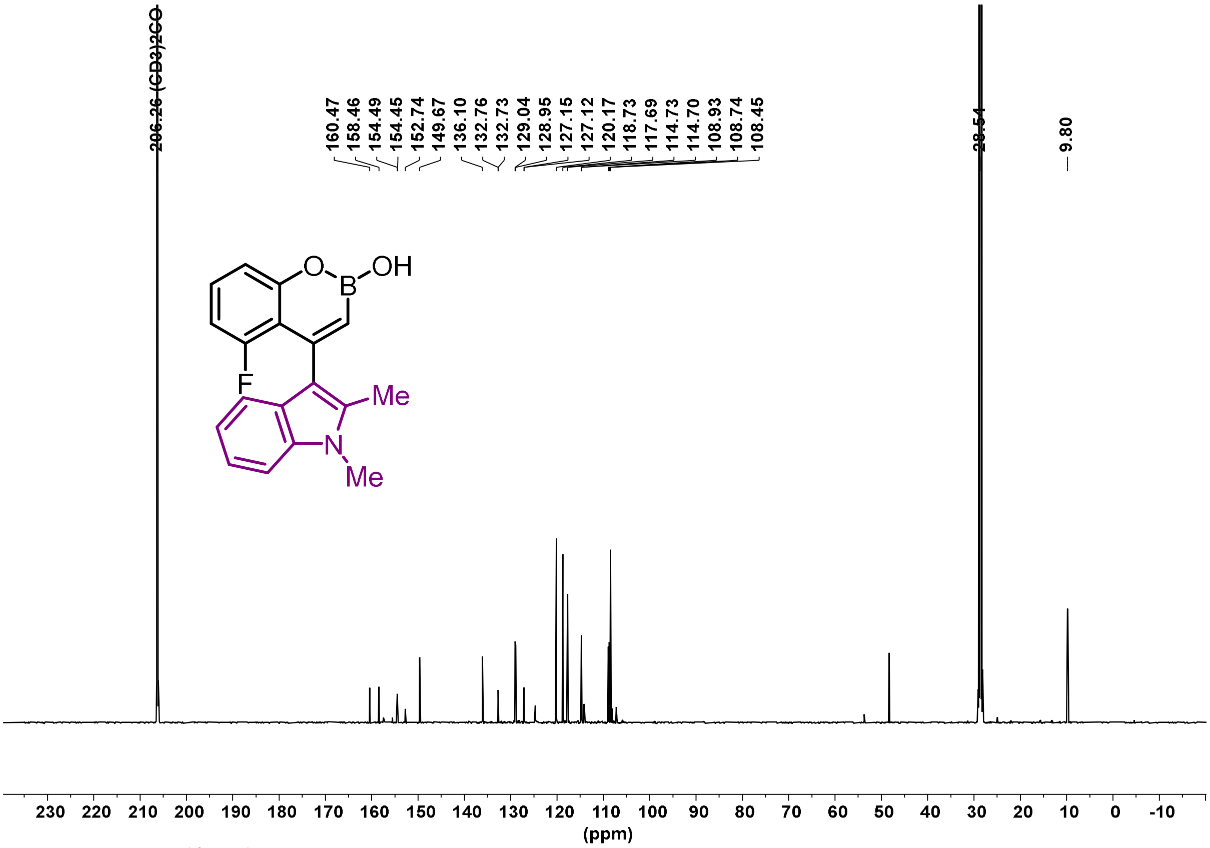

Figure S128. $^{13}C\{^1H\}$ NMR spectrum of compound **5j** in acetone-D6 + 40 uL D_2O.

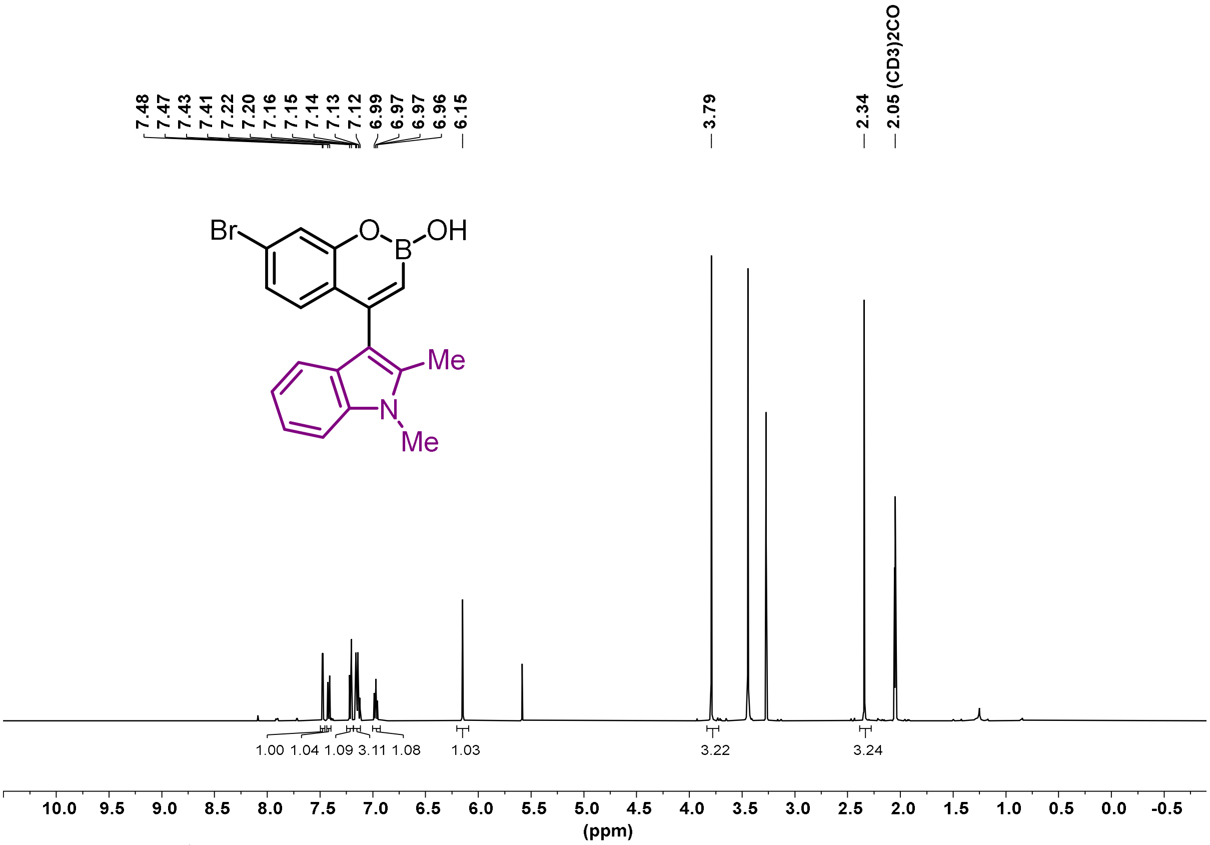

Figure S129. ^{1}H NMR spectrum of compound **5k** in acetone-D_6 + 40 uL D_2O.

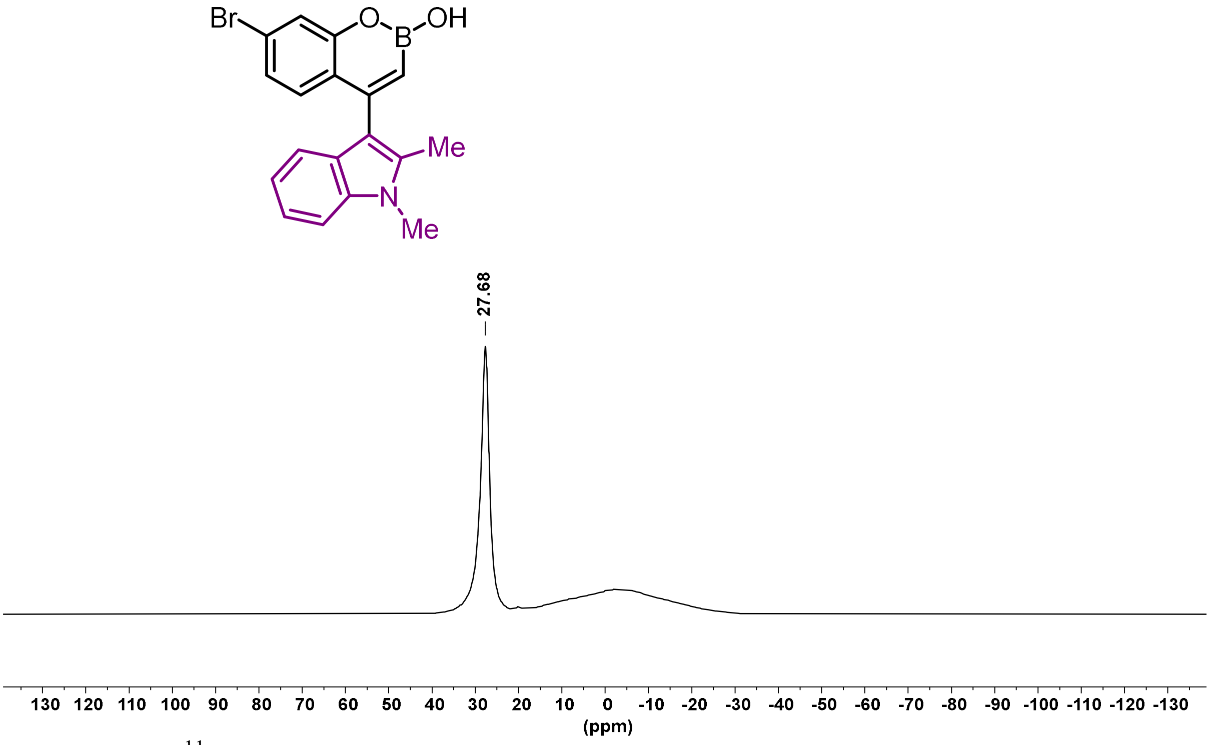

Figure S130. ^{11}B NMR spectrum of compound **5k** in acetone-D_6.

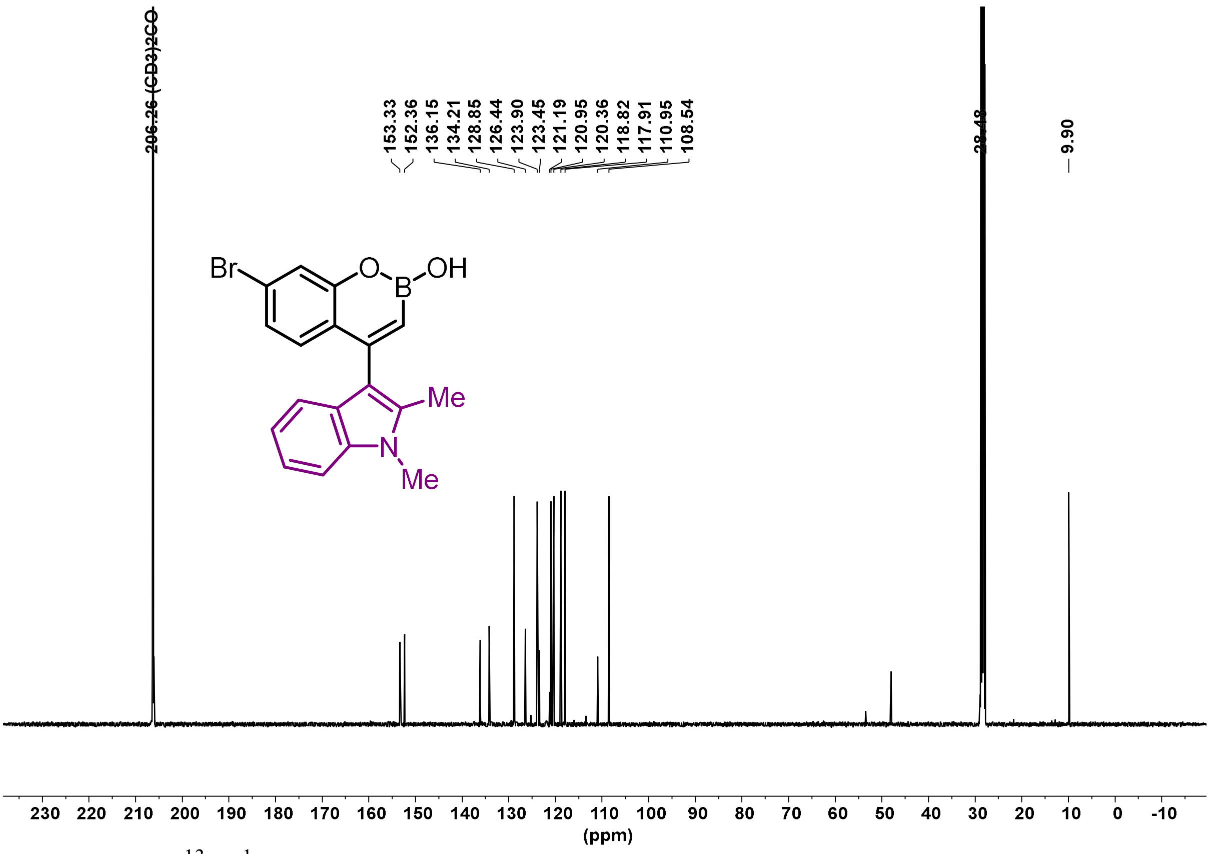

Figure S131. $^{13}C\{^{1}H\}$ NMR spectrum of compound **5k** in acetone-D_6 + 40 uL D_2O.

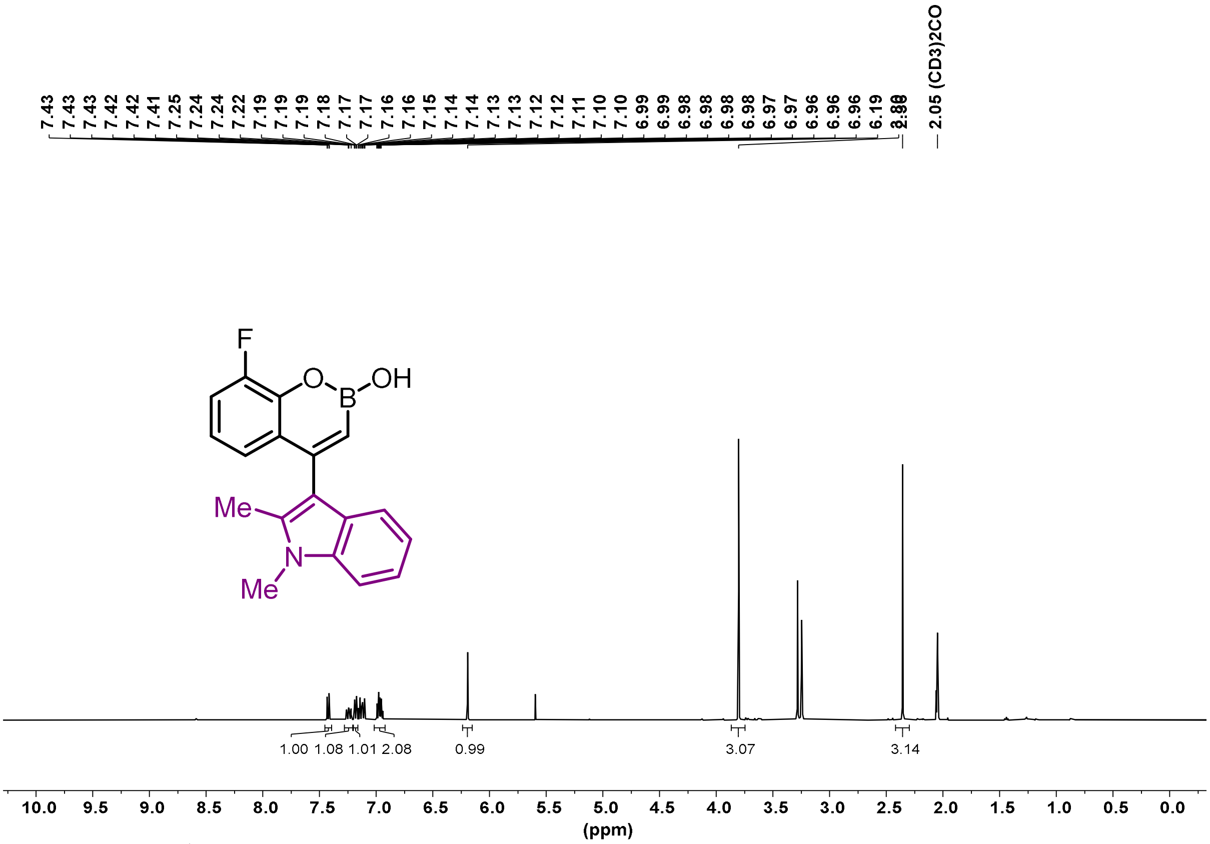

Figure S132. ^{1}H NMR spectrum of compound **5l** in acetone-D_6 + 40 uL D_2O.

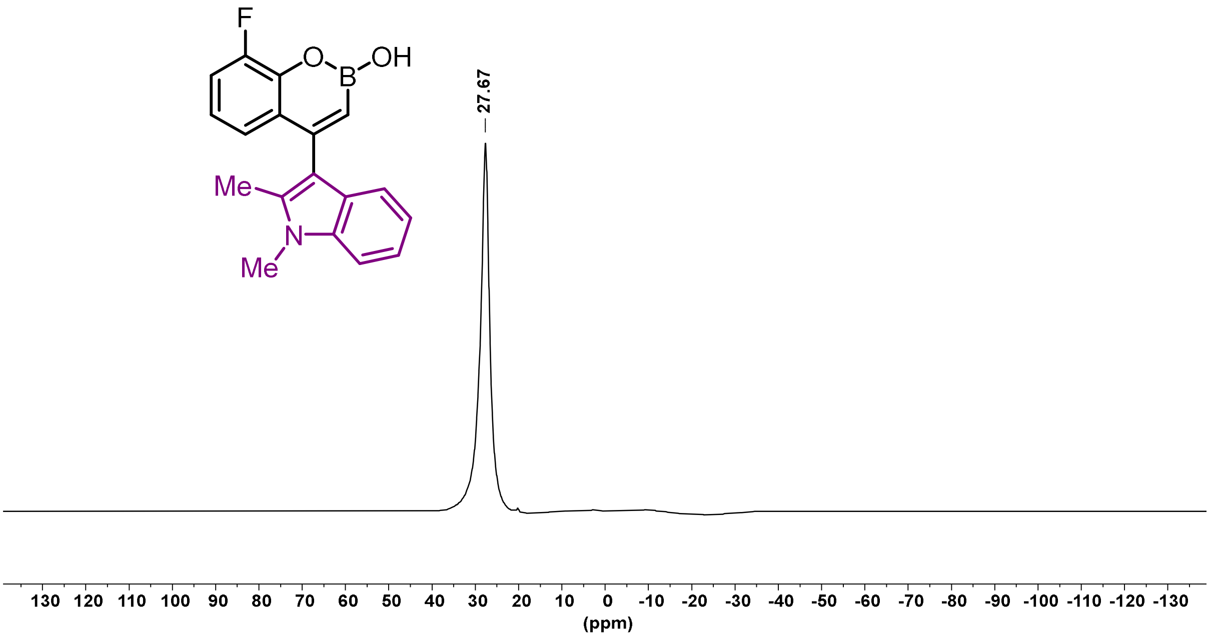

Figure S133. ^{11}B NMR spectrum of compound **5l** in acetone-D$_6$.

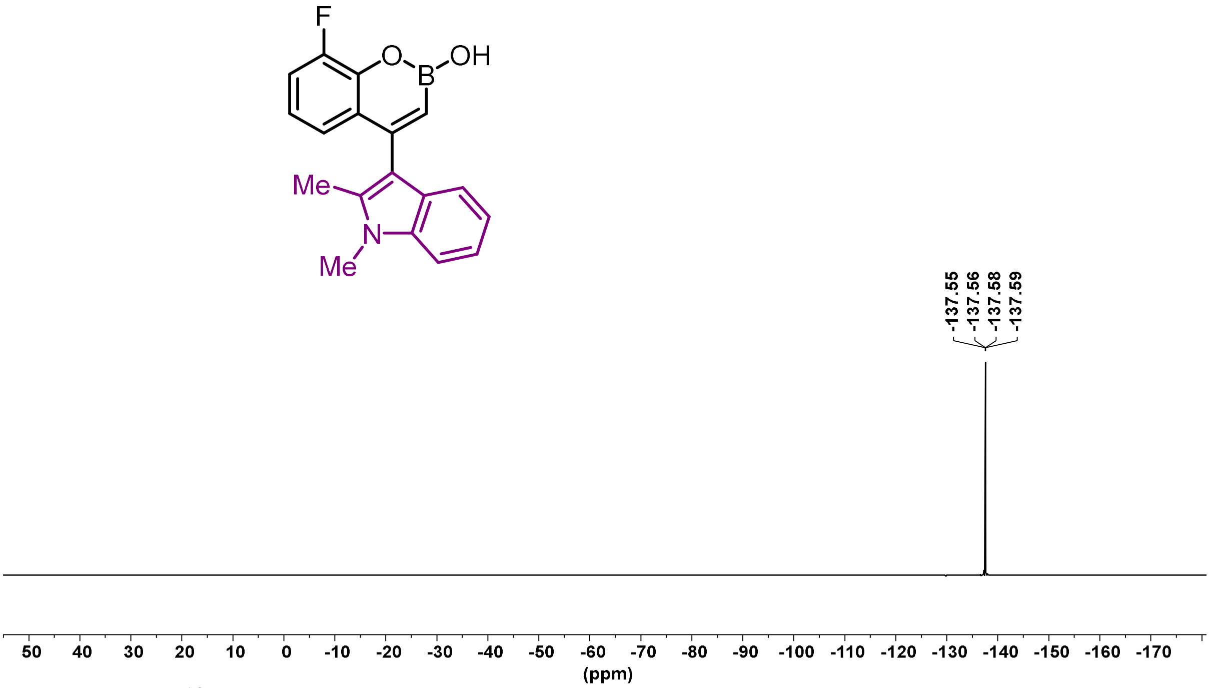

Figure S134. ^{19}F NMR spectrum of compound **5l** in acetone-D$_6$.

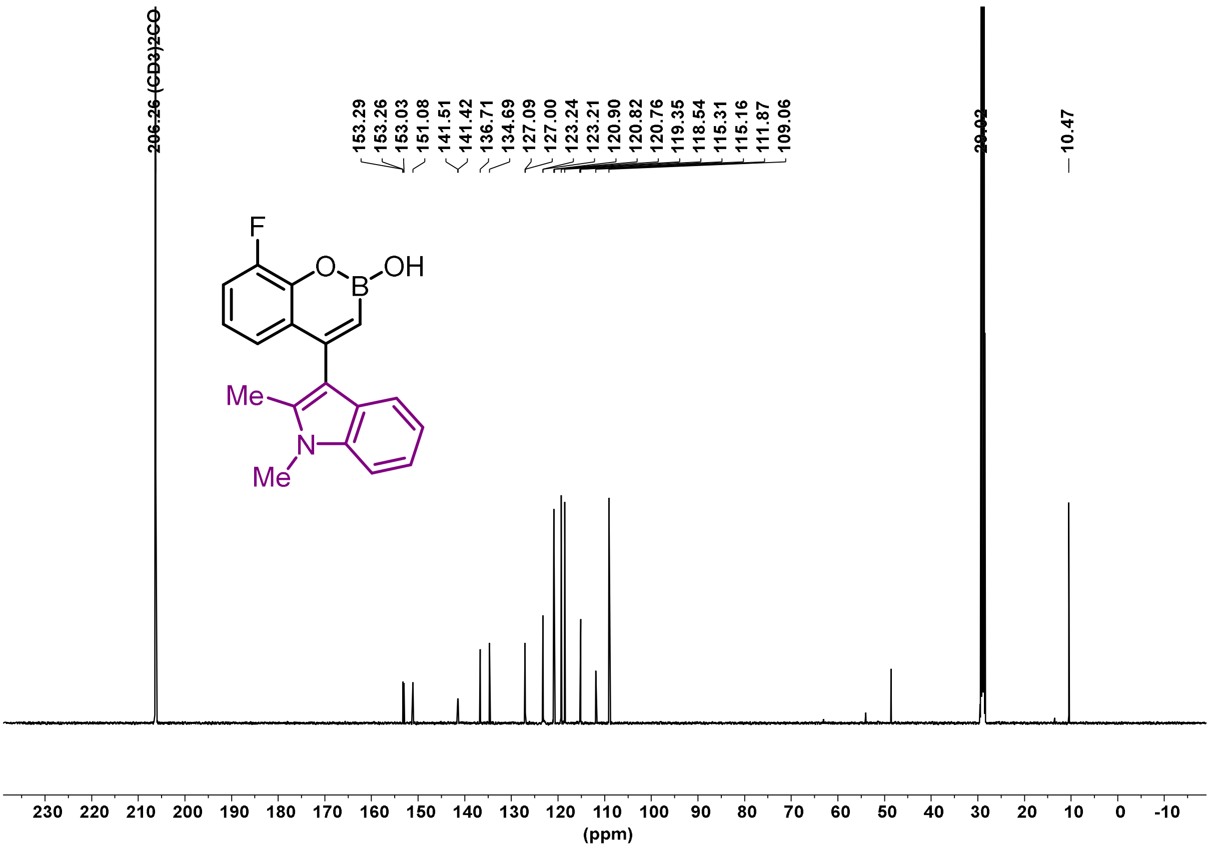

Figure S135. $^{13}C\{^{1}H\}$ NMR spectrum of compound **5l** in acetone-D_6 + 40 uL D_2O.

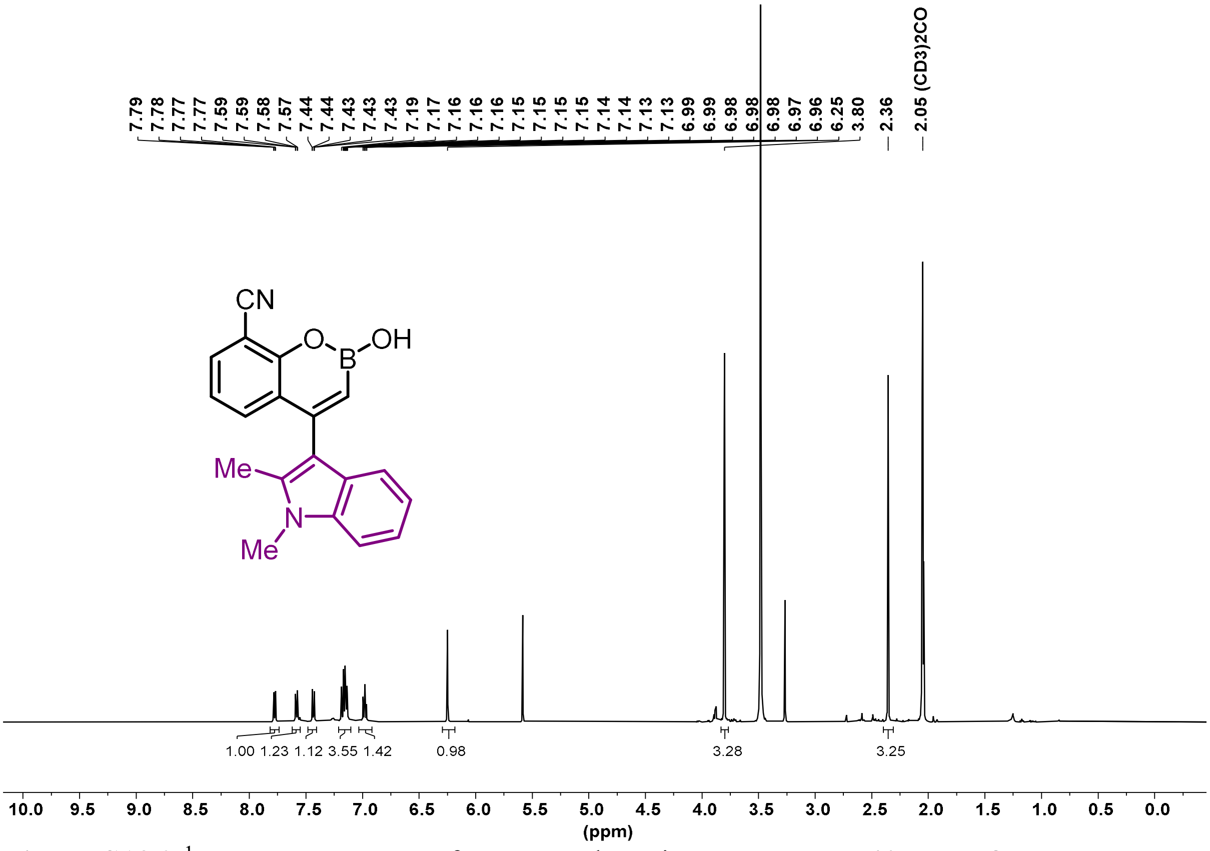

Figure S136. ^{1}H NMR spectrum of compound **5m** in acetone-D_6 + 40 uL D_2O.

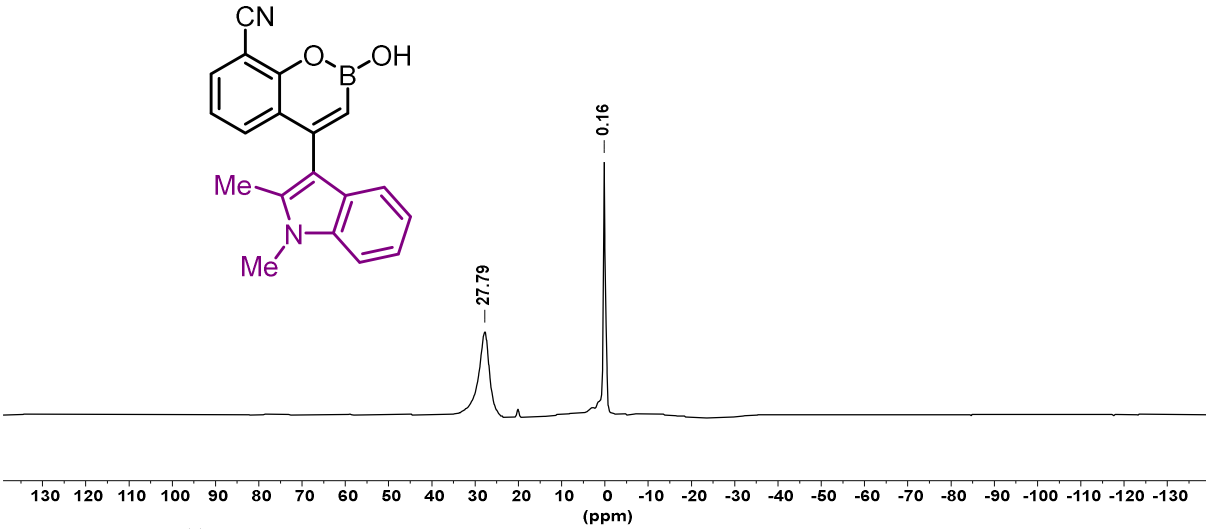

Figure S137. ^{11}B NMR spectrum of compound **5m** in acetone-D_6.
Note the second ^{11}B resonance is due to $[BF_4]$ present, this is a minor component (based on relative integrals)

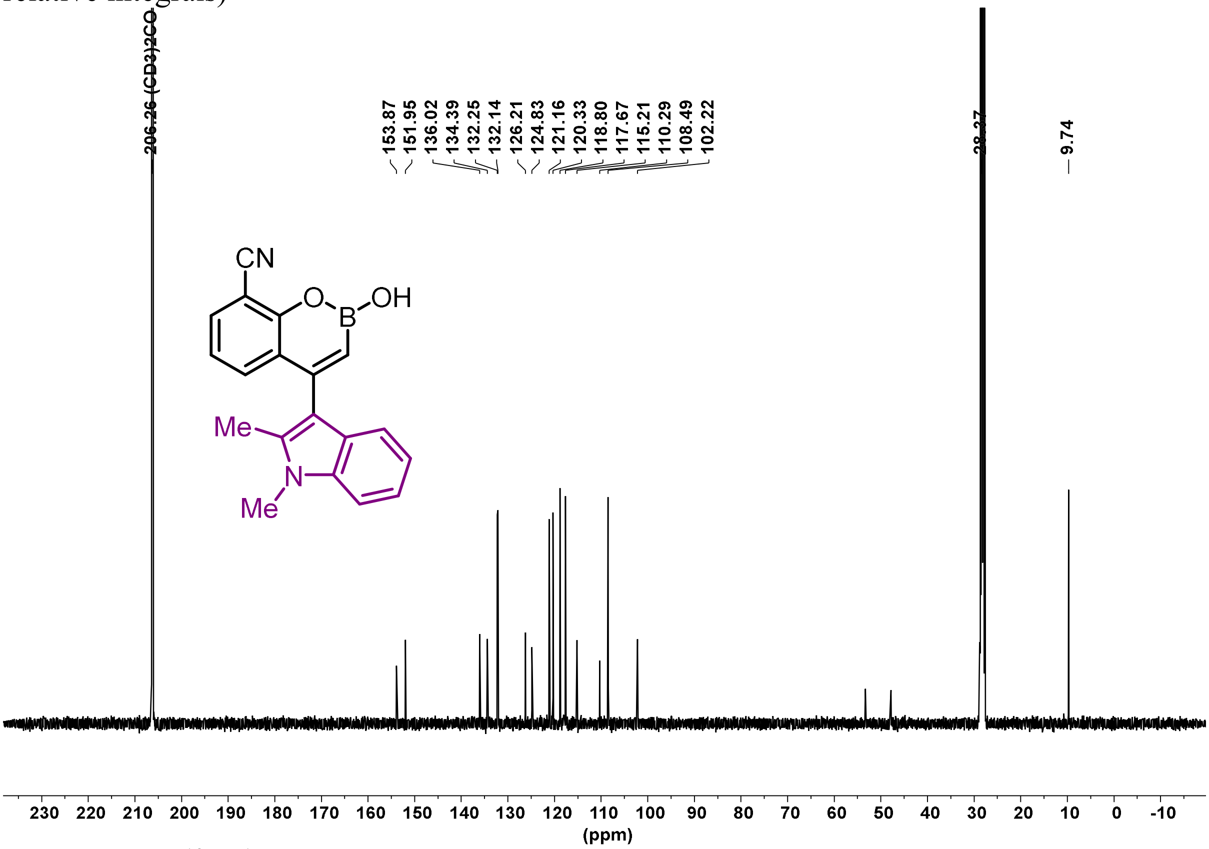

Figure S138. $^{13}C\{^{1}H\}$ NMR spectrum of compound **5m** in acetone-D_6 + 40 uL D_2O.

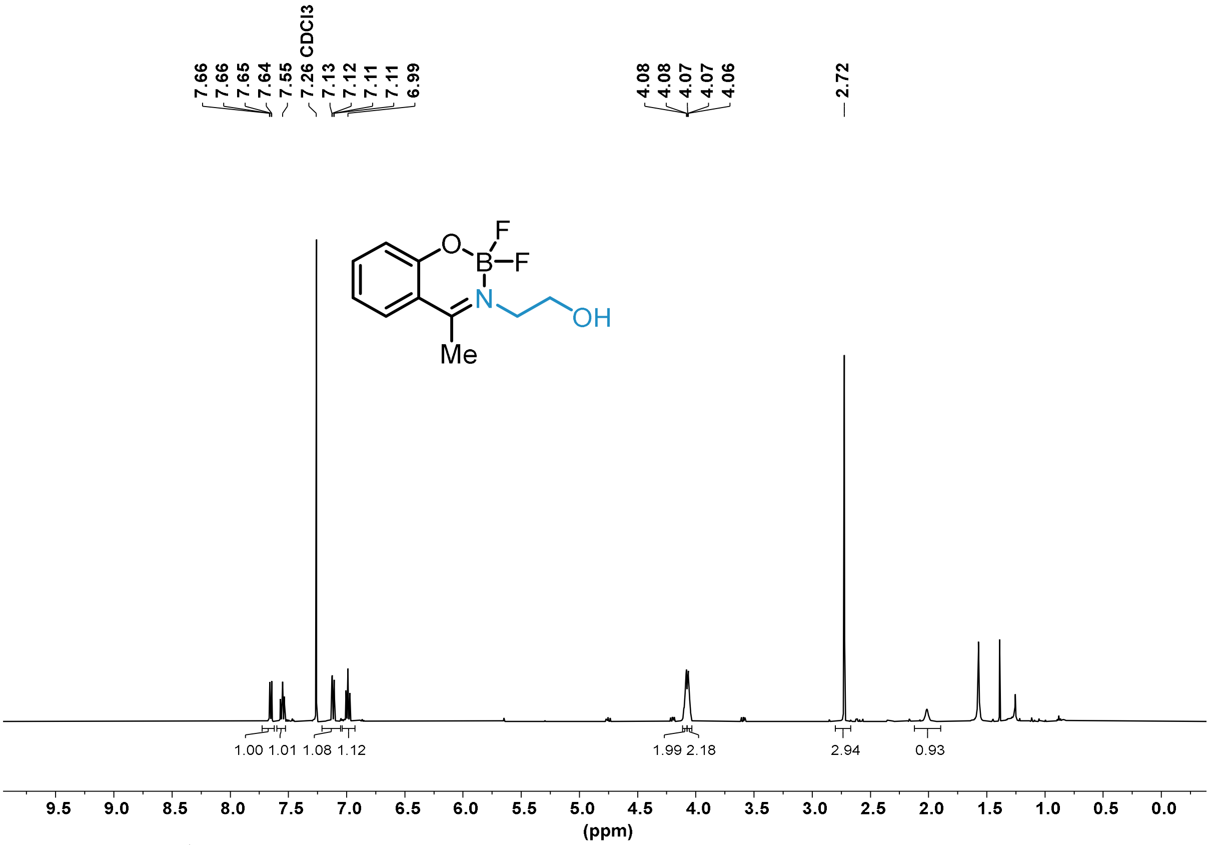

Figure S139. ^{1}H NMR spectrum of compound **6a** in $CDCl_3$.

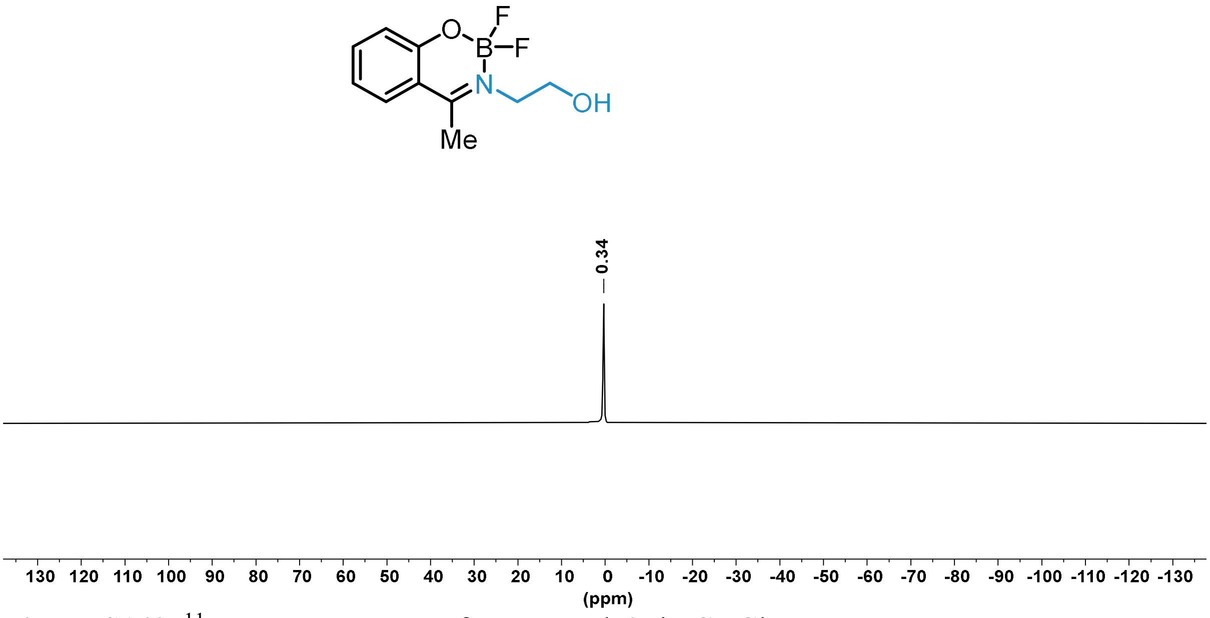

Figure S140. ^{11}B NMR spectrum of compound **6a** in $CDCl_3$.

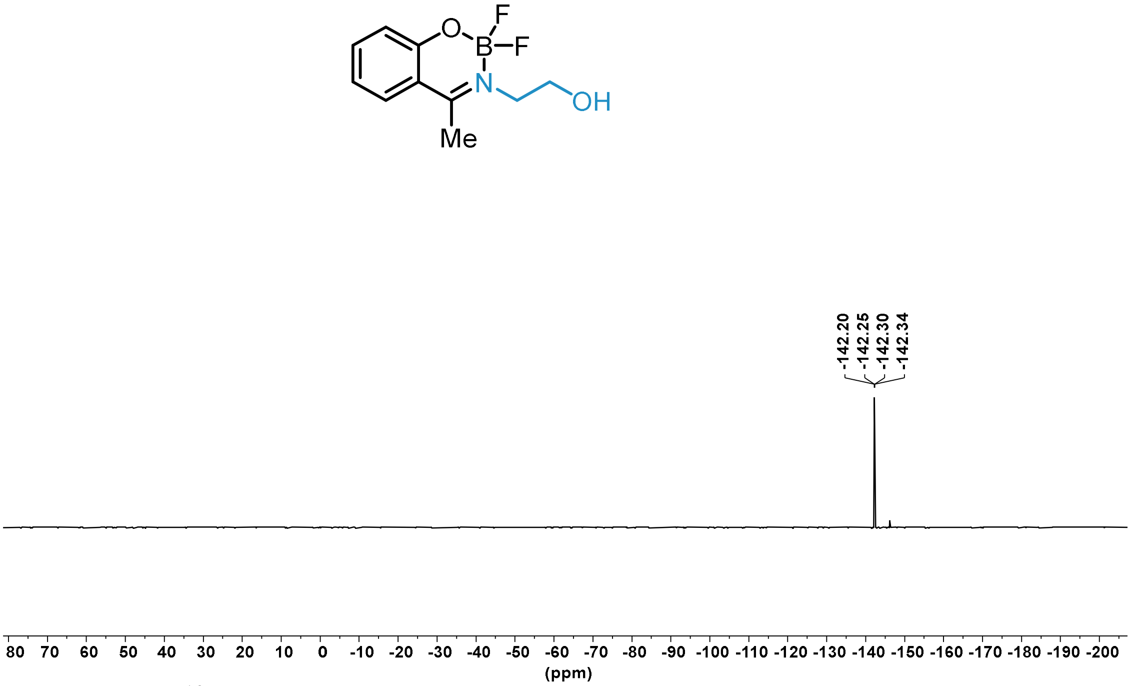

Figure S141. ^{19}F NMR spectrum of compound **6a** in $CDCl_3$.

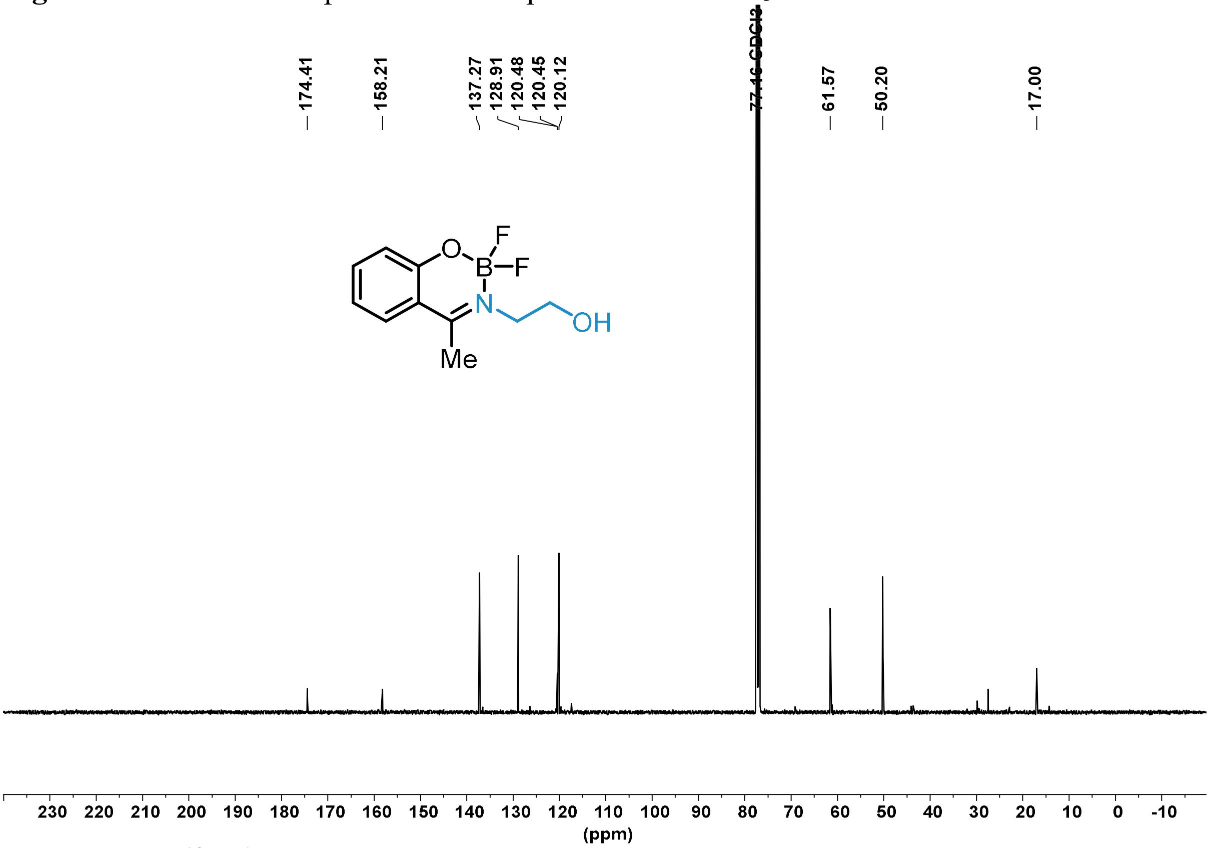

Figure S142. ^{13}C{^{1}H} NMR spectrum of compound **6b** in $CDCl_3$.

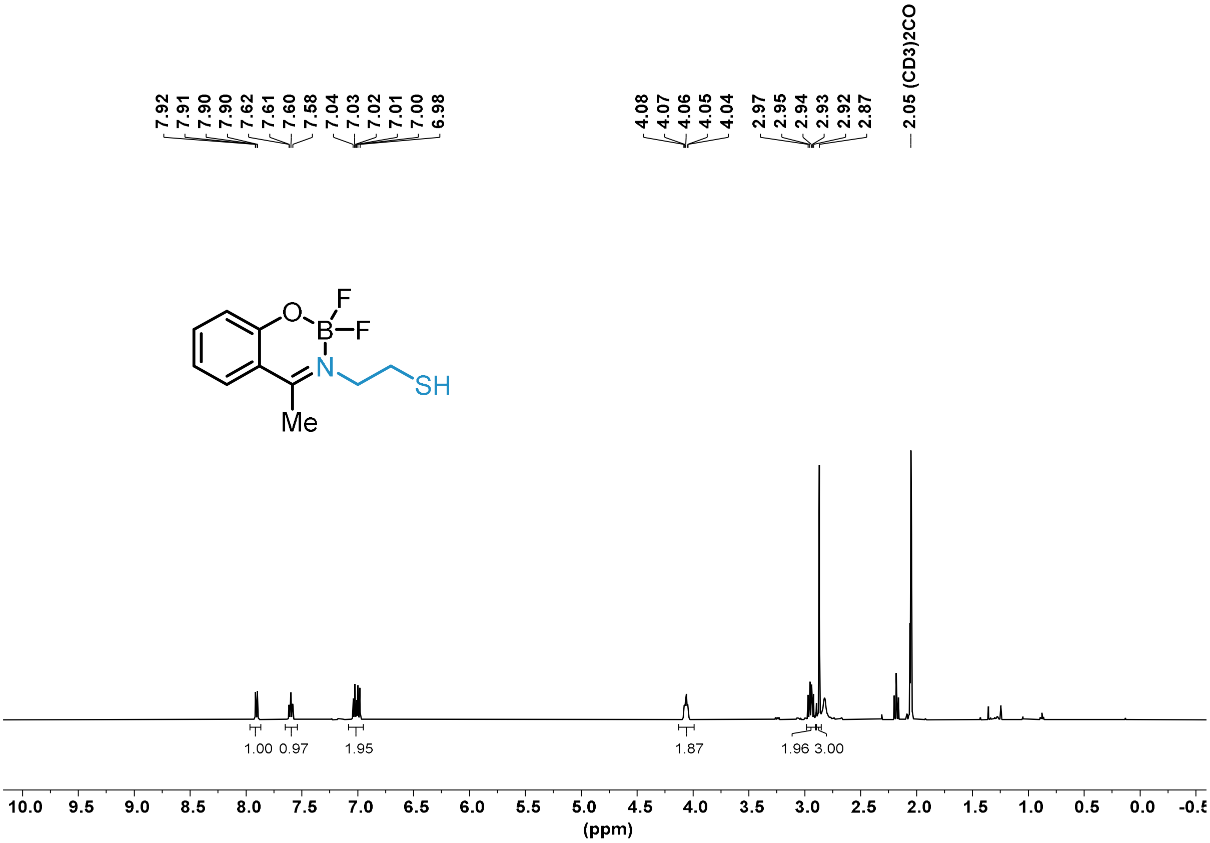

Figure S143. ^{1}H NMR spectrum of compound **6b** in acetone-D_6.

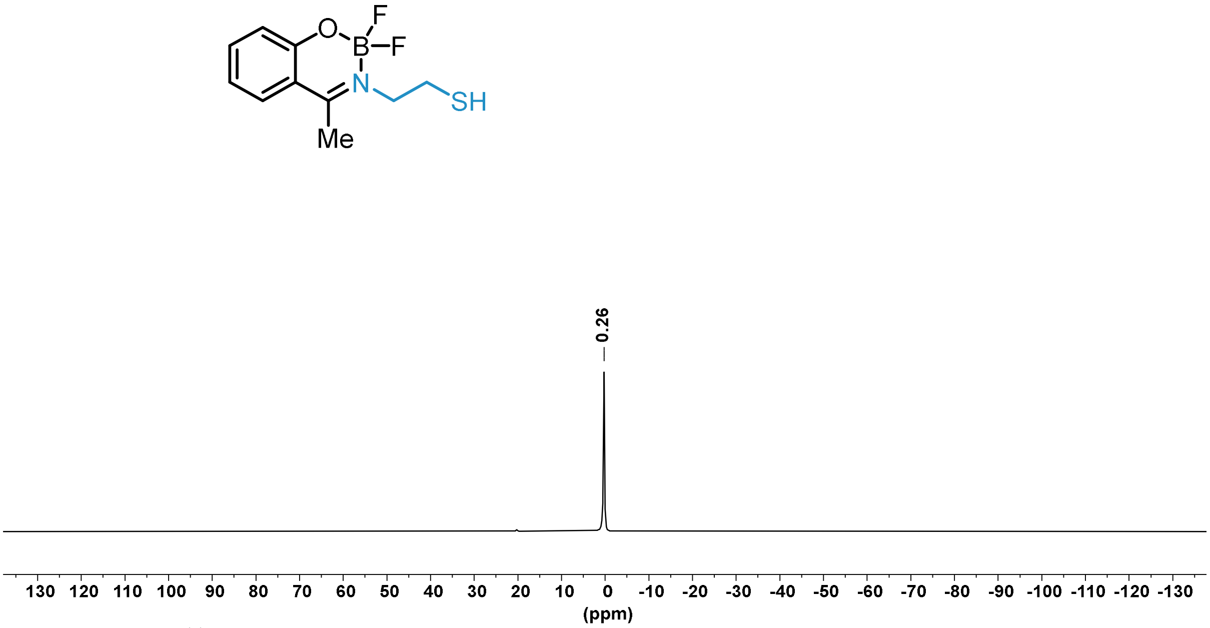

Figure S144. ^{11}B NMR spectrum of compound **6b** in acetone-D_6.

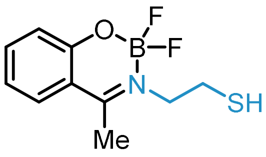

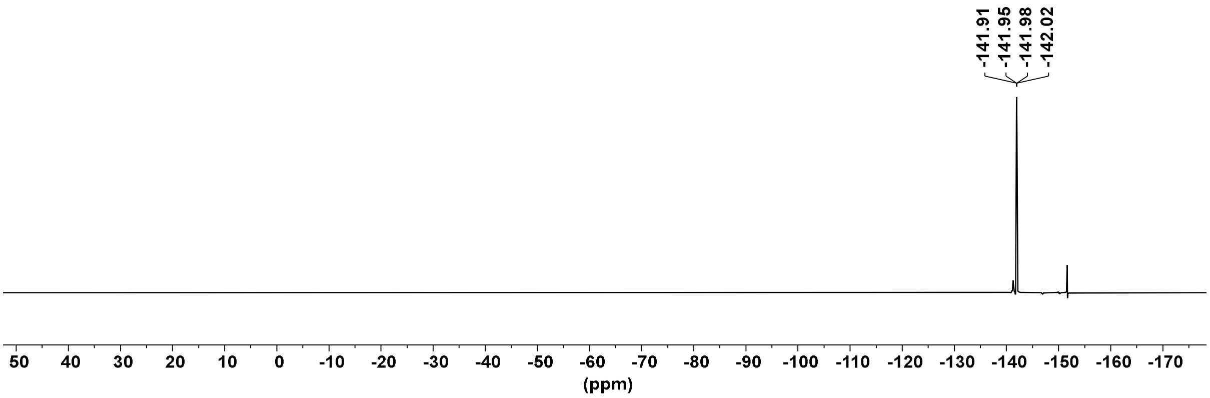

Figure S145. ^{19}F NMR spectrum of compound **6b** in acetone-D6.

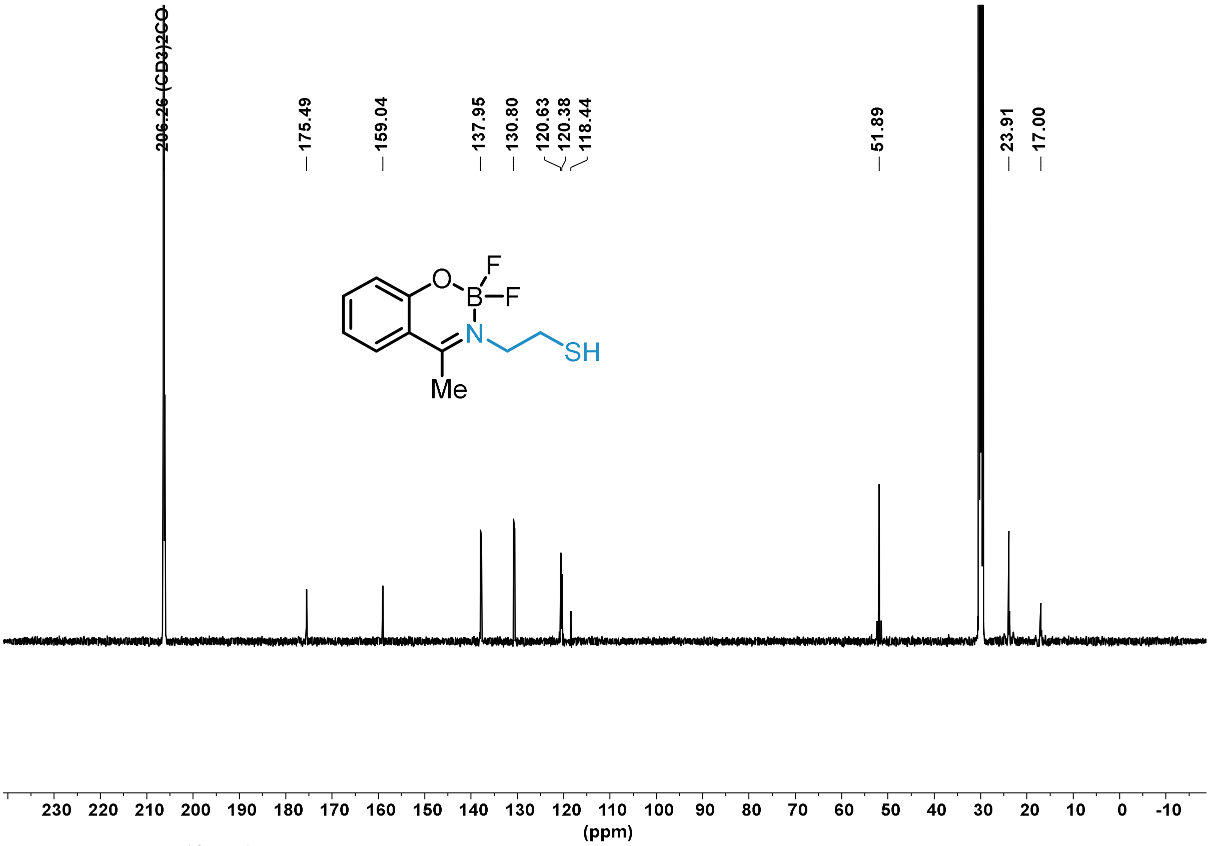

Figure S146. $^{13}C\{^{1}H\}$ NMR spectrum of compound **6b** in acetone-D6.

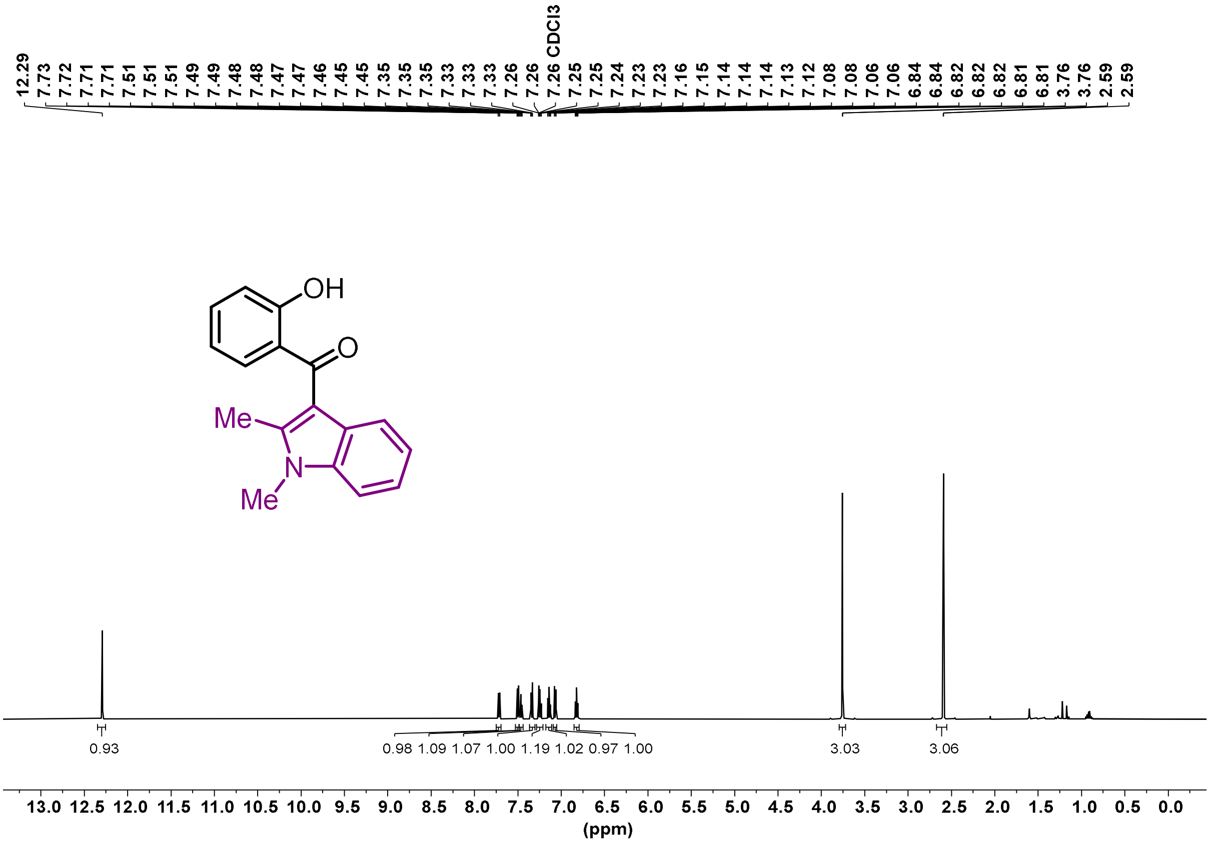

Figure S147. ^{1}H NMR spectrum of compound **7** in $CDCl_3$.

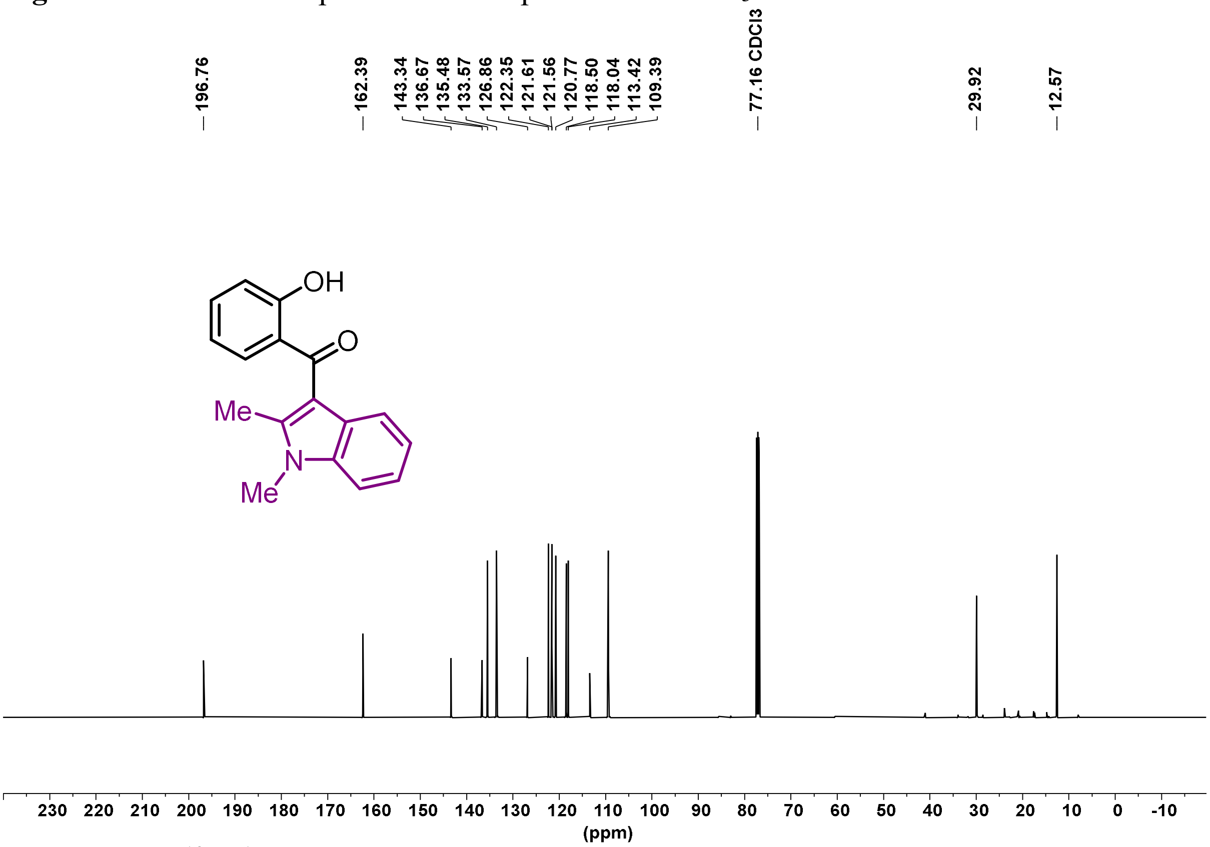

Figure S148. $^{13}C\{^{1}H\}$ NMR spectrum of compound **7** in $CDCl_3$.

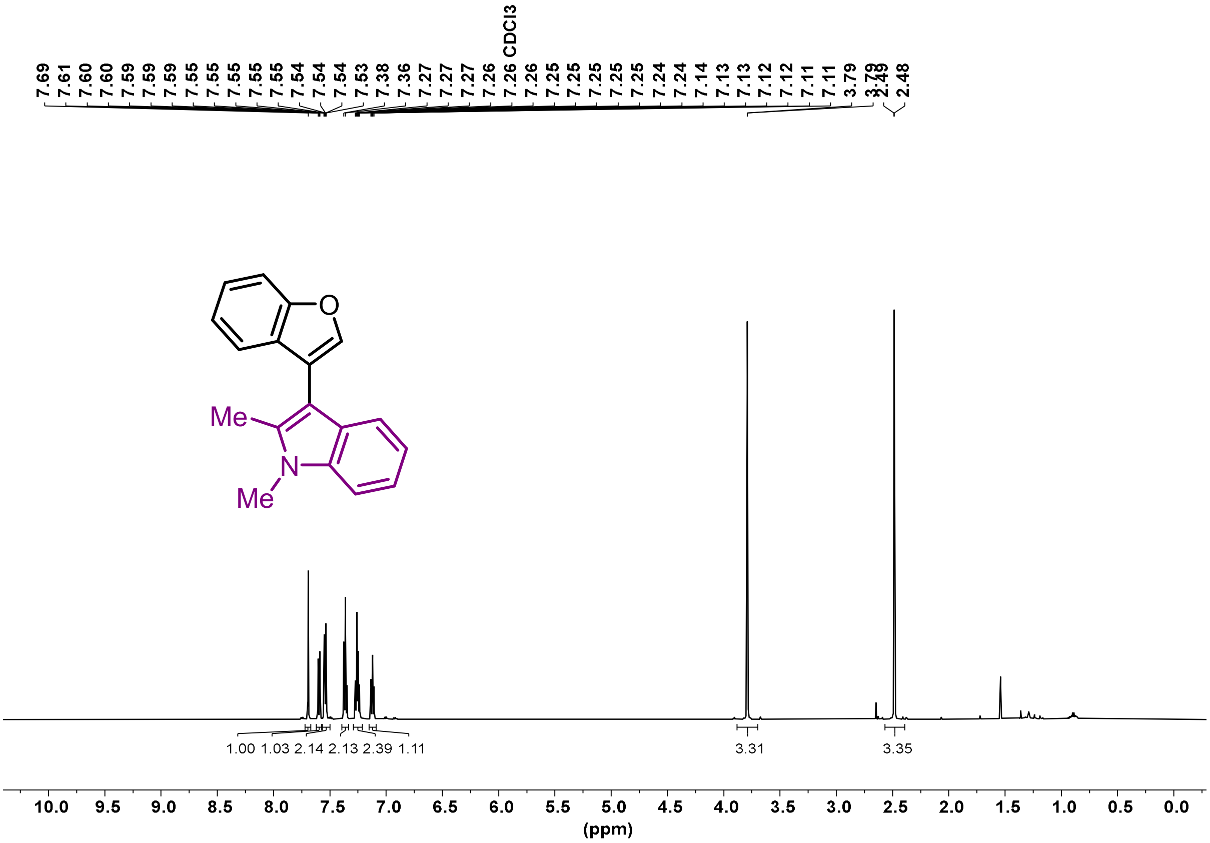

Figure S149. ^{1}H NMR spectrum of compound **8** in $CDCl_3$.

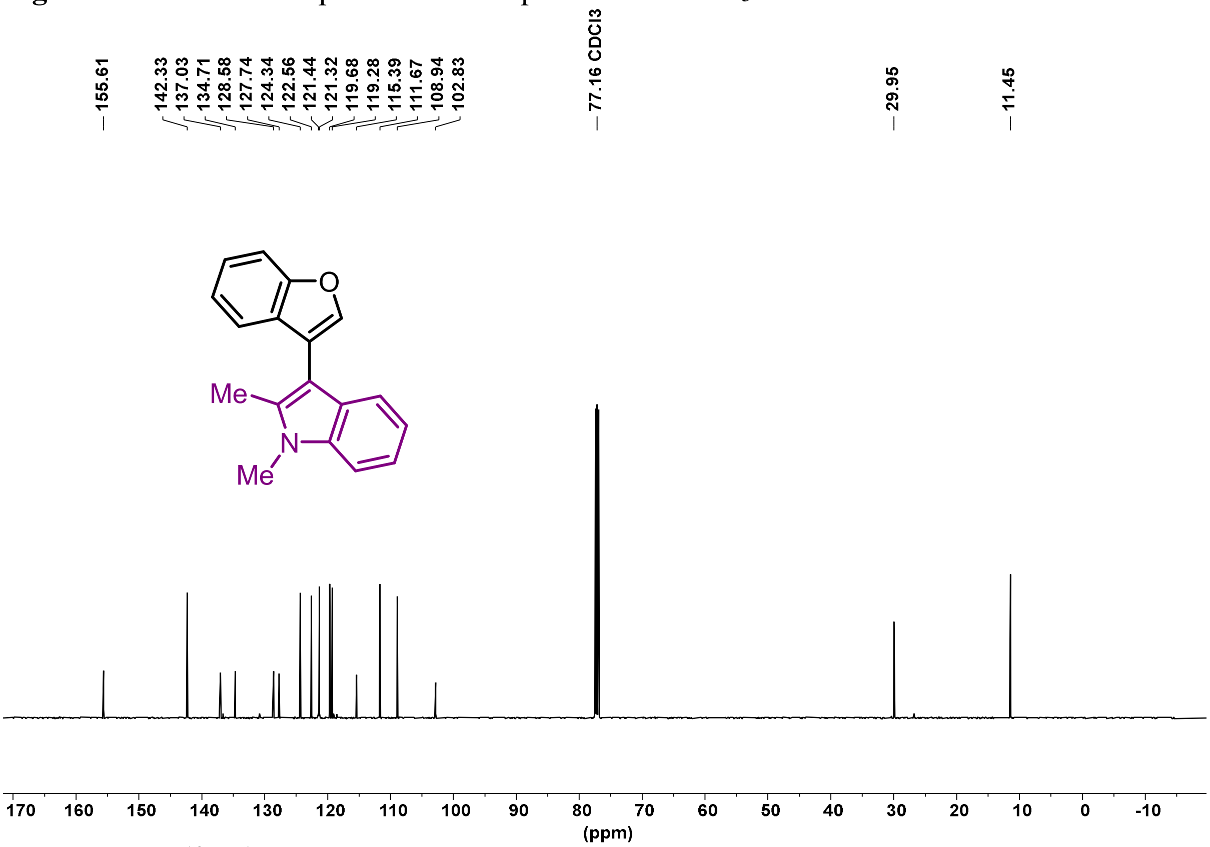

Figure S150. $^{13}C\{^{1}H\}$ NMR spectrum of compound **8** in $CDCl_3$.

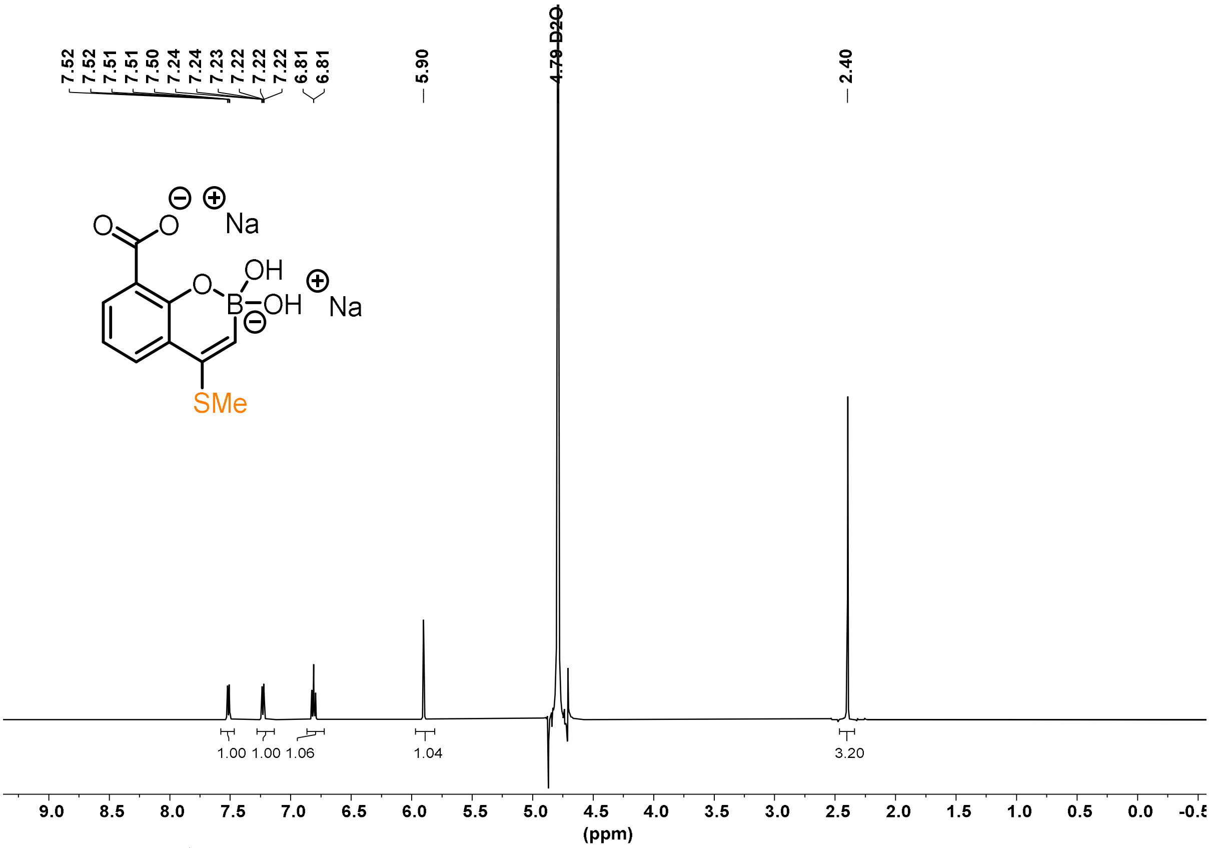

Figure S151. ¹H NMR spectrum of compound **9** in D_2O.

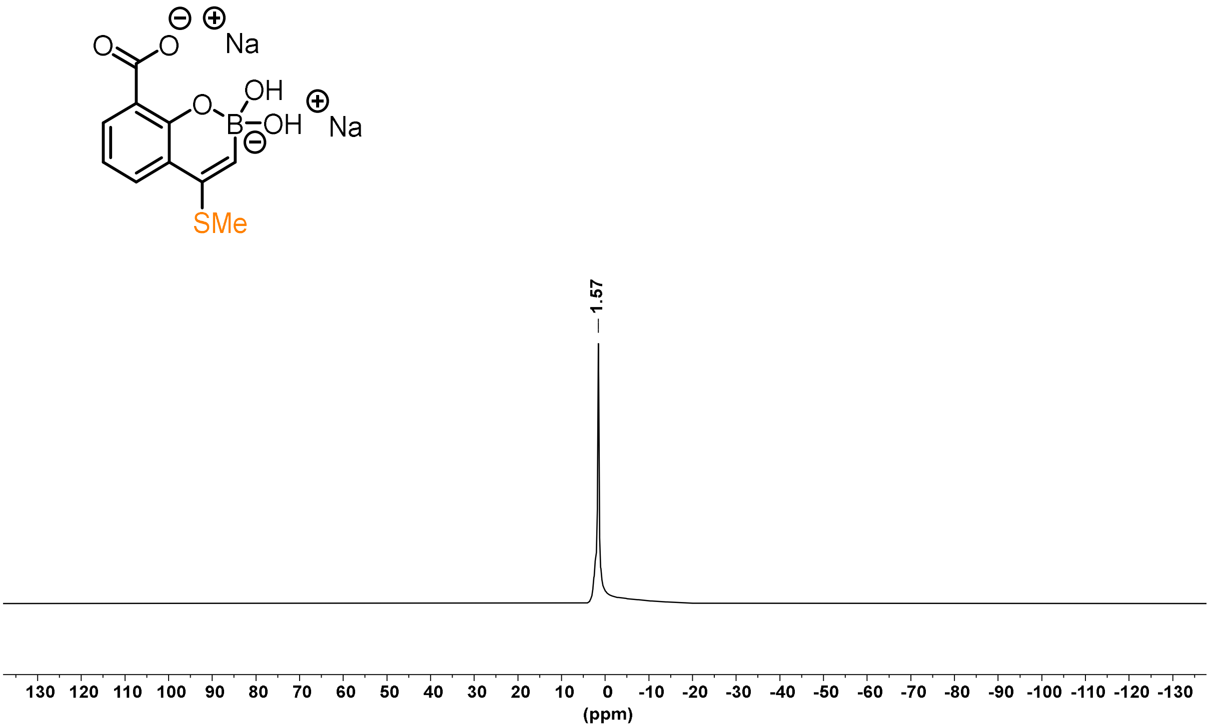

Figure S152. ¹¹B NMR spectrum of compound **9** in D_2O.

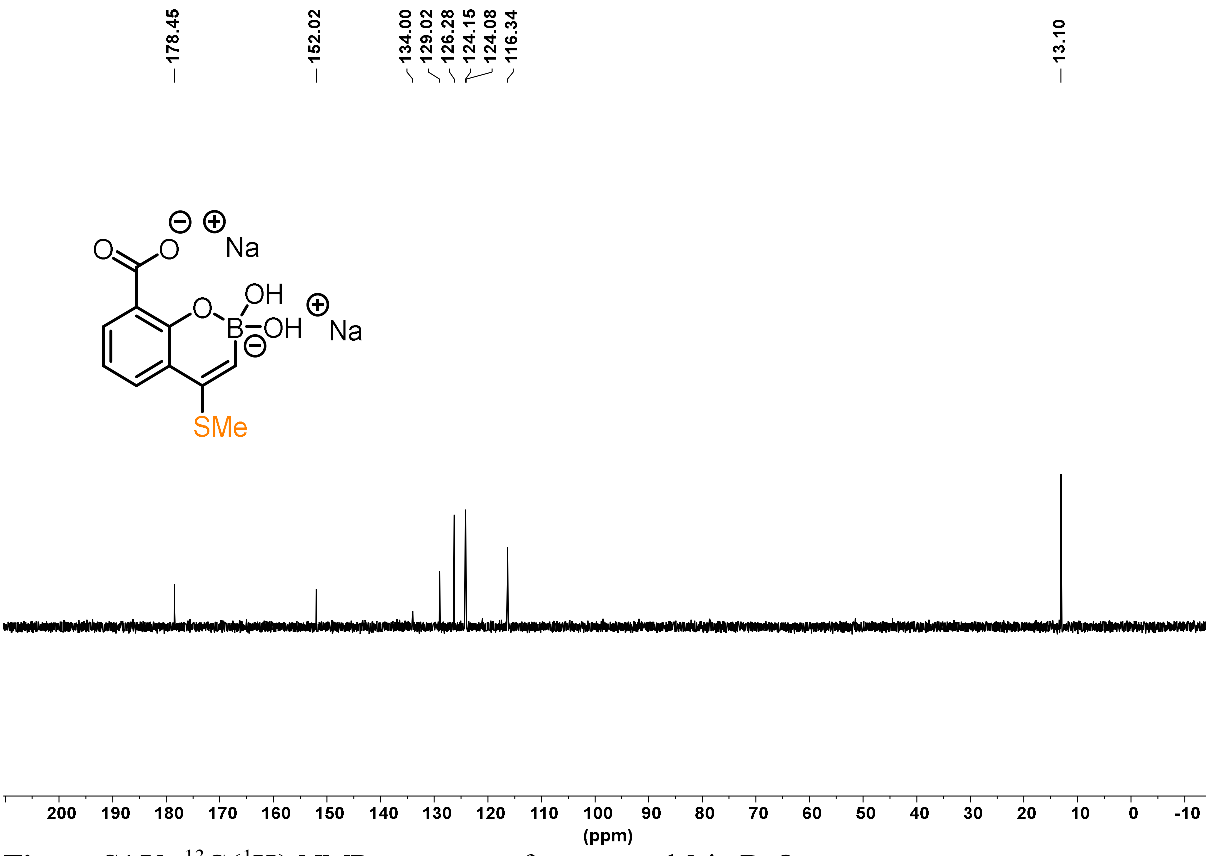

Figure S153. $^{13}C\{^{1}H\}$ NMR spectrum of compound **9** in D_2O.

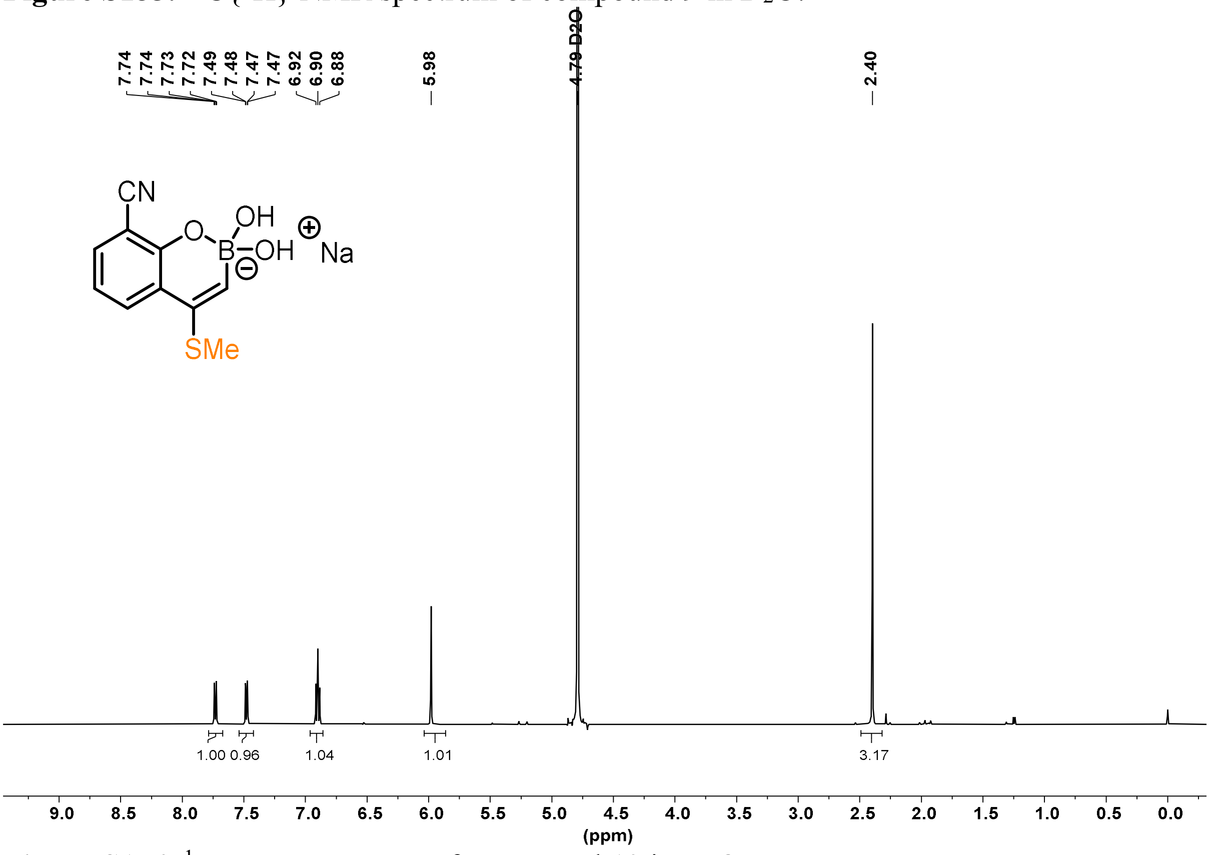

Figure S154. ^{1}H NMR spectrum of compound **10** in D_2O.

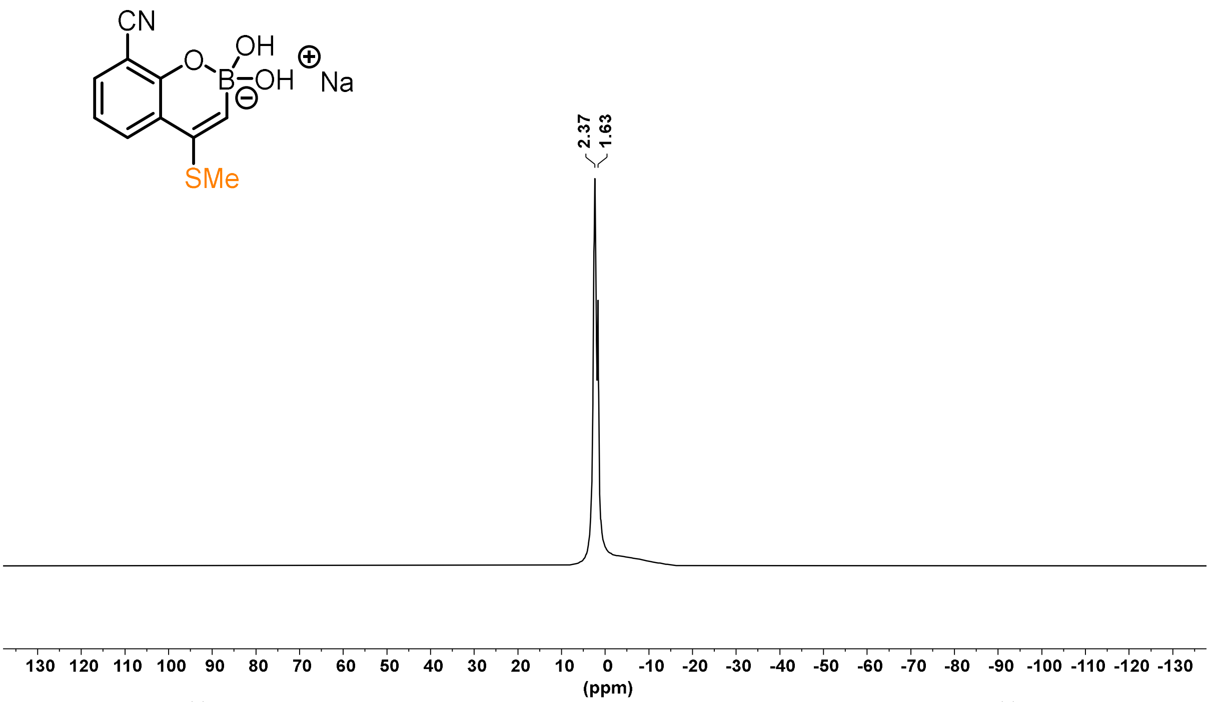

Figure S155. ^{11}B NMR spectrum of compound **10** in D_2O. Note the second ^{11}B resonance is due to trace $Na[B(OH)_4]$.

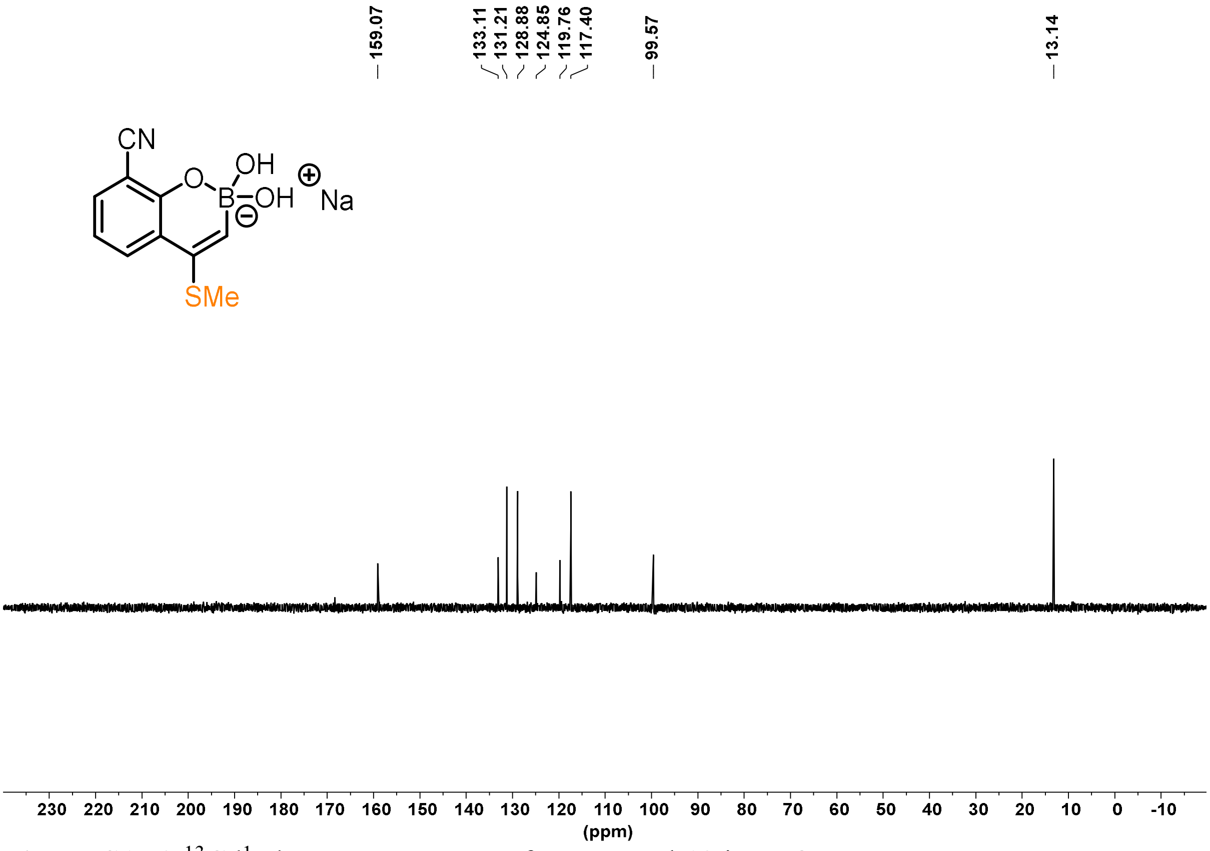

Figure S156. $^{13}C\{^{1}H\}$ NMR spectrum of compound **10** in D_2O.

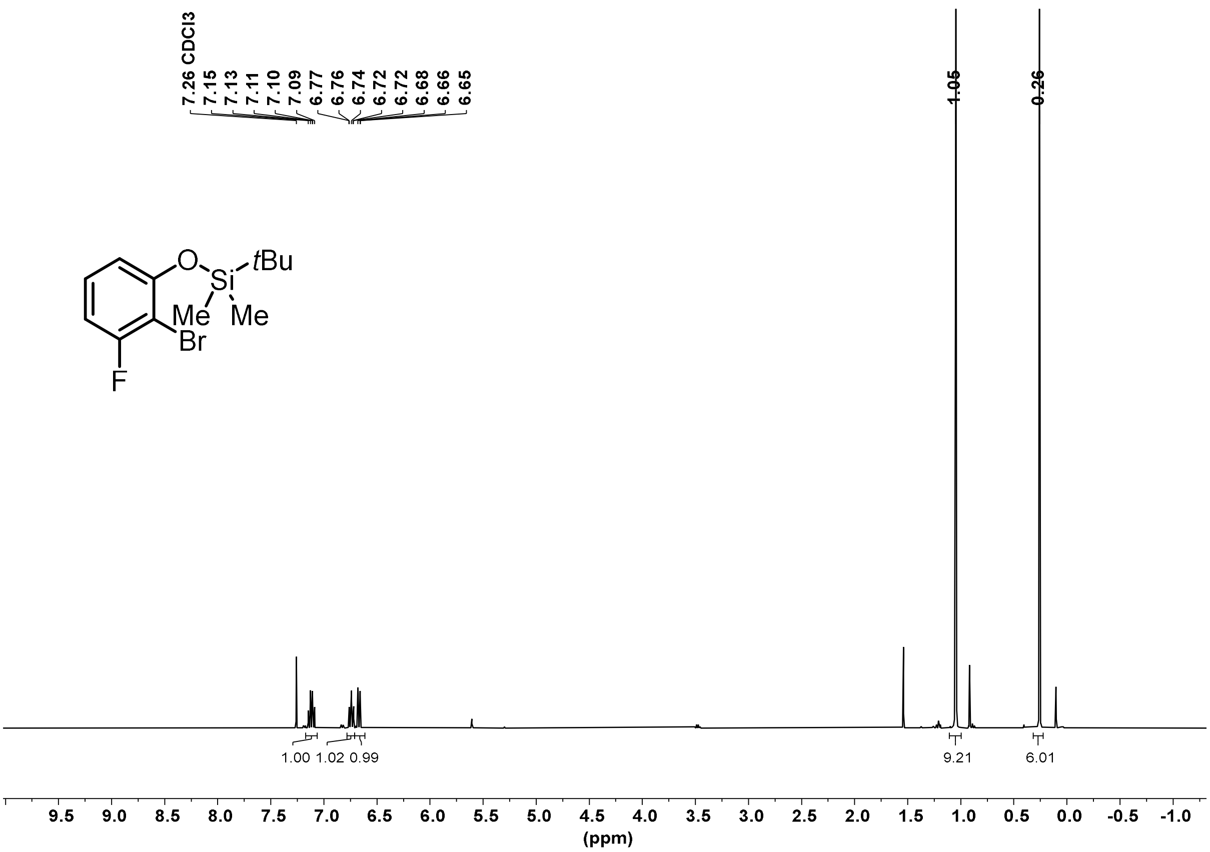

Figure S157. ^{1}H NMR spectrum of compound **14b** in $CDCl_3$.

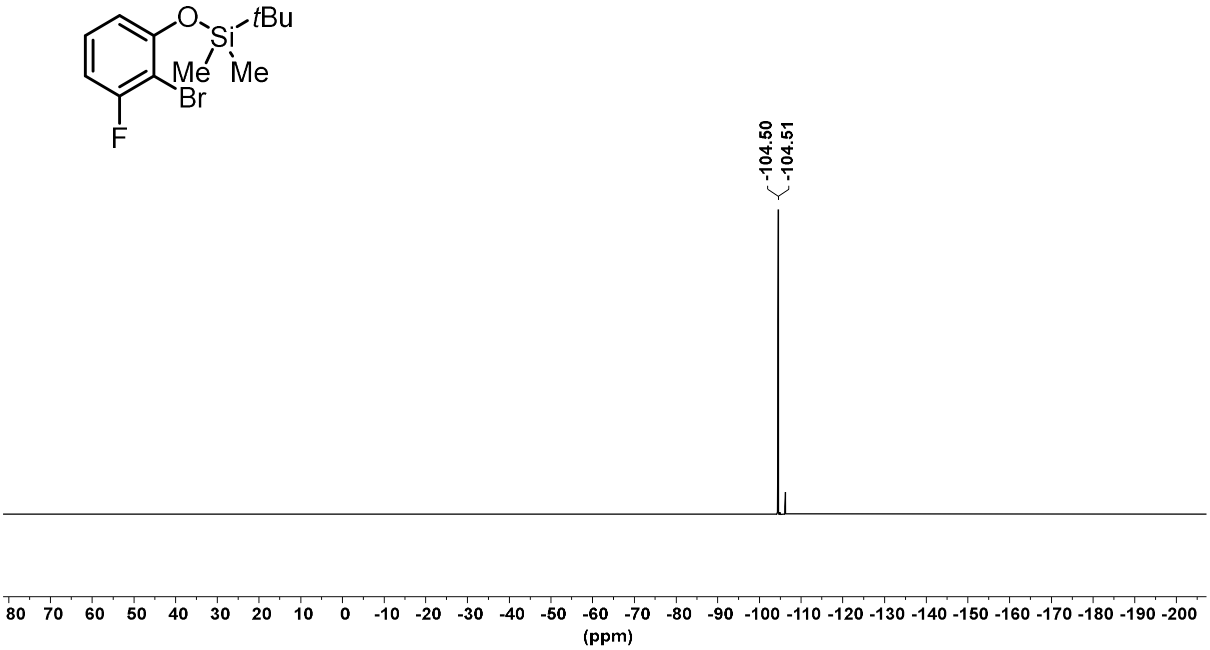

Figure S158. ^{19}F NMR spectrum of compound **14b** in $CDCl_3$.

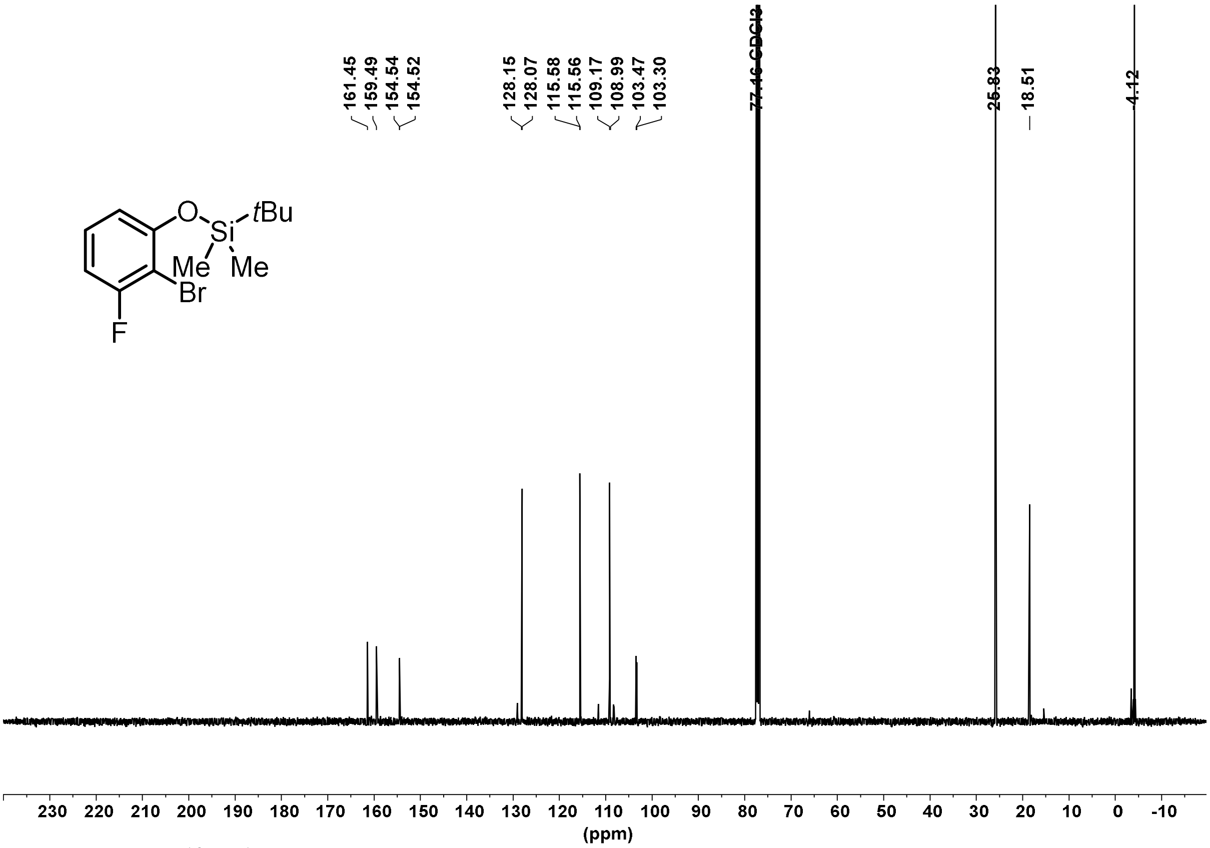

Figure S159. $^{13}C\{^1H\}$ NMR spectrum of compound **14b** in $CDCl_3$.

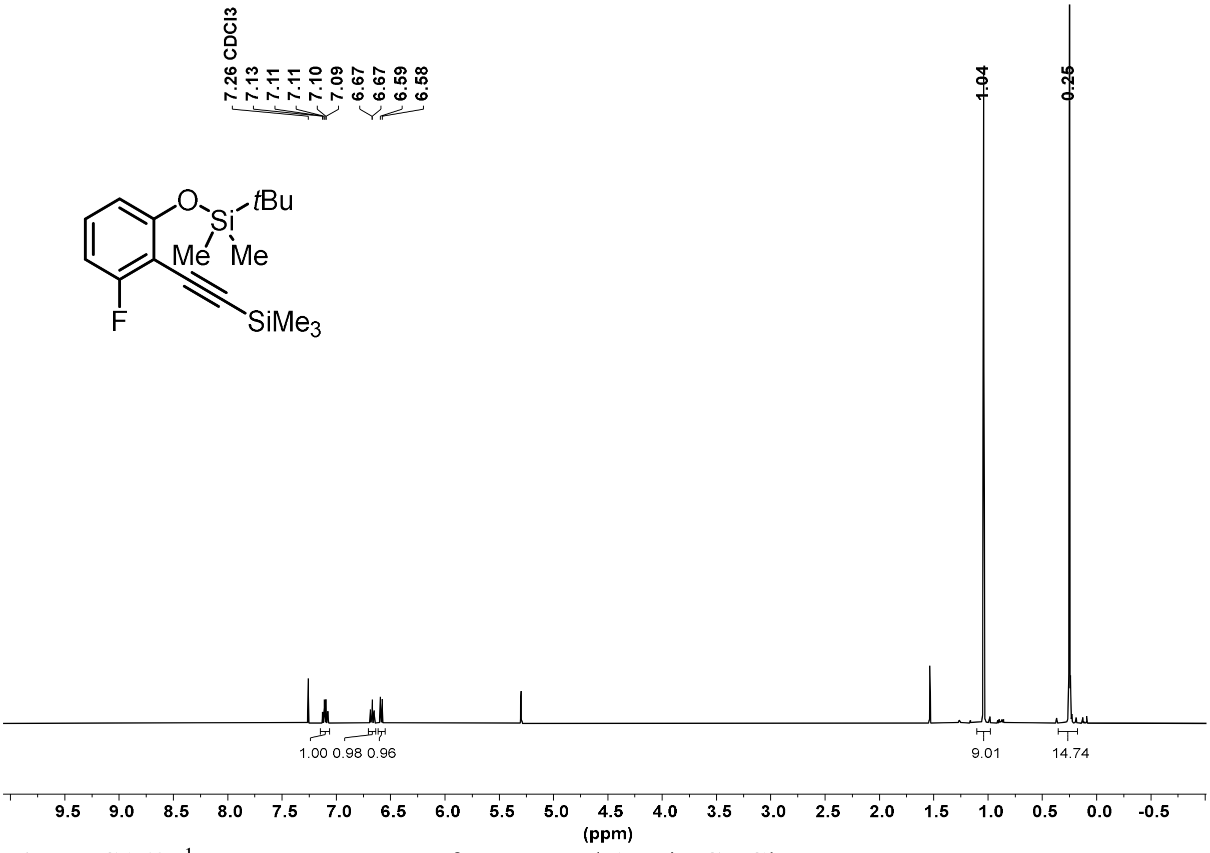

Figure S160. ^1H NMR spectrum of compound **15b** in $CDCl_3$.

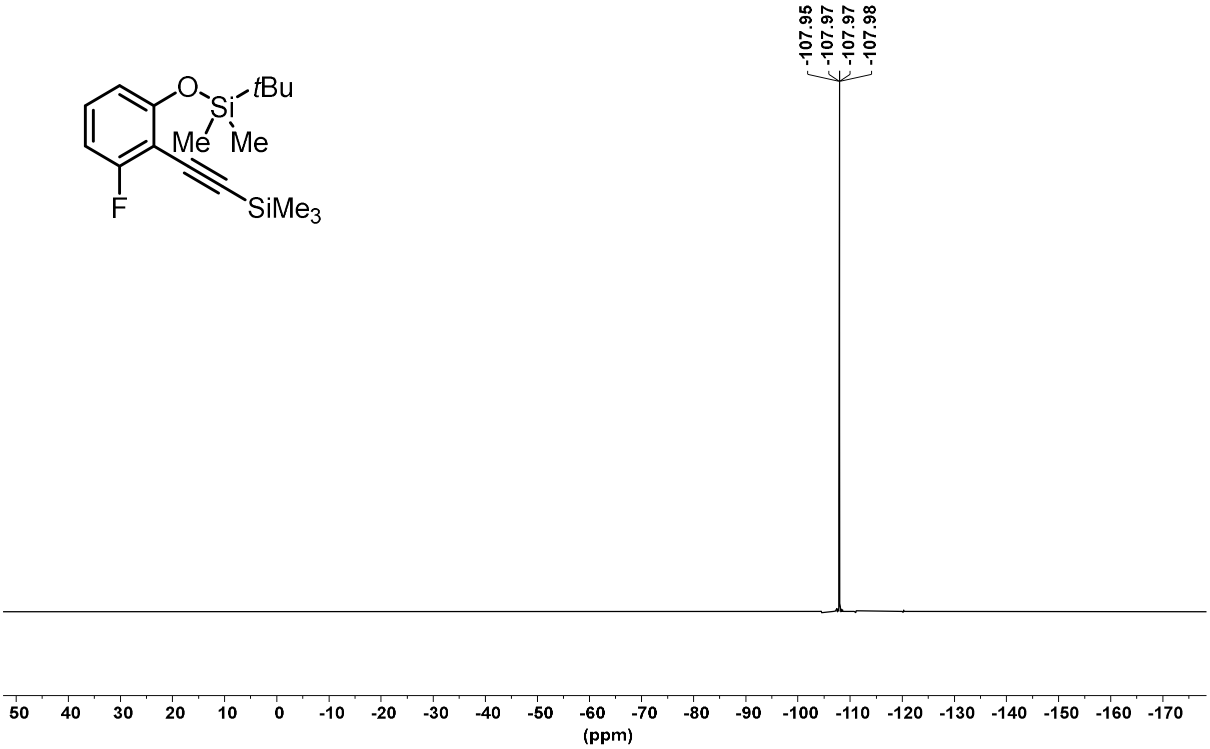

Figure S161. ^{19}F NMR spectrum of compound **15b** in $CDCl_3$.

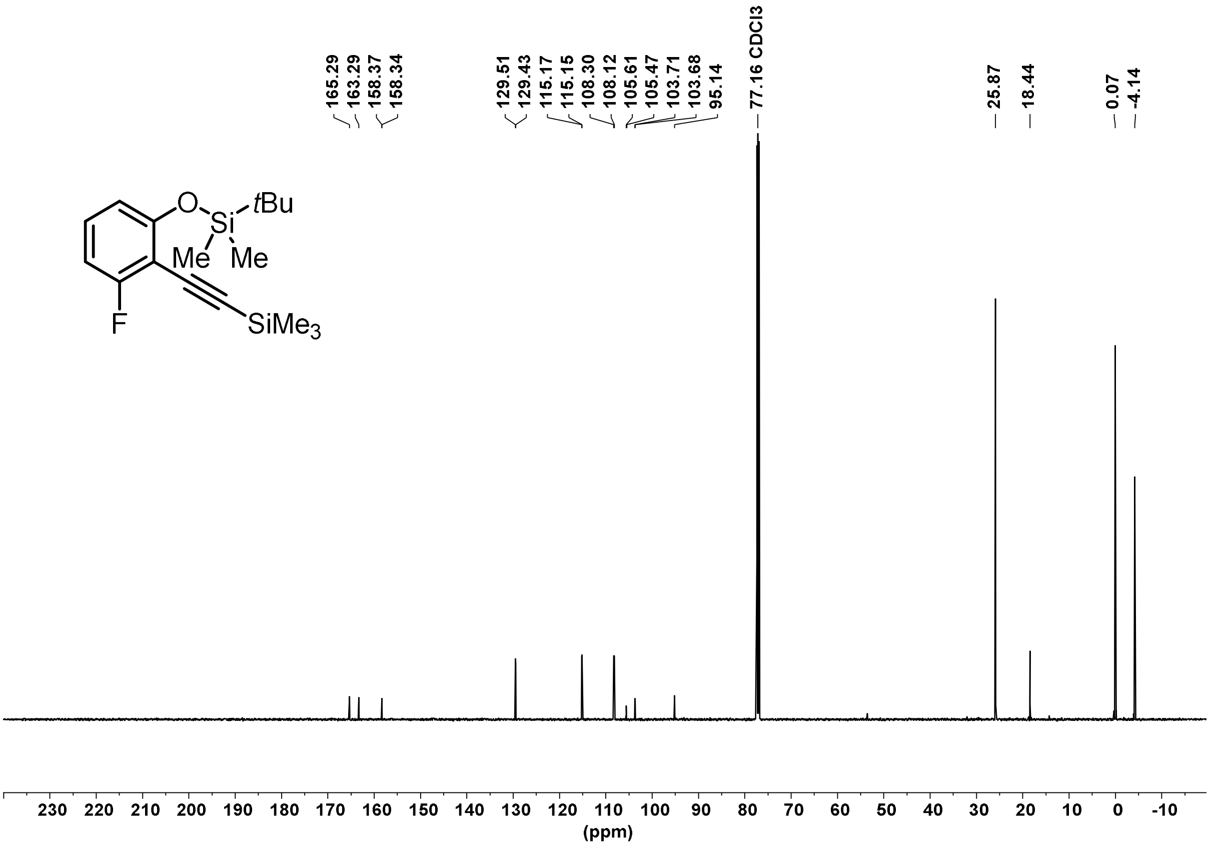

Figure S162. $^{13}C\{^1H\}$ NMR spectrum of compound **15b** in $CDCl_3$.

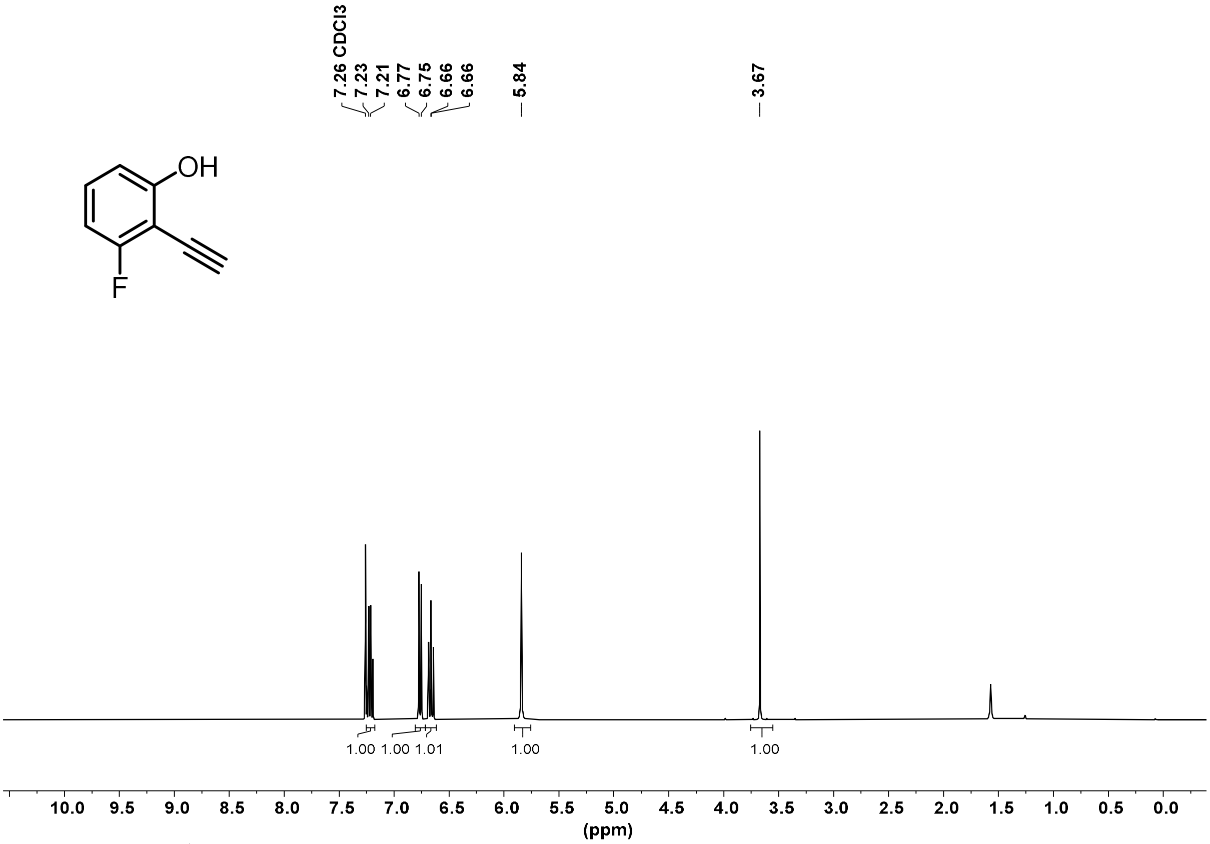

Figure S163. ^{1}H NMR spectrum of compound **1g** in $CDCl_3$.

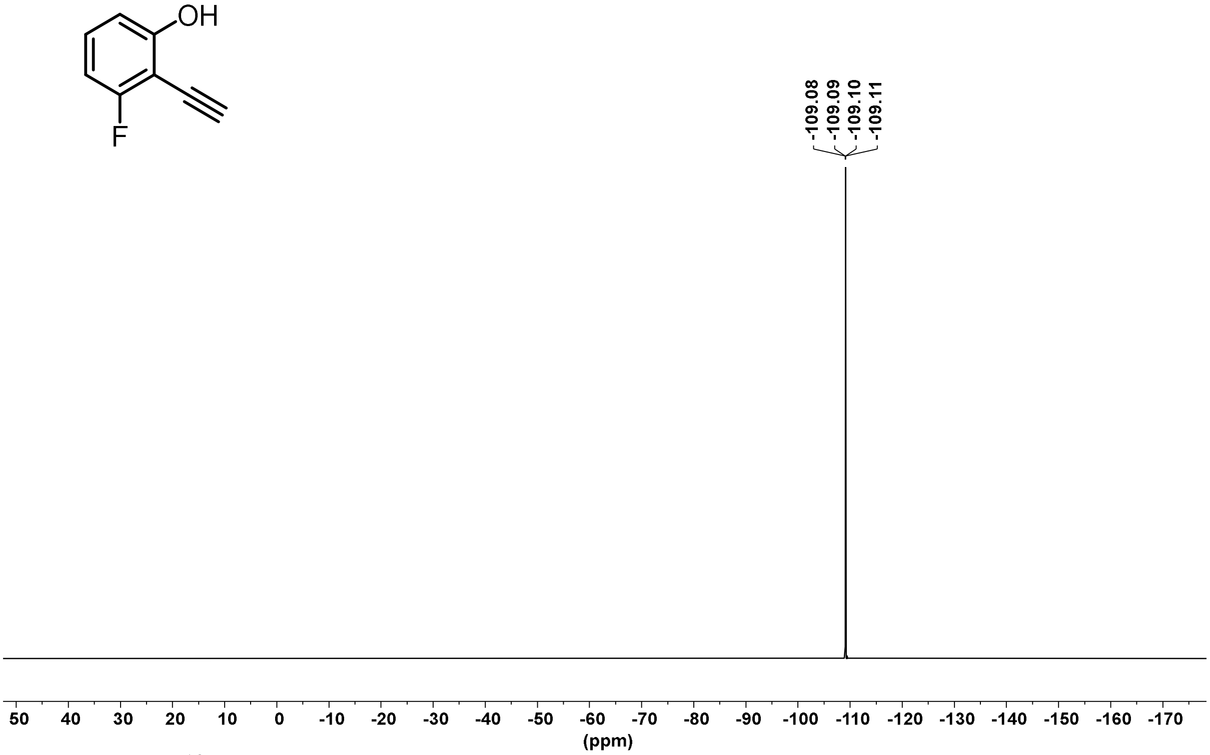

Figure S164. ^{19}F NMR spectrum of compound **1g** in $CDCl_3$.

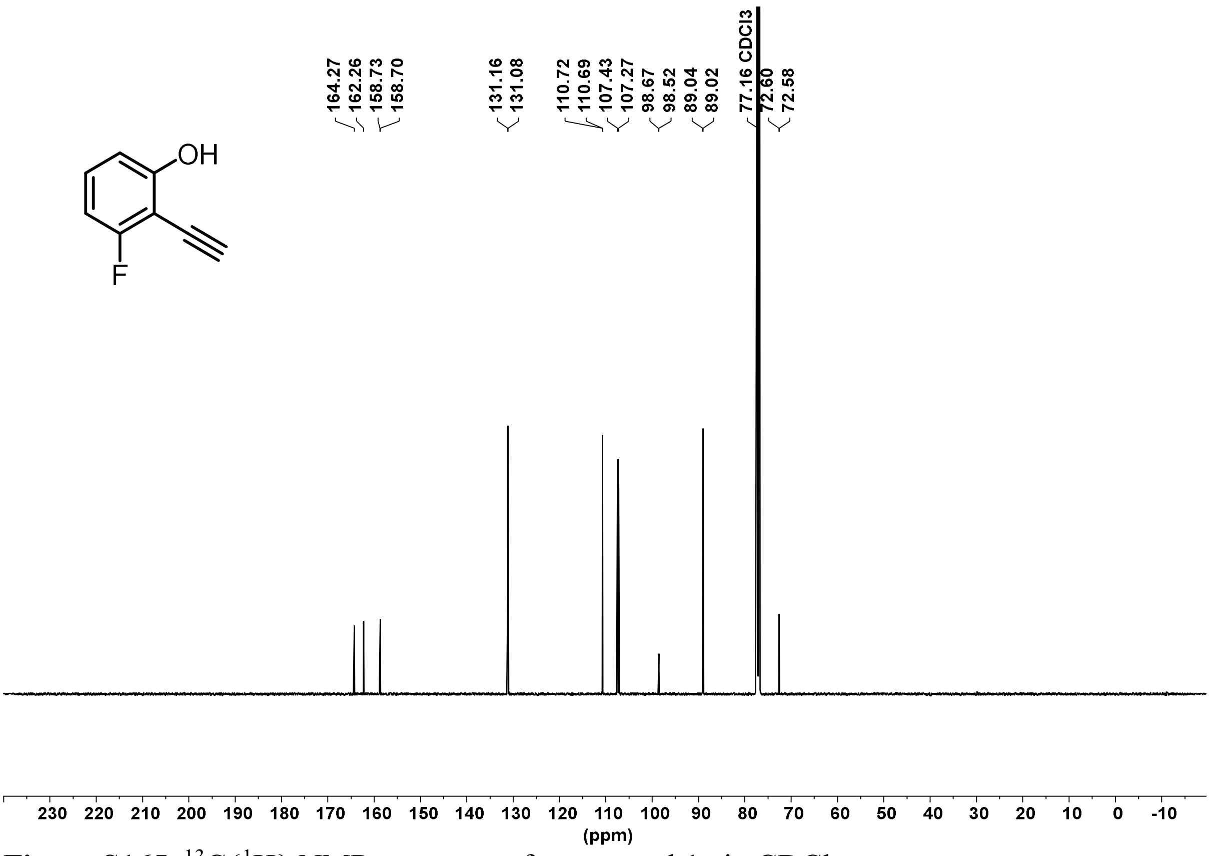

Figure S165. $^{13}C\{^{1}H\}$ NMR spectrum of compound **1g** in $CDCl_3$.

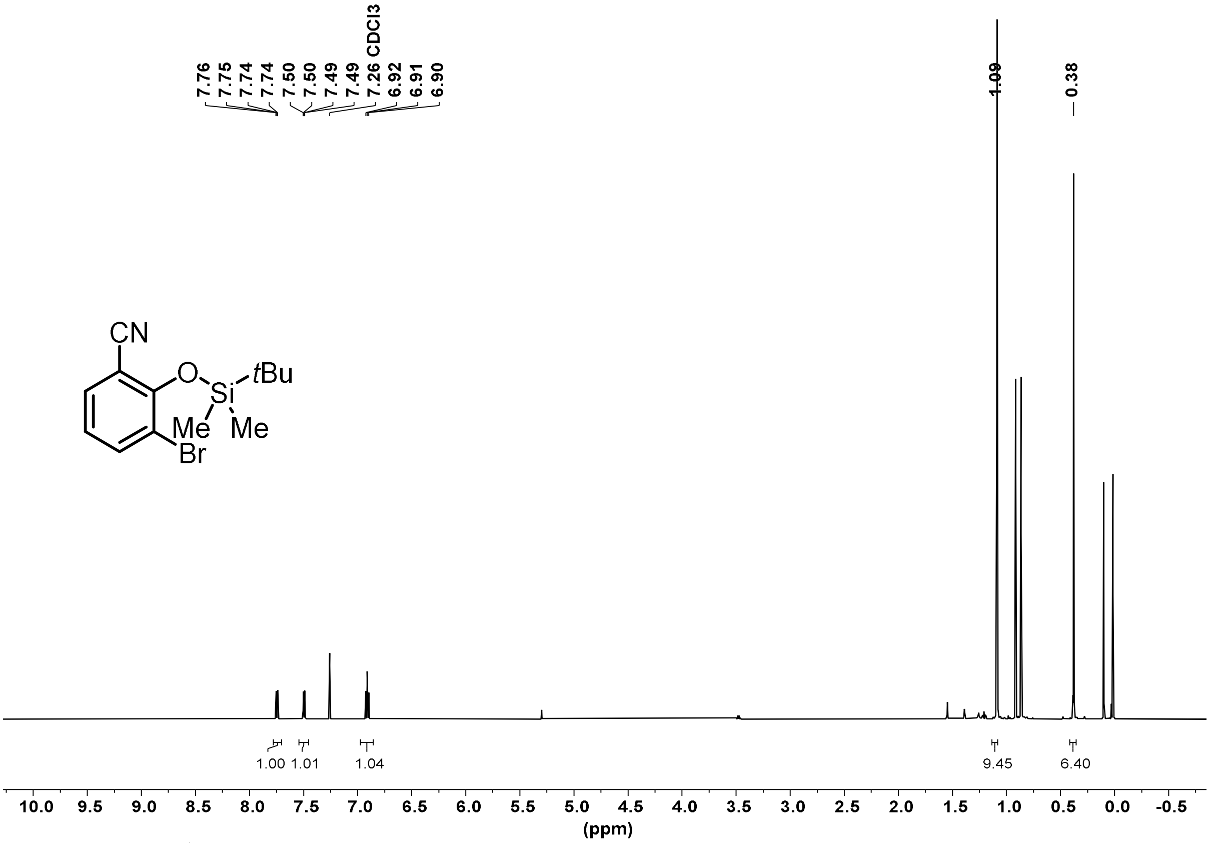

Figure S166. ^{1}H NMR spectrum of compound **14c** in $CDCl_3$.

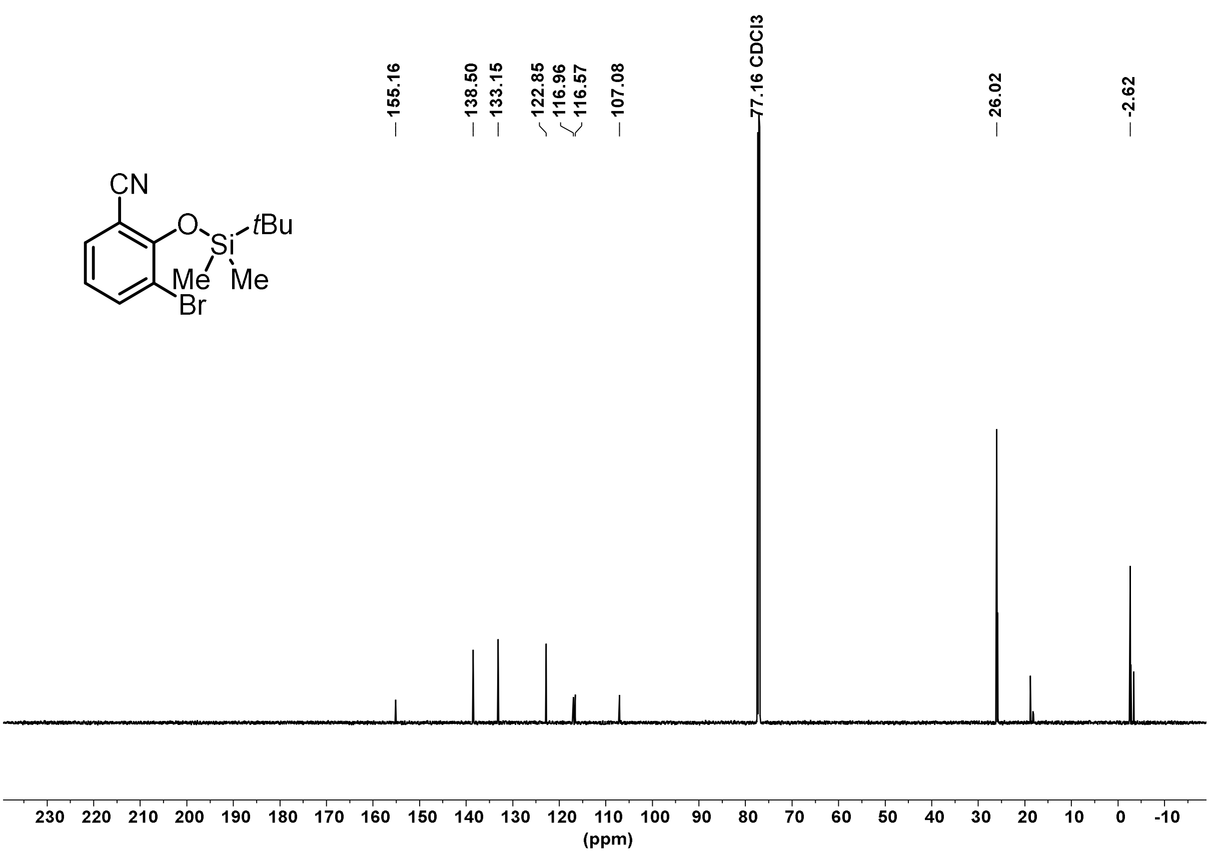

Figure S167. $^{13}C\{^{1}H\}$ NMR spectrum of compound **14c** in $CDCl_3$.

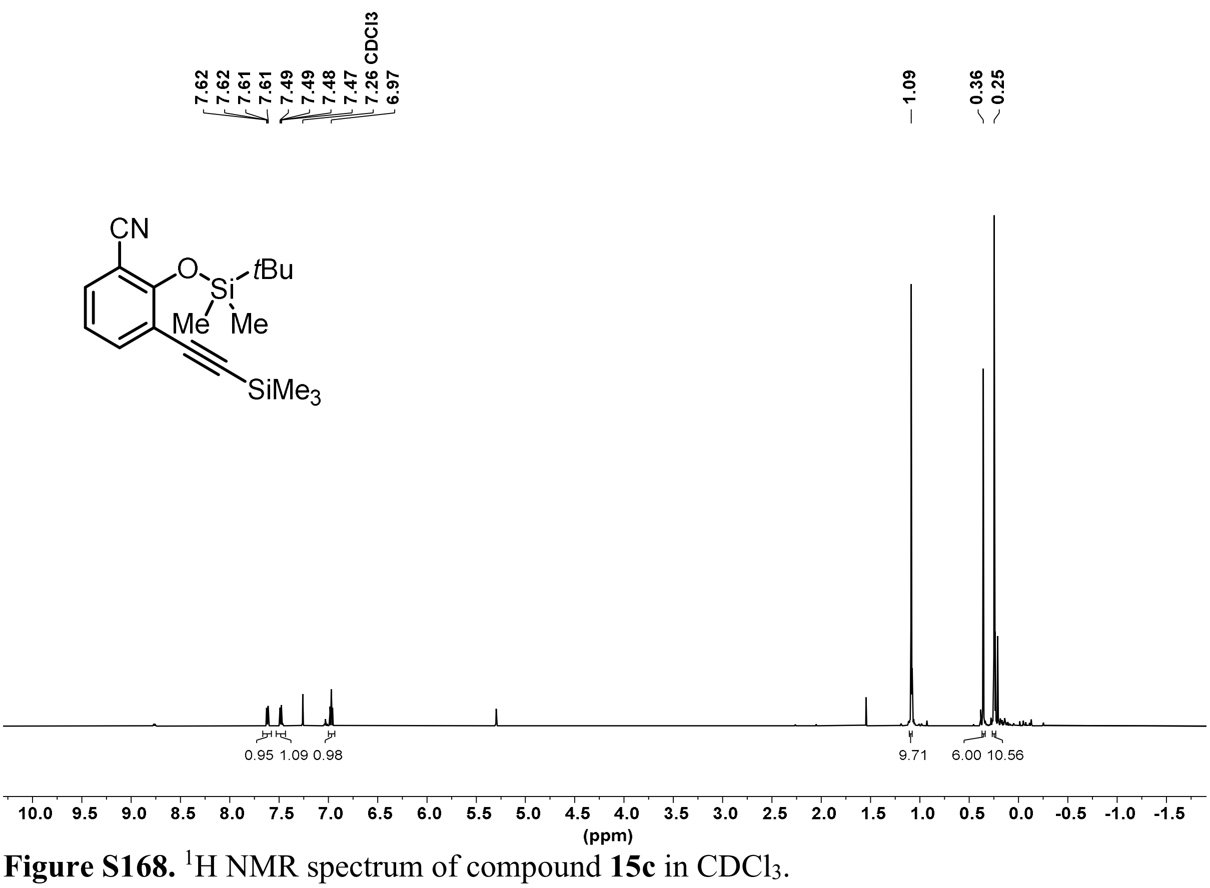

Figure S168. ^{1}H NMR spectrum of compound **15c** in $CDCl_3$.

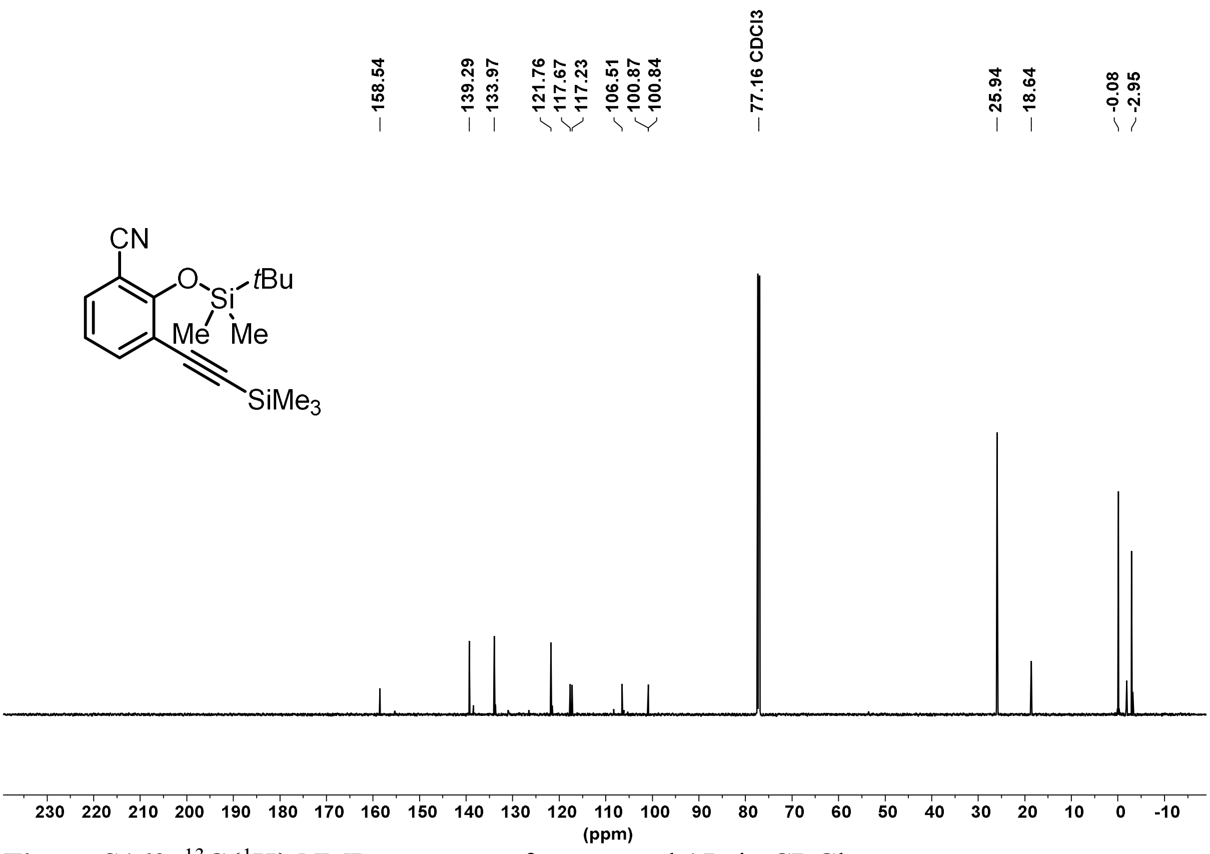

Figure S169. $^{13}C\{^1H\}$ NMR spectrum of compound **15c** in $CDCl_3$.

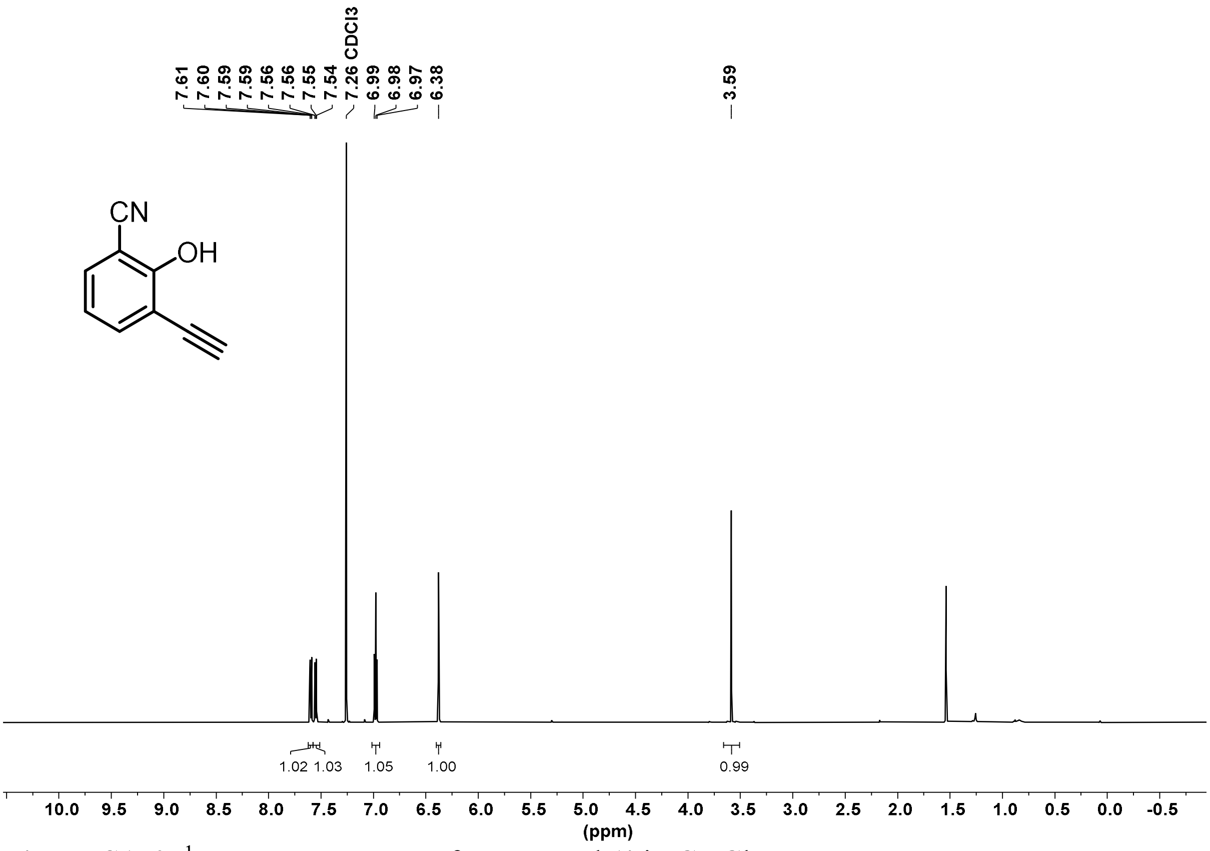

Figure S170. ^1H NMR spectrum of compound **1i** in $CDCl_3$.

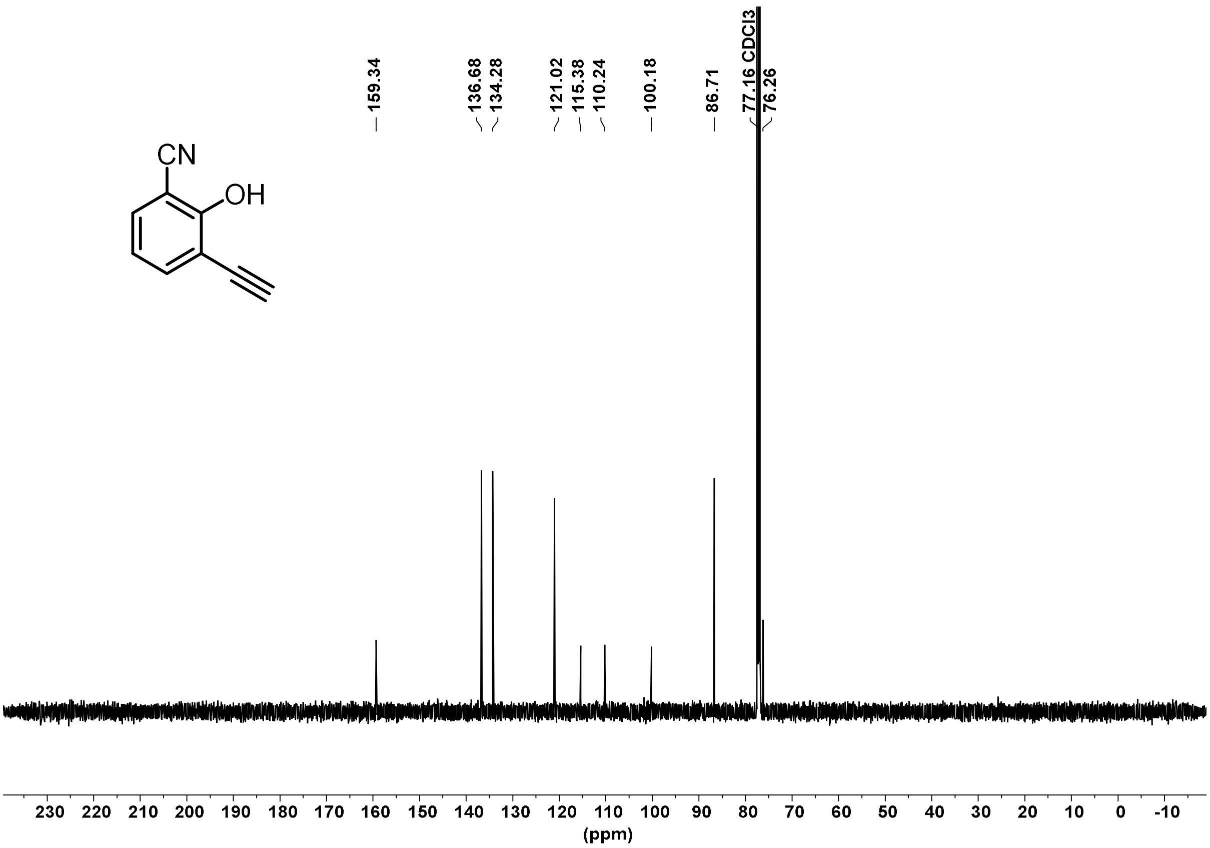

Figure S171. $^{13}C\{^1H\}$ NMR spectrum of compound **1i** in $CDCl_3$.

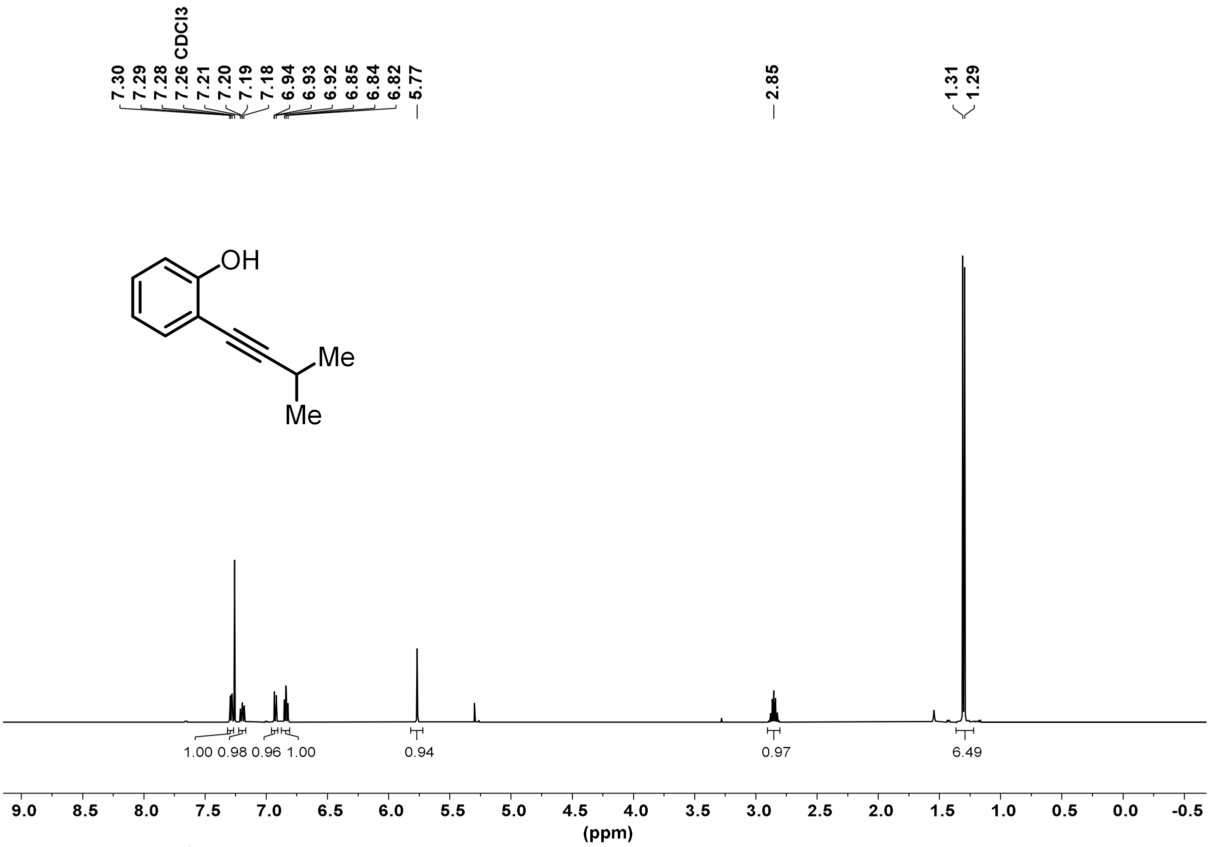

Figure S172. ^1H NMR spectrum of compound **1aa** in $CDCl_3$.

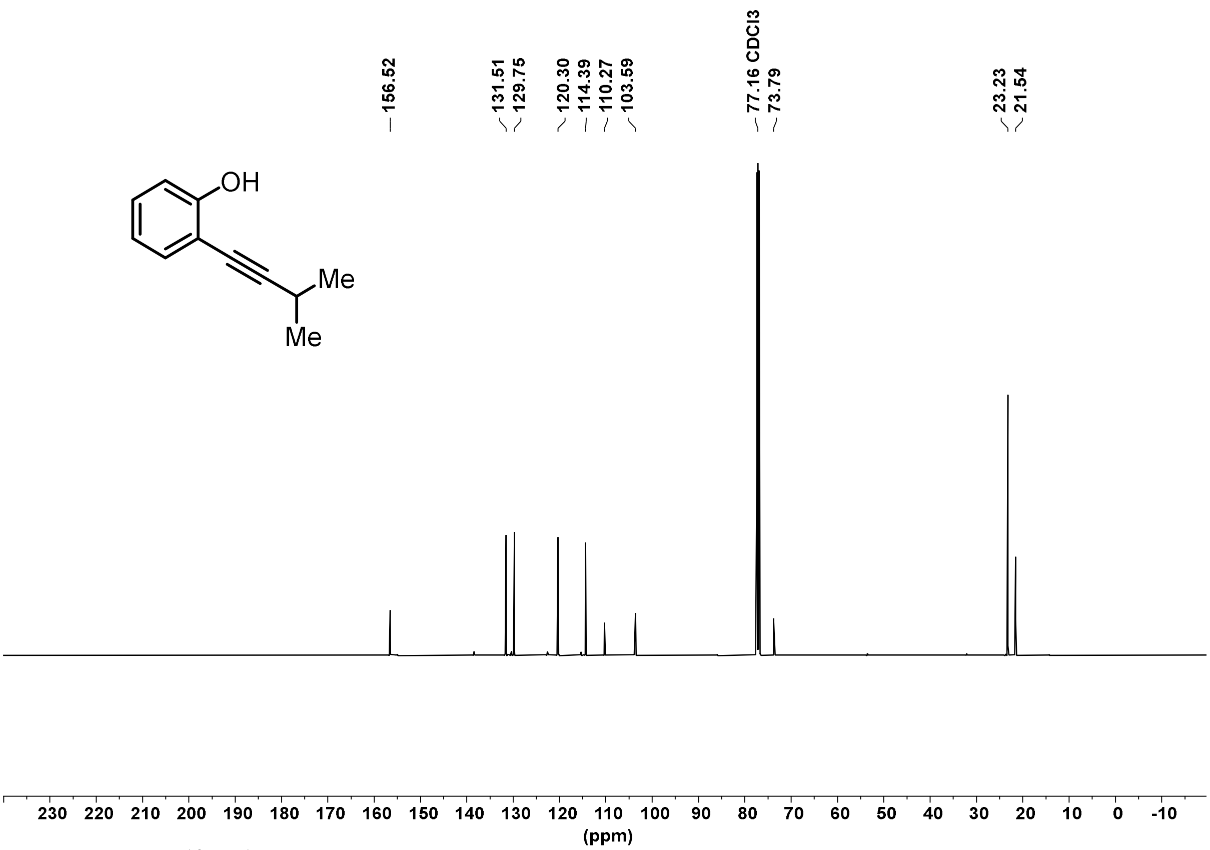

Figure S173. $^{13}C\{^{1}H\}$ NMR spectrum of compound **1aa** in $CDCl_3$.

S6. Computational Details

All calculations were performed using Orca 6.1. Automated job script generation and data processing was carried out using in-house developed Python code. Conformers for each ground-state compound were generated and ranked using the GOAT algorithm available in Orca[26] at the GFN2-xTB level of theory,[27] and the lowest energy conformer taken forward for further calculations. Geometry optimisation was carried out at the M06-2X/ma-Def2-SVP(SMD: toluene) level of theory[28,29] and frequency calculations carried out at the same level to confirm a minima (no imaginary frequencies) or saddle point (a single imaginary frequency) and obtain thermochemical corrections using the default Quasi-RRHO approach (frequency cutoff = 100 cm^{-1}) as implemented in Orca. For transition states, an initial transition state was found at the M06-2X/ma-Def2-SVP(SMD: toluene) level of theory (confirmed by a single imaginary frequency and IRC calculations). This was used as a starting point for GOAT, where the atoms involved in the imaginary frequency were frozen using the "--constrain" option and the conformational ensemble generated as above. In cases where the generated ensemble was larger than 100 structures, the 100 lowest energy structures (at the GFN2-xTB level) were taken forward. The remaining ensemble was optimised at the M06-2X/ma-Def2-SVP(SMD: toluene) level of theory with the same atoms frozen and ranked by the Gibbs free energy. Structures which did not converge within a 48-hour period were dropped. Structures within 0.1 kcal mol^{-1} were counted as rotamers, and a single structure taken forward. The ensemble was re-optimised at the same level of theory without constraints, ranked by the Gibbs free energy, and confirmed as transition states by frequency (one imaginary frequency) and IRC calculations, the lowest energy transition state was taken forward. A single-point correction was calculated at the M06-2X/ma-Def2-TZVPP(SMD: toluene) level of theory level of theory and combined with the previously calculated thermochemical corrections.

[26]B. de Souza, *Angew. Chem. Int. Ed.* 2025, **64**, e202500393.
[27]C. Bannwarth, S. Ehlert and S. Grimme, *J. Chem. Theory Comput.* 2019, **15**, 1652–167.
[28]J. Zheng, X. Xu and D. G. Truhlar, *Theor Chem Acc,* 2011, **128**, 295–305.
[29]Y. Zhao and D. G. Truhlar, *Theor Chem Account,* 2008, **120**, 215–241.

S6.1 Computational Data

Free Energy Profiles

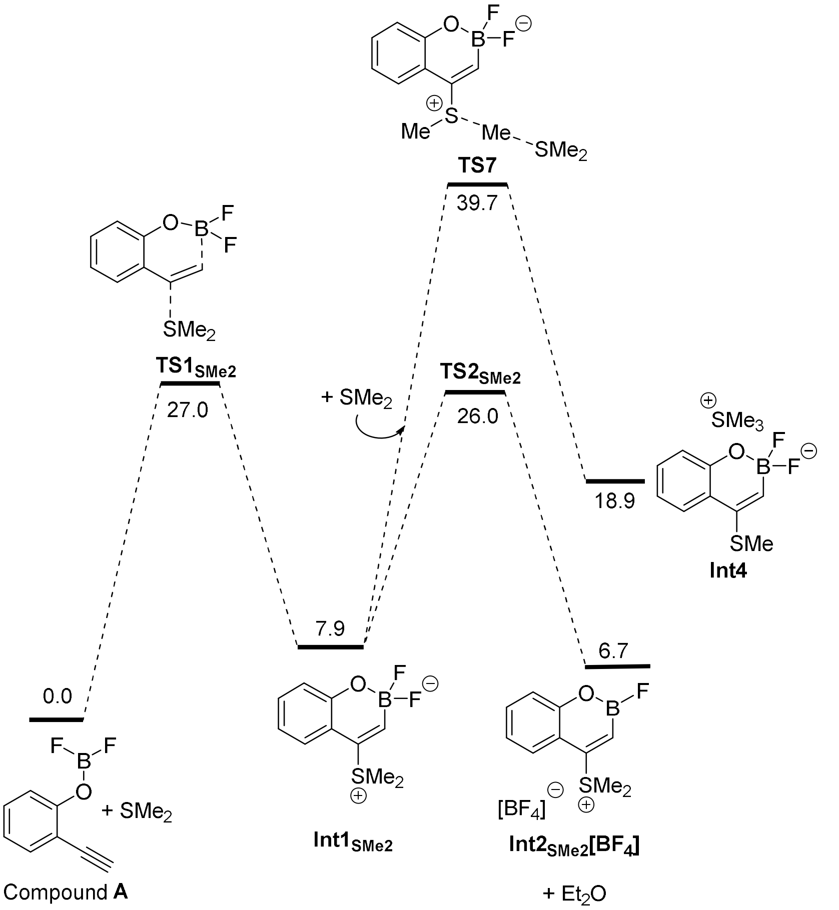

Figure S174. Formation of **B** with alternative dealkylation pathway to **Int4** (ΔG / kcal mol^{-1}).

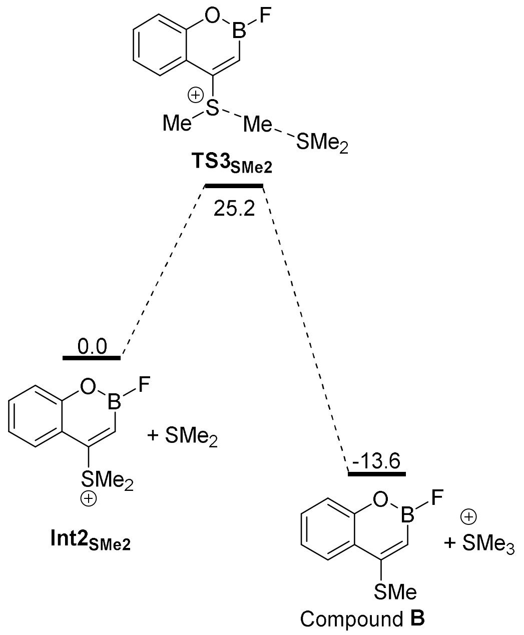

Figure S175. Methyl substitution of **Int2**$_{\mathbf{SMe2}}$ with dimethyl sulfide (ΔG / kcal mol^{-1}).

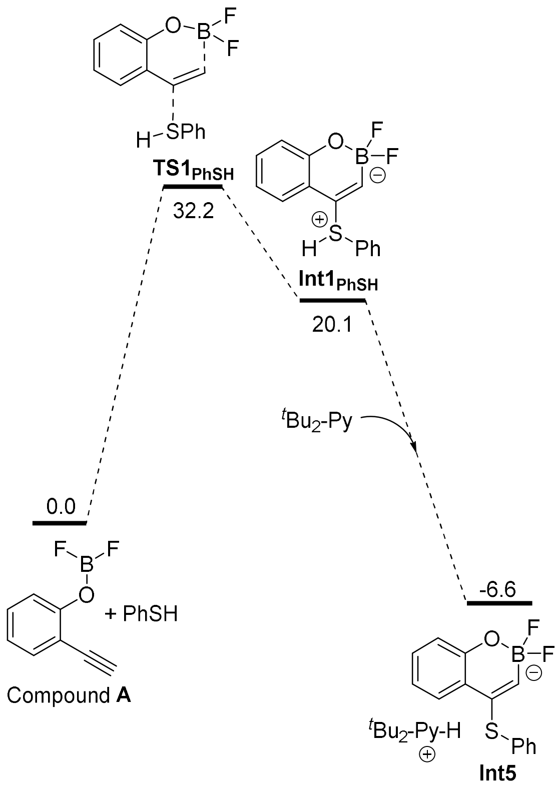

Figure S176. Direct nucleophilic attack with PhSH (ΔG / kcal mol^{-1}).

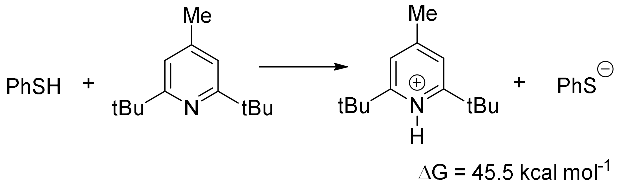

Scheme S2. Direct deprotonation of PhSH with ^{t}Bu$_2$-Py. *Note: attempted geometry optimisations of the discrete ion pair led to reformation of the starting materials.*

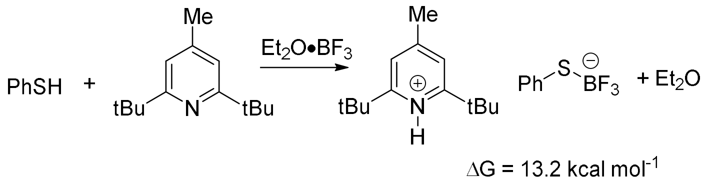

Scheme S3. Deprotonation of PhSH with Et_2O-BF_3.

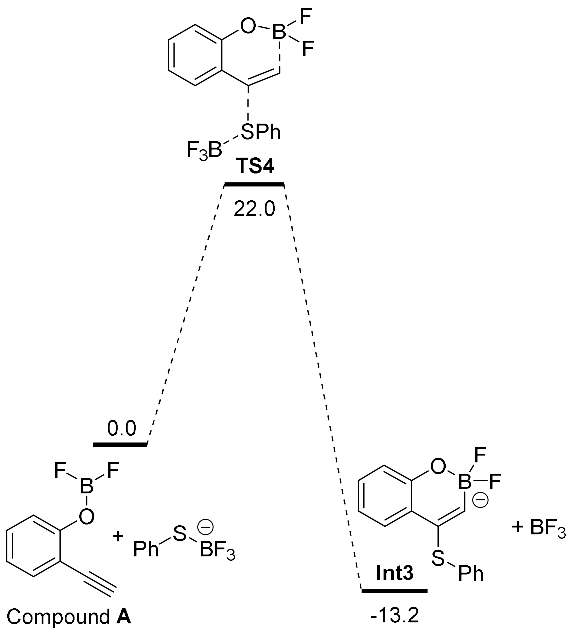

Figure S177. Nucleophilic attack with $[PhSBF_3]^-$ (ΔG / kcal mol^{-1}).

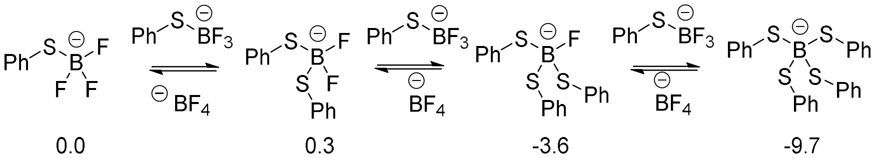

Scheme S4. Stepwise formation of $B(SPh)_4$ anion (ΔG / kcal mol^{-1}). *Note: all numbers are relative to $PhSBF_3$, rather than being for each individual step.*

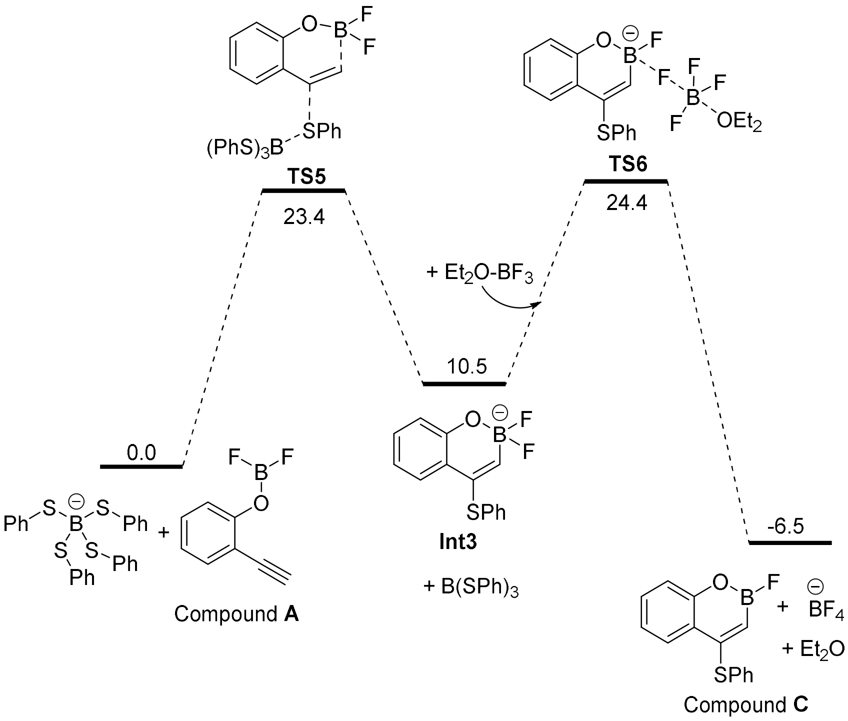

Figure S178. Formation of compound **C** with $B(SPh)_4$ anion (ΔG / kcal mol^{-1}).

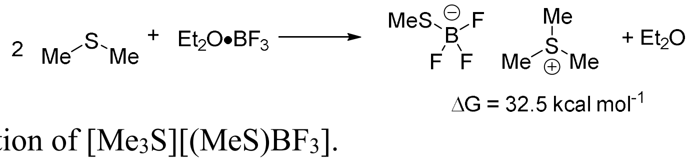

Scheme S5. Formation of $[Me_3S][(MeS)BF_3]$.

Table S14. DFT-Computed energies, thermochemical corrections, and final free energies. All values are given in Hartrees.

Compound	Energy (ma-def2-svp)	ZPE correction	Total thermal correction	Thermal enthalpy correction	Total enthalpy correction	Total free energy correction	Energy (ma-def2-tzvpp)	Final free energy (G)
$[(PhS)_2BF_2]^-$	-1483.500851	0.1947639	0.0153596	0.00094421	0.21106771	0.15340473	-1484.52051	-1484.367105
$[(PhS)_3BF]^-$	-2013.178963	0.28503452	0.02103319	0.00094421	0.30701192	0.23672301	-2014.45982	-2014.223096
$[(PhS)BF_3]^-$	-953.8271333	0.10456069	0.00990725	0.00094421	0.11541215	0.06897656	-954.5867669	-954.5177903
$[B(SPh)_4]^-$	-2542.864607	0.37542322	0.02651719	0.00094421	0.40288462	0.32250613	-2544.405106	-2544.0826
$[BF_4]^-$	-424.1571116	0.01426119	0.00437872	0.00094421	0.01958412	-0.01335582	-424.6545785	-424.6679343
$[Me_3S][(MeS)BF_3]$	-2294.191679	0.27813001	0.02244036	0.00094421	0.30151458	0.22967197	-2295.202015	-2294.972343
$[PhS]^-$	-629.5472303	0.09059687	0.00552159	0.00094421	0.09706267	0.06081557	-629.9276474	-629.8668318
$[SMe_3]^+$	-517.4167852	0.11538972	0.00655966	0.00094421	0.12289359	0.08613787	-517.6989795	-517.6128417
$[^t\text{Bu}_2\text{-Py-H}]^+$	-601.8386988	0.355717	0.0169046	0.00094421	0.37356581	0.31440811	-602.5078381	-602.19343
$[^t\text{Bu}_2\text{-Py-H}][(PhS)BF_3]$	-1555.726714	0.46107953	0.02866751	0.00094421	0.49069125	0.40762337	-1557.150075	-1556.742452
$B(SPh)_3$	-1913.266083	0.28356055	0.01970313	0.00094421	0.30420789	0.23708689	-1914.434635	-1914.197548
BF_3	-324.2253302	0.01240422	0.00351695	0.00094421	0.01686538	-0.01375398	-324.6091112	-324.6228652
Compound **A**	-607.0810252	0.11563033	0.0101065	0.00094421	0.12668104	0.08010977	-607.7657617	-607.6856519
Compound **B**	-945.2801384	0.15580211	0.01101113	0.00094421	0.16775745	0.11914052	-946.0216076	-945.9024671
Compound **C**	-1136.812162	0.20926229	0.01414996	0.00094421	0.22435646	0.16881089	-1137.755971	-1137.58716
Et_2O	-233.3732116	0.13699659	0.00675874	0.00094421	0.14469954	0.10718262	-233.6432768	-233.5360942
$Et_2O\text{-}BF_3$	-557.6388745	0.15342678	0.01040685	0.00094421	0.16477784	0.11829255	-558.2863741	-558.1680815
**Int1**$_{\mathbf{PhSH}}$	-1237.124191	0.22035439	0.01571078	0.00094421	0.23700938	0.17836971	-1238.189049	-1238.010679
**Int1**$_{\mathbf{SMe2}}$	-1084.868173	0.19581565	0.01433747	0.00094421	0.21109733	0.15523684	-1085.776121	-1085.620885
**Int2**$_{\mathbf{SMe2}}$	-984.9090865	0.19471633	0.0129889	0.00094421	0.20864944	0.15614136	-985.7018579	-985.5457165
**Int2**$_{\mathbf{SMe2}}$**[BF$_4$]**	-1409.134347	0.21101322	0.01854228	0.00094421	0.23049971	0.16676315	-1410.421606	-1410.254843
**Int3**	-1236.712464	0.21046313	0.01535421	0.00094421	0.22676155	0.16881772	-1237.770361	-1237.601544
**Int4**	-1562.659284	0.27481399	0.02000333	0.00094421	0.29576153	0.22878548	-1563.792665	-1563.563879
**Int5**	-1838.616223	0.56715191	0.03408833	0.00094421	0.60218445	0.50775563	-1840.336763	-1839.829007
PhSH	-630.041615	0.10019895	0.00622008	0.00094421	0.10736324	0.06973018	-630.427636	-630.3579059
SMe_2	-477.7682846	0.07610863	0.00477321	0.00094421	0.08182605	0.04925164	-477.9971253	-477.9478736

tBu$_2$-Py	-601.4069956	0.34093465	0.01700561	0.00094421	0.35888447	0.29929176	-602.0741473	-601.7748556
**TS1$_{PhSH}$**	-1237.100456	0.21651189	0.01653725	0.00094421	0.23399335	0.17318486	-1238.165354	-1237.99217
**TS1$_{SMe2}$**	-1084.831419	0.19165859	0.01526952	0.00094421	0.20787232	0.15001109	-1085.740519	-1085.590508
**TS2$_{SMe2}$**	-1642.502156	0.34876039	0.02623063	0.00094421	0.37593523	0.29685486	-1644.056954	-1643.760099
**TS3$_{SMe2}$**	-1462.659297	0.2715385	0.01916282	0.00094421	0.29164553	0.22549395	-1463.678853	-1463.453359
**TS4**	-1560.901732	0.21998257	0.02079572	0.00094421	0.2417225	0.17226395	-1562.340634	-1562.16837
**TS5**	-3149.942109	0.49231201	0.036977	0.00094421	0.53023322	0.42959364	-3152.158578	-3151.728984
**TS6**	-1794.345136	0.36402318	0.02743934	0.00094421	0.39240673	0.31040468	-1796.050331	-1795.739926
**TS7**	-1562.610483	0.27301541	0.02003655	0.00094421	0.29399617	0.22630469	-1563.744345	-1563.51804

S7. Crystallographic Details

All crystallographic data was collected on a XtaLAB Synergy-S diffractometer with a HyPix-Arc 100 detector at the University of Edinburgh. Data collections were recorded at T = 100.0(10) K using an Oxford Cryosystems Cryostream 1000 utilising liquid nitrogen boiloff.[30] Diffraction images from raw frame data were reduced using CryAlisPro. Structures were solved using SHELXT and refined to convergence using SHELXL through the OLEX2 GUI using full matrix least squares minimisation on *F2*.[31,32,33,34] Non-hydrogen atoms were refined anisotropically, and hydrogen atoms were placed geometrically, allowed to ride upon their parent atoms. The crystallographic data is summarised in **Tables S15-S18** and full details are given in the supplementary deposited CIF file (CCDC 2525929-2525932). This can be obtained free of charge from the Cambridge Crystallographic Data Centre.

S7.1 Crystallographic Data and Experimental

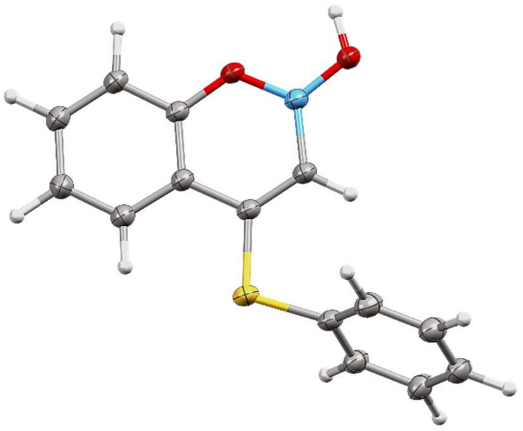

Experimental. Single clear colourless plate-shaped crystals of **2e** recrystallised from pentane by solvent layering. A suitable crystal with dimensions 0.14 × 0.09 × 0.01 mm^3 was selected and mounted on a MITIGEN holder in Paratone oil. on a XtaLAB Synergy R, HyPix-Arc 100 diffractometer. The crystal was kept at a steady T = 100.00(10) K during data collection. The structure was solved with the olex2.solve 1.5-beta (Bourhis et al., 2015) solution program using iterative methods and by using Olex2 1.5-beta (Dolomanov et al., 2009) as the graphical interface. The model was refined with olex2.refine 1.5-beta (Bourhis et al., 2015) using full matrix least squares minimisation on $\boldsymbol{F^2}$.

Crystal Data. $C_{14}H_{11}BO_2S$, M_r = 254.132, triclinic, P-1 (No. 2), a = 4.48173(15) Å, b = 11.0812(3) Å, c = 12.3982(3) Å, a = 104.199(2)°, b = 91.551(2)°, g = 96.291(3)°, V = 592.39(3) Å^3, T = 100.00(10) K, Z = 2, Z' = 1, m(Cu K_a) = 2.325, 12190 reflections measured, 2455 unique (R_{int} = 0.0471) which were used in all calculations. The final wR_2 was 0.0954 (all

---

[30] J. Cosier and A. M. Glazer, *J. Appl. Cryst.*, 1986, **19**, 105–107.
[31] G. M. Sheldrick, *Acta Cryst. C Struct Chem*, 2015, **71**, 3–8.
[32] G. M. Sheldrick, *Acta Cryst. A*, 2008, **64**, 112–122.
[33] O. V. Dolomanov, L. J. Bourhis, R. J. Gildea, J. A. K. Howard and H. Puschmann, *J. Appl. Cryst.*, 2009, **42**, 339–341.
[34] D. Kratzert and I. Krossing, *J. Appl. Cryst.*, 2018, **51**, 928–934.

data) and R_1 was 0.0356 (I≥2 s(I)).

Table S15.

**Compound**	**2e**
CCDC Number	2525929
Formula	$C_{14}H_{11}BO_2S$
$D_{calc.}$/ g cm^{-3}	1.425
μ/mm^{-1}	2.325
Formula Weight	254.132
Colour	clear colourless
Shape	plate-shaped
Size/mm^3	0.14×0.09×0.01
T/K	100.00(10)
Crystal System	triclinic
Space Group	P-1
a/Å	4.48173(15)
b/Å	11.0812(3)
c/Å	12.3982(3)
α/°	104.199(2)
β/°	91.551(2)
γ/°	96.291(3)
V/Å^3	592.39(3)
Z	2
Z'	1
Wavelength/Å	1.54184
Radiation type	Cu K_α
Θ_{min}/°	3.68
Θ_{max}/°	77.27
Measured Refl's.	12190
Indep't Refl's	2455
Refl's I≥2 σ(I)	2136
R_{int}	0.0471
Parameters	262
Restraints	0
Largest Peak	0.3933
Deepest Hole	-0.3320
GooF	1.0476
wR_2 (all data)	0.0954
wR_2	0.0921
R_1 (all data)	0.0408
R_1	0.0356

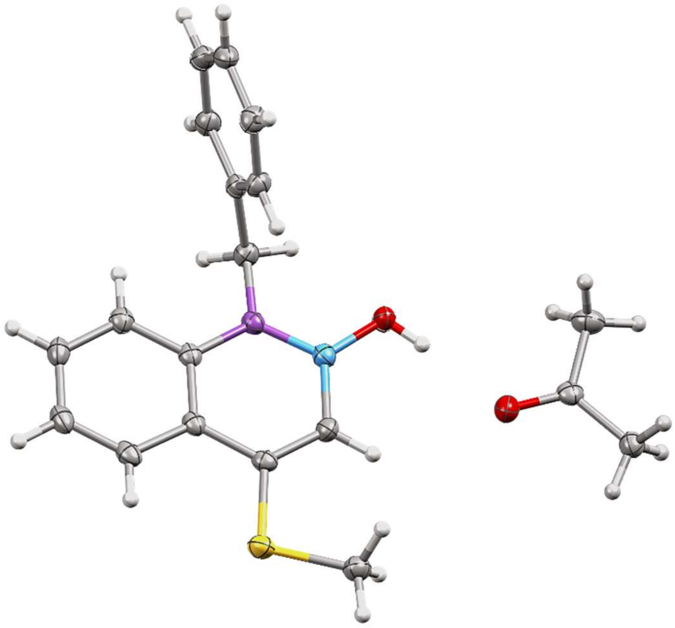

Experimental. Single colourless block-shaped crystals of **4a** were used as supplied. A suitable crystal with dimensions 0.36 × 0.29 × 0.19 mm was selected and mounted on a XtaLAB Synergy, Single source at home/near, HyPix-Arc 100 diffractometer. The crystal was kept at a steady *T* = 102(3) K during data collection. The structure was solved with the ShelXT 2018/2 (Sheldrick, 2018) solution program using dual methods and by using Olex2 1.5-beta (Dolomanov et al., 2009) as the graphical interface. The model was refined with olex2.refine 1.5-beta (Bourhis et al., 2015) using full matrix least squares minimisation on $|\boldsymbol{F}|^2$.

Crystal Data. $C_{19}H_{16}BD_6NO_2S$, M_r = 345.319, orthorhombic, *Pbca* (No. 61), a = 13.6683(3) Å, b = 14.1884(3) Å, c = 18.3110(4) Å, *a* = *b* = *g* = 90°, *V* = 3551.08(13) Å^3, *T* = 102(3) K, *Z* = 8, *Z'* = 1, *m*(Mo K$_a$) = 0.193, 18433 reflections measured, 4318 unique (R_{int} = 0.0211) which were used in all calculations. The final wR_2 was 0.0766 (all data) and R_1 was 0.0302 (I≥2*s*(I)).

Table S16.

**Compound**	**4a**
CCDC Number	2525930
Formula	$C_{19}H_{16}BD_6NO_2S$
$D_{calc.}$/ g cm^{-3}	1.292
μ/mm^{-1}	0.193
Formula Weight	345.319
Colour	colourless
Shape	block-shaped
Size/mm	0.36×0.29×0.19
T/K	102(3)
Crystal System	orthorhombic
Space Group	*Pbca*
a/Å	13.6683(3)
b/Å	14.1884(3)
c/Å	18.3110(4)
α/°	90
β/°	90
γ/°	90
V/Å^3	3551.08(13)
Z	8
Z'	1
Wavelength/Å	0.71073
Radiation type	Mo K_α
Θ_{min}/°	2.35
Θ_{max}/°	30.80
Index range h	-16 ≥ h ≥ 17
Index range k	-18 ≥ k ≥ 16
Index range l	-20 ≥ l ≥ 23
Measured Refl's.	18433
Indep't Refl's	4318
Refl's I≥2σ(I)	3720
R_{int}	0.0211
Parameters	399
Restraints	0
Largest Peak/eÅ^3	0.3584
Deepest Hole/eÅ^3	-0.2777
GooF	1.1062
R_1 (I≥2σ(I) / all)	0.0302 / 0.0387
wR_2 (I≥2σ(I) / all)	0.0724 / 0.0766

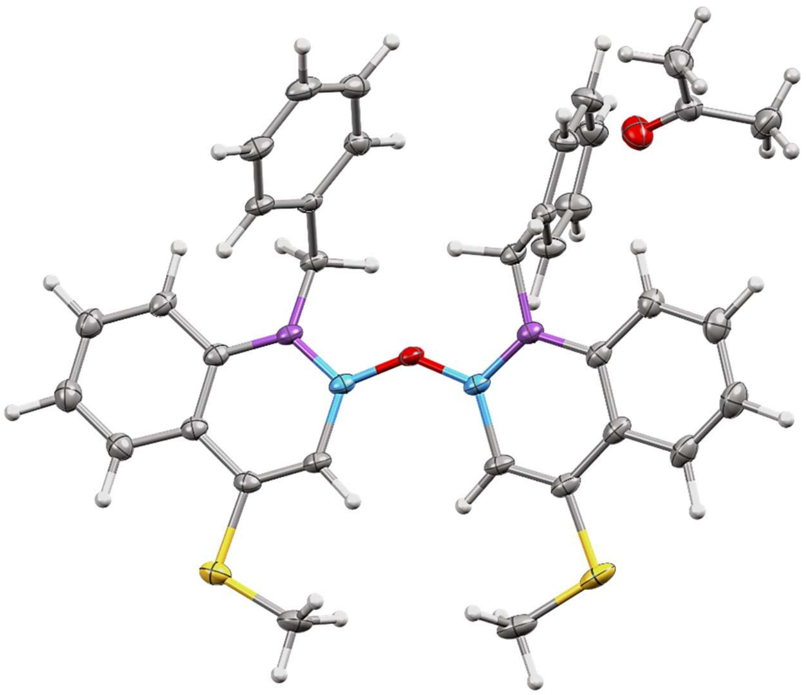

Experimental. Single colourless plate-shaped crystals of **4a-anhydride** were used as supplied. A suitable crystal with dimensions 0.18 × 0.07 × 0.02 mm was selected and mounted on a XtaLAB Synergy, Single source at home/near, HyPix-Arc 100 diffractometer. The crystal was kept at a steady *T* = 100.00(10) K during data collection. The structure was solved with the olex2.solve 1.5-beta (Bourhis et al., 2015) solution program using iterative methods and by using Olex2 1.5-beta (Dolomanov et al., 2009) as the graphical interface. The model was refined with olex2.refine 1.5-beta (Bourhis et al., 2015) using full matrix least squares minimisation on $|F|^2$.

Crystal Data. $C_{35}H_{30}B_2D_6N_2O_2S_2$, M_r = 608.506, monoclinic, $P2_1/n$ (No. 14), a = 8.3828(2) Å, b = 13.5290(3) Å, c = 27.3128(9) Å, *b* = 96.010(3)°, *a* = *g* = 90°, *V* = 3080.54(14) Å^3, *T* = 100.00(10) K, *Z* = 4, *Z'* = 1, *m*(Mo K_a) = 0.209, 21262 reflections measured, 7086 unique (R_{int} = 0.0465) which were used in all calculations. The final wR_2 was 0.0908 (all data) and R_1 was 0.0492 (I≥2*s*(I)).

Table S17.

**Compound**	**4a-anhydride**
CCDC Number	2525932
Formula	$C_{35}H_{30}B_2D_6N_2O_2S_2$
$D_{calc.}$/ g cm^{-3}	1.312
μ/mm^{-1}	0.209
Formula Weight	608.506
Colour	colourless
Shape	plate-shaped
Size/mm	0.18×0.07×0.02
T/K	100.00(10)
Crystal System	monoclinic
Space Group	$P2_1/n$
a/Å	8.3828(2)
b/Å	13.5290(3)
c/Å	27.3128(9)
α/°	90
β/°	96.010(3)
γ/°	90
V/Å^3	3080.54(14)
Z	4
Z'	1
Wavelength/Å	0.71073
Radiation type	Mo K_α
Θ_{min}/°	2.12
Θ_{max}/°	28.28
Index range h	-9 ≥ h ≥ 11
Index range k	-17 ≥ k ≥ 14
Index range l	-33 ≥ l ≥ 37
Measured Refl's.	21262
Indep't Refl's	7086
Refl's I≥2σ(I)	5506
R_{int}	0.0465
Parameters	696
Restraints	221
Largest Peak/eÅ^3	0.5391
Deepest Hole/eÅ^3	-0.4582
GooF	1.1105
R_1 (I≥2σ(I) / all)	0.0492 / 0.0746
wR_2 (I≥2σ(I) / all)	0.0841 / 0.0908

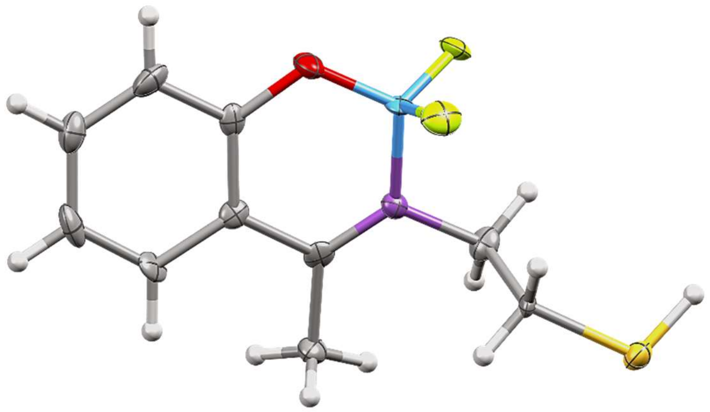

Experimental. Single clear colourless plate-shaped crystals of **6b**. Layering with toluene and hexane. A suitable crystal with dimensions 0.10 × 0.07 × 0.02 mm was selected and Fomblin oil on a XtaLAB Synergy, Single source at home/near, HyPix-Arc 100 diffractometer. The crystal was kept at a steady T = 100.00(10) K during data collection. The structure was solved with the ShelXT 2018/2 (Sheldrick, 2018) solution program using dual methods and by using Olex2 1.5-beta (Dolomanov et al., 2009) as the graphical interface. The model was refined with ShelXL 2025/1 (Sheldrick, 2015) using full matrix least squares minimisation on $|\boldsymbol{F}|^2$.

Crystal Data. $C_{10}H_{12}BF_2NOS$, M_r = 243.08, orthorhombic, $Pca2_1$ (No. 29), a = 8.9422(6) Å, b = 11.3738(5) Å, c = 21.3342(15) Å, $a = b = g = 90^{\circ}$, V = 2169.8(2) Å^3, T = 100.00(10) K, Z = 8, Z' = 2, m(Mo K_a) = 0.301, 9783 reflections measured, 3594 unique (R_{int} = 0.0698) which were used in all calculations. The final wR_2 was 0.1647 (all data) and R_1 was 0.0706 (I≥2s(I)).

Table S18.

**Compound**	**6b**
CCDC Number	2525931
Formula	$C_{10}H_{12}BF_2NOS$
$D_{calc.}$/ g cm^{-3}	1.488
μ/mm^{-1}	0.301
Formula Weight	243.08
Colour	clear colourless
Shape	plate-shaped
Size/mm	0.10×0.07×0.02
T/K	100.00(10)
Crystal System	orthorhombic
Flack Parameter	0.4(2)
Hooft Parameter	-0.03(10)
Space Group	$Pca2_1$
a/Å	8.9422(6)
b/Å	11.3738(5)
c/Å	21.3342(15)
α/°	90
β/°	90
γ/°	90
V/Å^3	2169.8(2)
Z	8
Z'	2
Wavelength/Å	0.71073
Radiation type	Mo K_α
Θ_{min}/°	2.618
Θ_{max}/°	25.348
Index range h	-10 ≥ h ≥ 9
Index range k	-13 ≥ k ≥ 13
Index range l	-23 ≥ l ≥ 25
Measured Refl's.	9783
Indep't Refl's	3594
Refl's I≥2σ(I)	2892
R_{int}	0.0698
Parameters	294
Restraints	13
Largest Peak/eÅ^3	0.947
Deepest Hole/eÅ^3	-0.266
GooF	1.081
R_1 (I≥2σ(I) / all)	0.0706 / 0.0893
wR_2 (I≥2σ(I) / all)	0.1561 / 0.1647
